# Boron trifluoride-mediated domino dehydration/electrophilic cyclization of alkynols: mechanistic insights and origin of stereoselectivity

**DOI:** 10.1039/d6ra05267c

**Published:** 2026-08-07

**Authors:** Takashi Okitsu, Takeshi Yoshikawa, Yuka Takayori, Syuto Hirata, Masanari Shinoki, Momoka Ito, Ken Sakata, Manabu Hatano

**Affiliations:** a Faculty of Pharmaceutical Sciences, University of Toyama Sugitani Toyama 930-0194 Japan okitsu@pha.u-toyama.ac.jp; b Faculty of Pharmaceutical Sciences, Toho University Funabashi Chiba 274-8510 Japan takeshi.yoshikawa@phar.toho-u.ac.jp; c Faculty of Pharmaceutical Sciences, Kobe Pharmaceutical University Kobe 658-8558 Japan

## Abstract

The mechanistic origin of substituent-dependent stereoselectivity in BF_3_·OEt_2_-mediated domino dehydration/electrophilic cyclization of alkynols was investigated through complementary experimental and computational studies. BF_3_·OH_2_, generated *in situ*, was found to function as the Brønsted acid responsible for initiating electrophilic cyclization. Comparison of silylated and non-silylated substrates by density functional theory calculations revealed that removal of the β-silyl effect shifts the rate-determining step along competing reaction pathways, thereby accounting for the observed *E*/*Z* stereoselectivity. These findings contribute to a better understanding of Brønsted acid-mediated electrophilic cyclization of alkynes.

## Abstract 2

BF_3_·OH_2_-mediated electrophilic cyclization enables tricyclic benzofulvene synthesis with substituent-controlled stereoselectivity.

## Graphical abstract

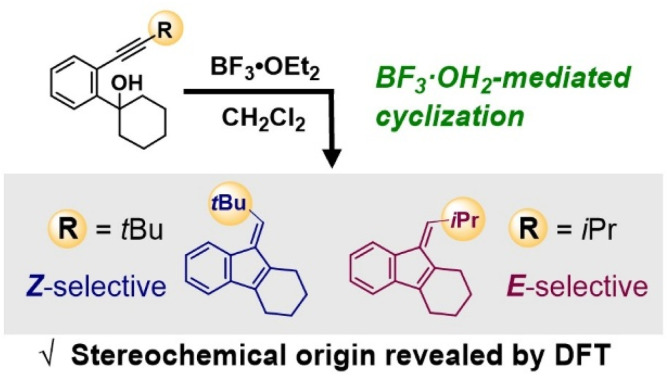

## 1 Introduction

Electrophilic cyclization of alkynes constitutes a powerful strategy for the construction of cyclic frameworks and typically relies on activation by iodine^1–6^ and gold catalysts.^7,8^ In contrast, Brønsted acid-promoted electrophilic cyclizations remain underdeveloped despite their potential advantages in operational simplicity and cost efficiency.^9^

**Scheme 1:**
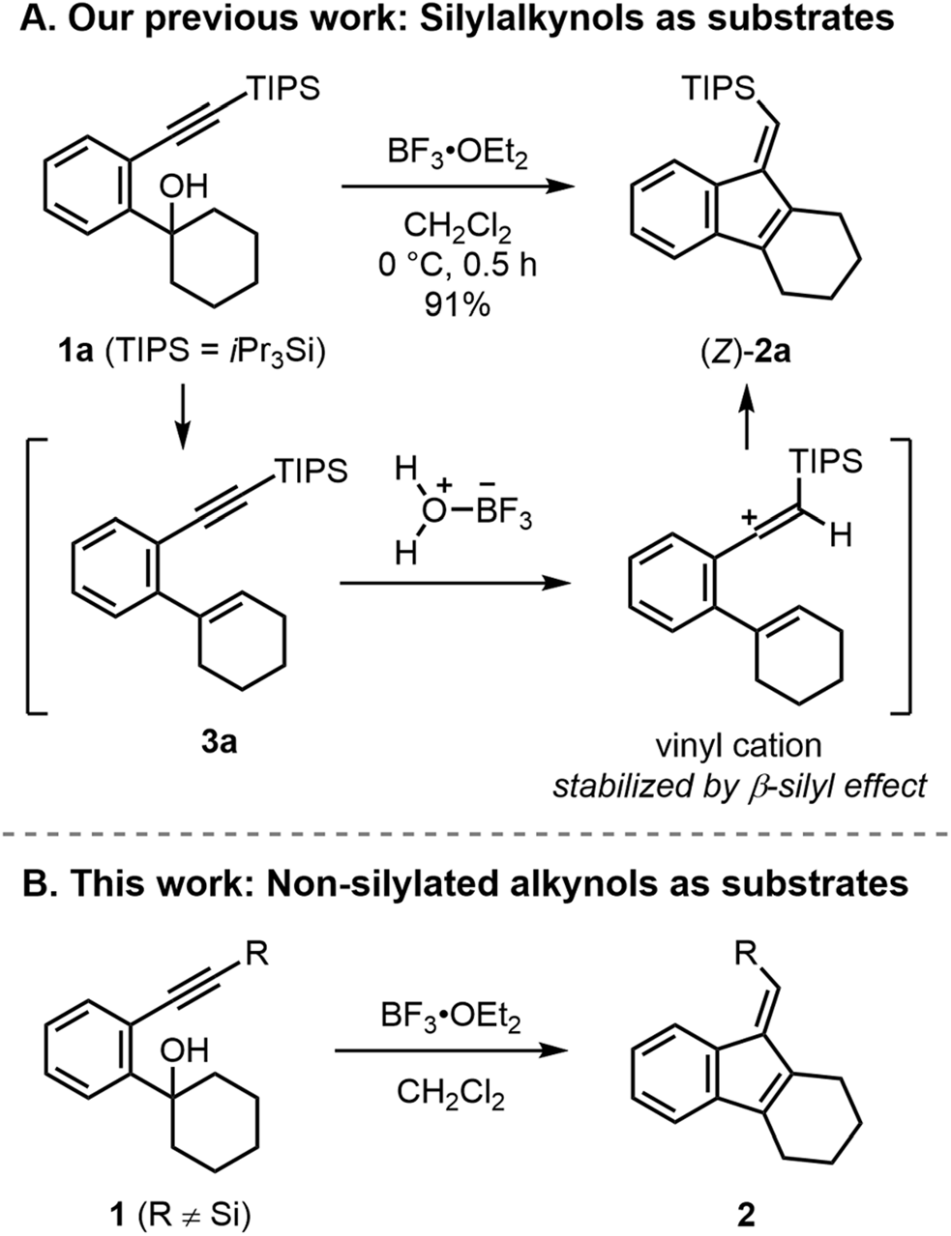
Domino dehydration/electrophilic cyclization of alkynols.

In our previous study,^10^ we demonstrated that BF_3_·OEt_2_ promotes a domino dehydration/electrophilic cyclization of silylalkynol 1a, affording the less accessible tricyclic benzofulvene 2a in high yield with complete *Z*-selectivity (Scheme 1A). Mechanistic studies suggested that BF_3_·OH_2_,^11,12^ generated *in situ* under the reaction conditions, acts as a Brønsted acid to activate an intermediate alkyne 3a, thereby promoting the subsequent electrophilic cyclization step. We also identified the silyl substituent on alkyne 1a as being particularly favorable for this transformation, with the triisopropylsilyl (TIPS) group proving optimal. The high reactivity of silyl-substituted alkynes is attributed to stabilization of vinyl cation intermediates through the β-silyl effect,^13^ which facilitates the protonation of alkynes. Although the β-silyl effect has been proposed to facilitate protonation, its role in controlling the overall reaction pathway and stereochemical outcome has not yet been clarified. Whether the β-silyl effect is essential for this transformation and how its removal influences the reaction mechanism and stereoselectivity remain unresolved. Addressing these questions would provide important mechanistic insights into Brønsted acid-mediated electrophilic cyclization of alkynes.

Herein, non-silylated alkynes were employed as mechanistic probes to clarify how the β-silyl effect governs the reaction pathway and stereochemical outcome in BF_3_-mediated domino dehydration/electrophilic cyclization. Combined experimental and computational studies reveal the mechanistic origin of the substituent-dependent stereoselectivity and the β-silyl effect in determining the reaction pathway (Scheme 1B).

## 2 Results and discussion

### 2.1 Optimization of the domino dehydration/electrophilic cyclization for non-silylated substrates

We initially examined the cyclization of substrate 1b bearing a *tert*-butyl (*t*Bu) substituent, which has a bulkiness similar to that of the silyl group (Table 1). Under the optimized conditions developed previously (1.1 equiv. of BF_3_·OEt_2_, 0 °C, 0.5 h), (*Z*)-2b (30%) and (*E*)-2b (12%) were obtained along with the dehydrated product 3b (57%) (entry 1). The stereochemistries of (*Z*)- and (*E*)-2b were determined by ^1^H NMR and NOE experiments (further details are provided in the SI). Prolonging the reaction time to 6 h resulted in the near-complete consumption of 3b and increased the yields of (*Z*)-2b (55%) and (*E*)-2b (20%) (entry 2), indicating that 3b serves as an intermediate. Comparison with the previously reported TIPS-substituted substrate (Scheme 1A) therefore indicates that replacement of the TIPS group with a *t*Bu group slows the cyclization step and allows formation of the *E*-isomer. Other boron Lewis acids, such as BCl_3_, B(C_6_F_5_)_3_, and B-chlorocatecholborane (ClBcat), exhibited significantly lower reactivities (entries 3–5). Conducting the reaction at room temperature accelerated cyclization and slightly improved the yield of (*Z*)-2b (61%) (entry 6). In contrast, using catalytic amounts of BF_3_·OEt_2_ resulted exclusively in dehydration, indicating that stoichiometric BF_3_·OEt_2_ is required for cyclization (entry 7). Therefore, the optimized reaction conditions were identified as in entry 6 (1.1 equiv. of BF_3_·OEt_2_ at room temperature).

**Table 1:** Optimization of reaction conditions^a^

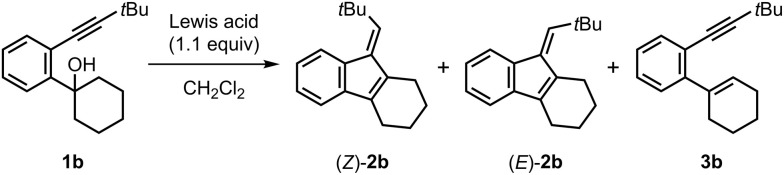						
Entry	Lewis acid	Temp.	Time	Yield^b^ (%)		
				(*Z*)-2b	(*E*)-2b	3b
1	BF_3_·OEt_2_	0 °C	0.5 h	30	12	57
2	BF_3_·OEt_2_	0 °C	6 h	55	20	4
3	BCl_3_	0 °C	6 h	3	1	2
4	B(C_6_F_5_)_3_	0 °C	6 h	10	0	15
5	ClBcat	0 °C	6 h	0	0	25
6	BF_3_·OEt_2_	rt	1 h	61	19	2
7^c^	BF_3_·OEt_2_	rt	1 h	0	0	80

a Standard conditions: 1b (1 equiv.), Lewis acid (1.1 equiv.), CH_2_Cl_2_ (0.1 M).

b Isolated yield.

c 0.1 equiv. of BF_3_·OEt_2_ was used.

### 2.2 Role of the β-silyl effect in reactivity and stereoselectivity

To clarify how removal of the β-silyl effect influences the reaction pathway and stereochemical outcome, a series of non-silylated alkynes was investigated (Table 2). Substrates 1b and 1c, bearing tertiary alkyl groups (*t*Bu and 1-adamantyl; 1-Ad), underwent domino dehydration/electrophilic cyclization within 1 h, affording 2b and 2c in good yield with predominant *Z*-selectivity (entries 1 and 2). In contrast, the triphenylmethyl (trityl; Tr) substituent (1d) completely suppressed the cyclization, leading only to dehydration (entry 3). For the secondary isopropyl (*i*Pr) substituent, although full consumption of both starting material 1e and dehydrated intermediate 3e required 4 h, cyclized product 2e was obtained in moderate yield (entry 4). Notably, the *E*-isomer of 2e was preferentially afforded, representing a reversal of stereoselectivity. A primary *n*Bu group (1f) also permitted this transformation to furnish 2f in moderate yield with *E*-selectivity (entry 5). These results demonstrate the strong dependence of both the reactivity and stereoselectivity on the steric and electronic properties of the substituent. In particular, the shift from *Z*-selectivity for tertiary alkyl substituents to *E*-selectivity for secondary and primary alkyl substituents suggests that removal of the β-silyl effect alters the relative accessibility of the competing stereochemical pathways.^14^

**Table 2:** Substituent effect on alkynes^a^

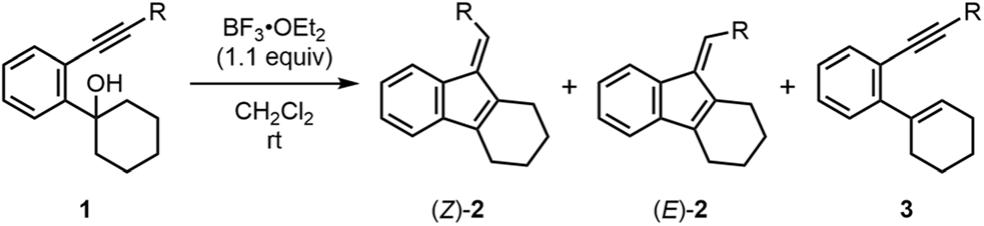					
Entry	Substrate 1	Time	Yield^b^ (%)		
			(*Z*)-2	(*E*)-2	3
1	1b (R = *t*Bu)	1 h	61	19	2
2	1c (*R* = 1-Ad)	1 h	52	10	0
3	1d (R = Tr)	6 h	0	0	62
4	1e (R = *i*Pr)	4 h	22	59	0
5	1f (R = *n*Bu)	3 h	15	40	Trace

a Standard conditions: 1 (1 equiv.), BF_3_·OEt_2_ (1.1 equiv.), CH_2_Cl_2_ (0.1 M), rt.

b Isolated yield.

### 2.3 Mechanistic studies

**Scheme 2:**
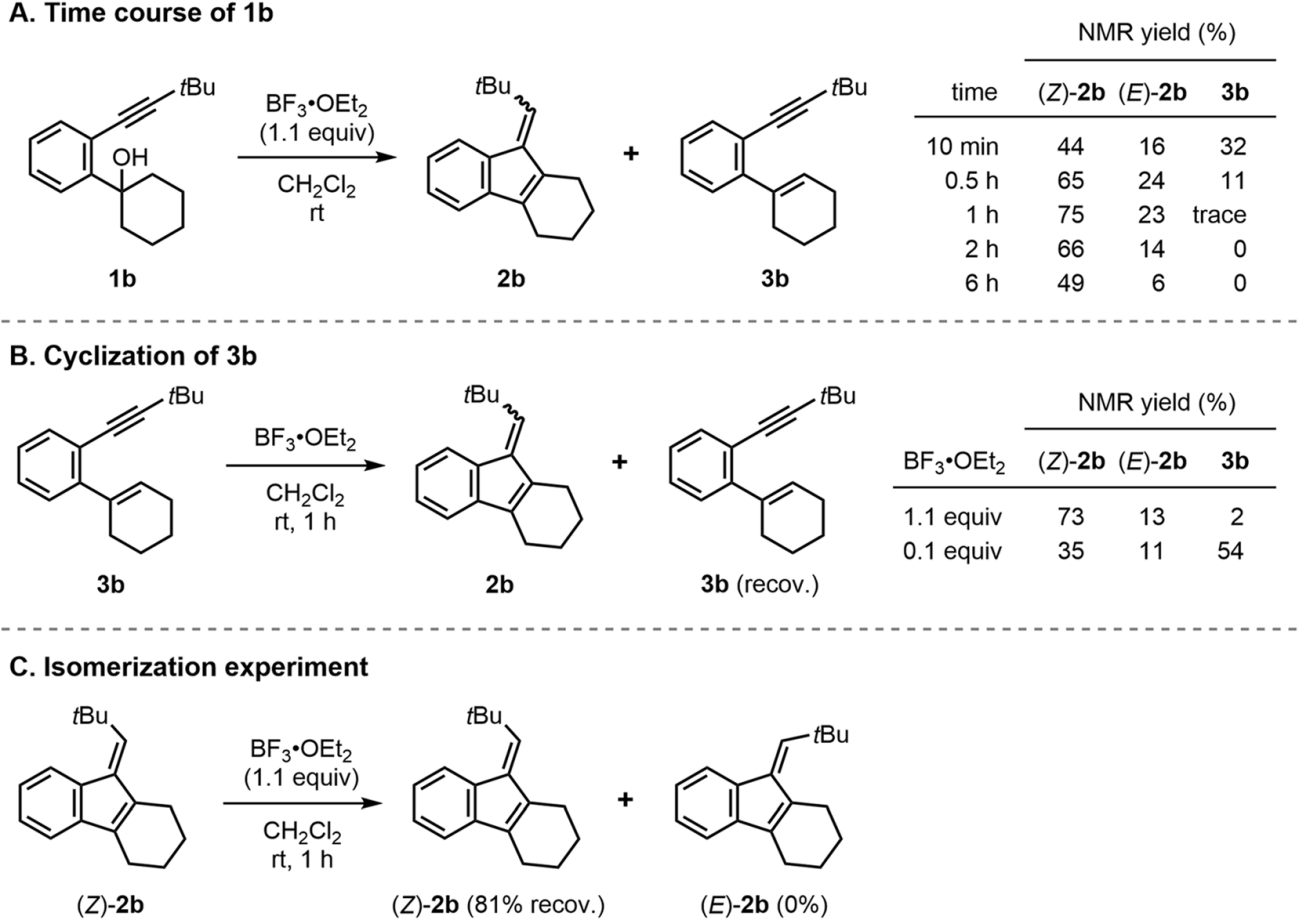
Control experiments.

Control experiments were conducted to gain insights into the reaction mechanism (Scheme 2). First, we monitored the time course of the transformation of 1b in detail (Scheme 2A). In the early stages of the reaction, the dehydrated product 3b was observed, followed by its gradual disappearance, concomitant with an increase in the yield of the cyclized product 2b, suggesting that 3b serves as a reaction intermediate. To confirm this inference, 3b was exposed to BF_3_·OEt_2_ (1.1 equiv.), which indeed produced 2b (Scheme 2B). Our previous study revealed that the treatment of 3a with BF_3_·OEt_2_ also led to cyclization. This strongly suggested that BF_3_·OH_2_, generated *in situ* between BF_3_·OEt_2_ and residual water, would function as an electrophile for the alkyne.^10^ Notably, even employing a catalytic amount of BF_3_·OEt_2_ afforded cyclized product 2b in a combined yield of 46%. We also examined whether (*Z*)-2b underwent isomerization under these reaction conditions; however, no conversion of (*Z*)-2b to (*E*)-2b was observed (Scheme 2C). This result indicated that the stereochemistry of 2b settled in the cyclization step.

**Scheme 3:**
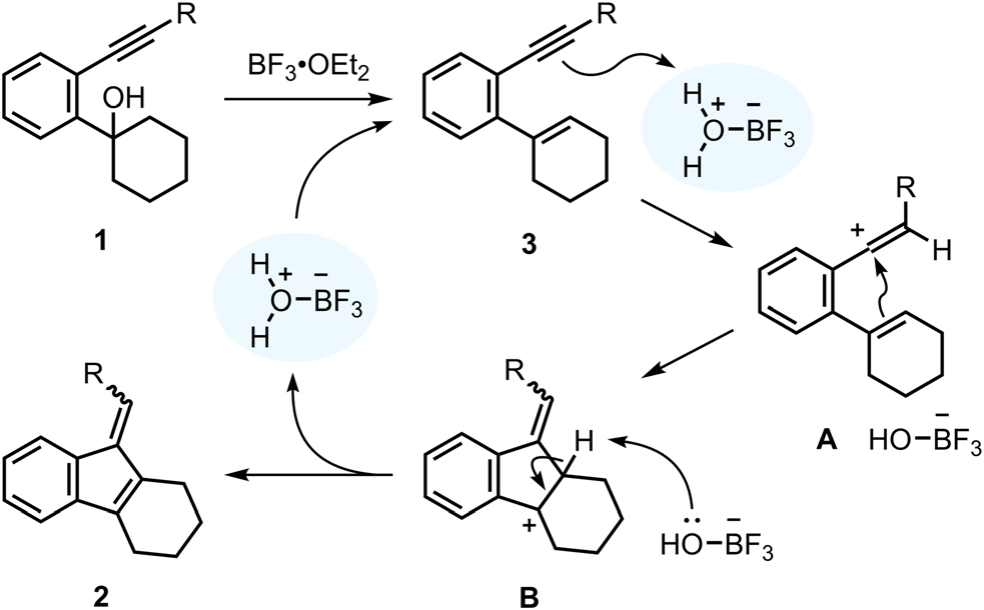
Plausible reaction mechanism.

Based on these results, a plausible reaction mechanism was proposed in Scheme 3. An initial dehydration of 1 produces alkene 3 along with BF_3_·OH_2_. Protonation of alkyne 3 by BF_3_·OH_2_ forms vinyl cation A, which undergoes intramolecular cyclization to give benzylic cation B, followed by deprotonation to furnish 2. In a silyl-substituted system, the β-silyl effect stabilizes vinyl cation A, thereby lowering the barrier for its formation. In contrast, alkyl substituents lack this stabilizing effect, rendering protonation of the alkyne slower and consequently retarding the overall cyclization process. To clarify how the β-silyl effect influences the energetics of the competing reaction pathways and the origin of the observed stereoselectivity, density functional theory (DFT) calculations were subsequently carried out.

### 2.4 DFT studies on the origin of stereoselectivity

#### 2.4.1 Mechanistic reference: TIPS-substituted system

To elucidate the origin of the observed *E*/*Z* selectivity, DFT calculations were performed for the key cyclization steps. In our previous study, noncovalent interactions (NCIs) were suggested to play a crucial role in determining stereoselectivity. Accordingly, all the calculations were performed at the TPSSh-D4(CPCM)/def2-TZVPPD//r^2^SCAN-3c(CPCM) level of theory. Geometry optimizations were performed using the r^2^SCAN-3c composite method, which combines the numerically efficient r^2^SCAN *meta*-GGA functional with three corrections (dispersion, basis-set superposition error, and basis-set incompleteness corrections). Extensive benchmark studies have demonstrated that this approach provides near triple-*ζ* DFT accuracy for molecular geometries and energetics at a significantly reduced computational cost.^15,16^ Single-point energies were subsequently evaluated at the TPSSh-D4/def2-TZVPPD level of theory to obtain more accurate electronic energies.

Before discussing the reaction mechanism, we evaluated the relative stability of the two acid species, BF_3_·OH_2_ and BF_3_·OEt_2_. BF_3_·OH_2_ was calculated to be only 3.8 kcal mol^−1^ less stable than BF_3_·OEt_2_, indicating that its *in situ* formation from BF_3_·OEt_2_ in the presence of residual water is thermodynamically feasible. Furthermore, direct activation of the alkyne by BF_3_ proceeds with substantially higher activation barriers than the corresponding BF_3_·OH_2_-mediated pathway (Fig. S1(a)). This computational result supports the mechanistic proposal in Section 2.3 that BF_3_·OH_2_ generated *in situ* functions as the Brønsted acid responsible for initiating electrophilic cyclization. Full energy profiles and NCI plots for 3a are provided in the SI. Briefly, BF_3_·OH_2_-mediated cyclization of 3a proceeds through π-coordination and protonation of the alkyne, vinyl cation formation, intramolecular electrophilic cyclization, and final deprotonation.

In the TIPS-substituted system, protonation occurs with relatively low activation barriers, consistent with the stabilization of the vinyl cation intermediate by the β-silyl effect. Subsequent electrophilic cyclization was the rate-determining step and preferentially afforded the *Z*-isomer, as reported in our previous study. Herein, the pathways leading to the *Z*- and *E*-isomers are referred to as the (*Z*)-and (*E*)-pathways, respectively. The *Z*-selectivity arises because the competing (*E*)-pathway suffers from steric congestion between the TIPS substituent and the cyclohexene moiety, whereas the (*Z*)-pathway is stabilized by an attractive H⋯F noncovalent interaction.

The key question in the present study is to understand how the removal of the β-silyl effect perturbs the relative energetics of protonation and cyclization, and consequently alters the stereochemical outcome. To address this question, *t*Bu- and *i*Pr-substituted systems were selected as representative non-silylated substrates because they exhibited opposite selectivity trends in the experimental results described above.

#### 2.4.2 *t*Bu-substituted system

**Fig. 1:**
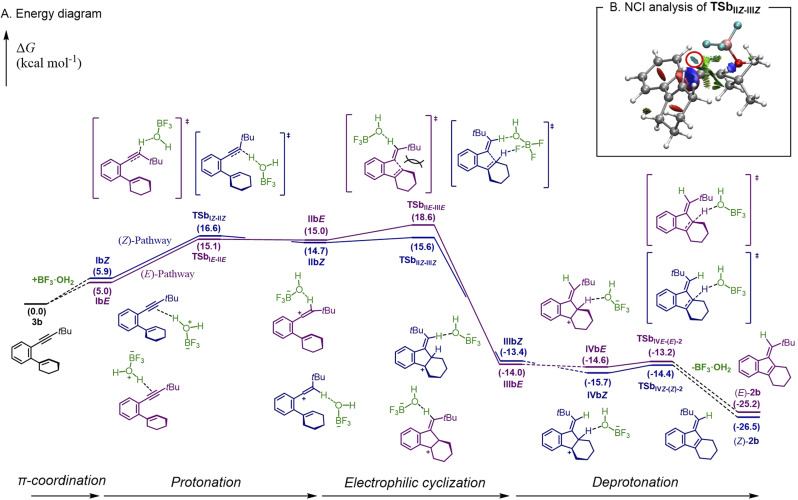
(A) Gibbs free energy diagram at the TPSSh-D4(CPCM)/def2-TZVPPD//r^2^SCAN-3c(CPCM) level in kcal mol^−1^ at 273 K for the cyclization of 3b (*t*Bu). (B) Three-dimensional NCI plot of TSb_II_*_Z_*_–III_*_Z_*. Green areas indicate weak noncovalent interactions, red regions depict strong nonbonded overlaps, and blue areas represent strong attractive interactions.

As the first representative non-silylated substrate, we examined the reaction pathway of the *t*Bu-substituted system 3b (Fig. 1A). In the (*Z*)-pathway, protonation is rate-determining (TSb_I_*_Z_*_–II_*_Z_*: 16.6 kcal mol^−1^), and the subsequent cyclization step proceeds through TSb_II_*_Z_*_–III_*_Z_* with a slightly lower barrier of 15.6 kcal mol^−1^. In contrast, in the (*E*)-pathway, protonation occurs with a lower activation barrier (TSb_I_*_E_*_–II_*_E_*: 15.1 kcal mol^−1^) than that in the (*Z*)-pathway. However, the cyclization step constituted the highest activation barrier (TSb_II_*_E_*_–III_*_E_*: 18.6 kcal mol^−1^).

Relative to the TIPS-substituted reference system 3a, the absence of β-silyl stabilization in the *t*Bu-substituted system 3b leads to the elevated barriers for protonation (TSb_I_*_E_*_–II_*_E_* and TSb_I_*_Z_*_–II_*_Z_*). Although protonation is slightly more favorable in the (*E*)-pathway, the significant steric congestion between the bulky *t*Bu group and the cyclohexene moiety destabilizes TSb_II_*_E_*_–III_*_E_*. In contrast, TSb_II_*_Z_*_–III_*_Z_* retains the attractive H⋯F interaction identified in the TIPS-substituted system, as supported by NCI analysis (Fig. 1B). As a result, the (*Z*)-pathway is kinetically favored, which is consistent with the experimentally observed *Z*-selectivity for the *t*Bu-substituted system. We also evaluated the BF_3_-mediated borylative cyclization pathway for 3b, which was found to be unfavorable under the present conditions (see the SI for details).

#### 2.4.3 *i*Pr-substituted system

**Fig. 2:**
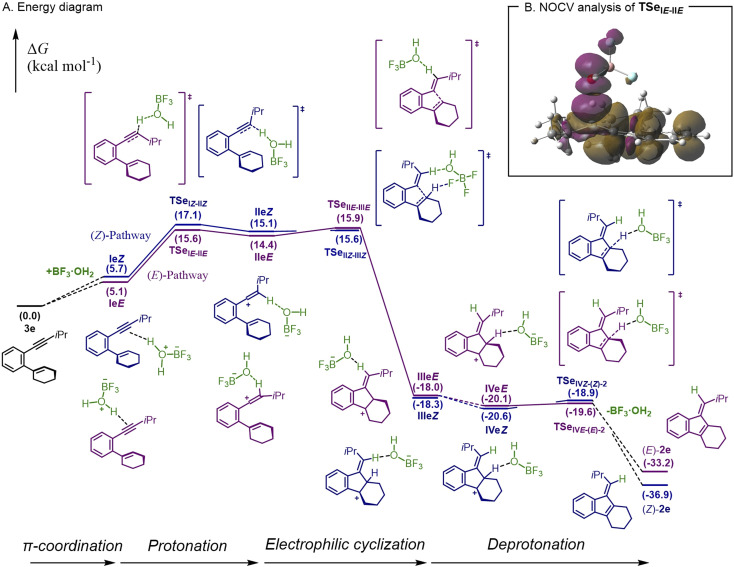
(A) Gibbs free energy diagram at the TPSSh-D4(CPCM)/def2-TZVPPD//r^2^SCAN-3c(CPCM) level in kcal mol^−1^ at 273 K: *i*Pr-substituted substrate 3e. (B) Deformation density obtained from NOCV analysis for TSe_I_*_E_*_–II_*_E_*. The density represents the dominant donor–acceptor interaction between the alkyne π orbital and the σ*(O–H) orbital during protonation. Brown and purple surfaces indicate electron donation and electron acceptance, respectively.

We next investigated the *i*Pr-substituted system to clarify why removal of the β-silyl effect leads to a reversal of stereoselectivity in less bulky alkyl-substituted systems. Fig. 2A shows the energy diagram of the *i*Pr-substituted system 3e. In the (*E*)-pathway, the cyclization barrier for 3e (TSe_II_*_E_*_–III_*_E_*: 15.9 kcal mol^−1^) is significantly lower than that for the *t*Bu-substituted system 3b (TSb_II_*_E_*_–III_*_E_*: 18.6 kcal mol^−1^, Fig. 1A), owing to reduced steric repulsion between the *i*Pr group and the cyclohexene moiety. As in the *t*Bu-substituted (Fig. 1A) system, protonation in the (*E*)-pathway is slightly more favorable than that in the competing (*Z*)-pathway (TSe_I_*_E_*_–II_*_E_*: 15.6 kcal mol^−1^*vs.*TSe_I_*_Z_*_–II_*_Z_*: 17.1 kcal mol^−1^). Consequently, the pathway leading to (*E*)-2e exhibits a lower overall activation barrier of 15.9 kcal mol^−1^ (TSe_II_*_E_*_–III_*_E_*) than the (*Z*)-pathway (TSe_I_*_Z_*_–II_*_Z_*: 17.1 kcal mol^−1^), rendering the (*E*)-pathway kinetically preferred, in agreement with the experimental results.

To gain further insights into the origin of the lower protonation barrier in the (*E*)-pathway, energy decomposition analysis (EDA) and natural orbitals for chemical valence (NOCV) analyses were performed for the protonation transition states corresponding to the vinyl cation formation step. The calculations suggested that TSe_I_*_E_*_–II_*_E_* is stabilized by enhanced orbital interactions arising from more effective donor–acceptor interaction between the alkyne π orbital and the σ*(O–H) orbital during protonation, as shown in Fig. 2B; further details are provided in the SI.

## 3 Conclusions

This study elucidated the mechanistic origin of substituent-dependent stereoselectivity in the BF_3_-mediated domino dehydration/electrophilic cyclization of alkynols. The latter electrophilic cyclization would be promoted by BF_3_·OH_2_, generated *in situ*. The stereochemical outcome is governed by substituent-dependent variations in the rate-determining step, as demonstrated by DFT calculations. These findings establish a mechanistic framework for understanding substituent effects in Brønsted acid-mediated electrophilic cyclization of alkynes.

## 4 Computational methods

Geometry optimizations of all stationary points were performed using the r^2^SCAN-3c method,^15,17^ a composite density functional method designed for efficient and reliable geometry optimization within the conductor-like polarizable continuum model (CPCM) for dichloromethane.^18^ Vibrational frequency calculations were carried out at the same level of theory to classify all structures as either minima or transition states. Subsequent single-point energy refinements were performed using the TPSSh functional^19,20^ with a D4 dispersion correction^21^ and the def2-TZVPPD basis set,^22^ which also employed the CPCM solvation model for dichloromethane. Reported Gibbs free energies include thermal corrections obtained from the frequency calculations at 273 K. EDA calculations^23–25^ were performed by partitioning the system into BF_3_·OH_2_ and dehydrated substrate 3 fragments, both treated as neutral singlet species (charge = 0, multiplicity = 1). Three-dimensional NCI plots were generated using NCIPLOT^26,27^ and visualized with VMD^28^ to analyze the noncovalent interactions in the transition states of the cyclization steps. All calculations were performed using the ORCA 6.1 program package.^29^

## Author contributions

Takashi Okitsu: conceptualization, methodology, investigation (experimental studies: lead), data curation, writing the original draft (lead), writing the review, and editing. Takeshi Yoshikawa: methodology, investigation (computational studies: lead), data curation, formal analysis (lead), writing the original draft (supporting), writing the review, and editing. Yuka Takayori: investigation (experimental studies: supporting), validation. Syuto Hirata: formal analysis (supporting), investigation (computational studies: supporting) and validation. Masanari Shinoki: formal analysis (supporting), investigation (computational studies: supporting), and validation. Momoka Ito: formal analysis (supporting), investigation (computational studies: supporting) and validation. Ken Sakata: resources, supervision (computational studies), writing, review, and editing. Manabu Hatano: resources, supervision (experimental studies), writing, reviewing, and editing.

## Conflicts of interest

There are no conflicts to declare.

## Supplementary Material

RA-OLF-D6RA05267C-s001

## Data Availability

Data supporting this article are included in the supplementary information (SI). Supplementary information: includes the experimental procedures, spectroscopic data, computational details, Cartesian coordinates, and the energies of all calculated structures. See DOI: https://doi.org/10.1039/d6ra05267c.

## References

[cit1] Togo H., Iida S. (2006). Synlett.

[cit2] Yamamoto Y., Gridnev I. D., Patil N. T., Jin T. (2009). Chem. Commun..

[cit3] Mphahlele M. J. (2009). Molecules.

[cit4] Godoi B., Schumacher R. F., Zeni G. (2011). Chem. Rev..

[cit5] Palisse A., Kirsch S. F. (2012). Org. Biomol. Chem..

[cit6] Aggarwal T., Kumar S., Verma A. K. (2016). Org. Biomol. Chem..

[cit7] Praveen C., Szafert S. (2023). ChemPlusChem.

[cit8] Dorel R., Echavarren A. M. (2015). Chem. Rev..

[cit9] Hermange P., Gicquiaud J., Barbier M., Karnat A., Toullec P. Y. (2022). Synthesis.

[cit10] Okitsu T., Yoshikawa T., Morohashi M., Aoki K., Yakura T., Sakata K., Hatano M. (2024). Org. Lett..

[cit11] Huang M., Hu L., Shen H., Liu Q., Hussain M. I., Pan J., Xiong Y. (2016). Green Chem..

[cit12] Prakash G. K. S., Gurung L., Marinez E. R., Mathew T., Olah G. A. (2016). Tetrahedron Lett..

[cit13] Siehl H.-U., Kaufmann F.-P. (1992). J. Am. Chem. Soc..

[cit14] An exception to this trend was observed when cycloheptanol 1g was used in place of cyclohexanol 1b. Both (*Z*)-2g and double bond-isomerized product 2g′ were formed, whereas the corresponding (*E*)-2g was not observed. Further studies aimed at elucidating the origin of this unexpected result are currently in progress and will be reported in the near future. 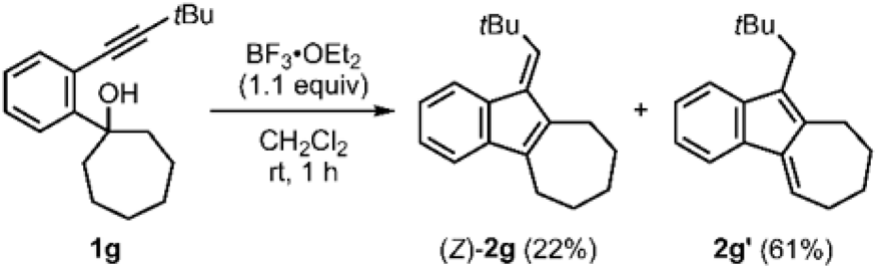

[cit15] Grimme S., Hansen A., Brandenburg J. G., Bannwarth C. (2021). J. Chem. Phys..

[cit16] Grimme S., Brandenburg J. G., Bannwarth C., Hansen A. (2015). J. Chem. Phys..

[cit17] Furness J. W., Kaplan A. D., Ning J., Perdew J. P., Sun J. (2020). J. Phys. Chem. Lett..

[cit18] Klamt A., Schüürmann G. (1993). J. Chem. Soc., Perkin Trans. 2.

[cit19] Staroverov V. N., Scuseria G. E., Tao J., Perdew J. P. (2003). J. Chem. Phys..

[cit20] Tao J., Perdew J. P., Staroverov V. N., Scuseria G. E. (2003). Phys. Rev. Lett..

[cit21] Caldeweyher E., Ehlert S., Hansen A., Neugebauer H., Spicher S., Bannwarth C., Grimme S. (2019). J. Chem. Phys..

[cit22] Weigend F., Ahlrichs R. (2005). Phys. Chem. Chem. Phys..

[cit23] Su P., Li H. (2009). J. Chem. Phys..

[cit24] Mitoraj M. P., Michalak A. (2007). J. Mol. Model..

[cit25] Mitoraj M. P., Michalak A., Ziegler T. (2009). J. Chem. Theory Comput..

[cit26] Contreras-García J., Johnson E. R., Keinan S., Chaudret R., Piquemal J.-P., Beratan D. N., Yang W. (2011). J. Chem. Theory Comput..

[cit27] Johnson E. R., Keinan S., Mori-Sánchez P., Contreras-García J., Cohen A. J., Yang W. (2010). J. Am. Chem. Soc..

[cit28] Humphrey W., Dalke A., Schulten K. (1996). J. Mol. Graphics.

[cit29] Neese F. (2025). Wiley Interdiscip. Rev.: Comput. Mol. Sci..

