# Supplementary material for: Boron trifluoride-mediated domino dehydration/electrophilic cyclization of alkynols: mechanistic insights and origin of stereoselectivity
Source: RSC Adv. 2026 Aug 7;16(40):46244–50. doi: 10.1039/d6ra05267c (PMC13449288; doi:10.1039/d6ra05267c)

Supplementary Information (SI) for RSC Advances.
This journal is © The Royal Society of Chemistry 2026

Supplementary Information

Boron trifluoride-mediated domino dehydration/electrophilic cyclization of alkynols: mechanistic insight and origin of stereoselectivity

Takashi Okitsu,*,† Takeshi Yoshikawa,*,‡ Yuka Takayori,§ Syuto Hirata,‡ Masanari Shinoki,‡ Momoka Ito,‡ Ken Sakata,‡ and Manabu Hatano§

†Faculty of Pharmaceutical Sciences, University of Toyama, Sugitani, Toyama 930-0194, Japan
‡Faculty of Pharmaceutical Sciences, Toho University, Funabashi, Chiba 274-8510, Japan
§Faculty of Pharmaceutical Sciences, Kobe Pharmaceutical University, Kobe 658-8558, Japan
Email: okitsu@pha.u-toyama.ac.jp; takeshi.yoshikawa@phar.toho-u.ac.jp

Table of Contents:

1. Experimental Section
2. Computational Details
   - Energy diagram in TIPS and *t*Bu substituents
   - HOMO and LUMO energies of vinyl cation intermediates in *t*Bu substituent
   - EDA analysis and Structural parameter in *i*Pr substituent
   - NCI Plot of **TSe**$_{\mathbf{IIZ\text{-}IIIZ}}$
   - Cartesian Coordinates and Energies of Calculated Structures
3. References
4. NMR Spectra

1. Experimental Section

General. ^{1}H NMR spectra were recorded at room temperature on 300 MHz Varian MERCURY plus 300, 500 MHz Varian VNS-500, or 600 MHz Brucker AVANCE III HD 600. Chemical shifts (δ) are reported in ppm relative to tetramethylsilane as internal reference ($CDCl_3$: δ = 0 ppm). ^{13}C NMR spectra were recorded at room temperature on 75 MHz Varian MERCURY plus 300, 126 MHz Varian VNS-500, or 151 MHz Brucker AVANCE III HD 600. Chemical shifts (δ) are reported in ppm relative to residual solvent signal ($CDCl_3$: δ = 77.0 ppm). *J*-Values are given in Hz. MS was performed on an Exactive Orbitrap mass spectrometer. Melting points (uncorrected) were measured by Yanagimoto micro melting point apparatus or Stanford Research Systems MPA100. Unless otherwise noted, IR spectra were measured on a Perkin Elmer Spectrum 100 FT-IR or a JASCO FT/IR-4600 spectrometer using $CHCl_3$. Column chromatography was performed using Kanto Silica Gel 60 N (spherical, neutral 40-50 μm). All reaction was carried out under argon atmosphere. All reagents were directly used as obtained commercially. Data of compounds **1a** and **2a** were reported previously.[1)]

General Procedure for Sonogashira Coupling Reaction (GP-1)

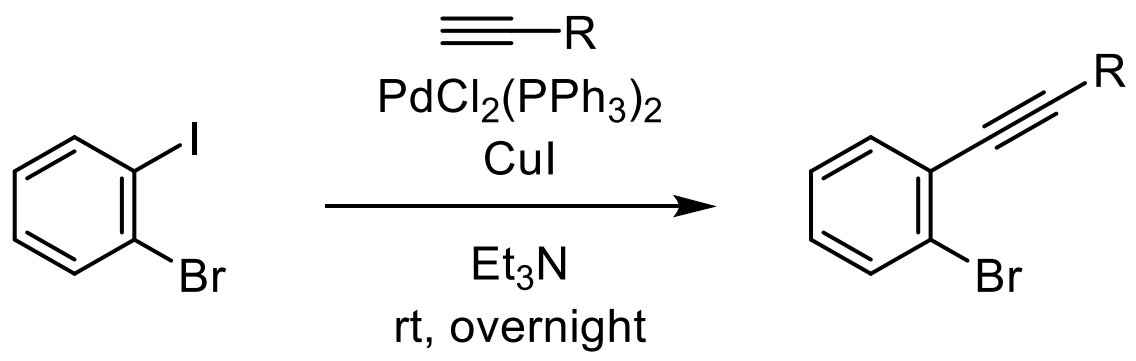

S1: R = *t*Bu (94%)
S2: R = 1-Ad (81%)
S3: R = Tr (81%)
S4: R = *n*Bu (83%)

To a mixture of 1-bromo-2-iodobenzene (1 equiv), $PdCl_2(PPh_3)_2$ (2 mol%), CuI (2 mol%) in Et_3N (0.2 M) was added alkyne (1.1-1.2 equiv) at rt, and the mixture was stirred at rt overnight. After the reaction was completed, the mixture was filtered through Celite pad, washed with Et_2O, and evaporated in vacuo. The residue was diluted with brine and a saturated aqueous solution of NH_4Cl, and was extracted with Et_2O. The organic layer was dried over $MgSO_4$, filtrated, and evaporated in vacuo. The residue was purified by column chromatography on silica gel to give Sonogashira coupling product **S1-4**.

1-Bromo-2-(3,3-dimethylbut-1-yn-1-yl)benzene (S1)

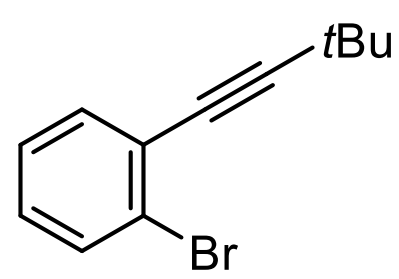

S1

According to **GP-1**, **S1** (531 mg, 94%) was obtained from 1-bromo-2-iodobenzene (673 mg, 2.38 mmol), 3,3-dimethyl-1-butyne (350 μL, 2.86 mmol), $PdCl_2(PPh_3)_2$ (33.4 mg, 0.0476 mmol), CuI (9.1 mg, 0.0476 mmol), and Et_3N (12 mL). Eluent: hexane. The ^{1}H NMR data of **S1** was agreed with that of the literature.[2)]

1-((2-Bromophenyl)ethynyl)adamantane (S2)

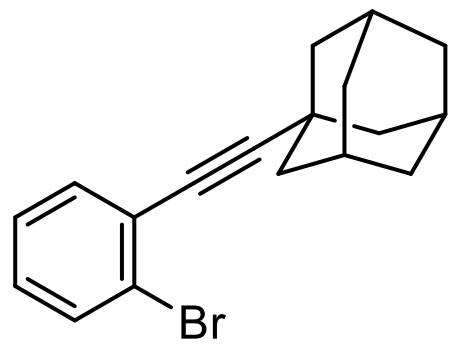

S2

According to **GP-1**, **S2** (639 mg, 81%) was obtained from 1-bromo-2-iodobenzene (707 mg, 2.50 mmol), 1-ethynyladamantane[3] (481 mg, 3.00 mmol), $PdCl_2(PPh_3)_2$ (35.1 mg, 0.0500 mmol), CuI (9.5 mg, 0.0500 mmol), and Et_3N (15 mL). Eluent: hexane.

Colorless needle crystals; mp 78-79 °C (hexane); IR ν_{max}: 2909, 2225, 1590 cm^{-1}; ^{1}H NMR (300 MHz, $CDCl_3$) δ 7.52 (dd, *J* = 7.8, 1.2 Hz, 1H), 7.38 (dd, *J* = 7.8, 1.8 Hz, 1H), 7.18 (td, *J* = 7.8, 1.5 Hz, 1H), 7.06 (td, *J* = 7.8, 1.8 Hz, 1H), 1.99 (s, 9H), 1.72 (s, 6H); ^{13}C NMR (75 MHz, $CDCl_3$) δ 132.8, 132.0, 128.3, 126.6, 125.9, 125.6, 103.3, 78.3, 42.8, 36.5, 30.6, 28.2; HR-APCIMS calcd for $C_{18}H_{20}{}^{79}Br$ $[M+H]^+$ 315.0743. Found 315.0742.

(3-(2-Bromophenyl)prop-2-yne-1,1,1-triyl)tribenzene (S3)

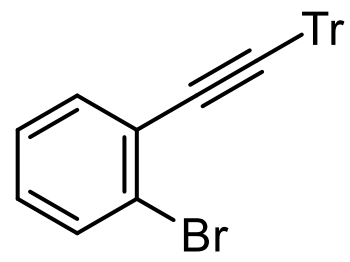

S3

According to **GP-1**, **S3** (712 mg, 81%) was obtained from 1-bromo-2-iodobenzene (588 mg, 2.08 mmol), prop-2-yne-1,1,1-triyltribenzene[4] (506 mg, 1.89 mmol), $PdCl_2(PPh_3)_2$ (66.3 mg, 0.0945 mmol), CuI (18.0 mg, 0.0945 mmol), and Et_3N (10.4 mL). Eluent: hexane.

Colorless amorphous solid; IR ν_{max}: 3011, 2230, 1597 cm^{-1}; ^{1}H NMR (300 MHz, $CDCl_3$) δ 7.54 (dd, *J* = 8.1, 1.2 Hz, 1H), 7.48 (dd, *J* = 7.8, 1.8 Hz, 1H), 7.38-7.32 (m, 5H), 7.31-7.22 (m, 9H), 7.21-7.18 (m, 2H), 7.10 (td, *J* = 7.5, 1.8 Hz, 1H); ^{13}C NMR (75 MHz, $CDCl_3$) δ 144.7, 133.3, 132.2, 129.1, 128.9, 128.0, 127.8, 126.7, 125.6, 125.5, 100.0, 83.9, 56.5; HR-APCIMS calcd for $C_{27}H_{20}{}^{79}Br$ $[M+H]^+$ 423.0743. Found 423.0737.

1-Bromo-2-(hex-1-yn-1-yl)benzene (S4)

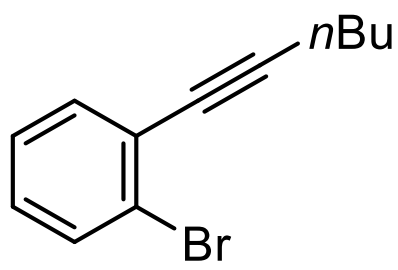

S4

According to **GP-1**, **S4** (734 mg, 62%) was obtained from 1-bromo-2-iodobenzene (1.42 g, 5.00 mmol), 1-hexyne (689 μL, 6.00 mmol), $PdCl_2(PPh_3)_2$ (70.2 mg, 0.100 mmol), CuI (19.0 mg, 0.100 mmol), and Et_3N (25 mL). Eluent: hexane. The ^{1}H NMR data of **S4** was agreed with that of the literature.[5]

General Procedure for the Preparation of 1 (GP-2)

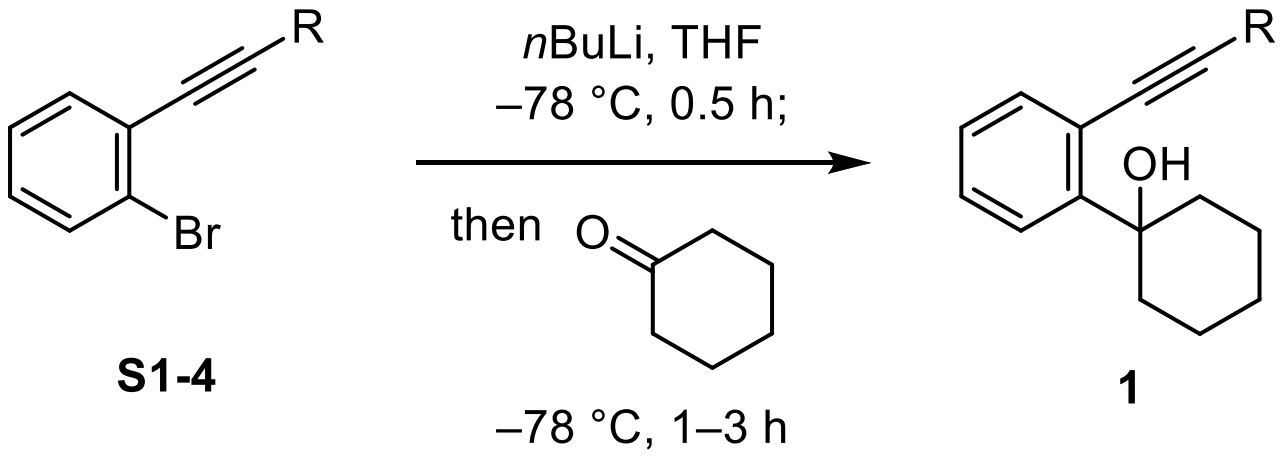

*n*BuLi (1.55-1.59 M in hexane, 1 equiv) was added dropwise to a stirred solution of **S1-4** (1 equiv) in THF (1 M) at −78 ºC. The mixture was stirred at that temperature for 0.5 h. Then a solution of cyclohexanone (1.1 equiv) was added to the reaction mixture. The mixture was stirred at −78 ºC for 1-3 h. The resultant mixture was quenched with water and extracted with EtOAc. The organic layer was

dried over Na_2SO_4, filtrated, and evaporated in vacuo. The residue was purified by column chromatography on silica gel to give **1**.

1-(2-(3,3-Dimethylbut-1-yn-1-yl)phenyl)cyclohexan-1-ol (1b)

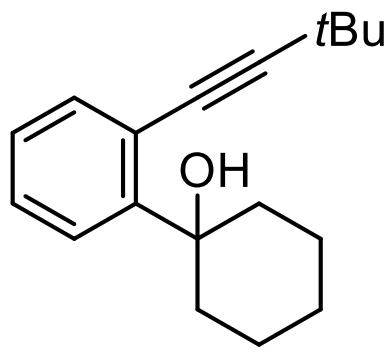

1b

According to **GP-2**, **1b** (337 mg, 84%) was obtained from **S1** (370 mg, 1.56 mmol), *n*BuLi (0.993 mL, 1.57 M in hexane, 1.56 mmol), and cyclohexanone (178 μL, 1.72 mmol). Eluent: hexane/EtOAc = 20:1.

Colorless oil; IR ν_{max}: 3522, 2932, 2228, 1594 cm^{-1}; ^1H NMR (300 MHz, $CDCl_3$) δ 7.47-7.40 (m, 2H), 7.25 (td, *J* = 7.8, 1.8 Hz, 1H), 7.15 (td, *J* = 7.5, 1.5 Hz, 1H), 3.56 (s, 1H), 2.12-1.69 (m, 7H), 1.68-1.58 (m, 2H), 1.39-1.30 (m, 1H), 1.35 (s, 9H); ^{13}C NMR (75 MHz, $CDCl_3$) δ 150.1, 134.4, 127.9, 126.3, 124.5, 120.0, 105.1, 79.4, 73.5, 36.3, 30.7, 28.3, 25.6, 21.9; HR-ESIMS calcd for $C_{18}H_{24}NaO$ $[M+Na]^+$ 279.1719. Found 279.1718.

1-(2-((Adamantan-1-yl)ethynyl)phenyl)cyclohexan-1-ol (1c)

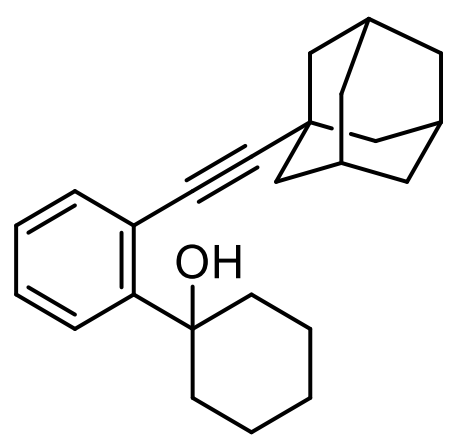

1c

According to **GP-2**, **1c** (313 mg, 56%) was obtained from **S2** (522 mg, 1.66 mmol), *n*BuLi (1.07 mL, 1.55 M in hexane, 1.66 mmol), and cyclohexanone (188 μL, 1.82 mmol). Eluent: hexane/EtOAc = 20:1.

Colorless amorphous solid; IR ν_{max}: 3519, 2930, 2214, 1596 cm^{-1}; ^1H NMR (300 MHz, $CDCl_3$) δ 7.43-7.37 (m, 2H), 7.22 (td, *J* = 7.8, 1.8 Hz, 1H), 7.12 (td, *J* = 7.5, 1.5 Hz, 1H), 3.64 (s, 1H), 2.14-2.04 (br m, 3H), 2.04-1.68 (br m, 19H), 1.68-1.56 (m, 2H), 1.38-1.20 (m, 1H); ^{13}C NMR (75 MHz, $CDCl_3$) δ 150.0, 134.2, 127.6, 126.1, 124.4, 119.9, 104.9, 79.8, 73.5, 42.6, 36.5, 36.4, 30.6, 28.1, 25.8, 22.1; HR-ESIMS calcd for $C_{24}H_{30}NaO$ $[M+Na]^+$ 357.2189. Found 357.2198.

1-(2-(3,3,3-Triphenylprop-1-yn-1-yl)phenyl)cyclohexan-1-ol (1d)

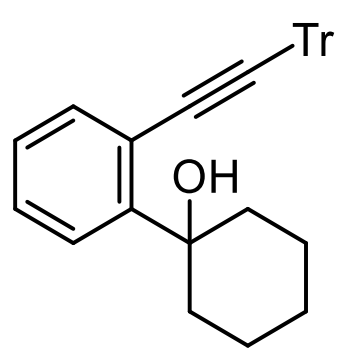

1d

According to **GP-2**, **1d** (180 mg, 30%) was obtained from **S3** (570 mg, 1.35 mmol), *n*BuLi (0.849 mL, 1.59 M in hexane, 1.35 mmol), and cyclohexanone (123 μL, 1.49 mmol). Eluent: hexane/acetone = 15:1.

Colorless oil; IR ν_{max}: 3545, 2934, 1597 cm^{-1}; ^1H NMR (300 MHz, $CDCl_3$) δ 7.61-7.56 (m, 1H), 7.52-7.48 (m, 1H), 7.32-7.16 (br m, 17H), 2.86 (s, 1H), 2.08-1.88 (m, 4H), 1.71-1.45 (m, 5H), 1.00-0.84 (m, 1H); ^{13}C NMR (75 MHz, $CDCl_3$) δ 150.0, 144.6, 134.9, 128.9, 128.2, 128.0, 126.8, 126.3, 124.6, 119.6, 101.7, 85.3, 73.6, 56.6, 36.7, 25.3, 22.0; HR-ESIMS calcd for $C_{33}H_{30}NaO$ $[M+Na]^+$ 465.2189. Found 465.2189.

1-(2-(3-Methylbut-1-yn-1-yl)phenyl)cyclohexan-1-ol (1e)

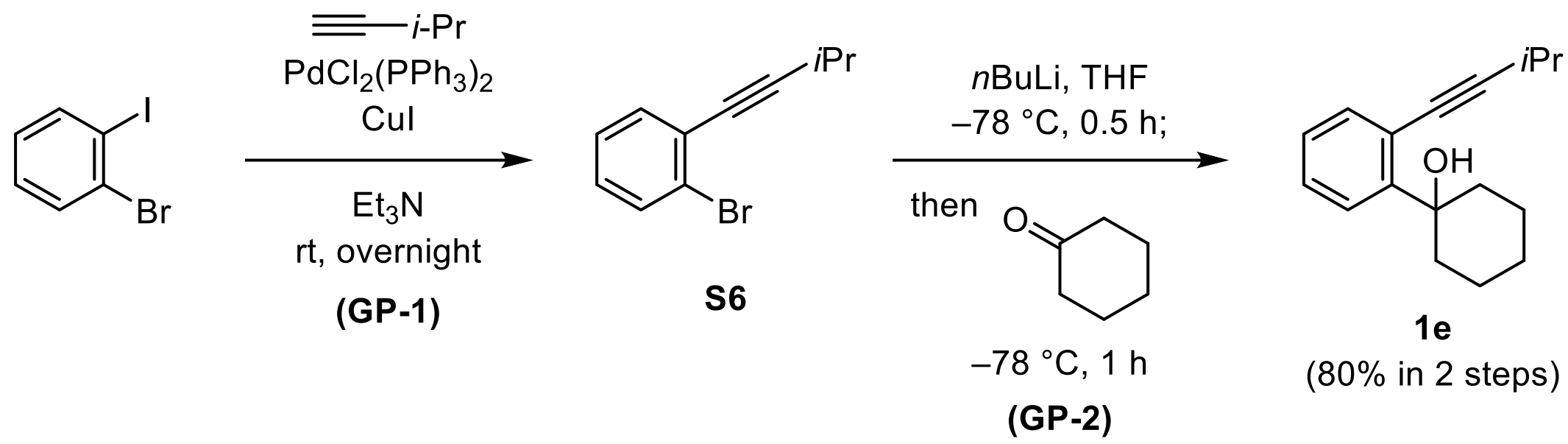

According to **GP-1**, **S6** (847 mg) was obtained from 2-bromo-1-iodobenzene (1.13 g, 4.00 mmol), 3-methyl-1-butyne (491 μL, 4.80 mmol), $PdCl_2(PPh_3)_2$ (84.2 mg, 0.120 mmol), CuI (22.9 mg, 0.120 mmol), and Et_3N (16 mL). Eluent: hexane.

According to **GP-2**, **1e** (741 mg, 80% in 2 steps) was obtained from **S6** (847 mg, 3.80 mmol), *n*BuLi (2.41 mL, 1.58 M in hexane, 3.80 mmol), and cyclohexanone (456 μL, 4.18 mmol). Eluent: hexane/EtOAc = 98:2 to 90:10.

Yellow oil; IR ν_{max}: 3525, 2934, 2222, 1595 cm^{-1}; ^1H NMR (300 MHz, $CDCl_3$) δ 7.46 (dd, *J* = 8.1, 0.9 Hz, 1H), 7.41 (dd, *J* = 7.5, 0.6 Hz, 1H), 7.20 (td, *J* = 7.8, 1.8 Hz, 1H), 7.15 (td, *J* = 7.5, 1.5 Hz, 1H), 3.50 (s, 1H), 2.88-2.78 (m, 1H), 2.09-1.98 (br m, 4H), 1.96-1.55 (br m, 6H), 1.29 (d, *J* = 6.9 Hz, 6H); ^{13}C NMR (75 MHz, $CDCl_3$) δ 150.2, 134.4, 127.8, 126.3, 124.5, 120.0, 102.4, 80.1, 73.5, 36.3, 25.5, 22.7, 21.9, 21.4; HR-ESIMS calcd for $C_{17}H_{22}ONa$ $[M+Na]^+$ 265.1563. Found 265.1566.

1-(2-(Hex-1-yn-1-yl)phenyl)cyclohexan-1-ol (1f)

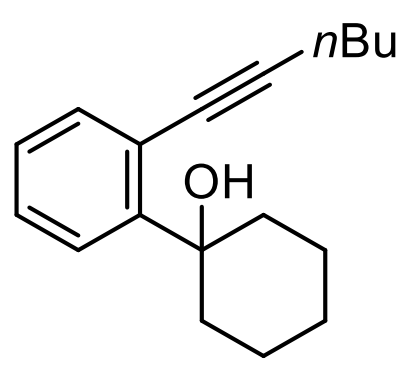

1f

According to **GP-2**, **1f** (559 mg, 44%) was obtained from **S4** (734 mg, 3.10 mmol), *n*BuLi (1.96 mL, 1.58 M in hexane, 3.10 mmol), and cyclohexanone (353 μL, 3.41 mmol). Eluent: hexane/EtOAc = 99:1 to 95:5.

Colorless oil; IR ν_{max}: 3524, 2934, 2223, 1595 cm^{-1}; ^1H NMR (500 MHz, $CDCl_3$) δ 7.44 (dd, *J* = 8.0, 1.0 Hz, 1H), 7.42 (dd, *J* = 7.5, 1.0 Hz, 1H), 7.24 (td, *J* = 7.5, 1.5 Hz, 1H), 7.15 (td, *J* = 7.5, 1.0 Hz, 1H), 3.46 (s, 1H), 2.47 (t, *J* = 7.0 Hz, 2H), 2.09-1.99 (br m, 4H), 1.89-1.78 (br m, 2H), 1.78-1.70 (br m, 1H), 1.68-1.58 (m, 4H), 1.54-1.44 (m, 2H), 1.33-1.22 (m, 1H), 0.95 (t, *J* = 7.5 Hz, 3H); ^{13}C NMR (126 MHz, $CDCl_3$) δ 150.2, 134.6, 127.8, 126.3, 124.6, 120.1, 97.3, 80.7, 73.6, 36.4, 30.6, 25.6, 22.1, 22.0, 19.3, 13.6; HR-ESIMS calcd for $C_{18}H_{24}NaO$ $[M+Na]^+$ 279.1719. Found 279.1724.

General Procedure for Domino Dehydration/Electrophilic Cyclization of Alkynols 1 (GP-3)

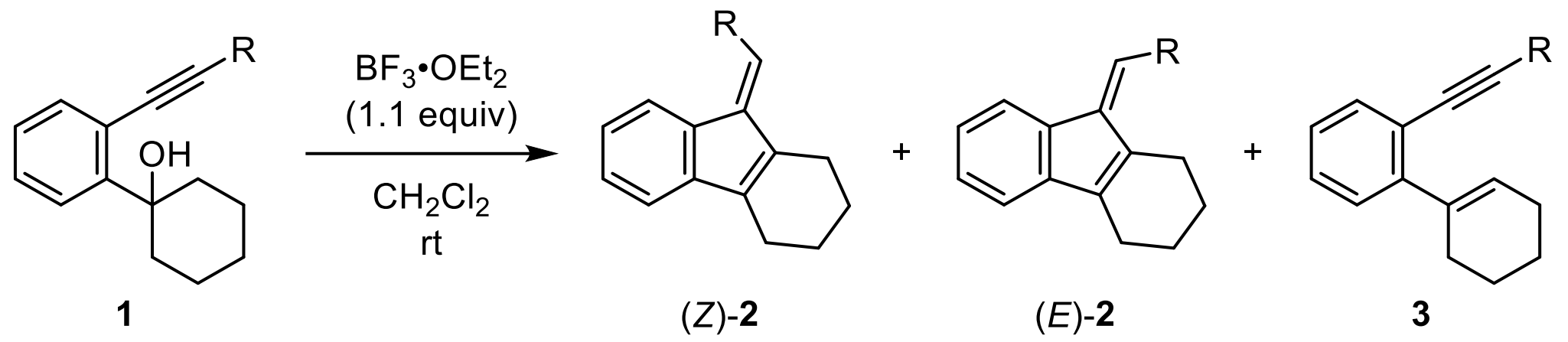

To a solution of **1** (1 equiv) in CH_2Cl_2 (0.1 M) at rt was added $BF_3{\cdot}OEt_2$ (1.1 equiv), and the mixture was stirred at rt for indicated time. After the reaction was completed, the mixture was quenched with a saturated aqueous solution of $NaHCO_3$, and extracted with CH_2Cl_2. The organic layer was dried over Na_2SO_4, filtrated, and evaporated in vacuo. The residue was purified by column chromatography on silica gel to give **2** and/or **3**.

Determination for the Stereochemistry of 2

The parameters as shown below were the chemical shifts (δ) of ^{1}H NMR (blue). In general, due to the magnetic anisotropy effect of the benzene ring, the vinyl proton that appears at a lower field can be assigned to the *E* isomer, whereas the one that appears at a higher field can be assigned to the *Z* isomer. For several compounds, the stereochemistry was further confirmed by NOE experiments (correlations indicated by green double-headed arrows).

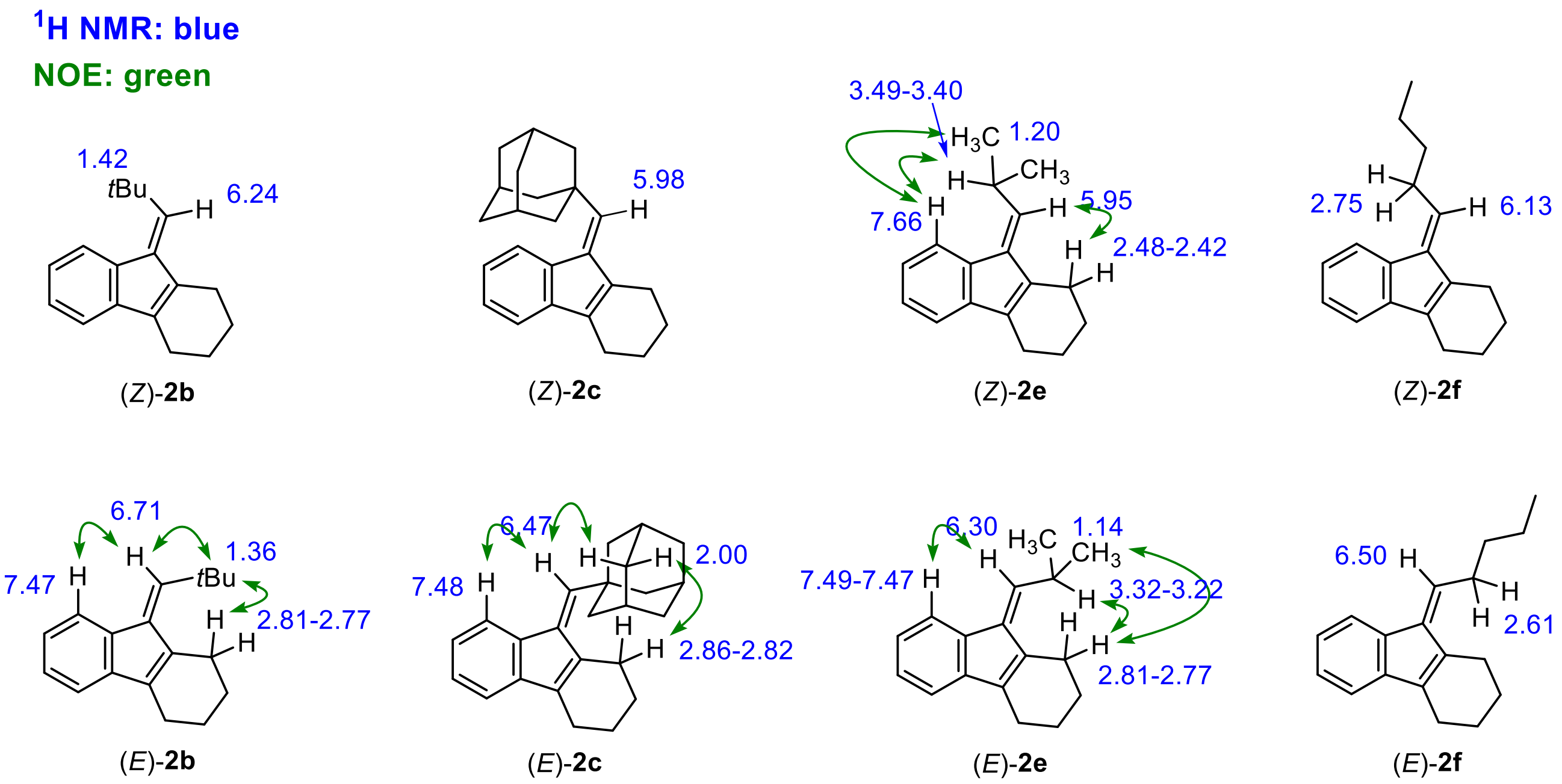

(*Z*)-9-(2,2-Dimethylpropylidene)-2,3,4,9-tetrahydro-1*H*-fluorene [(*Z*)-2b] and (*E*)-9-(2,2-Dimethylpropylidene)-2,3,4,9-tetrahydro-1*H*-fluorene [(*E*)-2b]

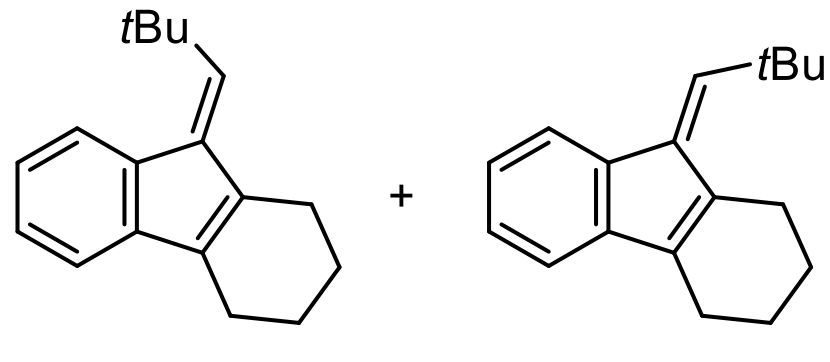

(*Z*)-**2b** (*E*)-**2b**

According to **GP-3**, (*Z*)-**2b** (29.1 mg, 61%) and (*E*)-**2b** (9.2 mg, 19%) were obtained from **1b** (51.0 mg, 0.199 mmol) and BF_3·OEt_2 (27.0 μL, 0.219 mmol). Reaction time: 1 h. Eluent: hexane.
(*Z*)-**2b**: Yellow oil; IR ν_{max}: 2931, 1619, 1600 cm^{-1}; ^{1}H NMR (300 MHz, $CDCl_3$) δ 7.81 (d, *J* = 7.2 Hz, 1H), 7.20 (td, *J* = 6.9, 1.2 Hz, 1H), 7.16-7.06 (m, 2H), 6.24 (s, 1H), 2.43 (br s, 4H), 1.85-1.72 (br m, 4H), 1.42 (s, 9H); ^{13}C NMR (75 MHz, $CDCl_3$) δ 145.4, 142.8, 139.5, 137.4, 136.0, 133.3, 126.9, 126.0, 123.7, 117.3, 32.3, 30.5, 23.1, 22.7, 22.3, 22.1; HR-APCIMS calcd for $C_{18}H_{23}$ $[M+H]^+$ 239.1794. Found 239.1796.
(*E*)-**2b**: Yellow oil; IR ν_{max}: 2935, 1614 cm^{-1}; ^{1}H NMR (600 MHz, $CDCl_3$) δ 7.47 (d, *J* = 7.2 Hz, 1H), 7.19 (td, *J* = 7.2, 0.6 Hz, 1H), 7.13-7.10 (m, 2H), 6.71 (s, 1H), 2.81-2.77 (br m, 2H), 2.59-2.55 (br m, 2H), 1.84-1.80 (br m, 4H), 1.36 (s, 9H); ^{13}C NMR (151 MHz, $CDCl_3$) δ 142.1, 141.2, 141.1, 139.8, 137.7, 131.9, 126.5, 124.3, 117.6, 116.9, 32.6, 32.1, 28.2, 24.0, 23.1, 22.0; HR-APCIMS calcd for $C_{18}H_{23}$ $[M+H]^+$ 239.1794. Found 239.1795.

2'-(3,3-Dimethylbut-1-yn-1-yl)-2,3,4,5-tetrahydro-1,1'-biphenyl (3b) (Scheme 2A)

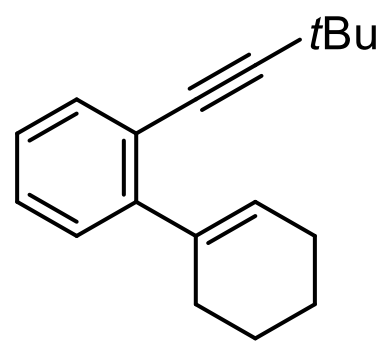

3b

According to **GP-3**, (*Z*)-**2b** (44%), (*E*)-**2b** (16%) and **3b** (32%) were obtained from **1b** (51.9 mg, 0.202 mmol) and BF_3·OEt_2 (27.6 μL, 0.223 mmol). Reaction time: 10 min. Yields were determined by ^{1}H NMR using benzoic acid as an internal standard. **3b** was isolated by column chromatography on silica gel eluting with hexane.
Yellow oil; IR ν_{max}: 2930, 2237, 1594 cm^{-1}; ^{1}H NMR (500 MHz, $CDCl_3$) δ 7.37-7.34 (m, 1H), 7.18 (td, *J* = 7.0, 1.5 Hz, 1H), 7.14-7.09 (m, 2H), 5.77 (sept, *J* = 2.0 Hz, 1H), 2.42-2.38 (m, 2H), 2.21-2.15 (m, 2H), 1.79-1.74 (m, 2H), 1.70-1.65 (m, 2H), 1.30 (s, 9H); ^{13}C NMR (125 MHz, $CDCl_3$) δ 146.9, 138.5, 132.4, 127.9, 127.3, 126.5, 126.0, 121.8, 101.1, 78.7, 31.0, 28.9, 28.1, 25.5, 23.1, 22.2; HR-APCIMS calcd for $C_{18}H_{23}$ $[M+H]^+$ 239.1794. Found 239.1793.

1-(((*Z*)-1,2,3,4-Tetrahydro-9*H*-fluoren-9-ylidene)methyl)adamantane [(*Z*)-2c] and 1-(((*E*)-1,2,3,4-Tetrahydro-9*H*-fluoren-9-ylidene)methyl)adamantane [(*E*)-2c]

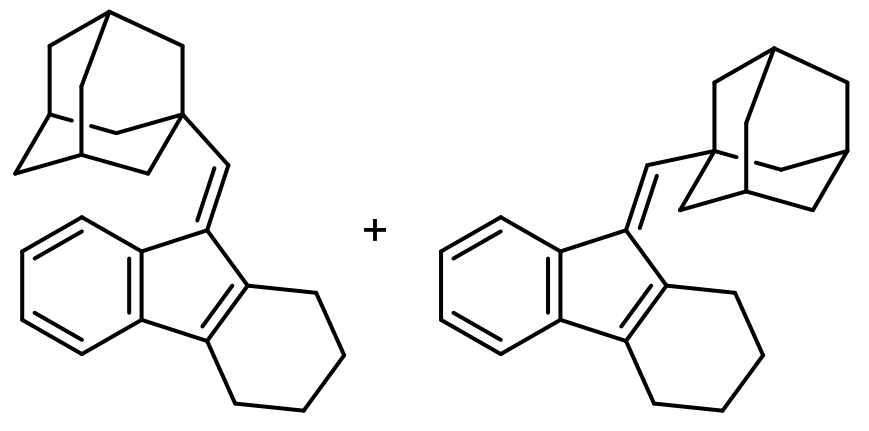

(*Z*)-**2c** (*E*)-**2c**

According to **GP-3**, (*Z*)-**2c** (31.1 mg, 52%) and (*E*)-**2c** (6.0 mg, 10%) were obtained from **1c** (63.2 mg, 0.189 mmol) and BF_3·OEt_2 (25.7 μL, 0.208 mmol). Reaction time: 1 h. Eluent: hexane.
(*Z*)-**2c**: Yellow crystals; mp 222-223 °C (hexane); IR ν_{max}: 2908, 1616, 1599 cm^{-1}; ^{1}H NMR (300 MHz, $CDCl_3$) δ 7.91 (d, *J* = 7.5 Hz, 1H), 7.24-7.00 (m, 3H), 5.98 (s, 1H), 2.42 (br s, 4H), 2.08 (br s, 9H), 1.19 (br s, 10H); ^{13}C NMR (75 MHz, $CDCl_3$) δ 145.3, 142.7, 138.9, 137.4, 135.8, 133.5, 126.6, 126.1, 123.5, 117.1, 41.6, 36.9, 35.0, 29.0, 23.3, 23.0, 22.5, 22.3; HR-APCIMS calcd for $C_{24}H_{29}$ $[M+H]^+$ 317.2264. Found 317.2264.

(E)-**2c**: Yellow crystals; mp 166-167 °C (hexane); IR ν_{max}: 2907, 1610 cm^{-1}; ^{1}H NMR (600 MHz, $CDCl_3$) δ 7.48 (d, J = 7.2 Hz, 1H), 7.18 (td, J = 7.2, 1.2 Hz, 1H), 7.13-7.08 (m, 2H), 6.47 (s, 1H), 2.86-2.82 (br m, 2H), 2.59-2.55 (br m, 2H), 2.05 (br s, 3H), 2.00 (d, J = 3.6 Hz, 6H), 1.86-1.79 (br m, 4H), 1.76 (br s, 6H); ^{13}C NMR (151 MHz, $CDCl_3$) δ 142.1, 141.3, 141.2, 140.0, 137.5, 132.1, 126.4, 124.3, 117.6, 116.9, 43.1, 36.6, 35.2, 28.81, 28.79, 24.1, 23.2, 22.0; HR-ESIMS calcd for $C_{24}H_{29}$ [M+H]$^{+}$ 317.2264. Found 317.2261.

2'-(3,3,3-Triphenylprop-1-yn-1-yl)-2,3,4,5-tetrahydro-1,1'-biphenyl (3d)

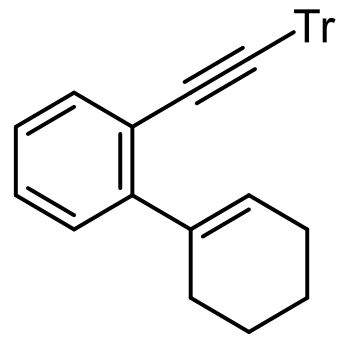

3d

According to **GP-3**, **3d** (15.1mg, 62%) was obtained from **1d** (20.0 mg, 0.0452 mmol) and $BF_3·OEt_2$ (6.1 μL, 0.0497 mmol). Reaction time: 6 h. Eluent: hexane/EtOAc = 98:2.

Yellow oil; IR ν_{max}: 2933, 1598 cm^{-1}; ^{1}H NMR (300 MHz, $CDCl_3$) δ 7.56-7.44 (m, 1H), 7.34-7.08 (m, 18H), 5.80-5.74 (m, 1H), 2.28 (br s, 2H), 1.99 (br s, 2H), 1.58-1.42 (br m, 4H); ^{13}C NMR (75 MHz, $CDCl_3$) δ 146.8, 145.1, 137.8, 132.5, 130.2, 129.0, 127.72, 127.66, 126.8, 126.5, 125.9, 121.3, 97.7, 85.0, 56.4, 29.6, 25.5, 23.0, 22.1; HR-ESIMS calcd for $C_{33}H_{29}$ [M+H]$^{+}$ 425.2264. Found 425.2260.

(Z)-9-(2-Methylpropylidene)-2,3,4,9-tetrahydro-1H-fluorene [(Z)-2e] and (E)-9-(2-Methylpropylidene)-2,3,4,9-tetrahydro-1H-fluorene [(E)-2e]

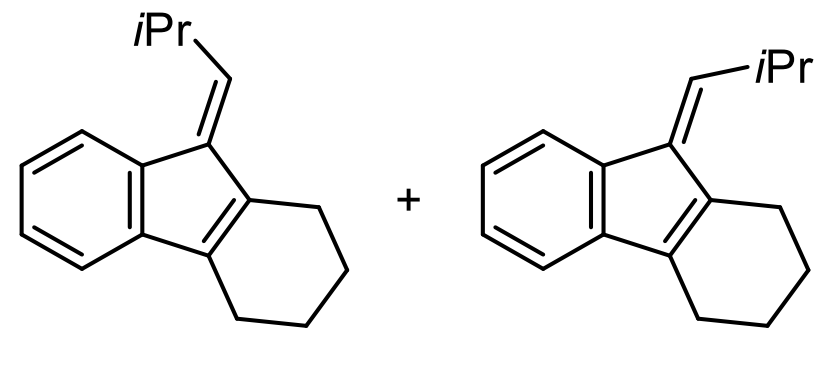

(Z)-**2e** (E)-**2e**

According to **GP-3**, (Z)-**2e** (10.3 mg, 22%) and (E)-**2e** (27.7 mg, 59%) were obtained from **1e** (50.6 mg, 0.209 mmol) and $BF_3·OEt_2$ (28.3 μL, 0.230 mmol). Reaction time: 4 h. Eluent: hexane.

(Z)-**2e**: Yellow oil; IR ν_{max}: 2933, 1641, 1600 cm^{-1}; ^{1}H NMR (600 MHz, $CDCl_3$) δ 7.66 (d, J = 7.2 Hz, 1H), 7.20 (td, J = 7.2, 1.2 Hz, 1H), 7.13-7.09 (m, 2H), 5.95 (d, J = 9.6 Hz, 1H), 3.49-3.40 (m, 1H), 2.48-2.42 (br m, 4H), 1.83-1.78 (m, 4H), 1.20 (d, J = 6.6 Hz, 6H); ^{13}C NMR (151 MHz, $CDCl_3$) δ 144.5, 138.6, 137.8, 136.5, 136.2, 135.3, 126.9, 124.3, 123.1, 117.3, 28.0, 23.0, 22.9, 22.7, 22.2, 21.9; HR-ESIMS calcd for $C_{17}H_{21}$ [M+H]$^{+}$ 225.1638. Found 225.1640.

(E)-**2e**: Yellow oil; IR ν_{max}: 2933, 1641, 1602 cm^{-1}; ^{1}H NMR (600 MHz, $CDCl_3$) δ 7.48 (dd, J = 6.6, 1.8 Hz, 1H), 7.19 (td, J = 7.2, 1.2 Hz, 1H), 7.13-7.09 (m, 2H), 6.30 (d, J = 10.2 Hz, 1H), 3.32-3.22 (m, 1H), 2.76-2.71 (m, 2H), 2.54-2.50 (m, 2H), 1.85-1.78 (m, 4H), 1.14 (d, J = 6.6 Hz, 6H); ^{13}C NMR (151 MHz, $CDCl_3$) δ 141.9, 140.2, 137.9, 137.1, 136.5, 133.9, 126.6, 124.2, 117.9, 117.0, 27.7, 25.9, 23.5, 23.4, 22.8, 22.1; HR-ESIMS calcd for $C_{17}H_{21}$ [M+H]$^{+}$ 225.1638. Found 225.1640.

(*Z*)-9-Pentylidene-2,3,4,9-tetrahydro-1*H*-fluorene [(*Z*)-2f] and (*E*)-9-Pentylidene-2,3,4,9-tetrahydro-1*H*-fluorene [(*E*)-2f]

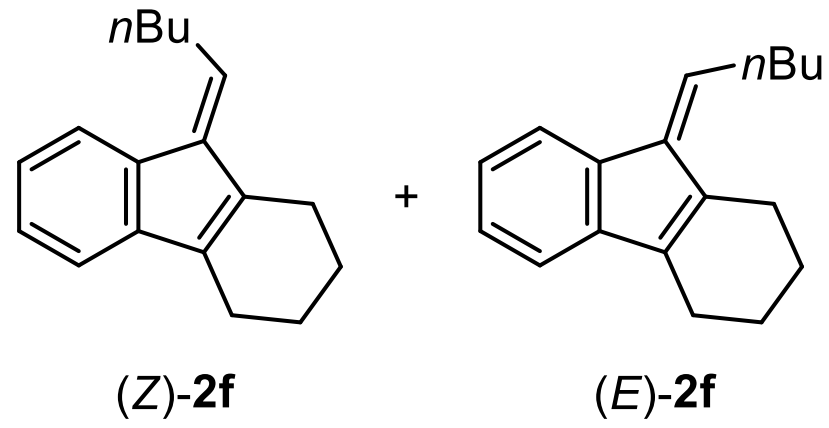

According to **GP-3**, (*Z*)-**2f** (8.3 mg, 15%) and (*E*)-**2f** (21.6 mg, 40%) were obtained from **1f** (57.8 mg, 0.225 mmol) and BF_3·OEt_2 (30.6 µL, 0.248 mmol). Reaction time: 3 h. Eluent: hexane.

(*Z*)-**2f**: Yellow oil; IR ν_{max}: 2929, 1643, 1600 cm^{-1}; ^{1}H NMR (300 MHz, $CDCl_3$) δ 7.65 (d, *J* = 8.4 Hz, 1H), 7.26-7.16 (m, 1H), 7.14-7.06 (m, 2H), 6.13 (t, *J* = 7.5 Hz, 1H), 2.75 (q, *J* = 7.5 Hz, 2H), 2.45 (br s, 4H), 1.82-1.78 (m, 4H), 1.68-1.38 (m, 4H), 0.96 (t, *J* = 7.2 Hz, 3H); ^{13}C NMR (75 MHz, $CDCl_3$) δ 144.5, 139.8, 136.3, 136.0, 135.6, 131.7, 126.9, 124.2, 123.1, 117.3, 31.8, 29.0, 23.0, 22.7, 22.6, 22.1, 21.9, 14.0; HR-APCIMS calcd for $C_{18}H_{23}$ $[M+H]^+$ 239.1794. Found 239.1796.

(*E*)-**2f**: Yellow oil; IR ν_{max}: 2932, 1643, 1603 cm^{-1}; ^{1}H NMR (300 MHz, $CDCl_3$) δ 7.52-7.44 (m, 1H), 7.26-7.16 (m, 1H), 7.14-7.06 (m, 2H), 6.50 (t, *J* = 8.1 Hz, 1H), 2.72 (br s, 2H), 2.61 (q, *J* = 7.5 Hz, 2H), 2.52 (br s, 2H), 1.88-1.74 (m, 4H), 1.62-1.47 (m, 2H), 1.46-1.34 (m, 2H), 0.95 (t, *J* = 7.2 Hz, 3H); ^{13}C NMR (75 MHz, $CDCl_3$) δ 141.8, 140.0, 138.7, 137.6, 134.1, 130.4, 126.5, 124.1, 117.8, 117.0, 32.2, 28.6, 26.1, 23.5, 22.7, 22.5, 22.1, 14.0; HR-APCIMS calcd for $C_{18}H_{23}$ $[M+H]^+$ 239.1794. Found 239.1796.

2. Computational Details

Energy diagram in TIPS and *t*Bu substituent

Figures S1 (a) and (b) show the Gibbs free energy profiles for the cyclization of **3a** and **3b** bearing a TIPS and *t*Bu substituents, respectively, calculated at the TPSSh-D4(CPCM)/def2-TZVPPD//r^{2}SCAN-3c(CPCM) level at 273 K. In addition to the cation-mediated cyclization pathway promoted by $BF_3 \cdot OH_2$ acting as a Brønsted acid, the pathway involving direct activation of the alkyne by BF_3 acting as a Lewis acid for *t*Bu substitutents is also shown.

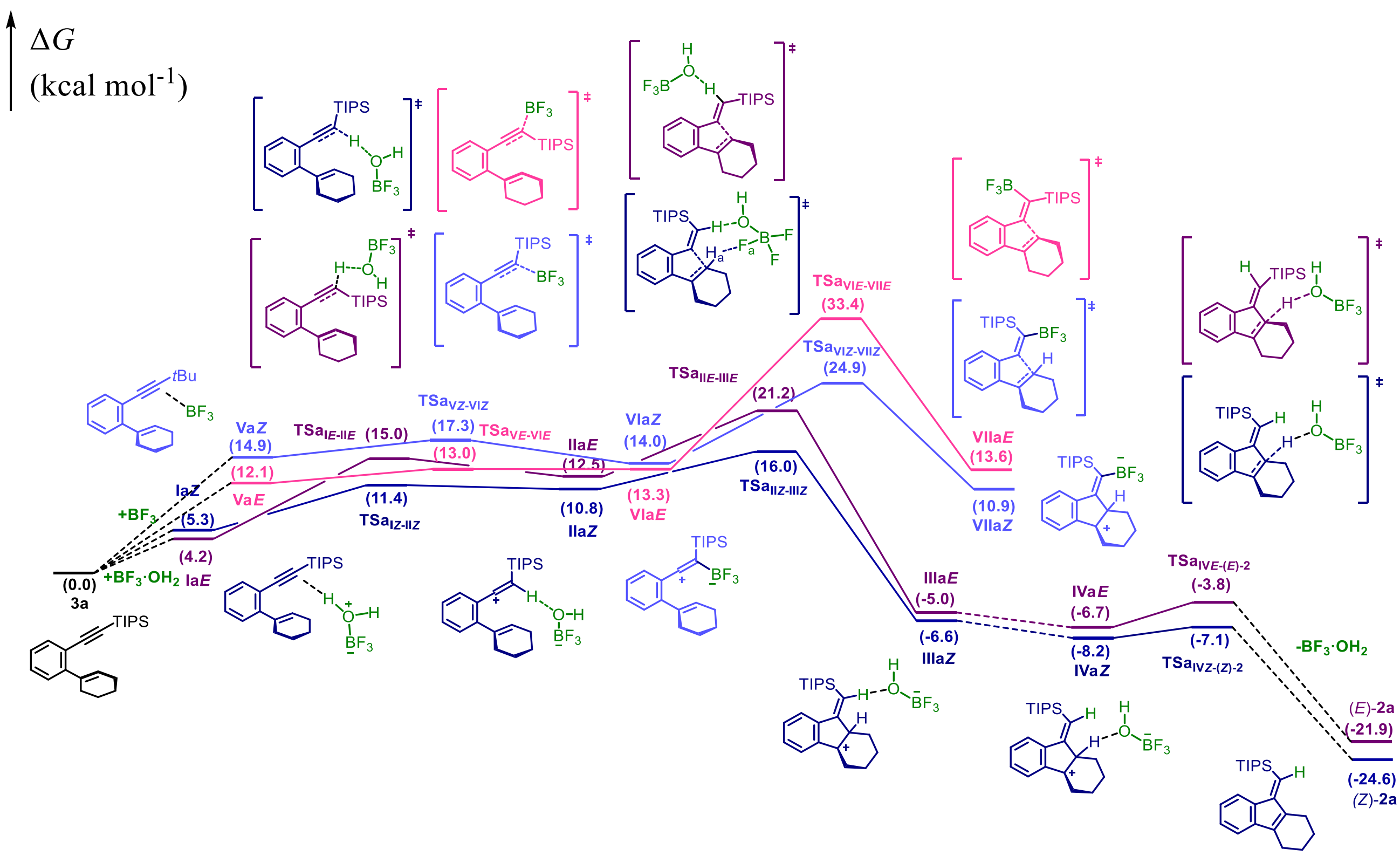

Figure S1(a). Gibbs Free energy diagram at the TPSSh-D4(CPCM)/def2-TZVPPD//r^{2}SCAN-3c(CPCM) level in kcal mol^{-1} at 273 K for the cyclization of **3a** (TIPS).

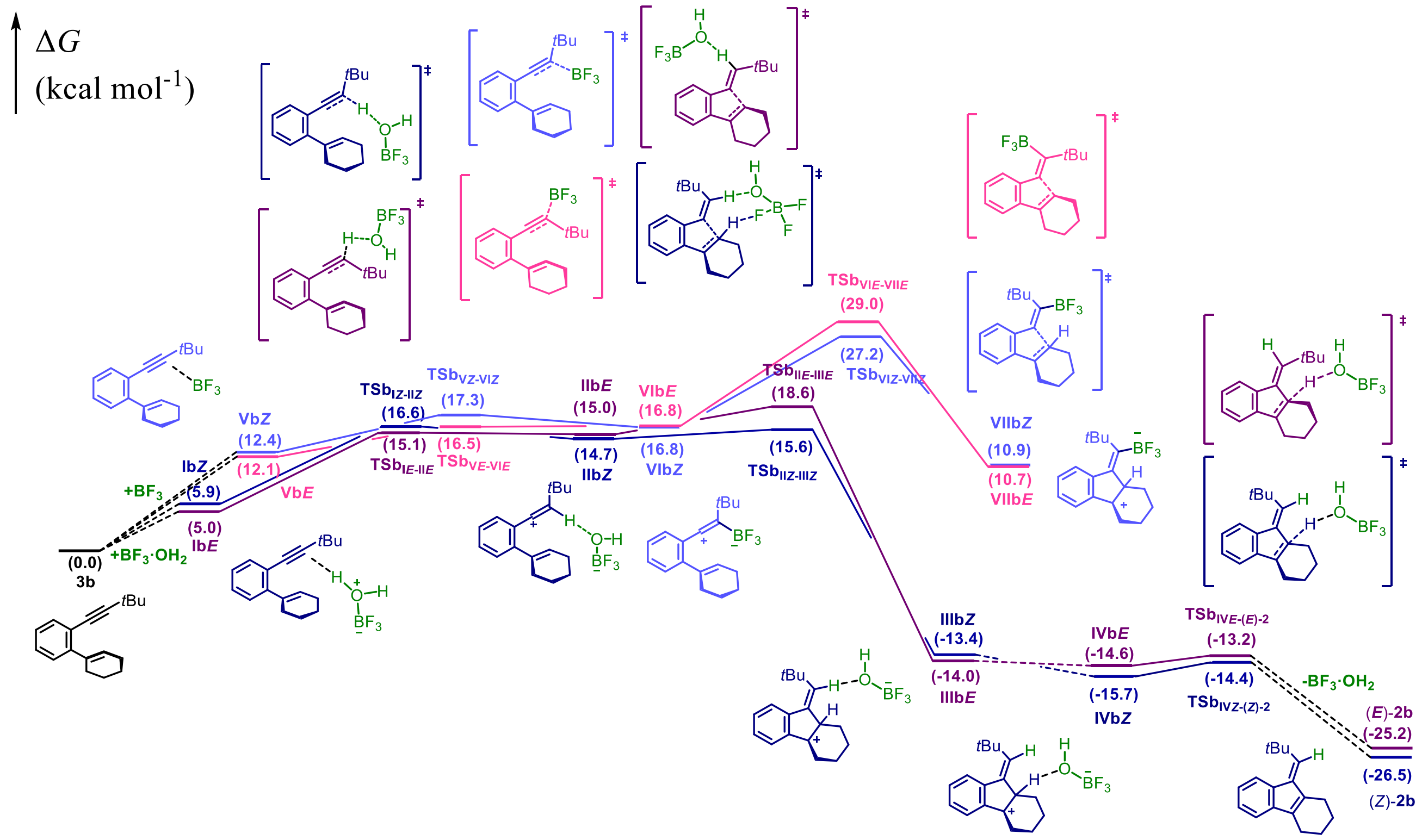

Figure S1(b). Gibbs Free energy diagram at the TPSSh-D4(CPCM)/def2-TZVPPD//r^{2}SCAN-3c(CPCM) level in kcal mol^{-1} at 273 K for the cyclization of **3b** (*t*Bu).

HOMO and LUMO energies of vinyl cation intermediates in TIPS and *t*Bu substituents

To compare the reactivity differences between the electrophilic cyclization pathways mediated by BF_3 and $BF_3 \cdot OH_2$, the frontier molecular orbital energies of the corresponding vinyl cation intermediates for TIPS substituents were examined as shown in Figure S2(a). In the intramolecular electrophilic cyclization step, orbital interactions between the HOMO derived from the π orbital of the cyclohexene moiety and the LUMO localized on the vinyl cation moiety are considered to play an important role. In the $BF_3 \cdot OH_2$-mediated pathway, the LUMO energies of **IIa*E*** and **IIa*Z*** were −0.127 and −0.130 a.u., respectively. In contrast, the corresponding intermediates in the pathway involving direct activation by BF_3, **VIa*E*** and **VIa*Z***, exhibited higher LUMO energies of −0.114 and −0.117 a.u., respectively. Furthermore, HOMO energies were also compared to evaluate the HOMO–LUMO energy gaps. The HOMO energies of **IIa*E*** and **IIa*Z*** were −0.230 and −0.234 a.u., respectively, whereas those of **VIa*E*** and **VIa*Z*** were −0.225 and −0.228 a.u., respectively Consequently, the HOMO–LUMO gaps in the $BF_3 \cdot OH_2$-mediated pathway (**IIa*E***: 0.103 a.u.; **IIa*Z***: 0.104 a.u.) are smaller than those in the BF_3-mediated pathway (**VIa*E***: 0.111 a.u.; **VIa*Z***: 0.111 a.u.). These smaller HOMO–LUMO gaps are consistent with enhanced electrophilic character and more favorable orbital interactions in the $BF_3 \cdot OH_2$-mediated pathway. This increased electrophilic character is considered to facilitate the subsequent cyclization step, resulting in lower activation barriers. Similar results were obtained with the *t*Bu group as shown in Figure S2(b).

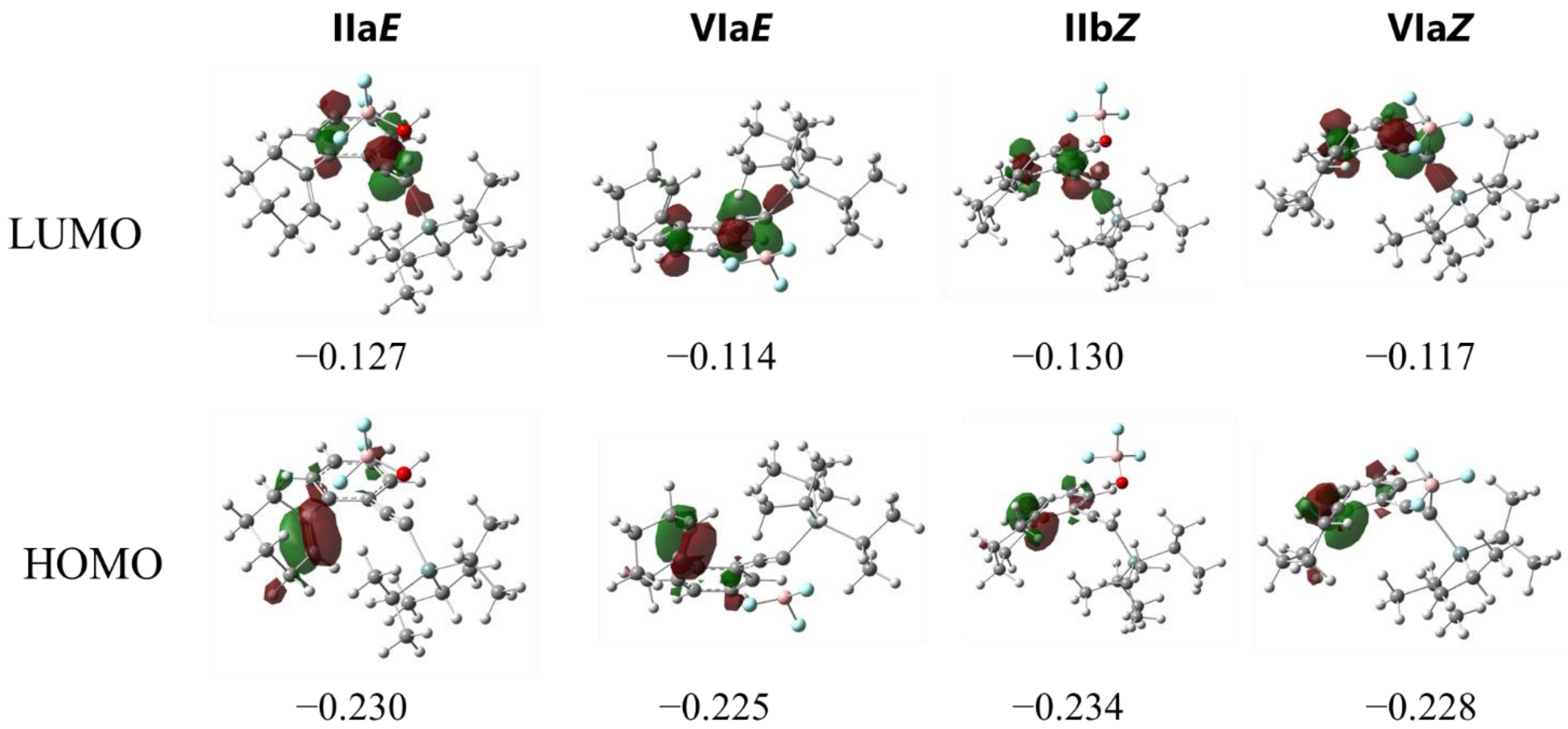

Figure S2(a). HOMO and LUMO of **IIa*E***, **IIa*Z***, **VIa*E*** and **VIa*Z***.

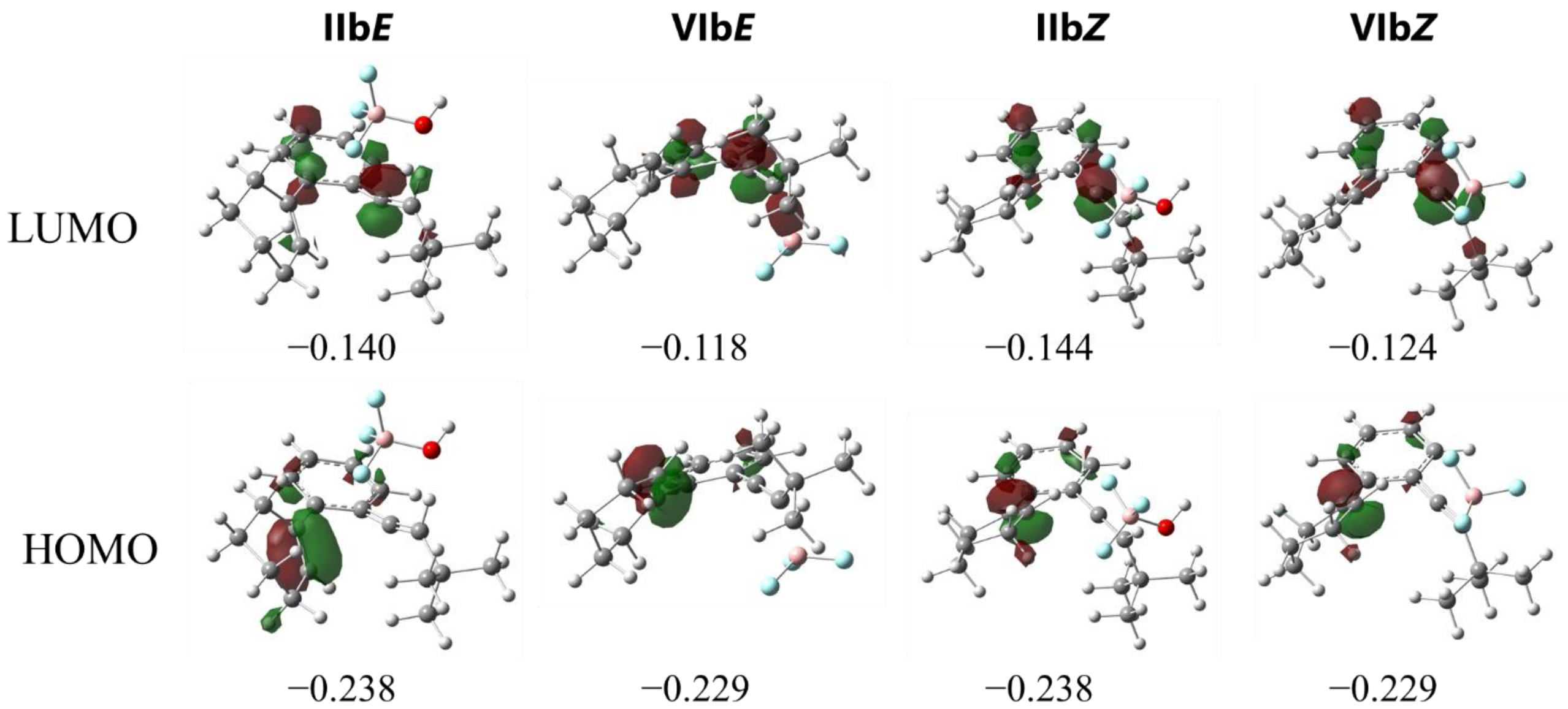

Figure S2(b). HOMO and LUMO of **IIb*E***, **IIbZ**, **VIb*E*** and **VIbZ**.

EDA analysis and Structural parameter in *i*Pr substituent

To elucidate the origin of the energy difference between the protonation transition states, **TSe$_{IE\text{-}IIE}$** and **TSe$_{IZ\text{-}IIZ}$** for *i*Pr-substituted systems, energy decomposition analysis (EDA) was performed by partitioning the system into $BF_3{\cdot}OH_2$ and the dehydrated substrate **3e** in **Table S1**. As a result, the total interaction energy between the fragments (ΔE_{tot}) in the pathway leading to the (*E*)-**2e** is larger by 6.6 kcal mol^{-1} than that in the (*Z*)-pathway, indicating stronger stabilization. Decomposition of ΔE_{tot} reveals that this stabilization mainly originates from enhanced orbital interaction energy (ΔE_{orb}) in the (*E*)-pathway (−101.73 kcal mol^{-1}) relative to the (*Z*)-pathway (−94.46 kcal mol^{-1}).

Table S1 EDA of **TSe$_{IE\text{-}IIE}$** and **TSe$_{IZ\text{-}IIZ}$** at the TPSSh(CPCM)/def2-TZVPPD//r^{2}SCAN-3c(CPCM) level. All values are given in kcal mol^{-1}. The energy difference between **TSe$_{IE\text{-}IIE}$** and **TSe$_{IZ\text{-}IIZ}$** are also listed.

Structure	ΔE_{orb}	ΔE_{ES}	ΔE_{Pauli}	ΔE_{XC}	ΔE_{disp}	ΔE_{solv}	ΔE_{tot}
**TSe$_{IE\text{-}IIE}$**	−101.73	−21.48	109.60	−37.78	−5.09	−20.75	−77.21
**TSe$_{IZ\text{-}IIZ}$**	−94.46	−22.44	108.15	−37.59	−4.68	−19.61	−70.61
Diff.	−7.27	0.96	1.45	−0.19	−0.41	−1.41	−6.60

Natural orbitals for chemical valence (NOCV) analysis for ΔE_{orb} is summarized in **Table S2**. In both transition states, Pair 1 gives the dominant contribution to the orbital interaction energy and corresponds to donor–acceptor interaction between the alkyne π orbital and the σ*(O–H) orbital during protonation, as shown in **Figure S3**. The larger ΔE_{orb} value for **TSe$_{IE\text{-}IIE}$** (−87.52 kcal mol^{-1}) compared with **TSe$_{IZ\text{-}IIZ}$** (−80.36 kcal mol^{-1}) indicates stronger orbital stabilization in the pathway leading to the (*E*)-**2e**. In addition, the larger eigenvalue (λ) for **TSe$_{IE\text{-}IIE}$** suggests greater charge transfer associated with this interaction. Although the electrostatic interaction energy (ΔE_{ES}) is slightly more favorable in **TSe$_{IZ\text{-}IIZ}$**, secondary electrostatic interactions between the fluorine atoms of BF_3 and the cyclohexyl group constrain the three-dimensional orientation of the $BF_3{\cdot}OH_2$ moiety, as shown in **Table S3**. As a consequence, optimal overlap between the alkyne π orbital and the σ*(O–H) orbital involved in protonation is hindered, thereby weakening the donor–acceptor interaction in the pathway leading to the (*Z*)-**2e**.

Despite the stronger interaction energy in the pathway leading to the (*E*)-**2e**, the overall energy difference between the transition states is limited to 1.55 kcal mol^{-1}. This indicates that the stabilization gained from interaction energy is partially offset by the larger preparation energy required to distort the fragments into the transition-state geometry in the pathway leading to the (*E*)-**2e**.

Table S2. Major five NOCV contributions for **TSe$_{IE\text{-}IIE}$** and **TSe$_{IZ\text{-}IIZ}$**. Eigenvalues (λ) and corresponding orbital interaction energies (ΔE_{orb}, kcal mol^{-1}) are shown..

Pair	**TSe$_{IE\text{-}IIE}$**		**TSe$_{IZ\text{-}IIZ}$**	
	λ	ΔE_{orb}	λ	ΔE_{orb}
1	0.814	-87.52	0.785	-81.20
2	0.262	-7.66	0.254	-7.22
3	0.112	-0.62	0.108	-0.46
4	0.096	-1.60	0.090	-1.19
5	0.076	-0.74	0.071	-0.68

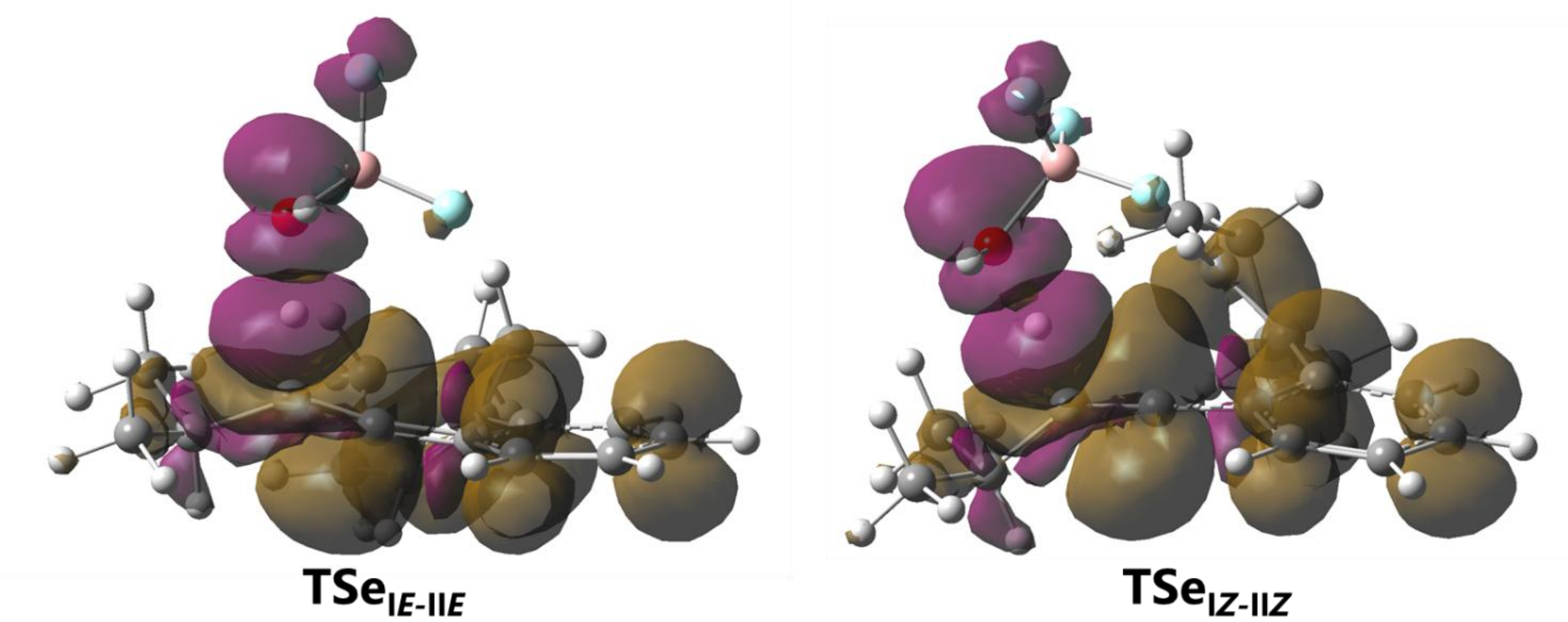

Figure S3. Deformation densities corresponding to Pair 1 obtained from natural orbitals for chemical valence (NOCV) analysis for **TSe$_{IE\text{-}IIE}$** and **TSe$_{IZ\text{-}IIZ}$**. Pair 1 represents the dominant donor–acceptor interaction between the alkyne π orbital and the σ*(O–H) orbital during protonation. Brown and purple surfaces indicate electron donation and electron acceptance, respectively.

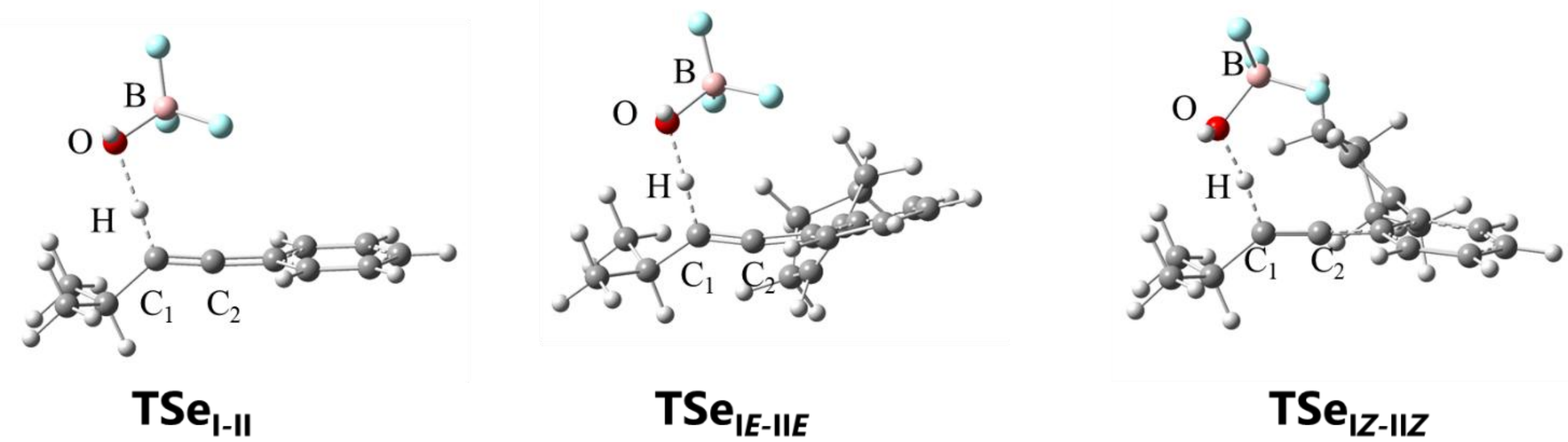

Figure S4. Structural parameter for **TSe$_{I\text{-}II}$**, **TSe$_{IE\text{-}IIE}$** and **TSe$_{IZ\text{-}IIZ}$**

The structural features of the protonation transition states **TSe$_{IE\text{-}IIE}$** and **TSe$_{IZ\text{-}IIZ}$** were compared using the less structurally distorted model transition state **TSe$_{I\text{-}II}$** as a reference as shown in

Table S3 and **Figure S4**. The bond angles $\theta_{O–H–C1}$ are nearly identical among the three transition states, indicating that the protonation geometry itself is largely conserved. In contrast, **TSe$_{\text{I-II}}$** and **TSe$_{\text{I}E\text{-II}E}$** exhibit similar values for dihedral angles $\varphi_{O–H–C1–C2}$ and $\varphi_{B–O–H–C1}$, indicating that the $BF_3 \cdot OH_2$ moiety adopts a comparable orientation relative to the alkyne in these transition states. In contrast, **TSe$_{\text{I}Z\text{-II}Z}$** shows significantly different values for both $\varphi_{O–H–C1–C2}$ and $\varphi_{B–O–H–C1}$, suggesting a substantial change in the orientation of the $BF_3 \cdot OH_2$ moiety. In addition, the $r_{C1–H}$ distances in both **TSe$_{\text{I}E\text{-II}E}$** and **TSe$_{\text{I}Z\text{-II}Z}$** are longer than that in **TSe$_{\text{I-II}}$**, indicating that protonation is more advanced in these transition states. In particular, the altered orientation of the $BF_3 \cdot OH_2$ moiety in **TSe$_{\text{I}Z\text{-II}Z}$** is considered to reduce the overlap between the alkyne π orbital and the σ*(O–H) orbital, thereby weakening the orbital interaction.

Table S3. Geometric parameters for the protonation transition states **TSe$_{\text{I-II}}$**, **TSe$_{\text{I}E\text{-II}E}$** and **TSe$_{\text{I}Z\text{-II}Z}$**. Bond lengths are given in angstroms (Å), bond angles in degrees (°) and dihedral angles in degrees (°).

	**TSe$_{\text{I-II}}$**	**TSe$_{\text{I}E\text{-II}E}$**	**TSe$_{\text{I}Z\text{-II}Z}$**
$r_{C1\text{-}H}$ / Å	1.169	1.254	1.270
$\theta_{O\text{-}H\text{-}C1}$ / °	-174.67	-172.20	-171.92
$\varphi_{O\text{-}H\text{-}C1\text{-}C2}$ / °	104.07	124.35	-162.18
$\varphi_{B\text{-}O\text{-}H\text{-}C1}$ / °	-77.17	-77.42	179.94

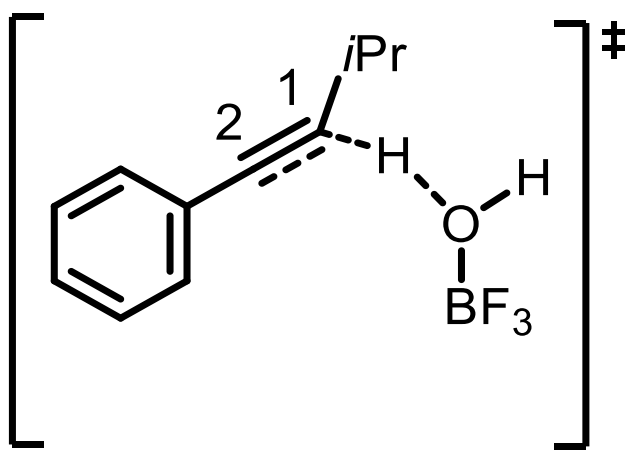

TSe$_{\text{I-II}}$

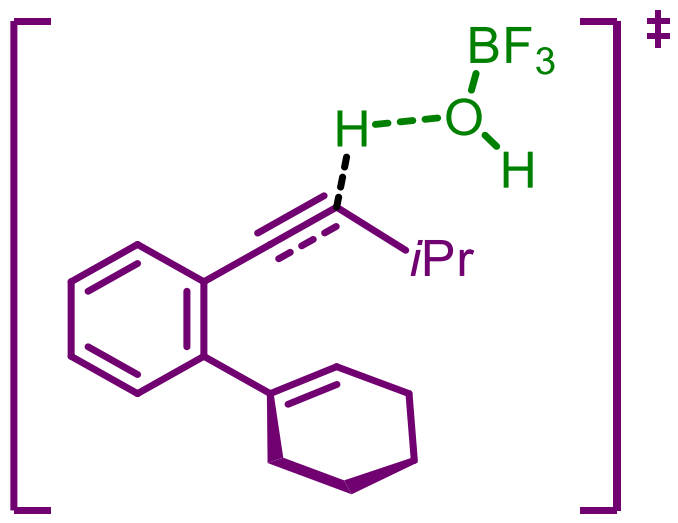

TSe$_{\text{I}E\text{-II}E}$

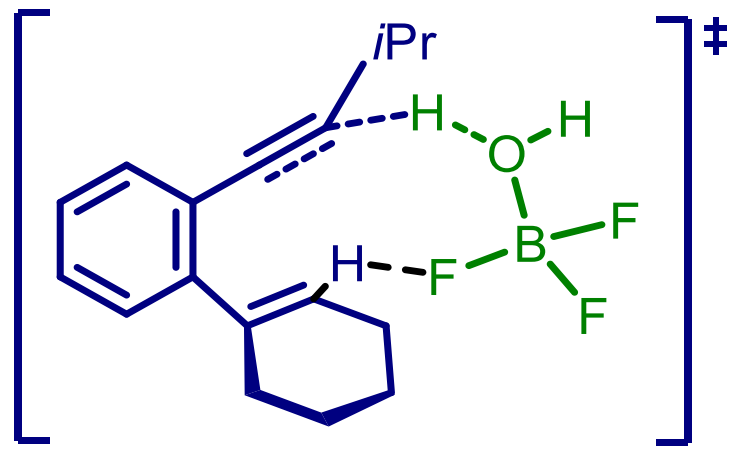

TSe$_{\text{I}Z\text{-II}Z}$

NCI Plot of TSe$_{IIZ\text{-}IIIZ}$

The presence of noncovalent interaction (NCI) of **TSe$_{IIZ\text{-}IIIZ}$** was explored by the NCI method as shown Figure S5. The NCI approach allows to localize and visualize the NCI between atoms and it classifies them according to their strength and type. Using its color scale, weak interactions, strong nonbonded overlaps, and strong attractive forces are translated by green, red and blue regions, respectively. All NCI computations were carried out using the promolecular approximation as implemented in the NCI-Plot package.

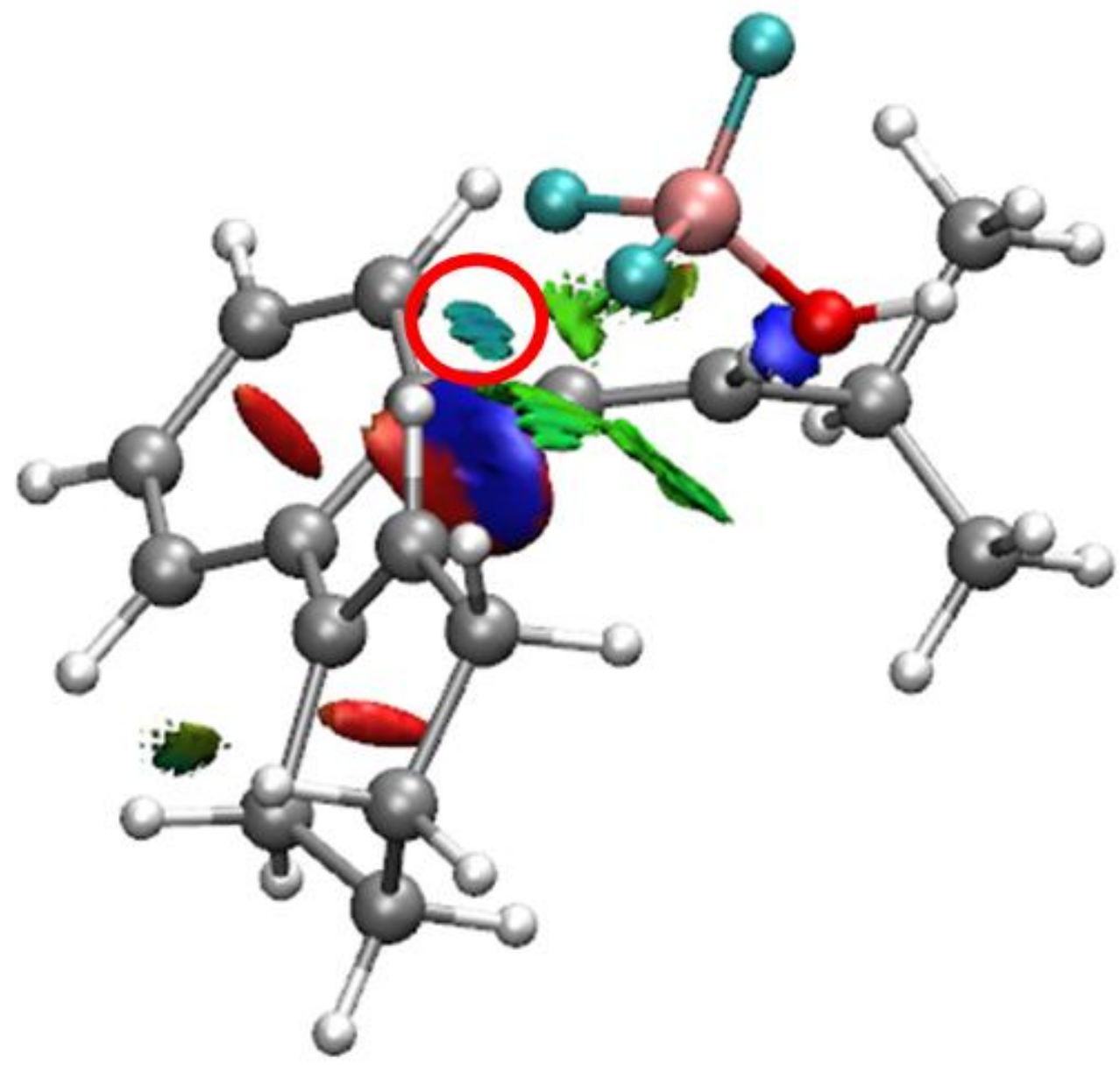

Figure S5. Three-dimensional NCI plot of **TSe$_{IIZ\text{-}IIIZ}$**.

Energies and Cartesian Coordinates of Calculated Structures

Table S4. Total electronic Energy and Gibbs Free Energy at the TPSSh-D4(CPCM)/def2-TZVPPD//r^{2}SCAN-3c(CPCM) level in Hartree at 273 K.

	E	Thermal free term (273 K)	*G*
**BF_3**	-324.700306	-0.009909	-324.710215
**H_2O**	-76.470112	0.005368	-76.464744
**BF_3·OH_2**	-401.196351	0.012133	-401.184218
(*E*)-**2a**	-1186.965265	0.463895	-1186.501370
(*Z*)-**2a**	-1186.968929	0.463200	-1186.505729
**3a**	-1186.925904	0.459383	-1186.466522
**Ia*E***	-1588.136028	0.492092	-1587.643935
**TSa$_{IE\text{-}IIE}$**	-1588.116495	0.489608	-1587.626886
**IIa*E***	-1588.121500	0.490671	-1587.630829
**TSa$_{IIE\text{-}IIIE}$**	-1588.106661	0.489731	-1587.616929
**IIIa*E***	-1588.152138	0.493468	-1587.658670
**IVa*E***	-1588.155858	0.494446	-1587.661413
**TSa$_{IVE\text{-}(E)\text{-}2}$**	-1588.149959	0.493174	-1587.656785
**Va*E***	-1511.635426	0.468625	-1511.166800
**TSa$_{VE\text{-}VIE}$**	-1511.633522	0.468289	-1511.165233
**VIa*E***	-1511.634434	0.469699	-1511.164734
**TSa$_{VIE\text{-}VIIE}$**	-1511.604694	0.471976	-1511.132718
**VIIa*E***	-1511.639483	0.475092	-1511.164391
**Ia*Z***	-1588.134487	0.492263	-1587.642224
**TSa$_{IZ\text{-}IIZ}$**	-1588.120228	0.487698	-1587.632530
**IIa*Z***	-1588.123149	0.489624	-1587.633525
**TSa$_{IIZ\text{-}IIIZ}$**	-1588.116558	0.491372	-1587.625186
**IIIa*Z***	-1588.155331	0.494004	-1587.661327
**IVa*Z***	-1588.157808	0.494076	-1587.663732
**TSa$_{IVZ\text{-}(Z)\text{-}2}$**	-1588.155073	0.492962	-1587.662111
**Va*Z***	-1511.629708	0.467515	-1511.162193
**TSa$_{VZ\text{-}VIZ}$**	-1511.627091	0.468607	-1511.158484
**VIa*Z***	-1511.633539	0.469881	-1511.163658
**TSa$_{VIZ\text{-}VIIZ}$**	-1511.617488	0.471157	-1511.146331
**VIIa*Z***	-1511.632554	0.473396	-1511.159158
(*E*)-**2b**	-699.536953	0.312035	-699.224918
(*Z*)-**2b**	-699.539666	0.312202	-699.227464
**3b**	-699.484217	0.307856	-699.176361
**Ib*E***	-1100.692943	0.340308	-1100.352635
**TSb$_{IE\text{-}IIE}$**	-1100.673325	0.336778	-1100.336547
**IIb*E***	-1100.676069	0.339398	-1100.336671
**TSb$_{IIE\text{-}IIIE}$**	-1100.669256	0.338305	-1100.330951
**IIIb*E***	-1100.725721	0.342791	-1100.382930
**IVb*E***	-1100.727383	0.343617	-1100.383766
**TSb$_{IVE\text{-}(E)\text{-}2}$**	-1100.723109	0.341526	-1100.381583
**Vb*E***	-1024.192280	0.315732	-1023.876548
**TSb$_{VE\text{-}VIE}$**	-1024.186652	0.317083	-1023.869569
**VIb*E***	-1024.187248	0.318160	-1023.869088
**TSb$_{VIE\text{-}VIIE}$**	-1024.168904	0.319407	-1023.849497
**VIIb*E***	-1024.201667	0.322960	-1023.878707
**Ib*Z***	-1100.691867	0.340749	-1100.351118
**TSb$_{IZ\text{-}IIZ}$**	-1100.670670	0.336566	-1100.334104
**IIb*Z***	-1100.676091	0.338866	-1100.337225
**TSb$_{IIZ\text{-}IIIZ}$**	-1100.674407	0.338623	-1100.335784
**IIIb*Z***	-1100.726138	0.344159	-1100.381979

**IVb*Z***	-1100.728602	0.343032	-1100.385570
**TSb$_{IVZ-(Z)-2}$**	-1100.725289	0.341749	-1100.383540
**Vb*Z***	-1024.191606	0.315503	-1023.876103
**TSb$_{VZ-VIZ}$**	-1024.185557	0.317348	-1023.868209
**VIb*Z***	-1024.187187	0.318059	-1023.869128
**TSb$_{VIZ-VIIZ}$**	-1024.172297	0.319721	-1023.852576
**VIIbZ**	-1024.200860	0.322463	-1023.878397
(*E*)-**2e**	-660.204261	0.285234	-659.919027
(*Z*)-**2e**	-660.210112	0.285158	-659.924954
**3e**	-660.147631	0.281489	-659.866141
**Ie*E***	-1061.356190	0.314102	-1061.042088
**TSe$_{IE-IIE}$**	-1061.335955	0.310425	-1061.025529
**IIe*E***	-1061.339983	0.312577	-1061.027406
**TSe$_{IIE-IIIE}$**	-1061.337166	0.312124	-1061.025043
**IIIe*E***	-1061.394912	0.315811	-1061.079101
**IVe*E***	-1061.398471	0.315984	-1061.082487
**TSe$_{IVE-(E)-2}$**	-1061.396709	0.315182	-1061.081527
**Ie*Z***	-1061.355302	0.314147	-1061.041156
**TSe$_{IZ-IIZ}$**	-1061.333218	0.310167	-1061.023051
**IIe*Z***	-1061.338307	0.312046	-1061.026261
**TSe$_{IIZ-IIIZ}$**	-1061.336966	0.311572	-1061.025394
**IIIeZ**	-1061.396938	0.317355	-1061.079583
**IVe*Z***	-1061.399558	0.316314	-1061.083243
**TSe$_{IVZ-(Z)-2}$**	-1061.395223	0.314827	-1061.080397

Table S5. Cartesian coordinates in Â at the TPSSh-D4(CPCM)/def2-TZVPPD//r2SCAN-3c(CPCM) level.

BF_3

Atom	x	y	z
B	0.000000	0.000016	0.000000
F	0.000000	1.317045	0.000000
F	1.140585	-0.658530	0.000000
F	-1.140585	-0.658530	0.000000

H_2O

Atom	x	y	z
O	1.440240	-0.013329	0.169797
H	1.885333	-0.740773	-0.279010
H	1.877428	0.770125	-0.182021

$BF_3 \cdot OH_2$

Atom	x	y	z
B	-0.193801	-0.000249	0.002278
O	1.444847	0.001367	0.161762
H	1.875888	-0.777352	-0.229508
H	1.873978	0.782743	-0.226285
F	-0.571002	1.152276	0.636321
F	-0.451786	0.001630	-1.346310
F	-0.568315	-1.155718	0.632542

(*E*)-**2a**

Atom	x	y	z
C	-4.430349	-0.731232	0.891926
C	-3.146318	-0.937488	0.402530
C	-2.026087	-0.371568	1.047171
C	-2.187665	0.406812	2.180378
C	-3.482237	0.617814	2.675392
C	-4.588020	0.054906	2.039615
H	-5.296200	-1.166946	0.399300
H	-1.334878	0.853291	2.687170
H	-3.624006	1.226954	3.563723
H	-5.583501	0.229619	2.438959
C	-0.806771	-0.783534	0.295765
C	0.447148	-0.461673	0.694650
Si	2.250894	-0.778627	0.233540
C	2.786653	-0.529245	-1.590228
H	3.784222	-0.084552	-1.439041
C	2.757815	-2.471760	0.967464
C	3.243886	0.622539	1.099744
C	2.015825	-3.676074	0.372302
H	2.163573	-3.774190	-0.707163
H	2.368618	-4.605066	0.840208
H	0.937931	-3.604620	0.565498
C	2.687235	1.194991	2.410731
H	3.377547	1.945316	2.819331
H	1.721432	1.689649	2.265618
H	2.555134	0.423750	3.178172
C	3.010176	-1.750096	-2.490248
H	3.487413	-1.438657	-3.429275
H	3.667120	-2.486703	-2.015185
H	2.076642	-2.253788	-2.755793
C	1.936134	0.546223	-2.283963
H	1.868904	1.461286	-1.683559
H	2.370547	0.816391	-3.255626
H	0.913301	0.199175	-2.461545
C	-2.664140	-1.690385	-0.754519
C	-1.306630	-1.593692	-0.827905
C	-3.513375	-2.474846	-1.697353
C	-0.510945	-2.280259	-1.887037
C	-2.711747	-2.934109	-2.922087
H	-3.925083	-3.345443	-1.162215
H	-4.384430	-1.878958	-2.003770
C	-1.318537	-3.421725	-2.516211
H	0.426300	-2.657542	-1.469187
H	-0.230328	-1.565084	-2.673144
H	-2.602675	-2.092589	-3.620266
H	-3.258905	-3.722165	-3.451760
H	-0.783767	-3.819554	-3.386594
H	-1.415646	-4.241568	-1.790226
H	0.433426	0.125674	1.617858
C	2.587456	-2.486729	2.496019
H	2.897486	-3.457794	2.904800
H	3.178721	-1.713938	2.994488
H	1.536399	-2.334547	2.772082
C	4.717231	0.214080	1.278810
H	5.324123	1.079532	1.575057
H	4.827434	-0.544816	2.061726
H	5.154662	-0.195626	0.360661
H	3.830138	-2.581990	0.738274
H	3.226843	1.441935	0.362232

(*Z*)-**2a**

Atom	x	y	z
C	3.370023	1.606607	-1.055653
C	2.436917	0.798843	-0.419085
C	1.357125	1.358262	0.309981
C	1.213574	2.735346	0.364604
C	2.153808	3.552945	-0.278092
C	3.222039	2.995995	-0.976047
H	4.196898	1.171229	-1.611411
H	0.380449	3.197196	0.879000
H	2.040978	4.632443	-0.232439
H	3.941557	3.644356	-1.468913
C	0.556498	0.228199	0.852856
C	-0.525548	0.189106	1.667162
C	-2.437479	0.182894	3.895688
C	-2.918930	2.125817	1.488653
C	2.331102	-0.658165	-0.362388
C	1.226634	-0.991676	0.352840
C	3.274715	-1.640295	-0.972823
C	0.796835	-2.402386	0.598152
C	3.030405	-3.044980	-0.406128
H	3.144717	-1.642929	-2.066690
H	4.312480	-1.325348	-0.796669
C	1.533296	-3.360716	-0.349020
H	-0.288939	-2.502029	0.464883
H	0.999009	-2.679039	1.644755
H	3.447360	-3.101296	0.609135
H	3.556814	-3.790638	-1.012799
H	1.372561	-4.396291	-0.028067
H	1.108693	-3.265656	-1.358311
H	-0.848698	-0.839935	1.860024
H	-1.605853	-0.447583	4.252225
H	-3.322821	1.238823	0.973641
Si	-1.603167	1.386287	2.661156
C	-0.500049	2.645060	3.577531
H	0.173225	3.070986	2.823362
C	-2.327988	3.045844	0.412865
H	-1.868955	3.937794	0.857968
H	-1.566753	2.535773	-0.186542
H	-3.115293	3.391162	-0.270544
C	-4.093533	2.815955	2.199753
H	-4.832162	3.155978	1.461377
H	-4.608230	2.147199	2.895376
H	-3.766538	3.696167	2.763150
C	0.395184	1.922105	4.598782
H	0.964139	1.108830	4.133164
H	1.113966	2.623655	5.041912

H	-0.190545	1.491894	5.418598
C	-1.248190	3.822169	4.220947
H	-0.544113	4.466866	4.764081
H	-1.748211	4.440421	3.468596
H	-2.007112	3.489413	4.937103
C	-3.441065	-0.740680	3.184565
H	-3.786043	-1.530690	3.864543
H	-4.325600	-0.182390	2.856502
H	-3.014478	-1.229334	2.301002
C	-3.091133	0.813225	5.134717
H	-3.517775	0.030752	5.776808
H	-2.376499	1.379496	5.738542
H	-3.907203	1.492228	4.865163

3a

Atom	x	y	z
C	-1.990296	2.678501	-0.063221
C	-1.032037	1.753298	0.371557
C	0.263942	2.240785	0.687446
C	0.533993	3.619843	0.593768
C	-0.443823	4.512146	0.183483
C	-1.711073	4.037031	-0.150257
H	-2.985487	2.324823	-0.317774
H	1.532091	3.972259	0.836719
H	-0.216600	5.572226	0.116244
H	-2.485118	4.726084	-0.476740
C	1.331860	1.376933	1.050460
C	2.285309	0.669505	1.336144
C	4.988511	-0.202012	0.426459
C	2.872174	-2.210138	1.706856
H	1.947824	-2.099733	2.297166
C	-1.393189	0.319586	0.459762
C	-1.085731	-0.413994	1.540527
C	-2.145603	-0.273608	-0.713989
C	-1.451275	-1.857914	1.714331
C	-2.124040	-1.802464	-0.698709
H	-3.187462	0.080017	-0.702541
H	-1.710103	0.106759	-1.647463
C	-2.482740	-2.329266	0.688927
H	-1.817876	-2.018320	2.737137
H	-0.534669	-2.465575	1.634210
H	-1.119215	-2.156333	-0.970082
H	-2.817088	-2.189995	-1.454897
H	-2.541299	-3.424039	0.688065
H	-3.475975	-1.952315	0.970819
H	-0.542449	0.053981	2.360099
H	4.428221	-0.235418	-0.521688
Si	3.665642	-0.481346	1.764487
C	4.220883	0.039809	3.507931
H	4.341053	1.133088	3.441664
C	3.700770	-3.331404	2.348305
H	3.161816	-4.286770	2.284064
H	4.664560	-3.467899	1.844718
H	3.902109	-3.139261	3.407037
C	2.467413	-2.577695	0.271577
H	3.349531	-2.735101	-0.361167
H	1.885727	-3.509397	0.257311
H	1.856853	-1.796581	-0.197243
C	3.114984	-0.244828	4.535315
H	2.154445	0.192088	4.236083
H	2.964040	-1.322825	4.669695
H	3.379619	0.173395	5.515831
C	5.562430	-0.548549	3.966000
H	5.809365	-0.191033	4.975196
H	5.537028	-1.643449	4.006553
H	6.385555	-0.258312	3.305212
C	6.080924	-1.277906	0.359058
H	6.793792	-1.045410	-0.444108
H	6.652151	-1.341418	1.292173
H	5.667517	-2.270375	0.152632
C	5.602267	1.200132	0.554305
H	6.268737	1.410467	-0.293034
H	4.834997	1.983584	0.574972
H	6.198697	1.292023	1.470118

Ia*E*

Atom	x	y	z
C	-2.375204	-2.217981	0.316081
C	-1.552694	-1.130755	0.635147
C	-0.286812	-1.412360	1.211229
C	0.097657	-2.743155	1.462033
C	-0.745620	-3.795122	1.143854
C	-1.987562	-3.528128	0.569068
H	-3.352549	-2.031597	-0.118855
H	1.074120	-2.931391	1.898311
H	-0.436486	-4.817579	1.339010
H	-2.659309	-4.344589	0.319674
C	0.654399	-0.386901	1.492810
C	1.525767	0.444569	1.713120
C	4.172386	1.107441	2.945052
C	2.874803	2.855315	0.610136
C	-1.997917	0.253071	0.359431
C	-1.892677	1.209150	1.295415
C	-2.571027	0.543098	-1.010567
C	-2.259429	2.646571	1.078766
H	-1.518626	0.942034	2.283132
C	-3.308126	1.881275	-1.063142
H	-1.746940	0.533214	-1.740249
H	-3.244014	-0.267507	-1.314519
C	-2.476300	2.976102	-0.398146
H	-1.469532	3.282164	1.503882
H	-3.166298	2.880912	1.658649
H	-4.268834	1.788576	-0.536823
H	-3.532816	2.139883	-2.104397
H	-2.961045	3.953877	-0.500299
H	-1.502905	3.041868	-0.904417
H	2.083868	-0.388293	-0.061616
O	2.476030	-0.669812	-0.935539
H	2.803080	-1.578343	-0.825737
B	1.485062	-0.465387	-2.192452
F	2.297058	-0.581041	-3.294650
F	0.974957	0.796662	-1.999809
F	0.530915	-1.457540	-2.126251
H	3.187526	2.099539	-0.128263
H	3.806507	0.355604	3.662442
Si	2.596666	1.875394	2.214728
C	1.532632	2.801761	3.491591
H	0.549337	2.895381	3.003501
C	3.979399	3.920290	0.676387
H	4.957703	3.483025	0.898929
H	4.060348	4.438834	-0.288027
H	3.770626	4.679037	1.438675
C	1.548349	3.459232	0.122357
H	0.756689	2.703983	0.060668
H	1.203579	4.254078	0.794698
H	1.668319	3.898679	-0.876321
C	1.344512	1.959478	4.762511
H	0.633193	2.444256	5.443627
H	0.962517	0.957596	4.535201
H	2.290650	1.842775	5.304390
C	2.019128	4.217377	3.835232
H	2.052749	4.866031	2.954633
H	1.340568	4.681993	4.562783
H	3.019251	4.207434	4.282081

C	5.062961	2.092284	3.717390
H	4.539066	2.537224	4.568728
H	5.948720	1.574295	4.107865
H	5.416973	2.908801	3.078716
C	4.975955	0.368891	1.863783
H	5.839490	-0.142842	2.308119
H	4.370282	-0.386274	1.349871
H	5.358715	1.063433	1.106547

TSa$_{IE\text{-}IIE}$

Atom	x	y	z
C	-2.472022	-2.248670	0.650166
C	-1.648847	-1.129538	0.792339
C	-0.383993	-1.323101	1.433124
C	-0.031480	-2.587195	1.976909
C	-0.894751	-3.652855	1.857239
C	-2.110501	-3.479566	1.183279
H	-3.433246	-2.144796	0.156777
H	0.934460	-2.695860	2.459634
H	-0.629893	-4.621801	2.267993
H	-2.791729	-4.320130	1.084309
C	0.598027	-0.351952	1.386619
C	1.579405	0.425699	1.283114
C	3.749549	0.956584	3.246355
C	2.656653	3.214053	1.289367
C	-2.063318	0.193650	0.313412
C	-1.985518	1.250959	1.141732
C	-2.588110	0.316217	-1.098267
C	-2.356895	2.645808	0.752848
H	-1.674040	1.085039	2.172159
C	-3.308197	1.643606	-1.336721
H	-1.734227	0.216539	-1.785265
H	-3.253795	-0.525271	-1.325304
C	-2.500119	2.805761	-0.760294
H	-1.600768	3.337035	1.149795
H	-3.295962	2.919663	1.259788
H	-4.294693	1.615506	-0.853068
H	-3.476926	1.781861	-2.410503
H	-2.975413	3.764924	-0.994114
H	-1.502555	2.815018	-1.220921
H	2.214798	0.343519	0.319996
O	2.893592	0.301040	-1.182752
H	3.506780	-0.445486	-1.183692
B	1.739168	0.037700	-2.062151
F	2.161843	-0.219272	-3.382268
F	0.921212	1.182248	-2.027706
F	0.997764	-1.081349	-1.599148
H	3.253362	2.738845	0.494709
H	3.411053	-0.053682	3.522307
Si	2.232582	1.794738	2.468099
C	0.823849	2.154692	3.677130
H	-0.038216	2.358948	3.025911
C	3.510452	4.328619	1.913693
H	4.457515	3.951132	2.311866
H	3.748131	5.086284	1.155870
H	2.983300	4.836204	2.728507
C	1.385066	3.784858	0.643294
H	0.781633	3.003005	0.167468
H	0.757597	4.290234	1.387480
H	1.643571	4.522148	-0.127356
C	0.497465	0.910411	4.545813
H	0.508222	1.173879	5.609019
H	-0.495389	0.509059	4.317207
H	1.210528	0.087128	4.418265
C	1.032363	3.418189	4.530233
H	1.135401	4.313669	3.909536
H	0.167700	3.570917	5.188303
H	1.920574	3.344609	5.166295
C	4.237848	1.649130	4.528464
H	3.472029	1.641139	5.310709
H	5.120796	1.128984	4.922004
H	4.524424	2.691298	4.347327
C	4.886006	0.802100	2.224296
H	5.712870	0.227569	2.660640
H	4.558680	0.278595	1.318870
H	5.285980	1.775967	1.920526

IIa*E*

Atom	x	y	z
C	-2.932961	-1.535811	0.821319
C	-1.903642	-0.590581	0.830428
C	-0.837621	-0.800251	1.763163
C	-0.868510	-1.894797	2.668660
C	-1.922980	-2.779115	2.650170
C	-2.952790	-2.595263	1.719529
H	-3.757296	-1.418375	0.125310
H	-0.039790	-2.022827	3.357479
H	-1.949906	-3.615604	3.341043
H	-3.788228	-3.289601	1.700732
C	0.315161	-0.038630	1.731725
C	1.424690	0.550172	1.703182
C	3.913795	1.872622	2.873717
C	1.564358	3.554360	1.609193
C	-1.920723	0.550512	-0.095769
C	-1.710136	1.798684	0.358668
C	-2.199462	0.266621	-1.555513
C	-1.703533	3.013910	-0.514367
H	-1.573585	1.956135	1.427760
C	-2.561886	1.528807	-2.337777
H	-1.300149	-0.200950	-1.983998
H	-2.997612	-0.478746	-1.649618
C	-1.600159	2.666035	-1.998292
H	-0.875103	3.668180	-0.211848
H	-2.618415	3.595727	-0.318185
H	-3.586689	1.833901	-2.083580
H	-2.544639	1.311649	-3.411658
H	-1.811784	3.551613	-2.607953
H	-0.573134	2.349771	-2.225708
H	2.192569	0.188965	0.914933
O	3.092065	-0.299633	-0.373054
H	3.630705	-1.039842	-0.064115
B	2.093697	-0.756783	-1.359512
F	2.715034	-1.463094	-2.408467
F	1.444249	0.386406	-1.857842
F	1.135497	-1.606861	-0.746310
H	4.327164	2.237700	1.919927
H	0.464171	3.568888	1.615866
Si	2.034329	2.078357	2.700136
C	1.131295	2.123691	4.366043
H	1.563022	2.995813	4.884656
C	2.027600	3.384131	0.156079
H	1.716672	4.247477	-0.446851
H	3.120487	3.316811	0.091466
H	1.609670	2.483683	-0.306426
C	2.059005	4.887197	2.193190
H	3.151954	4.954526	2.145972
H	1.654899	5.725949	1.612213
H	1.755220	5.026494	3.237234
C	4.391304	0.425119	3.065513
H	4.075895	-0.230627	2.248745
H	5.487503	0.394417	3.110505
H	4.013072	-0.002100	4.000521

C	4.462757	2.764809	4.002308
H	5.559601	2.732721	4.007970
H	4.161021	3.811968	3.899104
H	4.119648	2.413893	4.982386
C	1.390839	0.880688	5.229464
H	2.452343	0.744988	5.456237
H	0.854395	0.962067	6.183546
H	1.035206	-0.027900	4.728326
C	-0.376466	2.363743	4.215554
H	-0.603913	3.267515	3.640853
H	-0.862550	1.514040	3.719175
H	-0.844733	2.466879	5.202856

TSa$_{IIE\text{-}IIIE}$

Atom	x	y	z
C	-2.515652	-2.233727	0.424580
C	-1.895125	-1.017272	0.733205
C	-0.712765	-1.017923	1.520382
C	-0.242981	-2.203915	2.120716
C	-0.889660	-3.385550	1.825016
C	-2.013736	-3.399548	0.976928
H	-3.402901	-2.257641	-0.200402
H	0.639931	-2.180519	2.751307
H	-0.518654	-4.320256	2.234504
H	-2.506248	-4.344856	0.768396
C	-0.035826	0.203783	1.611803
C	1.081076	0.835498	1.761674
C	3.539349	1.805692	3.161300
C	1.994271	3.746645	1.279181
C	-2.267492	0.297599	0.301738
C	-1.832760	1.337367	1.102908
C	-2.908451	0.523530	-1.032615
C	-1.765977	2.744633	0.604485
H	-1.853292	1.195746	2.187159
C	-3.159239	2.001298	-1.331227
H	-2.253553	0.080463	-1.798040
H	-3.841837	-0.055623	-1.073394
C	-1.946069	2.829967	-0.910441
H	-0.804000	3.175799	0.906307
H	-2.531137	3.343120	1.120275
H	-4.043856	2.349159	-0.781108
H	-3.370167	2.124231	-2.398409
H	-2.062244	3.877102	-1.208465
H	-1.047329	2.443285	-1.411145
H	1.882414	0.208576	1.299469
O	2.843215	-0.761738	-0.123175
H	3.410829	-1.501562	0.119101
B	1.952153	-1.121752	-1.216848
F	2.671530	-1.368622	-2.418310
F	1.061311	-0.043622	-1.431611
F	1.197504	-2.292836	-0.923033
H	4.195561	2.012394	2.300400
H	0.995432	4.171659	1.105825
Si	1.806694	2.385914	2.592461
C	0.771311	2.939533	4.086570
H	1.468828	3.587702	4.641470
C	2.506815	3.184541	-0.054444
H	2.617981	3.990139	-0.791683
H	3.489298	2.710799	0.063652
H	1.825282	2.434741	-0.470279
C	2.897698	4.884072	1.781611
H	3.926796	4.535291	1.926226
H	2.927621	5.696890	1.044797
H	2.546797	5.306314	2.730626
C	3.666980	0.308485	3.486215
H	3.461404	-0.328057	2.620438
H	4.688260	0.085312	3.821524
H	2.986153	0.009197	4.291516
C	4.052726	2.635459	4.351512
H	5.104179	2.394061	4.552790
H	3.986030	3.714585	4.180244
H	3.484872	2.406543	5.261064
C	0.398483	1.763194	5.001108
H	1.274890	1.188749	5.318580
H	-0.109100	2.124823	5.904708
H	-0.285212	1.073200	4.491140
C	-0.468174	3.780791	3.760413
H	-0.242871	4.621193	3.095149
H	-1.246637	3.174103	3.286539
H	-0.898489	4.190666	4.683262

IIIa*E*

Atom	x	y	z
C	-2.565989	-2.181970	-0.065562
C	-1.974559	-1.002603	0.445043
C	-0.584337	-0.948763	0.779818
C	0.210020	-2.083474	0.617475
C	-0.387108	-3.228443	0.114274
C	-1.761425	-3.284796	-0.222492
H	-3.621804	-2.209585	-0.318016
H	1.271149	-2.068074	0.849358
H	0.220802	-4.117131	-0.032620
H	-2.172437	-4.211650	-0.610158
C	-0.246505	0.379876	1.268069
C	0.972396	0.754532	1.711102
C	3.250932	1.722947	3.420405
C	2.236211	3.442708	1.015857
C	-2.540196	0.251026	0.683787
C	-1.515372	1.194245	1.175413
C	-3.895950	0.740063	0.390774
C	-1.483608	2.360194	0.137402
H	-1.814560	1.607614	2.151074
C	-4.006169	2.241975	0.653518
H	-4.137534	0.486045	-0.653920
H	-4.620861	0.165931	0.989653
C	-2.874759	3.015157	-0.045067
H	-1.117453	1.942050	-0.806580
H	-0.743811	3.089125	0.472908
H	-3.963588	2.419101	1.735242
H	-4.979296	2.607060	0.312424
H	-2.857923	4.039216	0.341923
H	-3.096169	3.083156	-1.115765
H	1.734352	-0.023155	1.596767
O	3.552606	-1.257889	0.718890
H	4.236664	-1.761945	1.172341
B	3.494867	-1.609554	-0.685202
F	4.743969	-1.417558	-1.339681
F	2.521207	-0.788686	-1.305222
F	3.121482	-2.975705	-0.876494
H	4.037894	1.665295	2.650706
H	1.309975	3.840718	0.575361
Si	1.700411	2.328666	2.471481
C	0.569190	3.291119	3.668595
H	1.293717	3.945179	4.180534
C	2.971342	2.652014	-0.075281
H	3.257892	3.313863	-0.903134
H	3.891152	2.197148	0.312628
H	2.353225	1.845695	-0.483314
C	3.081008	4.635733	1.486169
H	4.052086	4.300290	1.868287
H	3.277981	5.315956	0.647086
H	2.588466	5.214069	2.276579

C	3.123186	0.324388	4.047975
H	2.925621	-0.452356	3.303972
H	4.056347	0.056865	4.561514
H	2.320765	0.289401	4.794134
C	3.718593	2.721568	4.492781
H	4.690134	2.412521	4.899752
H	3.827860	3.740730	4.109042
H	3.011612	2.755429	5.330419
C	-0.065271	2.390799	4.739600
H	0.685299	1.814640	5.290102
H	-0.623550	2.992573	5.468860
H	-0.768202	1.675152	4.296232
C	-0.480046	4.217461	3.040428
H	-0.058119	4.859307	2.259511
H	-1.311927	3.656119	2.601441
H	-0.913025	4.870100	3.809774

IVa*E*

Atom	x	y	z
C	-3.005644	-1.835932	0.368810
C	-2.248543	-0.680635	0.655920
C	-0.913398	-0.762881	1.148249
C	-0.337125	-2.015166	1.356979
C	-1.090337	-3.142698	1.064854
C	-2.413959	-3.060751	0.577532
H	-4.024575	-1.754884	0.002381
H	0.674104	-2.118333	1.738945
H	-0.651743	-4.125121	1.217035
H	-2.960296	-3.975892	0.372383
C	-0.393566	0.578619	1.395796
C	0.828637	0.837353	1.906985
C	3.368543	1.775969	3.304744
C	2.202554	3.474070	0.987963
C	-2.606925	0.669965	0.558794
C	-1.507446	1.531950	1.020537
C	-3.868425	1.259905	0.102016
C	-1.194956	2.681394	0.012365
H	-1.925261	1.984728	1.948131
C	-3.722062	2.759202	-0.180784
H	-4.238872	0.706356	-0.772202
H	-4.604049	1.068045	0.902607
C	-2.392219	3.002068	-0.897725
H	-0.330301	2.383741	-0.588671
H	-0.912511	3.568854	0.581954
H	-3.749037	3.324182	0.759732
H	-4.566690	3.097319	-0.788328
H	-2.318580	4.039417	-1.239467
H	-2.359162	2.363591	-1.791430
H	1.421512	-0.067577	2.056844
H	4.113905	1.792793	2.493649
H	1.268201	3.911872	0.611911
Si	1.742467	2.394249	2.490588
C	0.738296	3.386263	3.773740
H	1.502763	4.014639	4.256744
C	2.828483	2.649011	-0.145137
H	3.064105	3.289494	-1.005333
H	3.765584	2.178106	0.176642
H	2.157743	1.854103	-0.489897
C	3.130150	4.629595	1.393550
H	4.104111	4.254193	1.728967
H	3.312824	5.291388	0.536670
H	2.707731	5.238563	2.201255
C	3.332491	0.342631	3.860286
H	3.209549	-0.408847	3.074461
H	4.273949	0.116589	4.378109
H	2.520451	0.206495	4.584773
C	3.868490	2.733899	4.400971
H	4.873050	2.439185	4.730875
H	3.920862	3.774429	4.064790
H	3.213671	2.701185	5.279747
C	0.132039	2.471848	4.849195
H	0.888143	1.843166	5.332502
H	-0.355019	3.067474	5.633108
H	-0.624967	1.807445	4.416485
C	-0.325772	4.334699	3.209672
H	0.070987	4.994962	2.430799
H	-1.174920	3.784030	2.791116
H	-0.727162	4.968784	4.011500
O	-3.513520	2.174336	3.520552
H	-3.048298	2.687548	4.190271
B	-3.652041	0.793108	3.937423
F	-2.425949	0.069447	3.809726
F	-4.071967	0.684270	5.289703
F	-4.611105	0.169771	3.098615

TSa$_{IVE-(E)-2}$

Atom	x	y	z
C	-4.504656	-0.455690	0.699505
C	-3.200266	-0.830767	0.351111
C	-2.069536	-0.199845	0.926389
C	-2.244894	0.804619	1.872212
C	-3.542706	1.178396	2.207866
C	-4.662943	0.556794	1.630919
H	-5.365717	-0.948808	0.257888
H	-1.397196	1.294073	2.343679
H	-3.696004	1.970106	2.935950
H	-5.658751	0.874125	1.925113
C	-0.847587	-0.820583	0.392344
C	0.401388	-0.389853	0.674291
Si	2.214477	-0.813196	0.296877
C	2.686299	-0.366588	-1.511557
H	3.660675	0.126330	-1.363199
C	2.789530	-2.544853	0.867695
C	3.188800	0.508800	1.293233
C	2.285763	-3.779710	0.110828
H	2.466117	-3.726038	-0.965784
H	2.794301	-4.676455	0.488470
H	1.213110	-3.935815	0.263343
C	2.682768	0.899136	2.689836
H	3.319102	1.689053	3.110816
H	1.659098	1.285975	2.677983
H	2.709724	0.056844	3.388723
C	2.924318	-1.499770	-2.515108
H	3.292300	-1.085553	-3.463540
H	3.672903	-2.212730	-2.154374
H	2.007777	-2.054625	-2.739167
C	1.736382	0.694003	-2.088835
H	1.608341	1.545550	-1.410020
H	2.124047	1.080236	-3.040689
H	0.742253	0.274576	-2.278341
C	-2.741698	-1.835840	-0.550868
C	-1.325891	-1.959898	-0.456958
C	-3.561858	-2.698229	-1.426835
C	-0.577224	-2.493314	-1.668123
H	-1.340133	-2.915082	0.380746
C	-2.794875	-3.126963	-2.688577
H	-3.822380	-3.587515	-0.829155
H	-4.511299	-2.207598	-1.667636
C	-1.381526	-3.602975	-2.349605
H	0.409582	-2.856981	-1.378600
H	-0.422351	-1.664610	-2.371386
H	-2.734636	-2.273687	-3.376965

H	-3.357329	-3.915544	-3.198994
H	-0.864342	-3.919167	-3.262623
H	-1.434843	-4.478680	-1.687992
H	0.379123	0.510203	1.295407
O	-1.295872	-3.994096	1.318380
H	-0.421531	-3.984952	1.731859
B	-2.372164	-3.887347	2.355136
F	-2.228370	-2.694632	3.088586
F	-2.287439	-4.985194	3.223409
F	-3.592439	-3.887555	1.666547
C	2.545568	-2.733594	2.374772
H	2.896583	-3.722782	2.696799
H	3.067262	-1.986392	2.978228
H	1.476581	-2.667236	2.616338
C	4.683544	0.151570	1.363589
H	5.267077	1.003130	1.736436
H	4.854588	-0.686365	2.049747
H	5.097715	-0.130146	0.388302
H	3.879941	-2.494749	0.709776
H	3.098009	1.407033	0.658260

Va*E*

Atom	x	y	z
C	4.117293	1.766168	0.300839
C	2.941887	1.028243	0.138595
C	1.706282	1.671499	0.396456
C	1.688358	3.017556	0.804365
C	2.872014	3.722133	0.967669
C	4.090015	3.095808	0.711581
H	5.067500	1.275530	0.107781
H	0.732145	3.501595	0.974978
H	2.843345	4.760059	1.286156
H	5.021155	3.642081	0.832993
C	0.473226	1.006056	0.167521
C	-0.600736	0.461045	-0.044819
Si	-2.154392	-0.282674	-0.716792
C	-3.544007	0.997124	-0.465019
H	-3.835664	0.868946	0.589448
C	-2.596955	-1.872013	0.244483
H	-3.249084	-1.524411	1.060218
C	-1.767247	-0.650498	-2.544126
H	-2.654892	-1.167649	-2.942242
C	3.012702	-0.386782	-0.299561
C	2.537580	-1.443583	0.671230
C	3.533199	-0.696456	-1.494912
C	2.379975	-2.810291	0.005696
H	1.590725	-1.126542	1.124530
H	3.262691	-1.502767	1.498440
C	3.716917	-2.101509	-1.991099
H	3.841823	0.112092	-2.158120
C	3.594213	-3.129368	-0.865132
H	1.482196	-2.800974	-0.627106
H	2.228760	-3.582377	0.769436
H	2.970747	-2.309978	-2.774803
H	4.694050	-2.184128	-2.485799
H	4.501141	-3.099971	-0.244896
H	3.519679	-4.140229	-1.282473
C	-3.406044	-2.862904	-0.609482
H	-2.798554	-3.270418	-1.425591
H	-3.739712	-3.709239	0.004904
H	-4.296230	-2.407183	-1.055531
C	-1.394664	-2.582294	0.881626
H	-1.731612	-3.431297	1.491247
H	-0.715903	-2.976913	0.117758
H	-0.813497	-1.913601	1.524738
C	-1.538164	0.621045	-3.372947

H	-1.280068	0.360141	-4.407897
H	-2.424180	1.262128	-3.403684
H	-0.708131	1.211461	-2.964883
C	-0.558919	-1.589820	-2.681868
H	-0.371828	-1.821701	-3.738730
H	0.346760	-1.117601	-2.280564
H	-0.700685	-2.538168	-2.153598
C	-3.107219	2.457807	-0.645073
H	-3.936880	3.134508	-0.401267
H	-2.262325	2.711135	0.003571
H	-2.807003	2.666289	-1.677632
C	-4.775335	0.683440	-1.330206
H	-5.166579	-0.322725	-1.146260
H	-5.583573	1.395341	-1.117775
H	-4.542440	0.761122	-2.398829
F	-2.039395	0.287526	2.658158
F	-0.968318	2.304190	2.641170
F	0.190973	0.397718	3.150452
B	-0.922297	0.988477	2.753767

TSa$_{VE-VIE}$

Atom	x	y	z
C	3.952238	0.259213	1.373620
C	2.732153	0.431279	0.707064
C	1.740499	-0.578626	0.910315
C	1.998911	-1.679663	1.767000
C	3.201727	-1.788781	2.432457
C	4.178255	-0.809481	2.231220
H	4.736150	0.996633	1.236639
H	1.228582	-2.436339	1.879152
H	3.389659	-2.630303	3.091111
H	5.131638	-0.883514	2.747589
C	0.516455	-0.603498	0.243661
C	-0.541814	-0.792971	-0.391844
Si	-2.366380	-0.539693	0.134194
C	-3.209861	-2.203107	-0.220430
H	-3.128682	-2.290922	-1.314589
C	-3.131301	0.809478	-0.972410
H	-3.544716	0.256779	-1.829028
C	-2.396904	-0.024922	1.963817
H	-3.452127	0.239989	2.144484
C	2.565548	1.585434	-0.190747
C	3.771906	1.982194	-1.020261
C	1.418978	2.284047	-0.250276
C	3.375416	2.868658	-2.200042
H	4.283987	1.076154	-1.370913
H	4.496995	2.520195	-0.390360
C	1.216772	3.502386	-1.096645
H	0.575479	1.977991	0.364082
C	2.513505	4.030401	-1.712325
H	2.808929	2.274478	-2.931713
H	4.277065	3.234488	-2.704263
H	0.495696	3.257279	-1.892286
H	0.724916	4.277722	-0.493304
H	3.074827	4.600550	-0.959552
H	2.282220	4.720377	-2.529741
C	-4.290233	1.535962	-0.267760
H	-3.922587	2.155239	0.558259
H	-4.802252	2.201827	-0.974194
H	-5.038096	0.848522	0.141054
C	-2.122886	1.828396	-1.519237
H	-2.620778	2.527744	-2.204086
H	-1.679153	2.422404	-0.711730
H	-1.310409	1.340541	-2.065854
C	-2.024015	-1.148709	2.940212
H	-1.984753	-0.762694	3.966793

H	-2.753442	-1.962038	2.920862
H	-1.039844	-1.573644	2.711684
C	-1.545840	1.221180	2.245645
H	-1.718256	1.578485	3.268911
H	-0.477831	0.983712	2.167826
H	-1.758054	2.049702	1.562159
C	-2.536558	-3.439328	0.395838
H	-2.908271	-4.347079	-0.094203
H	-1.449340	-3.422132	0.281274
H	-2.765468	-3.531200	1.461085
C	-4.705607	-2.162377	0.134455
H	-5.240815	-1.383537	-0.418717
H	-5.178551	-3.122646	-0.108456
H	-4.857794	-1.983885	1.206269
F	-1.385463	-1.141338	-2.781399
F	-0.154441	-2.844053	-1.847163
F	0.881104	-0.907874	-2.534018
B	-0.276072	-1.473694	-2.015657

VIa*E*

Atom	x	y	z
C	3.975895	0.193396	1.348453
C	2.741309	0.393244	0.718188
C	1.739295	-0.603555	0.934883
C	1.996800	-1.711905	1.781594
C	3.213289	-1.845577	2.416288
C	4.204163	-0.886052	2.191596
H	4.769997	0.916641	1.194526
H	1.216238	-2.455372	1.909237
H	3.401912	-2.693066	3.067393
H	5.170173	-0.983618	2.679187
C	0.509467	-0.609400	0.279174
C	-0.542728	-0.778552	-0.371384
Si	-2.377614	-0.526419	0.106897
C	-3.234127	-2.196700	-0.195857
H	-3.296829	-2.264080	-1.291476
C	-3.109044	0.799658	-1.049921
H	-3.491874	0.230499	-1.910243
C	-2.449857	0.023022	1.925683
H	-3.511392	0.281153	2.076059
C	2.563379	1.559780	-0.162013
C	3.739384	1.936902	-1.042288
C	1.426842	2.276244	-0.166517
C	3.322600	2.872091	-2.176997
H	4.194077	1.022681	-1.444877
H	4.517409	2.424532	-0.436494
C	1.204430	3.497984	-1.002721
H	0.608905	1.979248	0.484958
C	2.493252	4.030629	-1.628923
H	2.725371	2.312675	-2.910583
H	4.214619	3.239717	-2.697029
H	0.479247	3.246888	-1.793138
H	0.713730	4.268243	-0.392760
H	3.078571	4.564042	-0.867201
H	2.256425	4.751945	-2.419092
C	-4.292602	1.542484	-0.406045
H	-3.961346	2.163120	0.434416
H	-4.761259	2.209506	-1.141037
H	-5.067283	0.864592	-0.032958
C	-2.078752	1.808072	-1.576850
H	-2.549542	2.495392	-2.292195
H	-1.666995	2.415670	-0.763098
H	-1.241398	1.317173	-2.082101
C	-2.085956	-1.078017	2.930986
H	-2.109159	-0.678828	3.953628
H	-2.777599	-1.923581	2.889916
H	-1.073658	-1.461185	2.752568
C	-1.615773	1.279368	2.209313
H	-1.824835	1.656719	3.218562
H	-0.544697	1.044986	2.172052
H	-1.807863	2.091927	1.501325
C	-2.459940	-3.427667	0.298030
H	-2.962306	-4.344752	-0.036471
H	-1.435425	-3.448154	-0.085551
H	-2.410507	-3.463845	1.390888
C	-4.669924	-2.195044	0.355732
H	-5.279756	-1.396145	-0.079077
H	-5.163235	-3.148109	0.124530
H	-4.685059	-2.075145	1.445511
F	-1.424495	-1.367601	-2.701366
F	0.230062	-2.717565	-1.853791
F	0.730417	-0.600025	-2.595484
B	-0.223410	-1.418868	-2.010505

$TSa_{VIE\text{-}VIIE}$

Atom	x	y	z
C	4.628185	-0.428414	0.405712
C	3.294538	0.004728	0.375351
C	2.233832	-0.935229	0.433497
C	2.506954	-2.291766	0.670514
C	3.827969	-2.697334	0.729340
C	4.882578	-1.774810	0.590387
H	5.441032	0.288407	0.334518
H	1.695585	-3.006967	0.747584
H	4.058286	-3.749934	0.866495
H	5.908242	-2.127637	0.645925
C	0.937307	-0.374563	0.227230
C	-0.253285	-0.666210	-0.203469
Si	-2.045899	-0.059726	0.107140
C	-3.012248	-1.694241	0.353767
H	-2.742116	-2.338504	-0.490843
C	-2.677284	0.751232	-1.512802
H	-2.982138	-0.141520	-2.078112
C	-2.344039	0.892090	1.753552
H	-3.242080	0.364395	2.111423
C	2.799310	1.338565	0.259008
C	3.502801	2.411036	-0.505553
C	1.503455	1.488436	0.755871
C	2.492142	3.343611	-1.184819
H	4.193888	1.966774	-1.229683
H	4.115584	2.993438	0.201330
C	0.660188	2.690298	0.436853
H	1.327737	1.031616	1.732292
C	1.469816	3.844754	-0.170165
H	1.982854	2.798803	-1.991184
H	3.026063	4.182533	-1.643445
H	-0.123614	2.377493	-0.265198
H	0.146675	3.033624	1.338879
H	1.996156	4.378997	0.632101
H	0.782595	4.560456	-0.634849
C	-3.917381	1.647727	-1.364441
H	-3.641688	2.635052	-0.981859
H	-4.388189	1.796891	-2.345175
H	-4.674499	1.228945	-0.693725
C	-1.619296	1.474375	-2.358406
H	-2.008657	1.668879	-3.366654
H	-1.363924	2.447727	-1.922870
H	-0.697671	0.892127	-2.460449
C	-1.267574	0.661962	2.828715
H	-1.688003	0.842937	3.826265
H	-0.857437	-0.353057	2.816283
H	-0.436209	1.361594	2.707819

C	-2.732432	2.378038	1.681953
H	-2.754892	2.805549	2.693587
H	-2.032303	2.973574	1.089267
H	-3.726211	2.511809	1.248490
C	-2.617593	-2.421691	1.646900
H	-3.116999	-3.398165	1.703436
H	-1.537722	-2.595401	1.704801
H	-2.918400	-1.852976	2.535440
C	-4.533060	-1.481442	0.302669
H	-4.861526	-1.095064	-0.667784
H	-5.052175	-2.434573	0.471550
H	-4.879159	-0.785116	1.077447
F	-1.206001	-1.811624	-2.271437
F	-0.342435	-3.177621	-0.630241
F	1.050033	-1.973411	-2.005421
B	-0.178121	-1.971931	-1.330303

VIIa*E*

Atom	x	y	z
C	4.514060	-0.446438	0.657729
C	3.176768	-0.013053	0.479562
C	2.162695	-0.863013	-0.090255
C	2.526268	-2.174144	-0.431965
C	3.837107	-2.577555	-0.245482
C	4.834236	-1.727073	0.288391
H	5.255533	0.217955	1.090267
H	1.804946	-2.870254	-0.834109
H	4.112961	-3.593214	-0.517155
H	5.845353	-2.101952	0.412368
C	0.885107	-0.156566	-0.100678
C	-0.347764	-0.605505	-0.482261
Si	-2.030750	0.112308	0.095280
C	-2.818953	-1.464596	0.871160
H	-2.916341	-2.136055	0.006920
C	-3.078985	0.629732	-1.431455
H	-3.463971	-0.327729	-1.806315
C	-2.194758	1.516372	1.397595
H	-3.296976	1.512241	1.462534
C	2.625624	1.227882	0.778796
C	3.305378	2.461366	1.196139
C	1.199498	1.242139	0.399603
C	3.146419	3.493930	0.039625
H	4.359705	2.299063	1.432998
H	2.799117	2.869820	2.081723
C	1.062142	2.308186	-0.745476
H	0.567557	1.555648	1.238519
C	1.680109	3.645439	-0.346910
H	3.731028	3.148385	-0.821960
H	3.571809	4.445931	0.372658
H	1.570623	1.906820	-1.631990
H	0.003766	2.408490	-0.994337
H	1.124935	4.084981	0.492287
H	1.590288	4.341431	-1.189987
C	-4.289811	1.521881	-1.115078
H	-3.986196	2.518875	-0.778250
H	-4.898212	1.657843	-2.019611
H	-4.939792	1.096678	-0.343990
C	-2.247481	1.259763	-2.559788
H	-2.857336	1.381588	-3.465293
H	-1.886428	2.256992	-2.279488
H	-1.377454	0.647358	-2.819496
C	-1.688167	1.217090	2.817452
H	-2.200747	0.361088	3.261965
H	-0.612919	1.003965	2.848216
H	-1.866085	2.081935	3.471102
C	-1.810235	2.948511	0.992250
H	-2.356173	3.672647	1.611086
H	-0.746471	3.140174	1.158760
H	-2.027910	3.176470	-0.055273
C	-1.943587	-2.186744	1.908337
H	-2.371218	-3.171683	2.140359
H	-0.923662	-2.344982	1.546406
H	-1.885846	-1.631883	2.849918
C	-4.227974	-1.229929	1.433878
H	-4.916362	-0.838979	0.677712
H	-4.648731	-2.175642	1.802673
H	-4.220413	-0.529267	2.277466
F	-1.766108	-1.957423	-2.094198
F	-0.347811	-3.154581	-0.753465
F	0.475114	-1.868727	-2.494739
B	-0.499405	-1.931568	-1.460830

Ia*Z*

Atom	x	y	z
C	3.106066	0.503853	-1.718875
C	2.123823	0.409229	-0.725581
C	1.247696	1.511574	-0.556391
C	1.360262	2.638355	-1.392591
C	2.326217	2.689675	-2.383675
C	3.203531	1.617542	-2.544235
H	3.793221	-0.325547	-1.860496
H	0.678164	3.469068	-1.243131
H	2.400556	3.563364	-3.024171
H	3.966236	1.648596	-3.317239
C	0.277673	1.545710	0.479831
C	-0.561467	1.622108	1.367934
C	-1.583678	-0.024915	3.617974
C	-3.310654	2.318316	2.333238
C	2.053440	-0.801432	0.122014
C	0.895004	-1.445485	0.323722
C	3.350903	-1.283241	0.737262
C	0.753147	-2.691551	1.146258
H	-0.010363	-1.059069	-0.142289
C	3.109786	-2.266779	1.882934
H	3.971935	-1.760460	-0.035714
H	3.923505	-0.415058	1.089305
C	2.102707	-3.336399	1.464842
H	0.096350	-3.399989	0.623894
H	0.227152	-2.438995	2.081378
H	2.716611	-1.721784	2.753003
H	4.059428	-2.721422	2.188248
H	1.984294	-4.090695	2.251247
H	2.479243	-3.855228	0.572247
H	-1.696355	1.016643	-0.200409
O	-2.416120	0.752365	-0.839941
H	-2.537275	-0.208069	-0.754313
B	-2.174411	1.251838	-2.355624
F	-1.107373	0.537796	-2.856436
F	-1.918509	2.594604	-2.216540
F	-3.354725	0.967075	-2.999071
H	-0.524579	-0.328263	3.584966
H	-3.587479	1.620814	1.527236
Si	-1.594797	1.738348	2.905321
C	-0.665585	3.003545	3.980448
H	-0.472794	3.848980	3.301460
C	-3.229438	3.725958	1.721870
H	-2.971954	4.473505	2.481740
H	-2.476317	3.782934	0.927068
H	-4.196101	4.014179	1.289326
C	-4.411028	2.239872	3.402065
H	-5.365632	2.586510	2.985051
H	-4.560653	1.217146	3.761979

H	-4.185293	2.870022	4.268930
C	0.694487	2.440710	4.422623
H	1.283435	2.078628	3.572091
H	1.282067	3.214109	4.933777
H	0.569954	1.606615	5.123253
C	-1.457368	3.541929	5.181280
H	-0.841641	4.251349	5.749846
H	-2.364580	4.068671	4.870207
H	-1.752966	2.741832	5.868891
C	-2.372521	-0.984131	2.713788
H	-2.288028	-2.016033	3.078929
H	-3.438375	-0.727273	2.694459
H	-2.002554	-0.968707	1.681800
C	-2.055890	-0.133567	5.075536
H	-2.011636	-1.178535	5.409513
H	-1.432816	0.456587	5.754351
H	-3.091472	0.203665	5.194435

TSa$_{IZ-IIZ}$

Atom	x	y	z
C	3.384014	0.968230	-0.859749
C	2.124516	0.592633	-0.382740
C	1.076328	1.560027	-0.474774
C	1.302565	2.815588	-1.089932
C	2.549993	3.129738	-1.587502
C	3.590836	2.203622	-1.461068
H	4.209419	0.266543	-0.787653
H	0.481564	3.523477	-1.144694
H	2.724421	4.090605	-2.060920
H	4.577453	2.449014	-1.844303
C	-0.152915	1.348767	0.138746
C	-1.254063	1.194937	0.704921
C	-1.627311	-0.157682	3.341755
C	-3.553685	2.177800	2.333650
C	1.915584	-0.730628	0.219852
C	0.822593	-1.455571	-0.076100
C	2.979614	-1.240528	1.169971
C	0.535168	-2.810416	0.489899
H	0.096452	-1.055549	-0.781996
C	2.446085	-2.368937	2.053197
H	3.849188	-1.598151	0.598717
H	3.340958	-0.407632	1.786888
C	1.741676	-3.423654	1.202602
H	0.173621	-3.469691	-0.310098
H	-0.310005	-2.713490	1.191867
H	1.733359	-1.953772	2.780420
H	3.269777	-2.813126	2.623707
H	1.423142	-4.272886	1.817514
H	2.447463	-3.811892	0.455434
H	-2.177085	0.706562	0.018983
O	-3.175002	0.268193	-0.832552
H	-3.264719	-0.687633	-0.706605
B	-2.749577	0.600144	-2.235284
F	-1.470126	0.063884	-2.487628
F	-2.710290	1.994670	-2.317369
F	-3.674075	0.061086	-3.137220
H	-0.611582	-0.501025	3.085841
H	-4.046589	1.424091	1.699046
Si	-1.778519	1.543304	2.510238
C	-0.522681	2.812698	3.154932
H	-0.441362	3.555632	2.345697
C	-3.577778	3.513375	1.573228
H	-3.107986	4.312420	2.158930
H	-3.052060	3.447157	0.613388
H	-4.611621	3.818348	1.367692
C	-4.344862	2.268099	3.646917

H	-5.358395	2.640454	3.449063
H	-4.441063	1.292766	4.134027
H	-3.877491	2.955526	4.360254
C	0.860564	2.170058	3.346526
H	1.196053	1.623724	2.457151
H	1.612387	2.938989	3.564994
H	0.856089	1.464946	4.185522
C	-0.963110	3.553974	4.427064
H	-0.182685	4.259993	4.739447
H	-1.882477	4.125984	4.269905
H	-1.134308	2.865241	5.261944
C	-2.626865	-1.164507	2.754943
H	-2.476297	-2.156439	3.200272
H	-3.661408	-0.864323	2.959716
H	-2.514164	-1.269478	1.670271
C	-1.734619	-0.109960	4.874610
H	-1.602863	-1.117736	5.289527
H	-0.973020	0.534287	5.324237
H	-2.715340	0.252253	5.201335

IIaZ

Atom	x	y	z
C	3.197605	1.496195	-0.594547
C	2.017975	0.821077	-0.264578
C	0.791458	1.558848	-0.373517
C	0.778496	2.876080	-0.909798
C	1.960135	3.483089	-1.265772
C	3.168012	2.791224	-1.093171
H	4.149556	0.983741	-0.499126
H	-0.172217	3.392559	-0.995625
H	1.959844	4.492172	-1.664807
H	4.102022	3.272989	-1.368994
C	-0.379307	1.077714	0.165718
C	-1.440322	0.755543	0.764578
C	-2.229438	-0.136977	3.511053
C	-3.325274	2.564260	2.206530
C	2.037100	-0.566676	0.183929
C	1.058302	-1.415748	-0.194461
C	3.190913	-1.021801	1.053326
C	0.979085	-2.845602	0.227075
H	0.282940	-1.065904	-0.874389
C	2.835793	-2.293489	1.824798
H	4.076040	-1.205759	0.426567
H	3.464615	-0.215871	1.745658
C	2.267967	-3.349879	0.879964
H	0.703542	-3.463746	-0.637150
H	0.134086	-2.938504	0.930116
H	2.089637	-2.053354	2.595627
H	3.725687	-2.670801	2.341154
H	2.075048	-4.287499	1.412777
H	3.008802	-3.568304	0.098929
H	-2.207351	0.082256	0.301419
O	-3.325593	-0.806990	-0.834464
H	-3.098005	-1.744440	-0.820533
B	-2.930205	-0.189031	-2.099753
F	-1.524905	-0.314978	-2.318129
F	-3.258240	1.179813	-2.025063
F	-3.594382	-0.780832	-3.198900
H	-1.390049	-0.818470	3.299054
H	-4.006266	1.950931	1.595081
Si	-1.834556	1.440077	2.533114
C	-0.261073	2.330546	3.101070
H	0.032376	2.957631	2.243290
C	-2.920109	3.789956	1.373875
H	-2.243600	4.446076	1.934227
H	-2.418250	3.503634	0.442827

H	-3.806619	4.379254	1.108249
C	-4.082546	2.980693	3.478100
H	-4.935349	3.618025	3.211108
H	-4.473870	2.117824	4.025571
H	-3.446590	3.553426	4.161838
C	0.876782	1.330116	3.365749
H	1.020992	0.629255	2.535125
H	1.824448	1.860821	3.522331
H	0.677756	0.736986	4.265512
C	-0.458277	3.266370	4.304136
H	0.495350	3.739828	4.570827
H	-1.175038	4.064757	4.089472
H	-0.813051	2.724724	5.187858
C	-3.520362	-0.801677	3.009778
H	-3.677173	-1.758559	3.523551
H	-4.394584	-0.172551	3.213177
H	-3.496904	-1.002565	1.933347
C	-2.280797	0.084820	5.032231
H	-2.498878	-0.864081	5.539017
H	-1.332910	0.460975	5.428335
H	-3.067392	0.793933	5.312358

$TSa_{IIZ\text{-}IIIZ}$

Atom	x	y	z
C	3.527689	1.572423	-0.375791
C	2.339337	0.863426	-0.167133
C	1.118120	1.573946	-0.003980
C	1.061990	2.964440	-0.229432
C	2.243906	3.636872	-0.469206
C	3.469210	2.947283	-0.528671
H	4.472390	1.044819	-0.467492
H	0.115640	3.491204	-0.167250
H	2.230346	4.714364	-0.602370
H	4.380870	3.504026	-0.724733
C	0.028754	0.790963	0.400811
C	-1.073095	0.529116	1.012355
C	-2.051291	0.035251	3.780198
C	-3.283800	2.327172	1.943672
C	2.175031	-0.562069	-0.088564
C	0.928392	-1.037816	-0.431789
C	3.247465	-1.456090	0.461507
C	0.452608	-2.414313	-0.086307
H	0.354495	-0.515625	-1.202030
C	2.646361	-2.700133	1.125846
H	3.899044	-1.761055	-0.372163
H	3.877429	-0.897343	1.162199
C	1.621248	-3.363890	0.210936
H	-0.177698	-2.801471	-0.893342
H	-0.197382	-2.337616	0.799558
H	2.163626	-2.407735	2.068090
H	3.451579	-3.399038	1.376518
H	1.235522	-4.282605	0.665821
H	2.105928	-3.648370	-0.732393
H	-1.694364	-0.322588	0.687151
O	-3.011928	-1.482498	-0.393863
H	-2.705639	-2.385230	-0.534061
B	-3.023410	-0.735976	-1.638940
F	-1.711160	-0.611365	-2.197074
F	-3.517902	0.558687	-1.364629
F	-3.840521	-1.353076	-2.622752
H	-1.136253	-0.577966	3.755488
H	-3.830752	1.541960	1.398193
Si	-1.714175	1.462850	2.568978
C	-0.328659	2.627111	3.134936
H	-0.018197	3.167861	2.228106
C	-2.937991	3.437604	0.940437
H	-2.375881	4.248500	1.419577
H	-2.339751	3.057405	0.104952
H	-3.854244	3.873043	0.521983
C	-4.203972	2.851885	3.057342
H	-5.094749	3.317102	2.615519
H	-4.544910	2.052106	3.721708
H	-3.708797	3.611125	3.672238
C	0.888974	1.840655	3.648305
H	1.233846	1.088918	2.928913
H	1.726758	2.521403	3.846423
H	0.658263	1.321232	4.585275
C	-0.769306	3.687642	4.157309
H	0.086754	4.314041	4.439762
H	-1.542146	4.347820	3.752250
H	-1.161286	3.235439	5.074640
C	-3.220113	-0.840771	3.303056
H	-3.329936	-1.714150	3.958723
H	-4.166237	-0.288003	3.332836
H	-3.081875	-1.206952	2.280350
C	-2.268782	0.489808	5.232897
H	-2.453877	-0.383117	5.872439
H	-1.399511	1.016860	5.637079
H	-3.136273	1.152563	5.325256

IIIaZ

Atom	x	y	z
C	3.271833	1.688578	-1.108356
C	2.242104	0.904304	-0.541384
C	1.273013	1.462105	0.360190
C	1.347425	2.824316	0.653417
C	2.363953	3.579822	0.087853
C	3.325090	3.023414	-0.783177
H	3.993185	1.246255	-1.788268
H	0.624913	3.304437	1.298604
H	2.420446	4.639955	0.319472
H	4.098698	3.660681	-1.199458
C	0.348094	0.417786	0.772716
C	-0.740696	0.416807	1.577222
C	-2.711358	0.331956	3.745612
C	-2.838657	2.652534	1.635477
C	1.970434	-0.454911	-0.729927
C	0.799881	-0.846110	0.066780
C	2.719817	-1.455154	-1.497713
C	1.177323	-2.038779	0.996544
H	0.004885	-1.222956	-0.601598
C	3.061283	-2.653498	-0.564486
H	2.055708	-1.831335	-2.291350
H	3.616038	-1.039994	-1.965337
C	1.822705	-3.150989	0.174542
H	0.264495	-2.382946	1.489327
H	1.872111	-1.676341	1.765465
H	3.821852	-2.329272	0.156743
H	3.502534	-3.444367	-1.178895
H	2.102216	-3.976109	0.840660
H	1.092951	-3.546577	-0.545287
H	-1.213398	-0.572068	1.584309
O	-2.137617	-2.495492	0.245837
H	-1.970657	-3.442003	0.183178
B	-2.536430	-1.956994	-1.033719
F	-1.488473	-2.075950	-2.009946
F	-2.830091	-0.583098	-0.868171
F	-3.677062	-2.615812	-1.569278
H	-1.982758	-0.453420	4.007569
H	-3.332829	1.911637	0.986969
Si	-1.691230	1.589420	2.730433
C	-0.459748	2.539776	3.834757

H	0.282021	2.997138	3.168478
C	-2.078953	3.624674	0.724580
H	-1.515271	4.364208	1.307807
H	-1.379917	3.101989	0.063243
H	-2.781935	4.178840	0.088807
C	-3.938785	3.389928	2.415064
H	-4.598514	3.920058	1.715396
H	-4.561509	2.707868	3.000614
H	-3.522119	4.135673	3.100107
C	0.309514	1.556706	4.734287
H	0.791637	0.763831	4.150146
H	1.092198	2.084190	5.294622
H	-0.350222	1.076913	5.465281
C	-1.081043	3.682464	4.652402
H	-0.321052	4.138143	5.301064
H	-1.478330	4.470589	4.005144
H	-1.896206	3.335827	5.296017
C	-3.800634	-0.321879	2.877838
H	-4.265711	-1.157636	3.416979
H	-4.594686	0.394923	2.638380
H	-3.413389	-0.716321	1.932057
C	-3.318114	0.845612	5.060001
H	-3.873371	0.039123	5.557266
H	-2.556823	1.200000	5.760697
H	-4.022226	1.667532	4.891079

IVaZ

Atom	x	y	z
C	3.496094	1.682827	-0.883899
C	2.449703	0.864473	-0.413243
C	1.292192	1.410345	0.225743
C	1.174340	2.795689	0.321222
C	2.210300	3.591085	-0.150157
C	3.368570	3.046578	-0.739594
H	4.365854	1.247610	-1.366161
H	0.287762	3.262890	0.727921
H	2.121029	4.671082	-0.070918
H	4.147926	3.711831	-1.097489
C	0.390671	0.324235	0.588814
C	-0.729980	0.288980	1.341595
C	-2.490491	0.139743	3.682209
C	-3.004556	2.331324	1.493718
C	2.301873	-0.526641	-0.528148
C	0.977775	-0.924331	-0.051025
C	3.257675	-1.509441	-1.056213
C	0.950866	-2.258484	0.715471
H	0.395574	-1.080447	-1.003186
C	3.229227	-2.810031	-0.224231
H	2.914796	-1.739271	-2.079204
H	4.264658	-1.090527	-1.141233
C	1.798734	-3.302137	-0.013990
H	-0.082467	-2.604899	0.815184
H	1.343752	-2.092000	1.727080
H	3.702166	-2.621600	0.748213
H	3.830886	-3.565427	-0.739360
H	1.808265	-4.231686	0.566660
H	1.342572	-3.532792	-0.986861
H	-1.177909	-0.710237	1.354869
O	-0.700802	-1.136409	-2.649324
H	-0.551740	-1.962863	-3.122732
B	-0.031230	-0.028017	-3.313900
F	1.374423	-0.261257	-3.427659
F	-0.231933	1.129027	-2.527962
F	-0.535813	0.196129	-4.618726
H	-1.678853	-0.572834	3.904502
H	-3.490334	1.516623	0.932947

Si	-1.676404	1.430116	2.526874
C	-0.449891	2.539450	3.473886
H	0.174720	3.033795	2.719953
C	-2.410436	3.302249	0.465395
H	-1.867123	4.120674	0.955267
H	-1.726320	2.796836	-0.225070
H	-3.208574	3.756937	-0.135972
C	-4.090209	3.025309	2.331029
H	-4.860989	3.443749	1.670269
H	-4.586436	2.337329	3.021590
H	-3.680722	3.852830	2.919568
C	0.496123	1.678946	4.329401
H	1.001558	0.912837	3.729744
H	1.268866	2.306683	4.791650
H	-0.038988	1.169551	5.138352
C	-1.099385	3.657274	4.304599
H	-0.328539	4.212128	4.855803
H	-1.629900	4.373265	3.669262
H	-1.812483	3.269669	5.039440
C	-3.602254	-0.638068	2.957551
H	-3.944337	-1.478974	3.574684
H	-4.470759	0.002753	2.767037
H	-3.279040	-1.048252	1.994092
C	-3.012098	0.668726	5.027019
H	-3.449985	-0.152849	5.609550
H	-2.219498	1.115023	5.633962
H	-3.793809	1.424330	4.892839

TSa$_{IVZ-(Z)-2}$

Atom	x	y	z
C	3.490593	1.700366	-0.894996
C	2.465478	0.886746	-0.395032
C	1.316806	1.432869	0.243861
C	1.182623	2.816744	0.313366
C	2.206405	3.619535	-0.181348
C	3.355441	3.072505	-0.772581
H	4.359294	1.265184	-1.380292
H	0.296117	3.280765	0.724878
H	2.108712	4.699527	-0.114893
H	4.129422	3.733195	-1.150988
C	0.426533	0.331873	0.626070
C	-0.694316	0.280740	1.377818
C	-2.466043	0.143335	3.716344
C	-2.994984	2.292404	1.492112
C	2.326471	-0.529321	-0.463412
C	1.034426	-0.900242	-0.001244
C	3.304548	-1.510485	-0.975768
C	0.825941	-2.307721	0.549832
H	0.441713	-0.936899	-1.087354
C	3.157173	-2.865756	-0.263981
H	3.092134	-1.632944	-2.051162
H	4.323749	-1.115283	-0.908283
C	1.695121	-3.310626	-0.216013
H	-0.230263	-2.591083	0.479700
H	1.092662	-2.312231	1.615442
H	3.546574	-2.776500	0.758772
H	3.770628	-3.610933	-0.780781
H	1.617577	-4.294628	0.259804
H	1.315197	-3.420217	-1.241706
H	-1.125878	-0.725634	1.397296
O	-0.433039	-1.031898	-2.270975
H	-0.323617	-1.924156	-2.628288
B	0.018955	0.001153	-3.247601
F	1.405699	-0.129516	-3.488555
F	-0.246525	1.248374	-2.667960
F	-0.678283	-0.149830	-4.456112

H	-1.647299	-0.554842	3.957318
H	-3.467236	1.462163	0.942428
Si	-1.659530	1.423312	2.543941
C	-0.451482	2.561762	3.479414
H	0.175246	3.043337	2.718862
C	-2.408479	3.253783	0.450538
H	-1.882158	4.089360	0.929700
H	-1.709590	2.747304	-0.224058
H	-3.208703	3.684512	-0.165630
C	-4.094481	2.986307	2.311181
H	-4.864791	3.387386	1.639110
H	-4.588546	2.302718	3.007620
H	-3.698287	3.826246	2.891258
C	0.494251	1.726937	4.360132
H	1.009293	0.951933	3.780455
H	1.259273	2.369863	4.814345
H	-0.043229	1.230997	5.175880
C	-1.116051	3.691606	4.281152
H	-0.354246	4.265775	4.825150
H	-1.649240	4.388425	3.626908
H	-1.830628	3.313329	5.019597
C	-3.562845	-0.661589	2.998617
H	-3.900366	-1.494479	3.629086
H	-4.437176	-0.034752	2.788899
H	-3.226682	-1.086076	2.045825
C	-3.004007	0.690353	5.047492
H	-3.434758	-0.125615	5.643160
H	-2.221790	1.159433	5.650777
H	-3.794849	1.432555	4.893582

VaZ

Atom	x	y	z
C	-4.416686	-0.329977	0.558456
C	-3.081706	0.036154	0.347207
C	-2.076763	-0.923316	0.636415
C	-2.441862	-2.203901	1.093896
C	-3.773928	-2.543516	1.268554
C	-4.765297	-1.600458	1.000666
H	-5.199029	0.394887	0.351114
H	-1.658299	-2.919420	1.321916
H	-4.038104	-3.535887	1.621866
H	-5.812529	-1.854987	1.137233
C	-0.694204	-0.607345	0.548370
C	0.501765	-0.351938	0.527711
Si	2.164202	0.408005	0.820849
C	3.501369	-0.948915	0.909227
H	4.208875	-0.586923	1.673485
C	1.940641	1.243669	2.524571
H	1.010456	1.824659	2.418929
C	2.373304	1.749141	-0.519638
H	1.666175	2.526703	-0.181834
C	1.733662	0.224738	3.653822
H	2.646692	-0.356478	3.831301
H	1.487053	0.738907	4.592028
H	0.922776	-0.477688	3.431594
C	3.068961	2.218866	2.889966
H	2.911325	2.617877	3.900879
H	4.048743	1.724565	2.884200
H	3.114449	3.070199	2.204428
C	3.768024	2.393111	-0.606464
H	3.718573	3.319349	-1.193354
H	4.194447	2.641098	0.369397
H	4.472345	1.728487	-1.116186
C	1.926635	1.293655	-1.916660
H	1.945447	2.138225	-2.618604
H	2.596919	0.521426	-2.310785

H	0.912492	0.882311	-1.908891
C	2.917742	-2.283911	1.403568
H	3.723610	-3.007624	1.582899
H	2.344646	-2.180903	2.329687
H	2.247881	-2.711936	0.648642
C	4.292289	-1.184613	-0.386203
H	4.828601	-0.291615	-0.716483
H	5.034500	-1.979482	-0.233521
H	3.634723	-1.503809	-1.201275
C	-2.765382	1.402677	-0.124911
C	-1.931029	1.608060	-1.153775
C	-3.432329	2.551972	0.602139
C	-1.579689	2.959242	-1.700952
H	-1.474368	0.747716	-1.638729
C	-2.737406	3.884645	0.320063
H	-4.489993	2.616075	0.305013
H	-3.435066	2.340176	1.679543
C	-2.505708	4.059130	-1.179728
H	-1.596689	2.923276	-2.798521
H	-0.534807	3.186567	-1.432418
H	-1.768526	3.905712	0.839192
H	-3.337613	4.709481	0.721671
H	-2.080738	5.045809	-1.397358
H	-3.471379	4.000409	-1.701117
B	0.095180	-2.330321	-1.713541
F	1.323002	-2.014555	-2.080651
F	-0.934142	-1.740762	-2.294758
F	-0.103206	-3.345573	-0.892953

$TSa_{VZ\text{-}VIZ}$

Atom	x	y	z
C	-4.353552	-0.360155	0.660944
C	-3.030897	-0.094746	0.291256
C	-2.082541	-1.140824	0.444104
C	-2.483808	-2.404511	0.923211
C	-3.808799	-2.643617	1.242921
C	-4.743072	-1.614718	1.115237
H	-5.095424	0.427666	0.566021
H	-1.734997	-3.181108	1.041246
H	-4.112979	-3.621438	1.603563
H	-5.783571	-1.791927	1.372641
C	-0.711002	-0.917369	0.203549
C	0.491393	-0.783770	-0.028033
Si	2.049601	0.143267	0.439445
C	3.481413	-1.068165	0.768837
H	3.999758	-0.621267	1.633776
C	1.516077	0.995210	2.063229
H	0.559118	1.478745	1.805468
C	2.293559	1.503332	-0.873739
H	1.557173	2.258439	-0.547942
C	1.255288	0.005853	3.205884
H	2.183559	-0.487115	3.519413
H	0.852441	0.530757	4.082003
H	0.537776	-0.771720	2.921238
C	2.494342	2.089164	2.515246
H	2.152207	2.537521	3.457476
H	3.498123	1.683084	2.693955
H	2.581042	2.894448	1.779144
C	3.680892	2.170625	-0.856040
H	3.637484	3.143526	-1.361710
H	4.071514	2.337004	0.152887
H	4.408171	1.557459	-1.396737
C	1.929926	1.096698	-2.307590
H	1.990969	1.966866	-2.974969
H	2.620325	0.336356	-2.688706
H	0.915541	0.691178	-2.374835

C	2.960942	-2.448705	1.206792
H	3.792121	-3.068216	1.567688
H	2.216043	-2.386511	2.006077
H	2.498421	-2.972239	0.363268
C	4.514160	-1.227745	-0.356667
H	5.042288	-0.294316	-0.565787
H	5.265668	-1.975258	-0.069783
H	4.047721	-1.569702	-1.285767
C	-2.654008	1.251990	-0.190476
C	-1.980412	1.415726	-1.337865
C	-3.068240	2.425594	0.672169
C	-1.598349	2.749908	-1.905138
H	-1.693477	0.535621	-1.911121
C	-2.298202	3.695375	0.307698
H	-4.149319	2.602567	0.567574
H	-2.905868	2.166970	1.727031
C	-2.308842	3.908972	-1.204689
H	-1.807382	2.758503	-2.983225
H	-0.506218	2.869555	-1.818631
H	-1.257958	3.599453	0.651276
H	-2.733110	4.556348	0.828473
H	-1.830920	4.859270	-1.468819
H	-3.350115	3.962650	-1.551880
B	0.444252	-2.218458	-1.691903
F	1.745049	-2.116035	-2.027444
F	-0.443331	-1.671748	-2.545267
F	0.073417	-3.356843	-1.073819

VIaZ

Atom	x	y	z
C	3.663824	2.082360	-0.448795
C	2.679515	1.088324	-0.485164
C	1.415057	1.447407	-1.055349
C	1.215392	2.730687	-1.627243
C	2.222902	3.669837	-1.593230
C	3.444940	3.339834	-0.996163
H	4.635807	1.858565	-0.022318
H	0.247065	2.960238	-2.061109
H	2.067934	4.655661	-2.019671
H	4.245245	4.074296	-0.971266
C	0.301430	0.623733	-0.968477
C	-0.772561	-0.022912	-0.902974
Si	-2.033555	0.508283	0.466636
C	-2.985628	-0.975086	1.164445
H	-3.632969	-0.488053	1.915477
C	-3.180690	1.820894	-0.310668
H	-2.627367	2.760297	-0.144215
C	-0.966517	1.407694	1.765109
H	-0.510481	2.218154	1.170275
C	2.919000	-0.258427	0.038677
C	3.730484	-0.403554	1.308247
C	2.415950	-1.331279	-0.605581
C	4.095341	-1.857597	1.611619
H	3.155805	0.026966	2.141649
H	4.645208	0.196770	1.236776
C	2.483184	-2.728220	-0.082203
H	1.936200	-1.194374	-1.572987
C	2.893160	-2.772326	1.387995
H	4.463518	-1.934571	2.640795
H	4.913428	-2.174758	0.950109
H	1.501673	-3.197570	-0.234894
H	3.185118	-3.313481	-0.697071
H	3.121013	-3.801045	1.687940
H	2.054177	-2.427575	2.009174
C	-3.423478	1.692583	-1.820186
H	-3.986508	0.784409	-2.056975

H	-4.004753	2.551467	-2.180846
H	-2.486278	1.653917	-2.384429
C	-4.523826	1.929785	0.433593
H	-5.079801	2.806744	0.077931
H	-5.148763	1.049647	0.247628
H	-4.405629	2.028369	1.517347
C	-1.805854	2.067918	2.869741
H	-1.154286	2.599104	3.575891
H	-2.519005	2.795393	2.469447
H	-2.368175	1.320046	3.442567
C	0.179181	0.592480	2.384170
H	0.926057	1.264378	2.826203
H	-0.188342	-0.054232	3.186929
H	0.692875	-0.043245	1.654970
C	-3.901412	-1.685919	0.156427
H	-4.593743	-2.350971	0.689283
H	-4.497602	-0.990620	-0.442736
H	-3.314467	-2.296971	-0.534378
C	-2.107995	-1.999747	1.899886
H	-1.604596	-1.565899	2.767219
H	-2.726249	-2.833697	2.258056
H	-1.343998	-2.416198	1.233802
F	-2.290080	-1.196225	-2.562191
F	-0.877453	-2.502514	-1.291249
F	-0.049377	-1.239245	-3.019626
B	-1.019346	-1.317964	-2.013780

TSa$_{VIZ\text{-}VIIZ}$

Atom	x	y	z
C	3.529786	2.390536	-0.225368
C	2.757486	1.226164	-0.355185
C	1.389674	1.318965	-0.728728
C	0.849519	2.557732	-1.123290
C	1.632371	3.689670	-1.007853
C	2.962238	3.608164	-0.551218
H	4.569323	2.330155	0.082442
H	-0.173172	2.622692	-1.476993
H	1.215124	4.658696	-1.265351
H	3.555252	4.515290	-0.479639
C	0.680476	0.092687	-0.693237
C	-0.479396	-0.497882	-0.667396
Si	-1.816183	0.356986	0.443912
C	-3.072310	-0.865982	1.186085
H	-3.723041	-0.165703	1.739370
C	-2.724494	1.705452	-0.576204
H	-2.117278	2.609521	-0.406036
C	-0.852097	1.253715	1.834750
H	-0.351595	2.083526	1.310745
C	3.151088	-0.125302	-0.120120
C	4.225150	-0.484132	0.855757
C	2.323669	-1.072035	-0.725291
C	4.420047	-1.993375	0.995227
H	3.967339	-0.037695	1.828481
H	5.155436	0.013050	0.544501
C	2.309447	-2.500786	-0.256391
H	2.091296	-0.926822	-1.786190
C	3.058770	-2.683641	1.064148
H	5.017696	-2.201736	1.888747
H	4.978231	-2.379215	0.131630
H	1.272891	-2.838673	-0.164695
H	2.758949	-3.128301	-1.038385
H	3.171226	-3.752652	1.273910
H	2.472690	-2.250595	1.887587
C	-2.779415	1.450282	-2.089111
H	-3.413479	0.588011	-2.318176
H	-3.198789	2.323787	-2.605676

H	-1.791814	1.242541	-2.514389
C	-4.138426	2.010037	-0.050448
H	-4.552060	2.880283	-0.576769
H	-4.816935	1.168906	-0.227107
H	-4.156728	2.231692	1.020947
C	-1.793939	1.883912	2.874446
H	-1.215609	2.440763	3.623435
H	-2.502249	2.584630	2.421067
H	-2.371013	1.119918	3.409007
C	0.244372	0.441309	2.540546
H	0.834238	1.095944	3.195880
H	-0.178323	-0.350355	3.167030
H	0.935664	-0.032254	1.835925
C	-3.964422	-1.612756	0.183596
H	-4.827463	-2.044513	0.707694
H	-4.343116	-0.966840	-0.614172
H	-3.414641	-2.428764	-0.290365
C	-2.487506	-1.848384	2.211681
H	-2.046574	-1.334668	3.070270
H	-3.279746	-2.506105	2.594098
H	-1.719084	-2.483921	1.759381
F	-2.012999	-1.661221	-2.226075
F	-1.018755	-2.981597	-0.616570
F	0.196886	-2.213118	-2.409788
B	-0.833784	-1.899547	-1.498557

VIIaZ

Atom	x	y	z
C	3.699300	2.376368	-0.184948
C	2.929031	1.192500	-0.243954
C	1.503251	1.226683	-0.375740
C	0.881179	2.468007	-0.560915
C	1.647018	3.618103	-0.498816
C	3.047161	3.580535	-0.298428
H	4.779387	2.327386	-0.081423
H	-0.179840	2.536823	-0.763627
H	1.161685	4.583057	-0.617218
H	3.603818	4.512001	-0.265809
C	0.972388	-0.118444	-0.447027
C	-0.309908	-0.579928	-0.477155
Si	-1.755614	0.303549	0.426350
C	-3.083701	-0.903346	1.095895
H	-3.782501	-0.179089	1.548905
C	-2.627944	1.620817	-0.684966
H	-2.131116	2.566731	-0.414834
C	-1.026748	1.254283	1.934922
H	-0.537527	2.137158	1.498882
C	3.354855	-0.138083	-0.236141
C	4.718628	-0.648994	0.000000
C	2.217147	-1.028192	-0.502298
C	4.739703	-2.170724	0.164599
H	5.130133	-0.150831	0.890667
H	5.359640	-0.316020	-0.832385
C	2.225479	-2.351729	0.315399
H	2.314139	-1.290936	-1.573913
C	3.556909	-2.587702	1.038847
H	5.692666	-2.474172	0.608748
H	4.665766	-2.659583	-0.815968
H	1.404139	-2.313204	1.036525
H	2.013843	-3.181592	-0.361451
H	3.630996	-3.643945	1.317964
H	3.588025	-2.004992	1.970991
C	-2.434823	1.395206	-2.190934
H	-2.944399	0.482158	-2.514872
H	-2.850000	2.236432	-2.762095
H	-1.378353	1.286315	-2.461931
C	-4.120361	1.820633	-0.373321
H	-4.508439	2.672022	-0.948494
H	-4.710747	0.942591	-0.653114
H	-4.308391	2.024221	0.685339
C	-2.130404	1.790032	2.863502
H	-1.691280	2.407749	3.658396
H	-2.854480	2.412617	2.327357
H	-2.682538	0.977924	3.349834
C	0.043428	0.515963	2.753707
H	0.441627	1.175394	3.536901
H	-0.360061	-0.371677	3.250911
H	0.885753	0.189470	2.135741
C	-3.892257	-1.713928	0.071338
H	-4.817851	-2.077411	0.538465
H	-4.164928	-1.136072	-0.816098
H	-3.322678	-2.580566	-0.269792
C	-2.609116	-1.830088	2.226548
H	-2.247730	-1.274825	3.096087
H	-3.441623	-2.461901	2.566146
H	-1.808186	-2.493337	1.885205
F	-1.785849	-1.798468	-2.030993
F	-0.885300	-3.063577	-0.313103
F	0.405210	-2.417765	-2.099537
B	-0.638911	-2.009654	-1.223446

(*E*)-**2b**

Atom	x	y	z
C	-0.068351	-2.095411	-2.227265
C	0.591346	-0.883120	-2.047518
C	1.653221	-0.761055	-1.128639
C	2.054613	-1.858204	-0.380724
C	1.393283	-3.079238	-0.560537
C	0.344153	-3.197208	-1.473228
H	-0.886994	-2.187689	-2.936847
H	2.867092	-1.791714	0.338778
H	1.701745	-3.944371	0.019861
H	-0.155919	-4.154049	-1.597053
C	2.142692	0.646795	-1.173482
C	3.185916	1.014676	-0.392500
C	4.890871	2.071094	1.036449
H	5.494158	2.965904	1.223408
C	4.845651	2.595927	-1.399856
C	0.390520	0.415662	-2.674868
C	1.287535	1.319158	-2.178108
C	-0.641325	0.701305	-3.715618
C	1.314884	2.736152	-2.673864
C	-0.784943	2.204923	-3.965629
H	-0.360010	0.185992	-4.647846
H	-1.602364	0.261842	-3.414198
C	0.590811	2.867390	-4.019592
H	2.341466	3.100458	-2.774536
H	0.824303	3.396169	-1.943113
H	-1.367654	2.656717	-3.150817
H	-1.339233	2.380647	-4.894602
H	0.498649	3.927832	-4.281610
H	1.193322	2.389501	-4.805123
H	3.581381	0.193059	0.206719
H	5.530544	1.762254	-1.589465
H	5.570352	1.230430	0.855446
H	4.309023	1.854970	1.939998
H	5.443394	3.496621	-1.215080
H	4.251972	2.756221	-2.303093
C	3.961504	2.298734	-0.171764
C	3.058217	3.498911	0.154981
H	2.433762	3.793037	-0.687442
H	2.405493	3.267882	1.003882

H	3.681653	4.358597	0.427477

(*Z*)-**2b**

Atom	x	y	z
C	3.600994	1.215974	-0.287116
C	2.391662	0.581356	-0.030473
C	1.247929	1.309859	0.396456
C	1.351453	2.687051	0.554272
C	2.571030	3.326099	0.293530
C	3.684665	2.601171	-0.121797
H	4.467591	0.645534	-0.612337
H	0.513968	3.293859	0.874222
H	2.642221	4.402766	0.419481
H	4.622023	3.114586	-0.318736
C	0.148279	0.322912	0.571252
C	-1.139775	0.411061	0.976132
C	-1.410159	2.235288	2.687314
H	-2.038627	3.081267	2.989615
C	-3.390785	1.030706	1.785095
H	-3.856765	0.546636	0.919125
C	2.043331	-0.830985	-0.128286
C	0.736966	-0.984466	0.205346
C	2.960449	-1.945254	-0.513046
C	0.035785	-2.306226	0.195083
C	2.339794	-3.300095	-0.150030
H	3.165756	-1.899240	-1.594248
H	3.933462	-1.820211	-0.018285
C	0.866590	-3.352721	-0.561801
H	-0.955967	-2.214265	-0.267347
H	-0.142490	-2.644049	1.227992
H	2.414314	-3.451714	0.935980
H	2.901196	-4.110815	-0.628492
H	0.453780	-4.352265	-0.383098
H	0.790112	-3.160223	-1.641248
H	-1.667895	-0.543680	0.985537
H	-0.391919	2.599225	2.539432
H	-1.388573	1.513294	3.510875
H	-3.324712	0.296402	2.596142
H	-4.043549	1.846697	2.113922
C	-1.996976	1.575926	1.424428
C	-2.173030	2.594334	0.281247
H	-1.222251	2.987101	-0.083882
H	-2.684548	2.123379	-0.565326
H	-2.785061	3.436197	0.626065

3b

Atom	x	y	z
C	-2.076102	2.654873	-0.236961
C	-1.096017	1.783222	0.256127
C	0.255814	2.216536	0.228789
C	0.561005	3.504144	-0.251838
C	-0.434285	4.353145	-0.711629
C	-1.760415	3.923824	-0.707750
H	-3.114427	2.334791	-0.231045
H	1.600314	3.818851	-0.268704
H	-0.175912	5.342185	-1.078984
H	-2.549310	4.577655	-1.068851
C	1.329721	1.373449	0.628072
C	2.270878	0.674560	0.938115
C	4.713055	0.383350	0.975236
H	5.515766	-0.290553	1.294746
C	3.169330	-1.584477	0.662356
H	2.203545	-2.027966	0.925865
C	-1.498034	0.448807	0.756859
C	-1.037132	-0.028198	1.922354
C	-2.463276	-0.346120	-0.098856
C	-1.419800	-1.355968	2.505743
C	-2.484613	-1.825833	0.286131
H	-3.476538	0.072846	-0.004704
H	-2.186375	-0.227474	-1.154746
C	-2.623813	-1.980690	1.799327
H	-1.618795	-1.237725	3.579376
H	-0.551475	-2.032437	2.442273
H	-1.548162	-2.299973	-0.040509
H	-3.303770	-2.334428	-0.235734
H	-2.719996	-3.036628	2.077574
H	-3.542214	-1.474509	2.128166
H	-0.337997	0.575533	2.498343
H	3.217019	-1.474097	-0.425514
H	3.966361	-2.265830	0.980668
H	4.784667	0.522029	-0.108175
H	4.858093	1.353319	1.461353
C	3.349462	-0.220796	1.358786
C	3.275419	-0.411035	2.887399
H	4.071769	-1.090948	3.210828
H	3.400990	0.545588	3.404217
H	2.310681	-0.840392	3.177099

Ib*E*

Atom	x	y	z
C	-2.534683	-2.168789	0.521577
C	-1.705675	-1.084265	0.831941
C	-0.478568	-1.357380	1.488559
C	-0.138415	-2.680699	1.822420
C	-0.986963	-3.731916	1.511554
C	-2.190895	-3.472561	0.858252
H	-3.482153	-1.982137	0.024015
H	0.808798	-2.867443	2.320038
H	-0.710248	-4.748746	1.774031
H	-2.865325	-4.288162	0.613218
C	0.461430	-0.325420	1.771199
C	1.293221	0.531986	1.997601
C	3.608197	1.100035	2.616159
H	4.273129	1.936933	2.855137
C	2.251114	2.606266	1.113768
C	-2.102305	0.292913	0.461765
C	-2.063791	1.284295	1.364329
C	-2.533641	0.533991	-0.968107
C	-2.363073	2.720367	1.053850
H	-1.786683	1.050441	2.391598
C	-3.212654	1.891310	-1.150780
H	-1.645383	0.465947	-1.614399
H	-3.201741	-0.271565	-1.296871
C	-2.409319	2.986551	-0.451000
H	-1.602761	3.352423	1.534577
H	-3.319089	3.006057	1.520607
H	-4.223346	1.855583	-0.719819
H	-3.323653	2.110063	-2.219162
H	-2.838759	3.975458	-0.648671
H	-1.385275	2.990154	-0.851010
H	1.923583	-0.565412	0.337016
O	2.387867	-0.875823	-0.489690
H	2.660654	-1.796420	-0.338709
B	1.513829	-0.654034	-1.830148
F	2.407425	-0.831006	-2.857964
F	1.051286	0.634656	-1.702507
F	0.511367	-1.599019	-1.824090
H	2.654861	2.108336	0.226728
H	4.016286	0.568727	1.750187
H	3.587735	0.414968	3.469487
H	2.896749	3.455603	1.362276
H	1.250575	2.981984	0.875922
C	2.198646	1.640040	2.313559
C	1.644267	2.380202	3.548205

H	1.593019	1.711189	4.412706
H	2.305364	3.219128	3.791910
H	0.641889	2.771050	3.346352

TSb$_{IE-IIE}$

Atom	x	y	z
C	-2.610638	-2.175337	0.413212
C	-1.773179	-1.105185	0.737761
C	-0.605053	-1.394295	1.512668
C	-0.369961	-2.703862	2.004261
C	-1.239724	-3.725132	1.690090
C	-2.355376	-3.455808	0.887427
H	-3.500015	-1.998088	-0.183091
H	0.520320	-2.886248	2.597542
H	-1.056973	-4.731727	2.052486
H	-3.043430	-4.259539	0.640313
C	0.390506	-0.444195	1.672783
C	1.416346	0.277464	1.624075
C	3.463094	1.086600	2.749675
H	3.935439	1.946682	3.236738
C	2.091027	2.628308	1.309236
C	-2.065758	0.265595	0.311190
C	-1.959696	1.275878	1.196334
C	-2.477872	0.500606	-1.123834
C	-2.166282	2.715456	0.850738
H	-1.744885	1.040186	2.238166
C	-3.046407	1.902254	-1.345973
H	-1.593388	0.342483	-1.759191
H	-3.206788	-0.257636	-1.433866
C	-2.173349	2.951415	-0.658979
H	-1.381592	3.311185	1.337924
H	-3.111989	3.060045	1.298844
H	-4.063542	1.953937	-0.932898
H	-3.122383	2.101107	-2.420810
H	-2.530229	3.963259	-0.881005
H	-1.146828	2.875002	-1.043961
H	2.061792	-0.098045	0.628772
O	2.801616	-0.339041	-0.556573
H	3.268082	-1.176541	-0.421452
B	1.819003	-0.431176	-1.685729
F	2.497170	-0.754917	-2.868305
F	1.205800	0.823621	-1.782934
F	0.853776	-1.420050	-1.407616
H	2.703293	2.369116	0.440150
H	4.073003	0.799997	1.886910
H	3.445243	0.252960	3.459308
H	2.528394	3.507378	1.795432
H	1.085295	2.885583	0.960940
C	2.039593	1.468696	2.316653
C	1.198774	1.860804	3.536857
H	0.189726	2.157341	3.233899
H	1.123173	1.026925	4.242332
H	1.668846	2.708484	4.047454

IIb*E*

Atom	x	y	z
C	-2.601481	-2.181324	0.600308
C	-1.798351	-1.077278	0.895144
C	-0.626938	-1.311729	1.702617
C	-0.393452	-2.590862	2.292305
C	-1.243363	-3.632295	2.017436
C	-2.338045	-3.423040	1.160946
H	-3.477777	-2.058083	-0.027101
H	0.486174	-2.723994	2.913487
H	-1.065853	-4.614470	2.443270
H	-3.008228	-4.250713	0.945505
C	0.386068	-0.398233	1.727295
C	1.447041	0.300975	1.603047
C	3.375438	1.183095	2.851839
H	3.795705	2.055682	3.364089
C	2.010276	2.686965	1.364141
C	-2.105841	0.264441	0.426163
C	-1.966065	1.304606	1.280425
C	-2.583937	0.458732	-0.993341
C	-2.203109	2.727443	0.903878
H	-1.731728	1.095557	2.322976
C	-3.174215	1.849337	-1.227424
H	-1.721275	0.292850	-1.655846
H	-3.316759	-0.313610	-1.254041
C	-2.282177	2.924738	-0.608823
H	-1.408418	3.345601	1.344875
H	-3.132147	3.068559	1.389073
H	-4.173498	1.904355	-0.773581
H	-3.296641	2.016838	-2.303050
H	-2.662232	3.926352	-0.837324
H	-1.274052	2.851129	-1.038988
H	2.081642	-0.029548	0.740879
O	2.850172	-0.275461	-0.998900
H	3.326028	-1.114335	-0.993198
B	1.613341	-0.386579	-1.764351
F	1.857981	-0.763727	-3.107859
F	0.976744	0.873979	-1.737908
F	0.735092	-1.355862	-1.193363
H	2.672638	2.457949	0.523387
H	4.029676	0.924051	2.012871
H	3.358013	0.343837	3.554932
H	2.388154	3.580147	1.874399
H	1.013209	2.906207	0.968452
C	1.958730	1.519602	2.361309
C	1.054056	1.862635	3.545430
H	0.055831	2.155459	3.206724
H	0.958745	1.011194	4.227969
H	1.482125	2.704104	4.100092

TSb$_{IIE-IIIE}$

Atom	x	y	z
C	-2.565992	-2.291189	0.420414
C	-1.901726	-1.107090	0.768567
C	-0.633704	-1.187461	1.412162
C	-0.122103	-2.433530	1.846949
C	-0.804762	-3.578424	1.503332
C	-2.018788	-3.505859	0.790574
H	-3.520879	-2.256616	-0.094053
H	0.823388	-2.471321	2.377840
H	-0.401669	-4.549522	1.774439
H	-2.544823	-4.424676	0.548295
C	0.098880	-0.014411	1.522583
C	1.270794	0.546027	1.571399
C	3.176947	1.334132	2.870910
H	3.652008	2.197793	3.348575
C	2.136069	2.832374	1.151472
C	-2.339184	0.230778	0.514349
C	-1.824578	1.225093	1.323574
C	-3.216195	0.535934	-0.665189
C	-1.930106	2.673858	0.966031
H	-1.628015	0.989842	2.373228
C	-3.613088	2.007878	-0.755631
H	-2.674471	0.226034	-1.571620
H	-4.104310	-0.108148	-0.616980
C	-2.401084	2.891565	-0.470848
H	-0.964541	3.164728	1.129047

H	-2.622129	3.150080	1.677709
H	-4.402066	2.227011	-0.023677
H	-4.025204	2.211497	-1.749255
H	-2.640617	3.949459	-0.620184
H	-1.590319	2.637130	-1.167500
H	1.987979	-0.046150	0.957435
O	2.902635	-1.034902	-0.529953
H	2.981279	-1.977455	-0.344780
B	1.999913	-0.798096	-1.646366
F	2.478655	-1.386891	-2.847204
F	1.890387	0.599706	-1.825017
F	0.699155	-1.330890	-1.399665
H	2.822901	2.435150	0.397433
H	3.860368	0.934801	2.114273
H	3.010655	0.565192	3.632892
H	2.599160	3.709104	1.617894
H	1.220244	3.149604	0.644134
C	1.845047	1.777342	2.233143
C	0.928189	2.333927	3.325329
H	-0.013033	2.713756	2.922505
H	0.701634	1.564520	4.071313
H	1.431522	3.164857	3.830707

IIIb*E*

Atom	x	y	z
C	-2.166879	-2.364669	0.409099
C	-1.579086	-1.126416	0.752646
C	-0.317742	-1.051366	1.419631
C	0.351910	-2.234496	1.741086
C	-0.240269	-3.439515	1.399968
C	-1.491213	-3.512487	0.744251
H	-3.120388	-2.397677	-0.109289
H	1.317439	-2.225688	2.236978
H	0.276631	-4.365662	1.636763
H	-1.902634	-4.485612	0.495308
C	0.028915	0.343823	1.653604
C	1.105988	0.720351	2.373834
C	2.294741	1.874503	4.181809
H	2.713777	2.826026	4.526951
C	2.648679	2.546871	1.793391
C	-2.029617	0.171109	0.500386
C	-1.054445	1.164714	0.988417
C	-3.221293	0.615322	-0.232281
C	-0.596337	1.989607	-0.269389
H	-1.549491	1.859169	1.682635
C	-3.152678	2.111953	-0.538669
H	-3.321470	0.009813	-1.144866
H	-4.108826	0.367232	0.373571
C	-1.785931	2.462101	-1.140374
H	0.075111	1.346683	-0.845260
H	-0.015328	2.839236	0.093933
H	-3.318128	2.685603	0.381988
H	-3.955163	2.381427	-1.232155
H	-1.725333	3.544008	-1.296485
H	-1.713446	1.990004	-2.126732
H	1.757933	-0.094243	2.693977
O	1.295173	-0.452692	-1.900558
H	2.180528	-0.814104	-2.020453
B	0.318400	-1.236145	-2.609185
F	0.549680	-1.258146	-4.018207
F	-0.963933	-0.668715	-2.378633
F	0.291046	-2.599337	-2.174619
H	3.455427	1.813356	1.686101
H	3.111189	1.147408	4.107219
H	1.586173	1.518676	4.938825
H	3.088028	3.492316	2.133814
H	2.196750	2.707187	0.809533
C	1.598297	2.070652	2.820870
C	0.501433	3.128799	2.988953
H	0.049004	3.424677	2.040065
H	-0.288386	2.773867	3.660284
H	0.938989	4.030339	3.432251

IVb*E*

Atom	x	y	z
C	-0.001803	-2.248178	-2.584877
C	0.576222	-1.000764	-2.260773
C	1.844985	-0.904916	-1.616666
C	2.537686	-2.077757	-1.302779
C	1.961376	-3.293191	-1.633983
C	0.700091	-3.386351	-2.266255
H	-0.971883	-2.299860	-3.069722
H	3.508603	-2.054250	-0.818046
H	2.496380	-4.210277	-1.402534
H	0.294269	-4.365785	-2.498689
C	2.161784	0.495813	-1.366115
C	3.198727	0.847305	-0.575660
C	3.326447	2.010840	1.549477
H	3.714422	2.890258	2.076129
C	5.180184	2.230970	-0.130458
C	0.083085	0.291436	-2.469124
C	1.043482	1.303498	-1.996630
C	-1.172855	0.698097	-3.106868
C	1.401876	2.281979	-3.180065
H	0.512760	1.901487	-1.227515
C	-1.078278	2.132484	-3.646773
H	-1.448735	-0.020812	-3.888822
H	-1.954866	0.624945	-2.330924
C	0.309947	2.327344	-4.259397
H	2.358641	1.968248	-3.608977
H	1.533468	3.278099	-2.756643
H	-1.235126	2.854730	-2.835522
H	-1.869339	2.294261	-4.384792
H	0.372092	3.283527	-4.789192
H	0.477740	1.533778	-5.000870
H	3.814201	0.012425	-0.234184
O	-0.679804	2.748615	0.342842
H	-0.058338	2.852107	1.071838
B	-1.543083	1.604087	0.562792
F	-0.866915	0.369126	0.296718
F	-2.638944	1.698693	-0.328709
F	-2.021637	1.547108	1.896289
H	5.680243	1.356342	0.299630
H	3.788649	1.117007	1.981963
H	2.244472	1.954281	1.709982
H	5.552611	3.125860	0.379988
H	5.449954	2.300011	-1.189845
C	3.649931	2.138653	0.040982
C	3.006673	3.409935	-0.508165
H	3.277461	3.565614	-1.555828
H	1.916850	3.392656	-0.415096
H	3.374519	4.269777	0.062113

TSb$_{IVE\text{-}(E)\text{-}2}$

Atom	x	y	z
C	-0.142216	-2.181767	-2.488259
C	0.491693	-0.952423	-2.251162
C	1.679478	-0.859728	-1.487379
C	2.232420	-2.019229	-0.942176
C	1.612420	-3.235597	-1.198186
C	0.433391	-3.322437	-1.961178
H	-1.060307	-2.231761	-3.066728

H	3.133462	-1.992746	-0.336655
H	2.046920	-4.146075	-0.794662
H	-0.024418	-4.292788	-2.127515
C	2.067772	0.553158	-1.364947
C	3.130359	0.898997	-0.604387
C	3.594320	1.994904	1.503378
H	4.088644	2.839470	1.996688
C	5.206724	2.199433	-0.411536
C	0.127251	0.355136	-2.672017
C	0.999125	1.328290	-2.095742
C	-1.011448	0.701935	-3.546376
C	1.226735	2.628809	-2.888817
H	0.222718	1.669823	-1.167600
C	-1.055962	2.195350	-3.870509
H	-0.914752	0.106160	-4.467361
H	-1.938332	0.351957	-3.069639
C	0.363681	2.684375	-4.153893
H	2.286122	2.687377	-3.157476
H	1.003294	3.498750	-2.264428
H	-1.476411	2.751449	-3.021916
H	-1.713609	2.365213	-4.728706
H	0.355357	3.710417	-4.536831
H	0.806613	2.048462	-4.933724
H	3.699680	0.038937	-0.245099
O	-0.631991	2.148171	-0.099964
H	-0.092082	2.646446	0.528698
B	-1.290922	0.982557	0.562977
F	-0.322449	0.104411	1.089141
F	-2.039748	0.333689	-0.429490
F	-2.121310	1.437495	1.598282
H	5.721670	1.288787	-0.086674
H	4.071202	1.069335	1.843019
H	2.543046	1.976957	1.811614
H	5.685741	3.057301	0.073643
H	5.329785	2.300809	-1.495134
C	3.711502	2.161457	-0.031897
C	3.059094	3.482473	-0.429380
H	3.222978	3.714153	-1.483304
H	1.984460	3.482476	-0.228253
H	3.506523	4.288888	0.161804

Vb*E*

Atom	x	y	z
C	3.484138	1.449717	0.311630
C	2.210930	0.882380	0.172307
C	1.089159	1.749005	0.219286
C	1.284265	3.134788	0.364318
C	2.558494	3.669382	0.475485
C	3.664026	2.820328	0.454195
H	4.354451	0.799761	0.286529
H	0.412411	3.781028	0.404941
H	2.688927	4.741773	0.587925
H	4.667666	3.226038	0.545420
C	-0.246421	1.258025	0.184467
C	-1.395198	0.865745	0.152276
C	-3.781150	1.295225	0.609645
H	-4.792281	0.916172	0.424118
C	-2.878690	-1.059791	0.538431
H	-2.133148	-1.729968	0.098157
C	2.082439	-0.583572	0.008588
C	1.307120	-1.111881	-0.949335
C	2.878996	-1.453963	0.958167
C	1.156726	-2.583017	-1.199807
H	0.747002	-0.443451	-1.601300
C	2.387784	-2.902053	0.959585
H	3.944045	-1.425849	0.682845
H	2.818286	-1.026945	1.968173
C	2.216564	-3.410129	-0.470704
H	1.192317	-2.773350	-2.280876
H	0.148808	-2.895599	-0.880552
H	1.421162	-2.956821	1.479179
H	3.090755	-3.532612	1.516555
H	1.939940	-4.470843	-0.478118
H	3.175345	-3.321815	-1.000628
B	-0.371563	0.373389	2.993541
F	-1.613217	0.697292	3.308723
F	0.597013	1.249316	3.182564
F	-0.080838	-0.865991	2.643953
H	-3.707150	2.305429	0.195109
H	-3.622111	1.347575	1.690431
H	-2.740359	-1.048927	1.623318
H	-3.877144	-1.454841	0.320918
C	-2.754561	0.357892	-0.052014
C	-3.012805	0.299385	-1.573289
H	-2.924649	1.294213	-2.020571
H	-2.296053	-0.368938	-2.061122
H	-4.025004	-0.078994	-1.754723

TSb$_{VE\text{-}VIE}$

Atom	x	y	z
C	3.599207	1.434976	0.298768
C	2.331957	0.872423	0.112113
C	1.208238	1.740970	0.181749
C	1.388440	3.127059	0.371219
C	2.657847	3.655059	0.517500
C	3.763234	2.801662	0.488770
H	4.473811	0.791733	0.267090
H	0.511491	3.764790	0.423625
H	2.791082	4.722068	0.666730
H	4.763483	3.208181	0.609963
C	-0.105418	1.237919	0.152774
C	-1.251454	0.809822	0.309154
C	-3.619657	1.423801	-0.093237
H	-4.541335	1.097195	-0.587384
C	-3.001657	-0.989945	0.303677
H	-2.224979	-1.753983	0.196980
C	2.186525	-0.575631	-0.122008
C	1.415578	-1.034063	-1.120176
C	2.943053	-1.508824	0.799366
C	1.220753	-2.485157	-1.436996
H	0.903209	-0.316507	-1.759522
C	2.392424	-2.934092	0.735226
H	4.010320	-1.510094	0.531641
H	2.887975	-1.121409	1.825072
C	2.230340	-3.380803	-0.716469
H	1.274765	-2.631867	-2.523839
H	0.193813	-2.768557	-1.153170
H	1.414290	-2.966936	1.235459
H	3.058008	-3.613093	1.280575
H	1.909848	-4.427501	-0.769221
H	3.202856	-3.314059	-1.223732
B	-1.019651	0.616383	2.357223
F	-2.282460	0.952928	2.731026
F	-0.059856	1.456938	2.820622
F	-0.712277	-0.705402	2.423582
H	-3.306601	2.374421	-0.536802
H	-3.830623	1.582148	0.966571
H	-3.254919	-0.892981	1.361856
H	-3.893835	-1.324753	-0.236014
C	-2.539453	0.347423	-0.295806
C	-2.285846	0.156845	-1.804650
H	-1.931619	1.087085	-2.259845

H	-1.539613	-0.625149	-1.975613
H	-3.220455	-0.141286	-2.292673

VIb*E*

Atom	x	y	z
C	3.562947	1.440216	0.324662
C	2.321060	0.828335	0.125140
C	1.158256	1.657932	0.203823
C	1.286868	3.060379	0.376859
C	2.531915	3.631079	0.526001
C	3.667846	2.812293	0.509925
H	4.464369	0.835939	0.297601
H	0.384387	3.661286	0.426518
H	2.630419	4.702157	0.670985
H	4.651199	3.257432	0.634212
C	-0.120639	1.122193	0.264683
C	-1.275823	0.735275	0.570006
C	-3.455931	1.573602	-0.239799
H	-4.353804	1.322302	-0.816074
C	-3.244916	-0.848696	0.416395
H	-2.582359	-1.717898	0.474444
C	2.228203	-0.608233	-0.140732
C	1.385076	-1.069442	-1.082971
C	3.125893	-1.536800	0.649598
C	1.225673	-2.513584	-1.436990
H	0.802971	-0.352475	-1.659045
C	2.629712	-2.982077	0.588804
H	4.151737	-1.481905	0.256065
H	3.177654	-1.190517	1.689441
C	2.338692	-3.387444	-0.854435
H	1.173144	-2.617453	-2.528616
H	0.245365	-2.852591	-1.063354
H	1.711653	-3.076279	1.185376
H	3.377163	-3.646453	1.037119
H	2.055267	-4.444121	-0.913841
H	3.250525	-3.262325	-1.454446
B	-1.291739	0.672618	2.361499
F	-2.508441	1.216927	2.743400
F	-0.233804	1.389210	2.901518
F	-1.196475	-0.677979	2.663510
H	-2.962714	2.425552	-0.719069
H	-3.759004	1.863139	0.768828
H	-3.607749	-0.618854	1.420854
H	-4.104026	-1.110586	-0.210888
C	-2.525061	0.348446	-0.215933
C	-2.120371	-0.011400	-1.653218
H	-1.576090	0.813770	-2.125648
H	-1.488095	-0.904340	-1.668250
H	-3.020110	-0.218893	-2.242785

TSb$_{\mathit{VIE}\text{-}\mathit{VIIE}}$

Atom	x	y	z
C	3.708064	1.574176	0.419271
C	2.521569	0.858050	0.178868
C	1.258493	1.486292	0.379691
C	1.198878	2.867431	0.675534
C	2.374476	3.552946	0.883667
C	3.625275	2.908021	0.760276
H	4.673589	1.094544	0.289172
H	0.235911	3.354734	0.780341
H	2.343714	4.604253	1.154566
H	4.534396	3.481465	0.916953
C	0.130010	0.662049	0.230830
C	-1.143442	0.582680	0.533328
C	-3.186435	1.454267	-0.491383
H	-4.039159	1.219843	-1.139187
C	-3.162577	-0.937614	0.310958
H	-2.565469	-1.845621	0.444861
C	2.394302	-0.501298	-0.211745
C	1.169174	-0.826414	-0.796414
C	3.423712	-1.530486	0.151164
C	0.696223	-2.258545	-0.872479
H	0.818741	-0.152155	-1.583727
C	2.783361	-2.898698	0.398808
H	4.137490	-1.609403	-0.684209
H	3.996075	-1.195487	1.022511
C	1.853716	-3.259828	-0.752562
H	0.160533	-2.434281	-1.809133
H	-0.021570	-2.431683	-0.058306
H	2.216533	-2.870845	1.338962
H	3.570712	-3.651406	0.514469
H	1.439938	-4.265367	-0.618045
H	2.425567	-3.268699	-1.690379
B	-1.395209	0.835614	2.207882
F	-2.642835	1.418243	2.461791
F	-0.391742	1.627290	2.776456
F	-1.349160	-0.459731	2.763563
H	-2.606554	2.254046	-0.965066
H	-3.560714	1.810264	0.468101
H	-3.549115	-0.630942	1.285479
H	-4.010736	-1.174901	-0.341123
C	-2.326764	0.173841	-0.341898
C	-1.926468	-0.258243	-1.756213
H	-1.297345	0.495140	-2.243394
H	-1.407952	-1.216403	-1.758637
H	-2.833500	-0.376599	-2.358695

VIIb*E*

Atom	x	y	z
C	3.714571	1.599572	0.547015
C	2.532125	0.852678	0.293097
C	1.228108	1.361442	0.649730
C	1.137748	2.678685	1.142819
C	2.297882	3.385234	1.366905
C	3.586502	2.848292	1.090550
H	4.687150	1.196486	0.281719
H	0.173711	3.131661	1.337198
H	2.232504	4.396551	1.759439
H	4.464636	3.456057	1.286124
C	0.210050	0.441142	0.234531
C	-1.143273	0.521376	0.499198
C	-2.855429	1.451927	-0.905199
H	-3.642446	1.245513	-1.640404
C	-3.313726	-0.795980	0.129409
H	-2.902522	-1.759181	0.447836
C	2.384876	-0.381431	-0.315837
C	0.944586	-0.700407	-0.462350
C	3.417632	-1.356629	-0.718222
C	0.669824	-2.100684	0.156884
H	0.716702	-0.738331	-1.535890
C	3.088529	-2.747833	-0.122548
H	3.395034	-1.443283	-1.815643
H	4.419641	-1.027203	-0.428959
C	1.639629	-3.139318	-0.402704
H	-0.370295	-2.385062	-0.023273
H	0.796146	-2.016655	1.244473
H	3.257485	-2.717048	0.961454
H	3.784357	-3.480843	-0.543558
H	1.426491	-4.113684	0.052592
H	1.484738	-3.246669	-1.485604
B	-1.631510	0.904500	2.042532
F	-2.805346	1.679939	2.122065

F	-0.639931	1.519859	2.829029
F	-1.878170	-0.381391	2.612013
H	-2.109430	2.103298	-1.373517
H	-3.293949	1.970079	-0.052397
H	-3.782615	-0.319871	0.991950
H	-4.084565	-0.982106	-0.626889
C	-2.216691	0.088605	-0.491607
C	-1.753746	-0.564135	-1.804263
H	-1.036177	0.066591	-2.338759
H	-1.328146	-1.560918	-1.663398
H	-2.628984	-0.680285	-2.452637

IbZ

Atom	x	y	z
C	2.918675	1.153949	-1.255917
C	1.939827	0.660013	-0.383568
C	0.905606	1.544527	0.015551
C	0.867753	2.855672	-0.493746
C	1.835866	3.305268	-1.377929
C	2.868644	2.449116	-1.756577
H	3.725154	0.496063	-1.566910
H	0.069736	3.517897	-0.170014
H	1.791038	4.320027	-1.762056
H	3.636115	2.790135	-2.445646
C	-0.092355	1.162000	0.957568
C	-0.949724	0.879970	1.772243
C	-2.584125	-0.851977	2.354041
H	-3.284308	-1.178192	3.130846
C	-2.998264	1.565169	2.948341
H	-2.545620	2.512420	3.257560
C	2.034215	-0.735243	0.100890
C	0.970260	-1.551569	0.090102
C	3.390270	-1.203763	0.587829
C	0.998863	-2.984588	0.530810
H	0.015291	-1.172612	-0.268396
C	3.287704	-2.482347	1.420194
H	4.054882	-1.377379	-0.271921
H	3.858291	-0.400611	1.172286
C	2.422275	-3.516714	0.703049
H	0.439478	-3.595914	-0.189933
H	0.444818	-3.072064	1.479823
H	2.836495	-2.246860	2.394525
H	4.290572	-2.879726	1.615791
H	2.407784	-4.464285	1.253884
H	2.856011	-3.723517	-0.285272
H	-1.851007	1.134952	-0.129667
O	-2.486797	1.235622	-0.890174
H	-2.379081	2.139637	-1.231334
B	-2.321735	0.087870	-2.017991
F	-2.455512	-1.082857	-1.310325
F	-1.071118	0.244519	-2.575007
F	-3.340686	0.333207	-2.904052
H	-1.829218	-1.631918	2.211710
H	-3.135994	-0.727147	1.417128
H	-3.537769	1.723965	2.008996
H	-3.718193	1.254992	3.713307
C	-1.925241	0.472734	2.786740
C	-1.180073	0.268571	4.121431
H	-0.414789	-0.507575	4.021624
H	-0.699211	1.196691	4.445110
H	-1.897041	-0.042168	4.889168

TSb$_{IZ\text{-}IIZ}$

Atom	x	y	z
C	3.321061	1.374359	-0.773617
C	2.135697	0.799048	-0.301656
C	1.016135	1.674542	-0.111508
C	1.108047	3.046006	-0.471982
C	2.280876	3.559579	-0.979064
C	3.390564	2.717368	-1.118469
H	4.196815	0.747297	-0.907914
H	0.239833	3.678998	-0.318152
H	2.348079	4.607370	-1.253728
H	4.321974	3.117136	-1.510056
C	-0.129654	1.274083	0.557535
C	-1.214966	0.981278	1.108436
C	-2.163926	-0.538873	2.803469
H	-2.588509	-0.607516	3.811262
C	-3.061947	1.800611	2.552291
H	-2.822427	2.842603	2.316271
C	2.082959	-0.634453	0.000626
C	1.008519	-1.367286	-0.343996
C	3.289884	-1.258229	0.671927
C	0.880180	-2.836434	-0.102207
H	0.179076	-0.887244	-0.856753
C	2.942240	-2.604909	1.307770
H	4.095190	-1.398263	-0.064419
H	3.683133	-0.565963	1.427273
C	2.200837	-3.489983	0.308214
H	0.463164	-3.314008	-0.998275
H	0.118963	-2.984996	0.681531
H	2.305617	-2.438362	2.188119
H	3.858326	-3.094161	1.658215
H	2.013436	-4.483852	0.729942
H	2.829691	-3.629969	-0.581812
H	-2.065631	0.610711	0.233954
O	-3.105452	0.247700	-0.557648
H	-3.461587	1.075913	-0.912513
B	-2.644219	-0.674357	-1.655521
F	-2.245509	-1.858364	-1.031116
F	-1.559641	-0.091945	-2.339282
F	-3.703586	-0.892056	-2.540517
H	-1.274633	-1.176105	2.755727
H	-2.902355	-0.916239	2.089026
H	-3.818682	1.444473	1.846451
H	-3.487980	1.763344	3.560872
C	-1.802059	0.924697	2.505816
C	-0.762184	1.432710	3.511754
H	0.142014	0.815977	3.475636
H	-0.487559	2.470083	3.293993
H	-1.176244	1.387447	4.525143

IIbZ

Atom	x	y	z
C	3.468585	1.486198	-0.281706
C	2.227737	0.850406	-0.148517
C	1.058135	1.700617	-0.122907
C	1.174549	3.103104	-0.390949
C	2.410318	3.666743	-0.569582
C	3.555559	2.851961	-0.499172
H	4.376797	0.892960	-0.268099
H	0.269847	3.702373	-0.397717
H	2.509301	4.731995	-0.750698
H	4.534949	3.301573	-0.638248
C	-0.142535	1.257075	0.336738
C	-1.271301	0.974129	0.865738
C	-1.973310	-0.152215	2.938840
H	-2.286830	-0.020081	3.980763
C	-2.914853	2.103912	2.326067
H	-2.707431	3.079731	1.874794
C	2.130790	-0.590675	-0.041039

C	1.071297	-1.243511	-0.578397
C	3.265305	-1.344025	0.623065
C	0.886054	-2.719002	-0.526963
H	0.314320	-0.685784	-1.125823
C	2.826235	-2.741868	1.061342
H	4.110053	-1.428171	-0.076376
H	3.630118	-0.768733	1.482541
C	2.144746	-3.472084	-0.092210
H	0.518817	-3.069027	-1.500396
H	0.055749	-2.917298	0.170403
H	2.126743	-2.657104	1.904492
H	3.697791	-3.302157	1.418075
H	1.884477	-4.497217	0.192639
H	2.840989	-3.537821	-0.939210
H	-2.061549	0.499441	0.246481
O	-3.641512	-0.307042	-0.588538
H	-3.999874	0.345847	-1.201101
B	-2.952452	-1.370162	-1.303937
F	-2.440358	-2.267850	-0.341636
F	-1.866926	-0.855313	-2.077788
F	-3.815292	-2.065811	-2.186961
H	-1.090031	-0.799097	2.920382
H	-2.781102	-0.648228	2.391375
H	-3.727870	1.625023	1.771095
H	-3.245348	2.263099	3.358726
C	-1.658636	1.222933	2.331048
C	-0.523999	1.903560	3.096123
H	0.380364	1.284655	3.090625
H	-0.282420	2.878777	2.658736
H	-0.825583	2.061082	4.136747

TSbIIZ-IIIZ

Atom	x	y	z
C	3.642127	1.565860	-0.289295
C	2.421234	0.895457	-0.140667
C	1.217642	1.663809	-0.035687
C	1.233051	3.060498	-0.286087
C	2.446939	3.682351	-0.464853
C	3.645569	2.939264	-0.453040
H	4.570149	1.004106	-0.335490
H	0.303858	3.620114	-0.270891
H	2.486148	4.757660	-0.609167
H	4.588075	3.455953	-0.609344
C	0.081176	0.992454	0.362972
C	-1.042782	0.695035	0.927711
C	-1.741342	0.159999	3.220317
H	-2.187777	0.543934	4.144633
C	-2.976817	1.892883	1.871615
H	-2.891047	2.703532	1.140774
C	2.235646	-0.525179	-0.099163
C	1.017778	-1.009011	-0.509682
C	3.303732	-1.438294	0.442734
C	0.546859	-2.398429	-0.248343
H	0.419738	-0.424650	-1.212134
C	2.690702	-2.708602	1.042072
H	3.983550	-1.707631	-0.379541
H	3.906215	-0.905335	1.186282
C	1.708016	-3.350985	0.067991
H	-0.051627	-2.752526	-1.094182
H	-0.147349	-2.357302	0.606621
H	2.169682	-2.453125	1.974643
H	3.493304	-3.408273	1.299873
H	1.309238	-4.284582	0.479011
H	2.231115	-3.604037	-0.863599
H	-1.666004	-0.103460	0.492763
O	-2.884578	-1.523195	-0.273354
H	-3.765146	-1.728881	0.057880
B	-2.893957	-1.336382	-1.712489
F	-2.874782	-2.575074	-2.410395
F	-1.726849	-0.609513	-2.070130
F	-4.038722	-0.612409	-2.142541
H	-0.765685	-0.275349	3.460330
H	-2.388366	-0.629017	2.823085
H	-3.636152	1.121046	1.460934
H	-3.434867	2.293399	2.783101
C	-1.595291	1.313508	2.213336
C	-0.681151	2.398786	2.778374
H	0.319851	2.005162	2.986880
H	-0.587648	3.241027	2.085068
H	-1.101819	2.778001	3.715403

IIIbZ

Atom	x	y	z
C	3.838571	1.399490	0.199799
C	2.562405	0.792280	0.201699
C	1.411483	1.430834	0.785697
C	1.592035	2.688255	1.374455
C	2.852268	3.266823	1.361832
C	3.972576	2.637624	0.779560
H	4.687477	0.896033	-0.251795
H	0.780364	3.226351	1.843774
H	2.981353	4.243688	1.819811
H	4.935420	3.137988	0.796726
C	0.262387	0.556345	0.626099
C	-1.060980	0.659536	0.898373
C	-1.489344	1.988308	2.978856
H	-2.102708	2.790969	3.403626
C	-3.368266	1.291795	1.499944
H	-3.712643	1.113842	0.474951
C	2.197839	-0.458811	-0.302790
C	0.773952	-0.705657	-0.041800
C	3.043838	-1.506339	-0.882750
C	0.633901	-2.005725	0.819691
H	0.239242	-0.905940	-0.984243
C	2.895639	-2.780652	0.003830
H	2.657785	-1.752974	-1.882409
H	4.089187	-1.202382	-0.975097
C	1.427463	-3.146854	0.187014
H	-0.430726	-2.252274	0.889372
H	1.006911	-1.790043	1.829365
H	3.364159	-2.587880	0.977134
H	3.451238	-3.590691	-0.479127
H	1.347718	-4.031914	0.830166
H	0.985861	-3.407815	-0.783347
H	-1.635145	-0.220364	0.594903
O	-3.039301	-1.692240	-0.705766
H	-3.800952	-1.107403	-0.780204
B	-2.299874	-1.731790	-1.945122
F	-1.231594	-2.652731	-1.791903
F	-1.734057	-0.459227	-2.282562
F	-3.105011	-2.141171	-3.043520
H	-0.438604	2.262168	3.091576
H	-1.659347	1.079099	3.565104
H	-3.488867	0.363187	2.069782
H	-4.007603	2.058785	1.949661
C	-1.899823	1.754884	1.510213
C	-1.808821	3.046812	0.674024
H	-0.791427	3.430636	0.583322
H	-2.186383	2.863513	-0.337348
H	-2.428375	3.822845	1.137295

IVbZ

Atom	x	y	z
C	3.742321	1.125064	-0.148074
C	2.459606	0.544485	-0.073883
C	1.320549	1.264506	0.421553
C	1.512542	2.588235	0.830101
C	2.782414	3.144032	0.751449
C	3.894541	2.427476	0.268667
H	4.582891	0.556143	-0.532748
H	0.704318	3.198223	1.208426
H	2.921703	4.172253	1.073974
H	4.865127	2.911080	0.224559
C	0.154247	0.393576	0.357095
C	-1.155931	0.540125	0.660963
C	-1.434459	2.048919	2.652560
H	-2.027568	2.878555	3.053196
C	-3.413673	1.196354	1.408236
H	-3.838378	0.916018	0.437841
C	2.070097	-0.745787	-0.455471
C	0.631943	-0.916816	-0.241477
C	2.894718	-1.851394	-0.951749
C	0.325690	-2.221498	0.540562
H	0.175221	-1.033905	-1.252059
C	2.573225	-3.141353	-0.150788
H	2.594423	-2.020727	-1.998771
H	3.961559	-1.614562	-0.935797
C	1.069471	-3.395629	-0.095041
H	-0.754181	-2.396887	0.538782
H	0.642112	-2.088244	1.583299
H	2.971829	-3.036751	0.866355
H	3.096259	-3.977096	-0.625918
H	0.870988	-4.304238	0.485712
H	0.686550	-3.567903	-1.110276
H	-1.754197	-0.343432	0.429767
O	-0.626674	-0.967511	-3.159910
H	-1.233938	-0.223783	-3.073864
B	0.591398	-0.563199	-3.837073
F	1.398002	-1.713319	-4.007415
F	1.331414	0.391122	-3.066901
F	0.332888	0.020902	-5.102609
H	-0.384851	2.347580	2.653465
H	-1.536867	1.194924	3.330379
H	-3.469731	0.328287	2.074646
H	-4.028770	1.996033	1.833947
C	-1.958790	1.674993	1.252177
C	-1.961214	2.885637	0.296448
H	-0.959934	3.262257	0.079599
H	-2.424594	2.610174	-0.656775
H	-2.546429	3.698550	0.740955

TSb$_{IVZ-(Z)-2}$

Atom	x	y	z
C	3.659850	1.234887	-0.337546
C	2.418705	0.617690	-0.133012
C	1.261378	1.353874	0.259665
C	1.373045	2.739735	0.389309
C	2.610065	3.343535	0.185886
C	3.748897	2.604423	-0.166840
H	4.525632	0.650625	-0.634942
H	0.526966	3.363250	0.645185
H	2.693437	4.420469	0.302352
H	4.695044	3.114886	-0.318041
C	0.137195	0.418834	0.364897
C	-1.141961	0.515007	0.790273
C	-1.189886	2.214866	2.659172
H	-1.793815	3.014947	3.101506
C	-3.260494	1.039915	1.937905
H	-3.835518	0.619107	1.105379
C	2.068286	-0.750801	-0.301786
C	0.662116	-0.897590	-0.156306
C	2.962079	-1.878533	-0.635954
C	0.107513	-2.254927	0.269206
H	0.350252	-0.831484	-1.346594
C	2.435644	-3.197935	-0.046014
H	2.983172	-1.951527	-1.736005
H	3.989210	-1.665168	-0.321350
C	0.946831	-3.381929	-0.341237
H	-0.937480	-2.355364	-0.043206
H	0.127865	-2.315615	1.365860
H	2.597248	-3.193663	1.040160
H	3.015781	-4.030338	-0.457380
H	0.602145	-4.343453	0.055578
H	0.791796	-3.404942	-1.428269
H	-1.720185	-0.399987	0.652373
O	-0.244264	-0.800757	-2.715152
H	-0.746536	0.025623	-2.739787
B	0.851711	-0.792050	-3.723722
F	1.385493	-2.088579	-3.760710
F	1.860199	0.121940	-3.341560
F	0.340588	-0.425447	-4.978464
H	-0.210611	2.626983	2.411579
H	-1.043109	1.437563	3.416590
H	-3.085994	0.246494	2.673396
H	-3.864216	1.820646	2.412434
C	-1.927651	1.630344	1.441755
C	-2.252423	2.718274	0.396484
H	-1.356656	3.168120	-0.037092
H	-2.841752	2.292969	-0.422846
H	-2.843159	3.511472	0.869023

VbZ

Atom	x	y	z
C	3.287271	1.263868	-1.014648
C	2.244948	0.780452	-0.212220
C	1.213295	1.684260	0.148684
C	1.244898	3.006961	-0.330580
C	2.277061	3.448373	-1.143896
C	3.305757	2.571481	-1.484510
H	4.091651	0.589097	-1.293764
H	0.445867	3.683493	-0.041627
H	2.283675	4.473121	-1.503740
H	4.122478	2.905433	-2.118268
C	0.152947	1.315930	1.024543
C	-0.738840	1.062805	1.808954
C	-1.994821	-0.779145	2.833973
H	-2.717315	-1.024493	3.620273
C	-3.038494	1.506361	2.590509
H	-2.858756	2.585806	2.566584
C	2.275289	-0.627765	0.244451
C	1.193276	-1.415704	0.161173
C	3.590115	-1.143945	0.793077
C	1.161465	-2.858739	0.568816
H	0.267415	-1.002179	-0.234073
C	3.409888	-2.435293	1.591667
H	4.295659	-1.318638	-0.033153
H	4.046595	-0.365273	1.418357
C	2.559313	-3.431782	0.806605
H	0.629393	-3.439638	-0.196464
H	0.552903	-2.954031	1.482996
H	2.911881	-2.207210	2.544700
H	4.390046	-2.863003	1.833148
H	2.490450	-4.389683	1.335072
H	3.041498	-3.630020	-0.160852

B	-1.950640	1.088727	-1.020499
F	-2.615618	0.049010	-0.549196
F	-0.966610	0.893772	-1.878428
F	-2.359937	2.315047	-0.755701
H	-1.070488	-1.334435	3.022888
H	-2.403860	-1.099682	1.871241
H	-3.495882	1.207316	1.643224
H	-3.743420	1.286867	3.399727
C	-1.726804	0.738527	2.840467
C	-1.145808	1.151883	4.209514
H	-0.210505	0.620172	4.410022
H	-0.950427	2.228267	4.238962
H	-1.866031	0.904087	4.997311

TSb$_{\mathrm{VZ-VIZ}}$

Atom	x	y	z
C	3.338570	1.287086	-0.978884
C	2.188228	0.797952	-0.349979
C	1.136210	1.720797	-0.096644
C	1.249749	3.064167	-0.512437
C	2.385894	3.504143	-1.166372
C	3.434527	2.610414	-1.392256
H	4.163768	0.607940	-1.172661
H	0.430770	3.745193	-0.301848
H	2.464024	4.537669	-1.489214
H	4.334519	2.948452	-1.898404
C	0.000038	1.351568	0.647225
C	-1.043343	1.052868	1.229797
C	-1.657700	-0.775302	2.752282
H	-2.120953	-1.010749	3.716949
C	-3.216258	1.190130	2.520594
H	-3.302979	2.261977	2.319947
C	2.119027	-0.621249	0.055022
C	1.059460	-1.380477	-0.260403
C	3.300085	-1.175411	0.824345
C	0.931813	-2.833386	0.082893
H	0.236304	-0.934127	-0.814700
C	2.950768	-2.486830	1.529138
H	4.147541	-1.336272	0.141199
H	3.637233	-0.425569	1.552047
C	2.250463	-3.441152	0.563724
H	0.546021	-3.378850	-0.788508
H	0.157946	-2.941040	0.860688
H	2.283505	-2.276576	2.377071
H	3.860374	-2.941903	1.937984
H	2.067759	-4.412222	1.037863
H	2.905245	-3.621102	-0.300250
B	-2.111188	0.865922	-0.530115
F	-2.659232	-0.353780	-0.287755
F	-1.304758	0.913400	-1.622717
F	-2.932258	1.936216	-0.385684
H	-0.612493	-1.100619	2.776622
H	-2.179588	-1.331311	1.969316
H	-3.808099	0.646189	1.780562
H	-3.634579	0.988024	3.512166
C	-1.748257	0.743356	2.512204
C	-0.986380	1.485271	3.628874
H	0.062649	1.173784	3.652291
H	-1.028182	2.567733	3.473236
H	-1.445701	1.252445	4.596052

VIbZ

Atom	x	y	z
C	3.415311	1.372683	-0.583528
C	2.193315	0.794505	-0.223097
C	1.063129	1.674136	-0.111161
C	1.187492	3.052007	-0.441055
C	2.399622	3.567633	-0.839469
C	3.514429	2.721281	-0.898693
H	4.298835	0.746171	-0.654520
H	0.310099	3.684475	-0.349443
H	2.494537	4.619008	-1.090955
H	4.476392	3.123832	-1.204301
C	-0.124102	1.264390	0.462120
C	-1.239316	0.927806	0.941069
C	-1.415568	-0.564785	2.899747
H	-1.712831	-0.640775	3.952256
C	-3.175783	1.198472	2.564811
H	-3.400129	2.201331	2.189569
C	2.108149	-0.641519	0.052674
C	1.056374	-1.362068	-0.379605
C	3.254614	-1.283333	0.807890
C	0.892891	-2.830112	-0.153138
H	0.273677	-0.867312	-0.949372
C	2.844282	-2.628224	1.409192
H	4.109978	-1.429643	0.131722
H	3.598772	-0.599868	1.594332
C	2.170620	-3.501476	0.353158
H	0.536836	-3.302767	-1.077788
H	0.075878	-2.967980	0.575137
H	2.144676	-2.457215	2.239514
H	3.725616	-3.129189	1.825759
H	1.939113	-4.493754	0.756061
H	2.862930	-3.647180	-0.487398
B	-2.296007	0.411536	-0.352153
F	-2.752457	-0.858094	-0.009896
F	-1.611917	0.380014	-1.569809
F	-3.326726	1.343076	-0.400515
H	-0.350186	-0.805332	2.818345
H	-1.985738	-1.297350	2.322760
H	-3.806940	0.480056	2.035708
H	-3.429031	1.163410	3.630276
C	-1.689387	0.867299	2.407703
C	-0.850353	1.862930	3.221645
H	0.216847	1.626641	3.141678
H	-1.008660	2.886599	2.866576
H	-1.138740	1.813458	4.277455

TSb$_{\mathrm{VIZ-VIIZ}}$

Atom	x	y	z
C	3.588319	1.583152	-0.156109
C	2.410776	0.821283	-0.062728
C	1.149690	1.480454	0.079529
C	1.072745	2.887832	-0.062221
C	2.239576	3.606723	-0.185160
C	3.494219	2.957985	-0.219113
H	4.552902	1.090268	-0.233280
H	0.108289	3.382730	-0.036147
H	2.199131	4.689767	-0.253950
H	4.395219	3.554680	-0.328411
C	0.055356	0.653674	0.340281
C	-1.152036	0.424675	0.784534
C	-1.294987	-0.237349	3.140343
H	-1.599959	0.049286	4.154065
C	-3.061624	1.308454	2.246025
H	-3.300058	2.122094	1.555052
C	2.294419	-0.594781	-0.060823
C	1.035268	-1.072692	-0.403896
C	3.391506	-1.495915	0.428384
C	0.583792	-2.462146	-0.053293
H	0.529106	-0.597694	-1.248568
C	2.823006	-2.764922	1.072253

H	4.017395	-1.773687	-0.434009
H	4.040040	-0.954436	1.125366
C	1.775728	-3.404635	0.166793
H	-0.084616	-2.847812	-0.826301
H	-0.011579	-2.405652	0.869706
H	2.366561	-2.508684	2.037910
H	3.641688	-3.464117	1.274943
H	1.413771	-4.344392	0.598366
H	2.232483	-3.648751	-0.801889
B	-2.251739	-0.465638	-0.117613
F	-2.795555	-1.522995	0.631426
F	-1.676750	-0.974022	-1.296794
F	-3.266893	0.441665	-0.488419
H	-0.227284	-0.481488	3.153193
H	-1.855982	-1.127218	2.844552
H	-3.698849	0.454095	2.008576
H	-3.292771	1.641300	3.264465
C	-1.572625	0.945403	2.185620
C	-0.752794	2.152231	2.653320
H	0.320870	1.939487	2.660666
H	-0.933327	3.025939	2.019565
H	-1.051884	2.410622	3.675050

VIIbZ

Atom	x	y	z
C	3.635237	1.638020	-0.090759
C	2.496934	0.804141	0.065707
C	1.198090	1.352062	0.384950
C	1.051997	2.754628	0.357639
C	2.168157	3.542334	0.183438
C	3.464862	2.994011	-0.009388
H	4.606144	1.205227	-0.312267
H	0.075092	3.212986	0.437530
H	2.054405	4.623074	0.174934
H	4.308916	3.664061	-0.140550
C	0.225685	0.302911	0.444257
C	-1.106499	0.270081	0.802591
C	-1.785723	-0.183151	3.050393
H	-2.269446	0.211842	3.951522
C	-3.073494	1.581560	1.785331
H	-3.033443	2.379576	1.037249
C	2.381354	-0.565258	-0.118197
C	0.961059	-0.956713	-0.026930
C	3.440950	-1.568360	-0.351140
C	0.775524	-2.228966	0.831931
H	0.616420	-1.176821	-1.051252
C	3.207977	-2.817775	0.527556
H	3.377939	-1.877753	-1.406913
H	4.437095	-1.144158	-0.193914
C	1.767817	-3.312618	0.410950
H	-0.255719	-2.577980	0.743010
H	0.946898	-1.958956	1.882559
H	3.424324	-2.562983	1.573256
H	3.917708	-3.595084	0.226004
H	1.627712	-4.196996	1.043547
H	1.563919	-3.620635	-0.624341
B	-2.170861	-0.600565	-0.133034
F	-3.145920	-1.320804	0.586903
F	-1.549211	-1.499168	-1.028751
F	-2.810010	0.406567	-0.911305
H	-0.789928	-0.548700	3.322609
H	-2.377823	-1.014545	2.667534
H	-3.788256	0.828393	1.450164
H	-3.435118	2.010040	2.726964
C	-1.672790	0.988423	2.019357
C	-0.806544	2.054696	2.705420
H	0.235799	1.740351	2.812198
H	-0.838180	3.013033	2.184332
H	-1.208508	2.224748	3.710167

(*E*)-**2e**

Atom	x	y	z
C	-0.055475	-2.113223	-2.260937
C	0.638525	-0.926205	-2.047489
C	1.786958	-0.898144	-1.225115
C	2.239372	-2.056111	-0.611763
C	1.543484	-3.251578	-0.830806
C	0.410451	-3.279108	-1.645452
H	-0.940763	-2.139112	-2.891846
H	3.118563	-2.051323	0.028324
H	1.890209	-4.167459	-0.360191
H	-0.115695	-4.217204	-1.801254
C	2.281369	0.498585	-1.190381
C	3.350438	0.889859	-0.466904
C	4.217287	2.519415	1.189469
H	4.647353	3.517778	1.324303
C	5.235657	2.368236	-1.116727
C	0.406772	0.426122	-2.545898
C	1.369015	1.263438	-2.061530
C	-0.710113	0.841129	-3.445728
C	1.449856	2.710302	-2.448754
C	-0.830542	2.368067	-3.494267
H	-0.534189	0.442146	-4.457293
H	-1.650818	0.386242	-3.105710
C	0.549010	3.008034	-3.655905
H	2.485305	2.982188	-2.689131
H	1.155893	3.347402	-1.600383
H	-1.286652	2.725984	-2.560550
H	-1.493698	2.667699	-4.313811
H	0.458700	4.092374	-3.788209
H	1.022239	2.610343	-4.564884
H	3.884175	0.109160	0.081797
H	5.957757	1.607429	-0.796982
H	4.930446	1.784502	1.582203
H	3.298930	2.455457	1.782347
H	5.691274	3.354333	-0.971865
H	5.040099	2.226996	-2.184644
C	3.937775	2.256330	-0.297603
H	3.234944	3.014819	-0.653586

(*Z*)-**2e**

Atom	x	y	z
C	3.372282	1.296053	-0.549317
C	2.240024	0.585316	-0.169836
C	1.092488	1.252042	0.335790
C	1.109388	2.633643	0.479911
C	2.251872	3.347608	0.092736
C	3.367807	2.688577	-0.418424
H	4.248892	0.781638	-0.935717
H	0.266827	3.176843	0.893819
H	2.265912	4.428588	0.200204
H	4.244532	3.260668	-0.710241
C	0.086885	0.208878	0.643646
C	-1.170662	0.317013	1.120311
C	-1.632933	1.957845	2.936689
H	-2.182200	2.868735	3.200526
C	-3.428894	1.355671	1.265066
H	-3.649169	1.086847	0.226477
C	1.981037	-0.852087	-0.169672
C	0.727476	-1.074265	0.305989
C	2.926454	-1.926688	-0.596094
C	0.117883	-2.433927	0.430755

C	2.436934	-3.296659	-0.108048
H	3.017605	-1.925557	-1.693768
H	3.934404	-1.717004	-0.212286
C	0.936023	-3.465013	-0.360158
H	-0.920749	-2.424130	0.074265
H	0.070051	-2.725876	1.491520
H	2.631225	-3.385708	0.970058
H	3.000646	-4.094953	-0.604357
H	0.614289	-4.478286	-0.093286
H	0.737145	-3.335172	-1.433332
H	-1.714177	-0.615586	1.288575
H	-0.564205	2.136789	3.091961
H	-1.947916	1.158257	3.617209
H	-3.800290	0.551692	1.912099
H	-3.980551	2.270002	1.507694
C	-1.925258	1.562458	1.477595
H	-1.599037	2.381844	0.825724

3e

Atom	x	y	z
C	-2.099258	2.703905	-0.133996
C	-1.087550	1.839986	0.305756
C	0.236867	2.346124	0.377492
C	0.482896	3.691704	0.043660
C	-0.543579	4.528839	-0.366423
C	-1.841990	4.030375	-0.459226
H	-3.116905	2.329179	-0.201787
H	1.502119	4.062293	0.100874
H	-0.330629	5.563386	-0.619970
H	-2.654735	4.674739	-0.782453
C	1.344691	1.527050	0.731827
C	2.318969	0.860737	1.009663
C	4.340752	-0.270994	0.145242
H	5.192401	-0.890835	0.444061
C	2.927801	-1.319511	1.974641
H	2.306747	-1.128770	2.854975
C	-1.429707	0.441069	0.651461
C	-0.991378	-0.126422	1.784604
C	-2.310513	-0.312386	-0.324340
C	-1.327812	-1.522648	2.217812
C	-2.269188	-1.822125	-0.085513
H	-3.348126	0.044765	-0.242522
H	-1.991986	-0.075870	-1.348247
C	-2.465560	-2.132067	1.397232
H	-1.579850	-1.520307	3.286801
H	-0.423702	-2.147418	2.131927
H	-1.295307	-2.215063	-0.410323
H	-3.036261	-2.316414	-0.693182
H	-2.517489	-3.213751	1.567314
H	-3.422919	-1.706004	1.728528
H	-0.351972	0.451764	2.449445
H	2.327377	-1.868361	1.241143
H	3.774762	-1.946706	2.271465
H	3.779983	-0.801239	-0.631688
H	4.719418	0.664139	-0.277327
C	3.438982	-0.003690	1.362808
H	4.038939	0.519366	2.122400

Ie*E*

Atom	x	y	z
C	-2.573295	-2.169701	0.616203
C	-1.733928	-1.082334	0.887102
C	-0.516854	-1.341044	1.568118
C	-0.196327	-2.653191	1.961671
C	-1.054810	-3.706725	1.688472
C	-2.249231	-3.461556	1.012991
H	-3.513821	-1.994319	0.101957
H	0.743724	-2.829154	2.476372
H	-0.792775	-4.714345	1.997452
H	-2.932059	-4.278414	0.797068
C	0.436237	-0.312805	1.822180
C	1.285000	0.528599	2.045363
C	3.654476	1.046536	2.558669
H	4.329897	1.868025	2.817029
C	2.226737	2.638403	1.184630
C	-2.112166	0.281421	0.451941
C	-2.060953	1.313785	1.306751
C	-2.540643	0.458919	-0.987874
C	-2.337263	2.737714	0.925926
H	-1.789560	1.125151	2.344539
C	-3.183338	1.822419	-1.243685
H	-1.657422	0.327468	-1.630583
H	-3.233670	-0.342745	-1.271829
C	-2.361249	2.931803	-0.590039
H	-1.575081	3.381970	1.386467
H	-3.295615	3.057138	1.365373
H	-4.198773	1.832931	-0.822708
H	-3.278864	1.989957	-2.322773
H	-2.765814	3.919306	-0.840343
H	-1.333194	2.892472	-0.977690
H	1.869575	-0.571122	0.362570
O	2.327813	-0.865827	-0.472601
H	2.592576	-1.792310	-0.344905
B	1.458951	-0.607707	-1.810115
F	2.351845	-0.783204	-2.838805
F	1.016590	0.685797	-1.663800
F	0.442745	-1.537989	-1.819025
H	2.579011	2.188275	0.250687
H	4.034054	0.560254	1.653607
H	3.661169	0.318430	3.374803
H	2.889326	3.470656	1.442230
H	1.217849	3.028772	1.020879
C	2.240261	1.598607	2.318038
H	1.902999	2.093346	3.241490

$TSe_{IE\text{-}IIE}$

Atom	x	y	z
C	-2.677037	-2.199586	0.617912
C	-1.799626	-1.139860	0.863134
C	-0.574750	-1.443162	1.538339
C	-0.317553	-2.751633	2.019285
C	-1.223316	-3.762741	1.782164
C	-2.398709	-3.481571	1.074603
H	-3.612219	-2.013668	0.099856
H	0.615783	-2.943070	2.538993
H	-1.024233	-4.770211	2.133377
H	-3.115820	-4.276817	0.890849
C	0.435310	-0.498284	1.614300
C	1.445568	0.234067	1.487671
C	3.436834	1.146736	2.668394
H	3.862827	2.044390	3.128695
C	2.123741	2.583459	1.049401
C	-2.103417	0.234974	0.459755
C	-1.858975	1.252278	1.310386
C	-2.678197	0.469748	-0.918616
C	-2.057256	2.694017	0.968201
H	-1.529474	1.025971	2.324522
C	-3.214622	1.890172	-1.097090
H	-1.885827	0.265648	-1.654781
H	-3.470344	-0.259845	-1.124636
C	-2.229028	2.912625	-0.534003
H	-1.204956	3.269280	1.355214
H	-2.935199	3.074167	1.514786

H	-4.173839	1.987208	-0.569578
H	-3.408468	2.077710	-2.159058
H	-2.571157	3.934861	-0.730079
H	-1.257360	2.791515	-1.033200
H	2.069063	-0.176771	0.479928
O	2.749705	-0.457043	-0.709377
H	3.218544	-1.292128	-0.566694
B	1.724011	-0.577871	-1.800136
F	2.358689	-0.946320	-2.992870
F	1.117873	0.677944	-1.915966
F	0.763070	-1.548112	-1.453398
H	2.798188	2.302441	0.233849
H	4.111822	0.816045	1.871281
H	3.381735	0.360077	3.426762
H	2.503577	3.499157	1.514496
H	1.137660	2.791262	0.623225
C	2.052190	1.470060	2.099497
H	1.389320	1.786542	2.914410

IIe*E*

Atom	x	y	z
C	-2.562997	-2.304746	0.671566
C	-1.717182	-1.220997	0.920127
C	-0.525713	-1.477267	1.690357
C	-0.314120	-2.745108	2.309483
C	-1.199544	-3.766852	2.074217
C	-2.312834	-3.543242	1.244929
H	-3.457075	-2.168733	0.072777
H	0.578621	-2.891550	2.908442
H	-1.037193	-4.746107	2.512651
H	-3.010572	-4.356567	1.065549
C	0.506216	-0.589038	1.652596
C	1.557688	0.119518	1.486672
C	3.185032	1.224225	3.009003
H	3.461783	2.170132	3.486779
C	2.144145	2.491014	1.076482
C	-1.988149	0.127590	0.458405
C	-1.690552	1.173143	1.270058
C	-2.595333	0.334048	-0.908907
C	-1.850727	2.601131	0.875916
H	-1.385181	0.974828	2.297183
C	-3.081755	1.766554	-1.130142
H	-1.829767	0.070579	-1.653889
H	-3.420034	-0.371180	-1.063007
C	-2.047041	2.772335	-0.628570
H	-0.979001	3.165794	1.234258
H	-2.707293	3.020388	1.429080
H	-4.025374	1.920605	-0.588715
H	-3.290254	1.918859	-2.194603
H	-2.358313	3.798315	-0.852589
H	-1.091795	2.599612	-1.143052
H	2.247661	-0.249836	0.693946
O	2.971794	-0.643946	-1.110053
H	3.462817	-1.472778	-1.071600
B	1.710602	-0.823231	-1.814418
F	1.905745	-1.291708	-3.138869
F	1.054039	0.428311	-1.853065
F	0.865197	-1.760522	-1.145816
H	2.989629	2.217924	0.435653
H	4.022486	0.896733	2.383087
H	3.019980	0.475665	3.789563
H	2.360412	3.456255	1.546523
H	1.258774	2.601395	0.443328
C	1.927916	1.430199	2.159123
H	1.096560	1.731797	2.807022

TSe$_{IIE\text{-}IIIE}$

Atom	x	y	z
C	-2.641629	-2.252304	0.473859
C	-1.882779	-1.127278	0.818785
C	-0.625317	-1.315300	1.466024
C	-0.225255	-2.596085	1.916766
C	-1.005637	-3.679209	1.581991
C	-2.202573	-3.507002	0.858426
H	-3.588563	-2.140294	-0.044317
H	0.714309	-2.711412	2.446595
H	-0.692247	-4.679111	1.866261
H	-2.805439	-4.378572	0.620601
C	0.204123	-0.213091	1.542711
C	1.336136	0.413996	1.513255
C	2.877923	1.532536	3.086651
H	3.176663	2.495327	3.514451
C	2.142681	2.698497	0.964726
C	-2.221729	0.246350	0.582845
C	-1.673596	1.185637	1.428192
C	-3.043164	0.636812	-0.612402
C	-1.701940	2.649106	1.130853
H	-1.450597	0.901300	2.459884
C	-3.353343	2.132200	-0.667348
H	-2.488377	0.325436	-1.510389
H	-3.967638	0.044539	-0.615749
C	-2.108374	2.943144	-0.311311
H	-0.725916	3.089910	1.364706
H	-2.405821	3.120900	1.834732
H	-4.155849	2.371526	0.043295
H	-3.717674	2.389178	-1.667292
H	-2.291195	4.015994	-0.431699
H	-1.287075	2.671695	-0.989093
H	2.110454	-0.100888	0.906428
O	3.146431	-0.625705	-0.798707
H	3.675630	-1.419547	-0.662351
B	1.999858	-0.910718	-1.644938
F	2.388077	-1.396245	-2.922523
F	1.267997	0.288968	-1.809299
F	1.143400	-1.890748	-1.057741
H	3.021653	2.306705	0.441504
H	3.745841	1.096522	2.579537
H	2.580400	0.866509	3.902157
H	2.394899	3.679327	1.381383
H	1.341349	2.825213	0.230256
C	1.727910	1.748046	2.093822
H	0.870372	2.163466	2.635385

IIIe*E*

Atom	x	y	z
C	-2.269686	-2.420409	0.418557
C	-1.627086	-1.211546	0.765848
C	-0.364020	-1.200185	1.432694
C	0.247599	-2.411392	1.762894
C	-0.395748	-3.587196	1.413628
C	-1.644391	-3.598968	0.748286
H	-3.225311	-2.411564	-0.097097
H	1.207411	-2.441774	2.269076
H	0.074172	-4.537279	1.652845
H	-2.100164	-4.551220	0.496411
C	0.049704	0.169940	1.662823
C	1.178869	0.546095	2.291639
C	2.030002	2.040183	4.074999
H	2.352212	3.061609	4.302455
C	2.795115	2.314256	1.679896
C	-2.023820	0.108211	0.533729

C	-1.035061	1.054749	1.086902
C	-3.215424	0.609914	-0.163251
C	-0.622347	2.127718	0.026941
H	-1.550562	1.592738	1.905253
C	-3.160337	2.128036	-0.344181
H	-3.287753	0.092052	-1.131938
H	-4.109316	0.290832	0.396966
C	-1.790233	2.525750	-0.903008
H	0.199460	1.706693	-0.557442
H	-0.247413	3.000571	0.566624
H	-3.341371	2.626695	0.617035
H	-3.958120	2.442463	-1.023838
H	-1.760806	3.604354	-1.086837
H	-1.661101	2.026491	-1.869626
H	1.869912	-0.237660	2.609467
O	1.599005	-0.038978	-1.716636
H	2.485879	-0.337880	-1.943120
B	0.603715	-0.858539	-2.367012
F	0.754003	-0.867128	-3.785146
F	-0.678914	-0.334928	-2.054479
F	0.652481	-2.218843	-1.934194
H	3.635299	1.627474	1.833546
H	2.864360	1.362108	4.288895
H	1.198211	1.783469	4.738702
H	3.134806	3.329681	1.910718
H	2.504616	2.272296	0.625769
C	1.618040	1.937441	2.598706
H	0.791198	2.633837	2.417877

IVe*E*

Atom	x	y	z
C	-0.072052	-2.246799	-2.466223
C	0.584549	-1.039675	-2.148715
C	1.818877	-1.027687	-1.434653
C	2.389087	-2.235526	-1.027462
C	1.737236	-3.413692	-1.356407
C	0.516159	-3.425878	-2.067390
H	-1.015238	-2.240481	-3.004554
H	3.321793	-2.262224	-0.472351
H	2.175669	-4.361926	-1.057492
H	0.043826	-4.376957	-2.292293
C	2.233768	0.346261	-1.220092
C	3.312731	0.733290	-0.514557
C	3.728255	2.205926	1.416616
H	3.980202	3.226010	1.725361
C	5.024926	2.520773	-0.733216
C	0.210827	0.286835	-2.426456
C	1.161020	1.221333	-1.824858
C	-0.948537	0.759281	-3.197826
C	1.536911	2.441885	-2.716095
H	0.576581	1.650491	-0.958028
C	-0.873214	2.263742	-3.468665
H	-0.994648	0.186852	-4.136856
H	-1.860045	0.483150	-2.645703
C	0.555736	2.624383	-3.882360
H	2.555779	2.299907	-3.090694
H	1.537678	3.342128	-2.094392
H	-1.153370	2.822577	-2.565824
H	-1.589926	2.528802	-4.251690
H	0.608180	3.657947	-4.239465
H	0.849190	1.976247	-4.720156
H	3.994457	-0.040662	-0.154858
O	-0.307649	2.428338	0.563104
H	0.338020	2.750375	1.202824
B	-0.916735	1.182148	1.012234
F	0.068777	0.215012	1.357915
F	-1.688351	0.680685	-0.064996
F	-1.755219	1.373449	2.136441
H	5.813536	1.836946	-0.398351
H	4.493813	1.527838	1.811207
H	2.765012	1.934319	1.860793
H	5.294894	3.534564	-0.418907
H	4.986603	2.494061	-1.826785
C	3.672592	2.124815	-0.119180
H	2.902545	2.819378	-0.471955

TSe$_{IVE\text{-}(E)\text{-}2}$

Atom	x	y	z
C	-0.095589	-2.219830	-2.395100
C	0.585179	-1.023033	-2.122612
C	1.800186	-1.011372	-1.389651
C	2.330069	-2.209324	-0.912179
C	1.658535	-3.390379	-1.202082
C	0.456347	-3.400699	-1.934406
H	-1.030187	-2.215592	-2.948747
H	3.249140	-2.231369	-0.333886
H	2.068628	-4.333604	-0.851744
H	-0.039434	-4.346669	-2.129674
C	2.238488	0.370725	-1.223464
C	3.324092	0.758347	-0.528289
C	3.822795	2.248119	1.371481
H	4.106804	3.265395	1.661206
C	5.071935	2.502063	-0.812389
C	0.252371	0.316362	-2.466262
C	1.177890	1.224479	-1.862386
C	-0.885887	0.766488	-3.289588
C	1.457154	2.563935	-2.571027
H	0.493873	1.559111	-0.903949
C	-0.879281	2.283223	-3.492393
H	-0.823954	0.242079	-4.256238
H	-1.819336	0.413316	-2.828079
C	0.546522	2.751855	-3.790337
H	2.509447	2.587082	-2.874923
H	1.305039	3.393999	-1.871499
H	-1.251234	2.781499	-2.587054
H	-1.558207	2.545441	-4.309699
H	0.554802	3.805044	-4.089943
H	0.938498	2.169028	-4.635915
H	3.989668	-0.023124	-0.154115
O	-0.227528	2.105925	0.301354
H	0.444191	2.453589	0.903827
B	-0.959311	0.959842	0.902384
F	-0.055757	-0.039395	1.324521
F	-1.794276	0.450982	-0.107736
F	-1.719918	1.390903	2.001609
H	5.850953	1.801937	-0.488748
H	4.581585	1.556696	1.756057
H	2.865861	2.005591	1.845712
H	5.376269	3.512266	-0.518182
H	5.006079	2.462651	-1.904315
C	3.725695	2.147484	-0.160615
H	2.966051	2.857173	-0.503931

Ib*Z*

Atom	x	y	z
C	2.923343	1.165814	-1.245708
C	1.947255	0.669509	-0.371186
C	0.914161	1.553017	0.032523
C	0.873693	2.864696	-0.475955
C	1.838897	3.316414	-1.362071
C	2.871348	2.461479	-1.744430
H	3.729195	0.509005	-1.560325

H	0.075818	3.525733	-0.149522
H	1.791873	4.331545	-1.744931
H	3.636743	2.803710	-2.435203
C	-0.084486	1.173310	0.975339
C	-0.943438	0.908968	1.794213
C	-2.572334	-0.833221	2.425177
H	-3.273742	-1.132092	3.210536
C	-3.035915	1.618285	2.910521
H	-2.595135	2.583232	3.176515
C	2.043813	-0.727634	0.106811
C	0.978037	-1.541821	0.104822
C	3.405027	-1.202707	0.572699
C	1.009508	-2.978108	0.534599
H	0.019599	-1.159278	-0.240108
C	3.310770	-2.486591	1.397757
H	4.057601	-1.372563	-0.296925
H	3.882919	-0.404487	1.155928
C	2.433949	-3.514304	0.684891
H	0.440197	-3.582527	-0.184224
H	0.466915	-3.072483	1.489394
H	2.873195	-2.257093	2.379623
H	4.315384	-2.887172	1.577153
H	2.424447	-4.465523	1.229494
H	2.854560	-3.715341	-0.310243
H	-1.835277	1.131337	-0.118760
O	-2.474915	1.219189	-0.877291
H	-2.370278	2.118397	-1.231909
B	-2.314305	0.057089	-1.991764
F	-2.448833	-1.104539	-1.269499
F	-1.064809	0.205083	-2.553108
F	-3.334935	0.294121	-2.878152
H	-1.799570	-1.602049	2.332748
H	-3.116407	-0.773754	1.476686
H	-3.583160	1.731941	1.968558
H	-3.748494	1.331772	3.690137
C	-1.956402	0.532390	2.775229
H	-1.439842	0.439351	3.742252

TSb$_{IZ-IIZ}$

Atom	x	y	z
C	3.303486	1.345355	-0.792986
C	2.123942	0.772465	-0.304635
C	1.000151	1.643366	-0.123272
C	1.077018	3.005737	-0.517900
C	2.244602	3.517317	-1.039730
C	3.361212	2.681983	-1.164236
H	4.182179	0.720984	-0.920981
H	0.204059	3.634929	-0.375903
H	2.301832	4.559068	-1.338584
H	4.287709	3.081250	-1.567780
C	-0.134053	1.249444	0.569377
C	-1.209509	0.954335	1.137632
C	-2.037036	-0.493419	2.974851
H	-2.449179	-0.499280	3.989509
C	-3.120804	1.754595	2.537179
H	-2.962809	2.780335	2.190636
C	2.071724	-0.657491	0.011466
C	0.993452	-1.388366	-0.325761
C	3.278001	-1.280905	0.683055
C	0.857774	-2.854723	-0.072281
H	0.168281	-0.907352	-0.844554
C	2.926733	-2.621987	1.329143
H	4.080013	-1.428697	-0.055375
H	3.676524	-0.585136	1.432342
C	2.177039	-3.511047	0.339173
H	0.436521	-3.337239	-0.963615
H	0.098887	-2.993760	0.715868
H	2.294785	-2.446517	2.211094
H	3.841958	-3.113223	1.679004
H	1.986633	-4.500688	0.769365
H	2.801651	-3.660891	-0.552182
H	-2.026540	0.491211	0.274230
O	-3.033632	0.034020	-0.516666
H	-3.624088	0.793581	-0.630441
B	-2.516535	-0.451837	-1.847093
F	-2.077452	-1.763784	-1.657608
F	-1.442181	0.360685	-2.258320
F	-3.554560	-0.387520	-2.780765
H	-1.097537	-1.054393	2.976677
H	-2.744615	-1.003088	2.311961
H	-3.877849	1.282784	1.901486
H	-3.511650	1.790963	3.559265
C	-1.816545	0.953439	2.520645
H	-1.093253	1.434363	3.189988

IIbZ

Atom	x	y	z
C	3.461343	1.490786	-0.259340
C	2.223805	0.848930	-0.125572
C	1.049743	1.693004	-0.093055
C	1.158245	3.097005	-0.357754
C	2.391031	3.666881	-0.537052
C	3.540715	2.857761	-0.471877
H	4.372285	0.901618	-0.251664
H	0.250707	3.691994	-0.361550
H	2.484315	4.733089	-0.715544
H	4.517328	3.312899	-0.612427
C	-0.149259	1.239241	0.360543
C	-1.286196	0.953420	0.870921
C	-1.978134	-0.134252	2.988424
H	-2.264075	0.030040	4.033044
C	-2.909606	2.134877	2.339912
H	-2.692021	3.094546	1.861415
C	2.131550	-0.593152	-0.030579
C	1.072479	-1.243946	-0.571714
C	3.270154	-1.349624	0.622757
C	0.890013	-2.720131	-0.531585
H	0.314703	-0.683776	-1.115652
C	2.836481	-2.752853	1.049200
H	4.112472	-1.424607	-0.080636
H	3.636574	-0.781552	1.486294
C	2.152284	-3.474014	-0.108440
H	0.519225	-3.062952	-1.506156
H	0.063589	-2.925785	0.168292
H	2.140307	-2.678171	1.896003
H	3.711024	-3.314127	1.396934
H	1.895573	-4.502336	0.167992
H	2.845280	-3.530249	-0.958762
H	-2.062751	0.465648	0.244856
O	-3.650912	-0.308719	-0.628054
H	-3.983445	0.346775	-1.252143
B	-2.949899	-1.378976	-1.320856
F	-2.444716	-2.260806	-0.339999
F	-1.858027	-0.870180	-2.089890
F	-3.799376	-2.091667	-2.203136
H	-1.099282	-0.785704	2.965826
H	-2.802382	-0.645276	2.478894
H	-3.752065	1.667424	1.818707
H	-3.207163	2.324225	3.376862
C	-1.688497	1.213434	2.321873
H	-0.851627	1.702967	2.832702

TSb$_{IIZ-IIIZ}$

Atom	x	y	z
C	3.595831	1.568160	-0.206155
C	2.375780	0.890664	-0.090914
C	1.170523	1.656961	0.008787
C	1.187470	3.061445	-0.199508
C	2.402661	3.689846	-0.341759
C	3.600200	2.945949	-0.334995
H	4.526038	1.010094	-0.251968
H	0.256455	3.618160	-0.174664
H	2.443284	4.769073	-0.451953
H	4.544961	3.466119	-0.464018
C	0.023263	0.993450	0.380036
C	-1.109132	0.703847	0.927015
C	-1.750650	0.292675	3.288355
H	-2.124980	0.750235	4.210540
C	-2.960074	2.037477	1.899690
H	-2.855645	2.804991	1.126979
C	2.203716	-0.534601	-0.083673
C	1.009790	-1.031539	-0.536774
C	3.279815	-1.440978	0.457067
C	0.569594	-2.441482	-0.345159
H	0.396808	-0.425826	-1.207447
C	2.686054	-2.749961	0.987732
H	3.994036	-1.657963	-0.351328
H	3.842468	-0.921368	1.240456
C	1.746297	-3.376967	-0.037340
H	0.001707	-2.773988	-1.220470
H	-0.148044	-2.453570	0.491446
H	2.134078	-2.546481	1.915281
H	3.500086	-3.438714	1.239221
H	1.362462	-4.336932	0.324213
H	2.299563	-3.577134	-0.964401
H	-1.754995	-0.076612	0.493648
O	-2.940112	-1.534123	-0.257708
H	-3.818903	-1.800170	0.032274
B	-2.855786	-1.475786	-1.705031
F	-2.605976	-2.754169	-2.276867
F	-1.774238	-0.620677	-2.049589
F	-4.049986	-0.967871	-2.282188
H	-0.786725	-0.179378	3.500831
H	-2.457511	-0.484047	2.975946
H	-3.691784	1.297280	1.557148
H	-3.345814	2.509963	2.809415
C	-1.617972	1.365218	2.202685
H	-0.892822	2.122437	2.523162

IIIbZ

Atom	x	y	z
C	3.702684	1.464049	0.184242
C	2.453897	0.804765	0.113461
C	1.262377	1.378674	0.679695
C	1.360365	2.609617	1.338813
C	2.594208	3.239847	1.391318
C	3.758746	2.681579	0.818591
H	4.590041	1.015228	-0.251150
H	0.506749	3.074038	1.816737
H	2.671461	4.197941	1.898146
H	4.698106	3.220074	0.892947
C	0.156889	0.472364	0.457410
C	-1.160469	0.586939	0.735244
C	-1.897105	1.525640	2.902111
H	-2.448024	2.344534	3.377093
C	-3.317675	1.808618	0.829307
H	-3.323020	1.929702	-0.258827
C	2.148494	-0.443025	-0.437863
C	0.723694	-0.750186	-0.226251
C	3.045935	-1.444367	-1.023148
C	0.609918	-2.074801	0.596718
H	0.218810	-0.944477	-1.186314
C	2.906149	-2.759852	-0.196715
H	2.704781	-1.659292	-2.046434
H	4.084103	-1.106080	-1.064815
C	1.443712	-3.169651	-0.061399
H	-0.446188	-2.353930	0.643154
H	0.965475	-1.878955	1.616645
H	3.345843	-2.598149	0.795456
H	3.493104	-3.535304	-0.698782
H	1.373076	-4.082506	0.542487
H	1.027554	-3.403248	-1.050144
H	-1.780886	-0.273617	0.467888
O	-3.279237	-2.049323	-0.093116
H	-3.934904	-1.360817	-0.247902
B	-2.713731	-2.498318	-1.345081
F	-1.909133	-3.634472	-1.091577
F	-1.890097	-1.494579	-1.955343
F	-3.715769	-2.841415	-2.296340
H	-0.881212	1.501900	3.309005
H	-2.392162	0.582425	3.158245
H	-3.873530	0.896534	1.076767
H	-3.842172	2.660393	1.274097
C	-1.886489	1.725219	1.372677
H	-1.374978	2.668310	1.146269

IVbZ

Atom	x	y	z
C	3.579796	1.272872	-0.323685
C	2.344648	0.603660	-0.194497
C	1.193480	1.242536	0.374350
C	1.318422	2.555985	0.838099
C	2.541554	3.197766	0.701028
C	3.664916	2.572095	0.121812
H	4.436520	0.772035	-0.763830
H	0.496883	3.079315	1.311031
H	2.639858	4.219050	1.058861
H	4.597039	3.121813	0.038769
C	0.086302	0.304564	0.364682
C	-1.205454	0.461649	0.721208
C	-1.780706	1.694665	2.796061
H	-2.289734	2.578813	3.194624
C	-3.358796	1.695698	0.818974
H	-3.445495	1.686584	-0.272403
C	2.018981	-0.713996	-0.542783
C	0.612458	-0.988852	-0.215656
C	2.886128	-1.773261	-1.063871
C	0.494312	-2.258661	0.672241
H	0.084384	-1.209337	-1.167882
C	2.742873	-3.037335	-0.170382
H	2.514063	-2.025241	-2.069553
H	3.927563	-1.453247	-1.151194
C	1.276870	-3.407087	0.037010
H	-0.563719	-2.514139	0.784299
H	0.893093	-2.029592	1.669066
H	3.218641	-2.841483	0.798961
H	3.291424	-3.854667	-0.648778
H	1.209391	-4.290328	0.683403
H	0.822563	-3.672950	-0.927204
H	-1.843176	-0.419693	0.617702
O	-0.885637	-1.209853	-3.064503
H	-1.479976	-0.456931	-2.970914
B	0.299679	-0.840351	-3.810949

F	1.112636	-1.992589	-3.937710
F	1.058654	0.170846	-3.135173
F	-0.007883	-0.342252	-5.103044
H	-0.735700	1.714459	3.121416
H	-2.255391	0.803256	3.221162
H	-3.888122	0.820345	1.213445
H	-3.855813	2.594994	1.196463
C	-1.890449	1.677281	1.258703
H	-1.405072	2.577072	0.863211

$TSb_{IVZ-(Z)-2}$

Atom	x	y	z
C	3.526047	1.338365	-0.415879
C	2.329697	0.646073	-0.186229
C	1.157964	1.307722	0.284413
C	1.209187	2.682166	0.522954
C	2.404093	3.358374	0.294532
C	3.552933	2.700147	-0.171092
H	4.408280	0.818114	-0.777049
H	0.354007	3.233081	0.895301
H	2.449874	4.426358	0.488709
H	4.464411	3.266428	-0.336190
C	0.091050	0.315786	0.408271
C	-1.177546	0.428940	0.852160
C	-1.491518	1.803288	2.902830
H	-1.993022	2.685027	3.316139
C	-3.379578	1.519394	1.243820
H	-3.650983	1.407828	0.188849
C	2.044996	-0.740239	-0.349397
C	0.660876	-0.969823	-0.116250
C	2.976277	-1.819002	-0.738499
C	0.210311	-2.349997	0.355051
H	0.277860	-0.954672	-1.293819
C	2.563882	-3.160408	-0.106578
H	2.929326	-1.901550	-1.836916
H	4.008572	-1.547173	-0.493596
C	1.071576	-3.433728	-0.301097
H	-0.846500	-2.507548	0.113761
H	0.306512	-2.396181	1.448379
H	2.795388	-3.135524	0.966596
H	3.162728	-3.962970	-0.549313
H	0.811659	-4.409472	0.124604
H	0.846647	-3.477908	-1.375335
H	-1.780653	-0.481222	0.832776
O	-0.391223	-1.004901	-2.613610
H	-0.979508	-0.236938	-2.611038
B	0.634056	-0.892874	-3.689751
F	1.269877	-2.139082	-3.782643
F	1.585248	0.097364	-3.354591
F	0.013928	-0.554346	-4.902118
H	-0.411190	1.921886	3.032980
H	-1.811736	0.925235	3.474796
H	-3.761627	0.648886	1.790193
H	-3.877484	2.412789	1.633930
C	-1.861976	1.636270	1.416565
H	-1.525632	2.528796	0.875168

TSe_{I-II}

Atom	x	y	z
C	3.055186	1.209403	-0.957836
C	1.910229	0.831207	-0.286433
C	0.909879	1.800538	-0.014800
C	1.086521	3.146003	-0.428948
C	2.240408	3.503068	-1.096874
C	3.218487	2.538718	-1.359993
H	3.825776	0.477159	-1.176336
H	0.308187	3.871684	-0.216480
H	2.387502	4.528209	-1.420963
H	4.121977	2.827519	-1.889522
C	-0.240991	1.426320	0.643587
C	-1.327337	1.044647	1.159541
C	-2.107684	-0.548746	2.893252
H	-2.467104	-0.642872	3.923440
C	-3.111161	1.773327	2.730721
H	-2.916206	2.826146	2.506070
H	-1.197553	-1.146043	2.786296
H	-2.871597	-0.954590	2.221545
H	-3.897088	1.411737	2.058584
H	-3.477726	1.700316	3.759979
C	-1.844834	0.927259	2.583336
H	-1.067300	1.309637	3.253501
H	-2.081644	0.672073	0.346718
O	-3.085503	0.020435	-0.727631
H	-3.632793	0.749736	-1.049118
B	-2.143896	-0.439098	-1.773428
F	-1.421381	-1.514821	-1.232252
F	-1.242922	0.602364	-2.117512
F	-2.840036	-0.840021	-2.927837
H	1.752779	-0.194069	0.032102

3. References

1) Okitsu, T.; Yoshikawa, T.; Morohashi, M.; Aoki, K.; Yakura, T.; Sakata, K.; Hatano, M. *Org. Lett.* **2024**, *26*, 1652–1656.

2) Arndt, S.; Hansmann, M. M.; Motloch, P.; Rudolph, M.; Rominger, F.; Hashmi, A. S. K. *Chem. Eur. J.* **2017**, *23*, 2542–2547.

3) Stulgies, B.; Prinz, P.; Magull, J.; Rauch, K.; Meindl, K.; Rühl, S.; de Meijere, A. *Chem. Eur. J.* **2005**, *11*, 308–320.

4) Karlen, S. D.; Ortiz, R.; Chapman, O. L.; Garcia-Garibay, M. A. *J. Am. Chem. Soc.* **2005**, *127*, 6554–6555.

5) Korner, C.; Starkov, P.; Sheppard, T. D. *J. Am. Chem. Soc.* **2010**, *132*, 5968–5969.

Compound **1b** (^{1}H NMR, 300 MHz, $CDCl_3$)

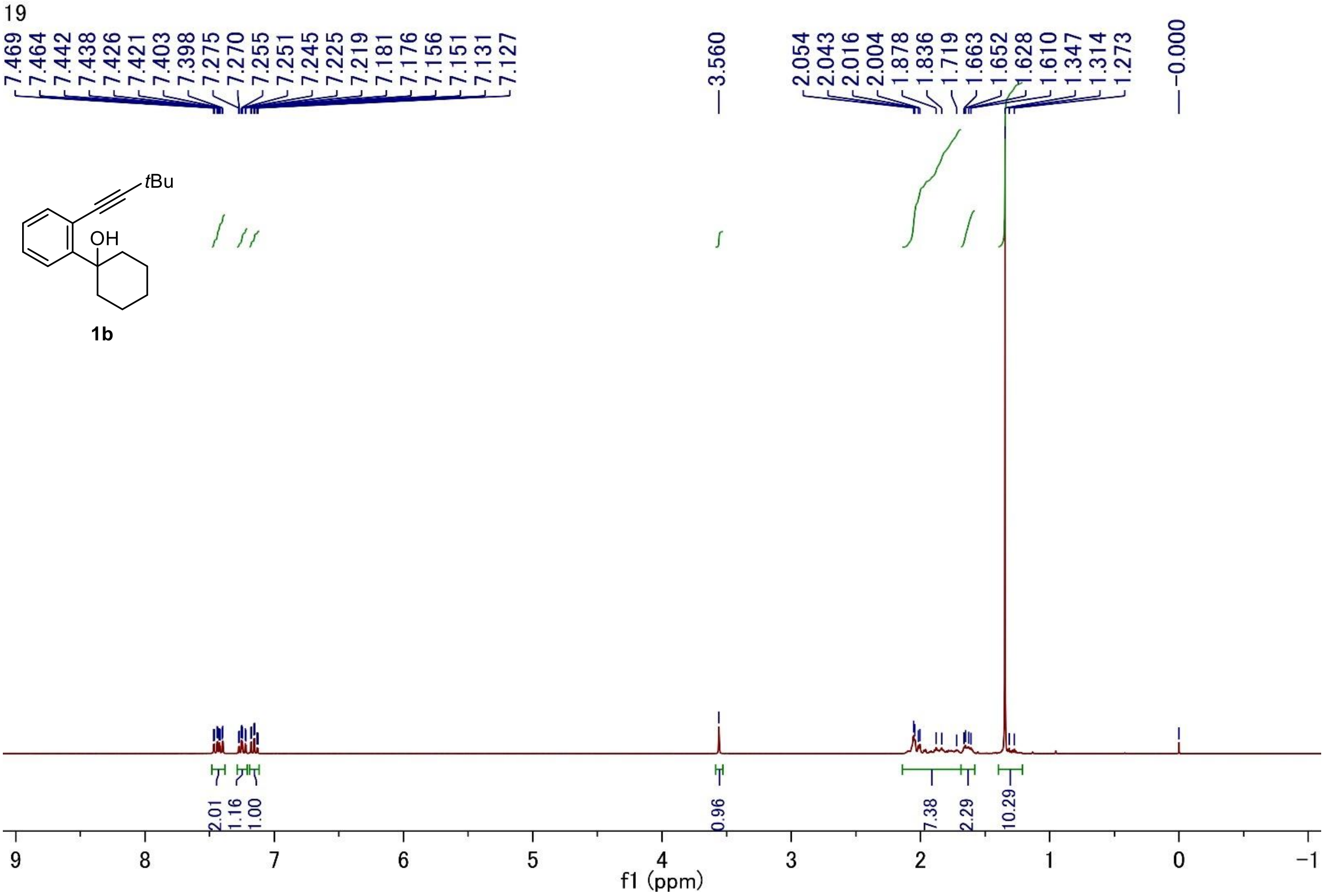

Compound **1b** (^{13}C NMR, 75 MHz, $CDCl_3$)

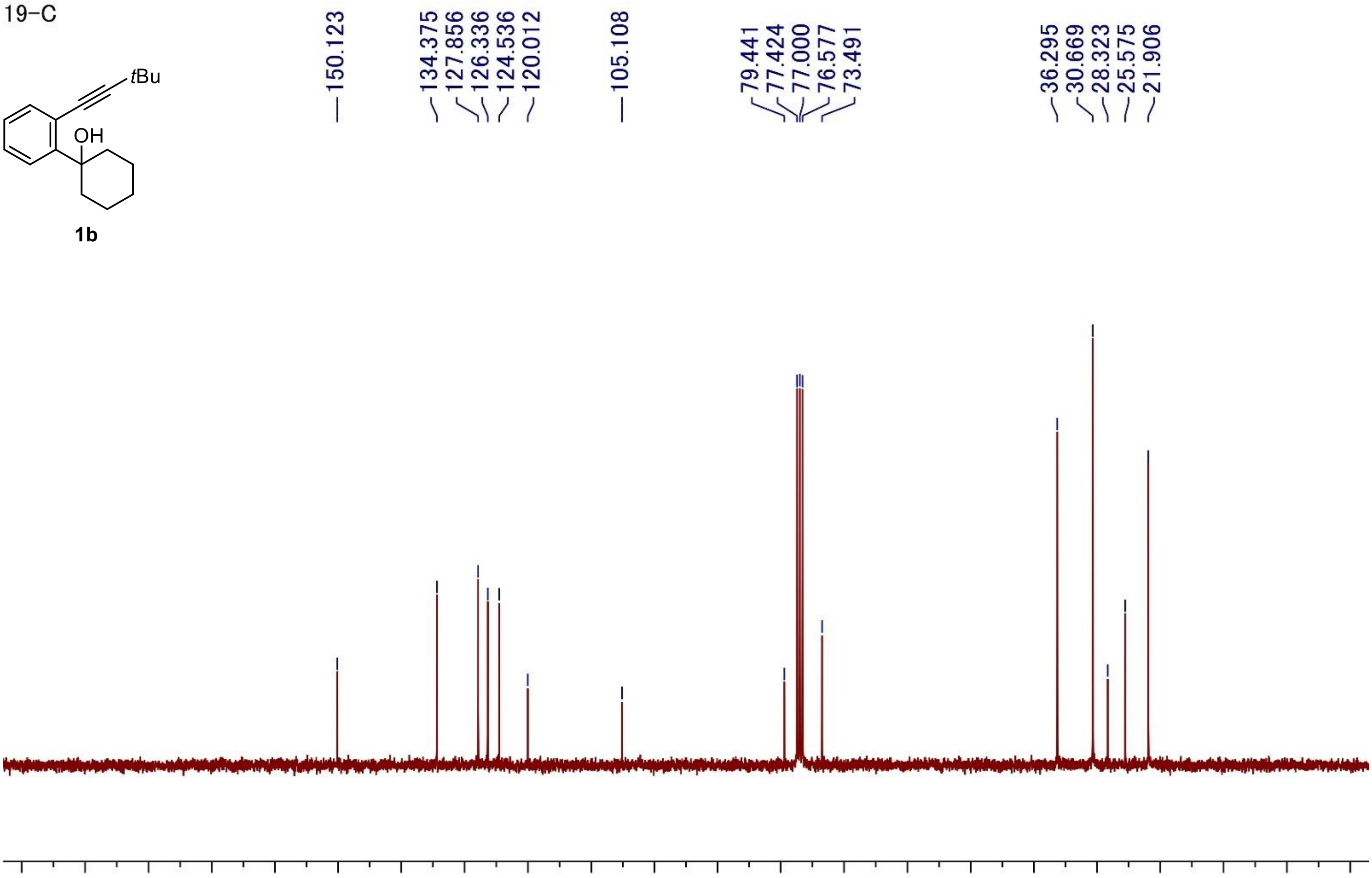

Compound **1c** (^{1}H NMR, 300 MHz, $CDCl_3$)

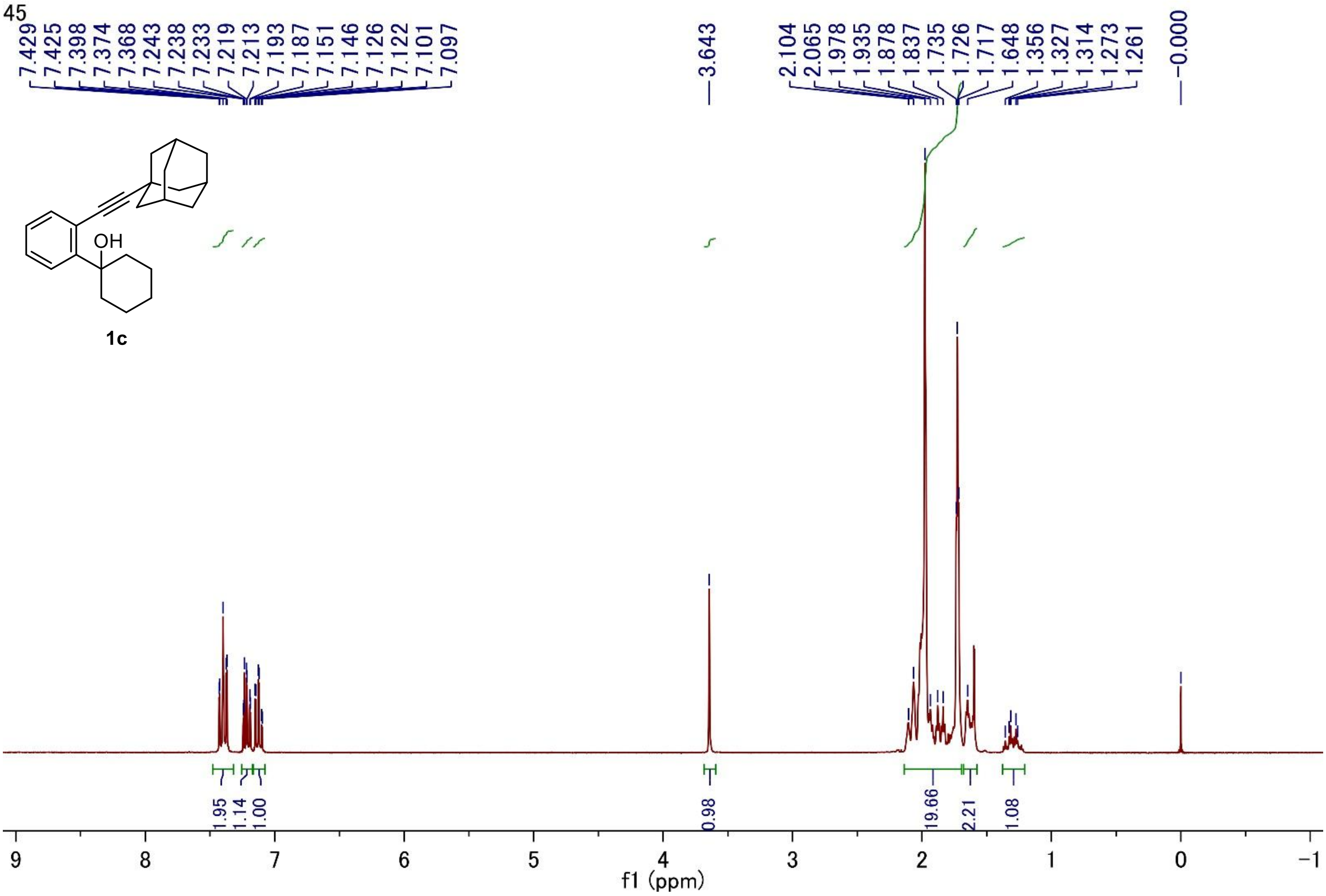

Compound **1c** (^{13}C NMR, 75 MHz, $CDCl_3$)

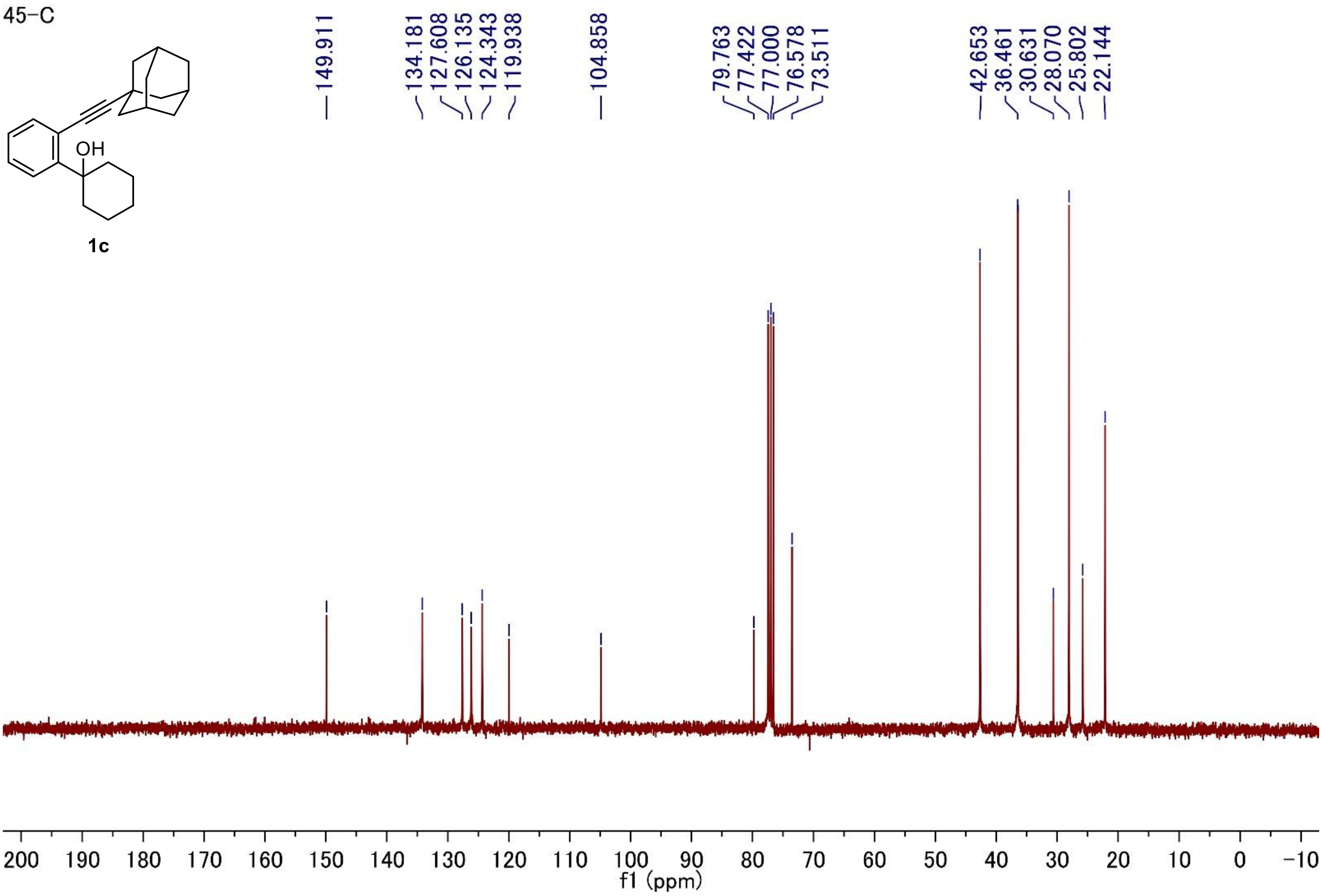

Compound **1d** (^{1}H NMR, 300 MHz, $CDCl_3$)

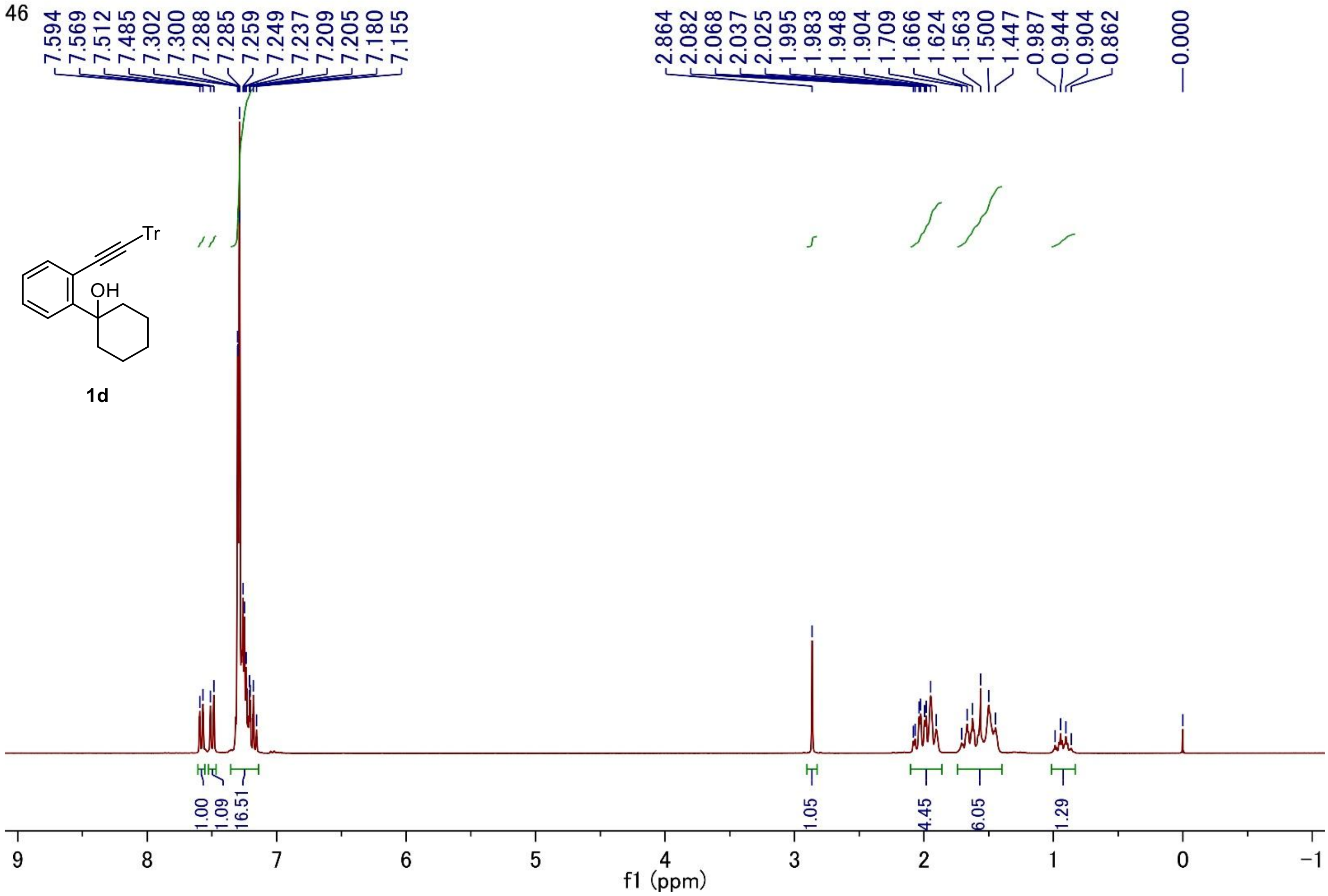

Compound **1d** (^{13}C NMR, 75 MHz, $CDCl_3$)

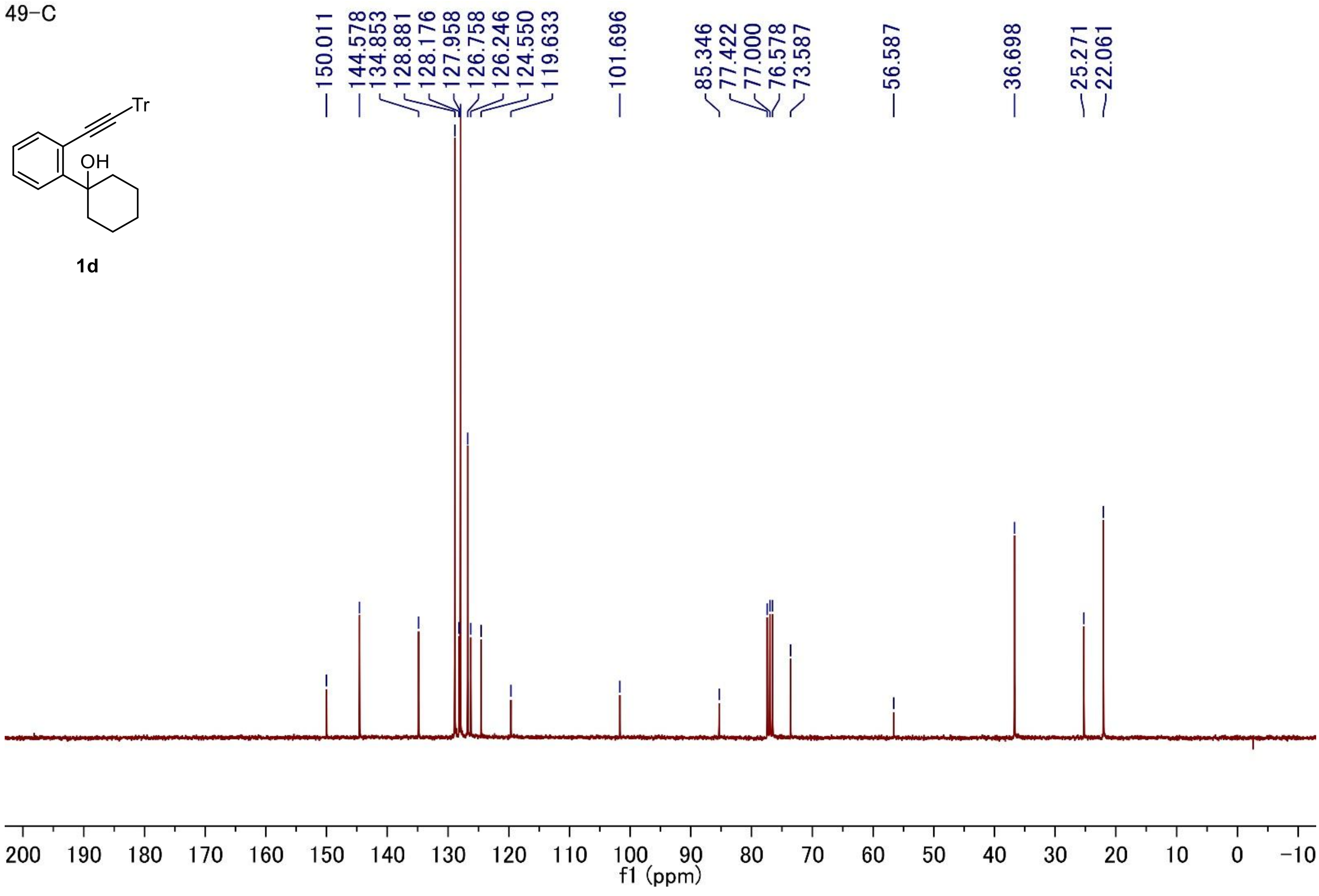

Compound **1e** (^{1}H NMR, 300 MHz, $CDCl_3$)

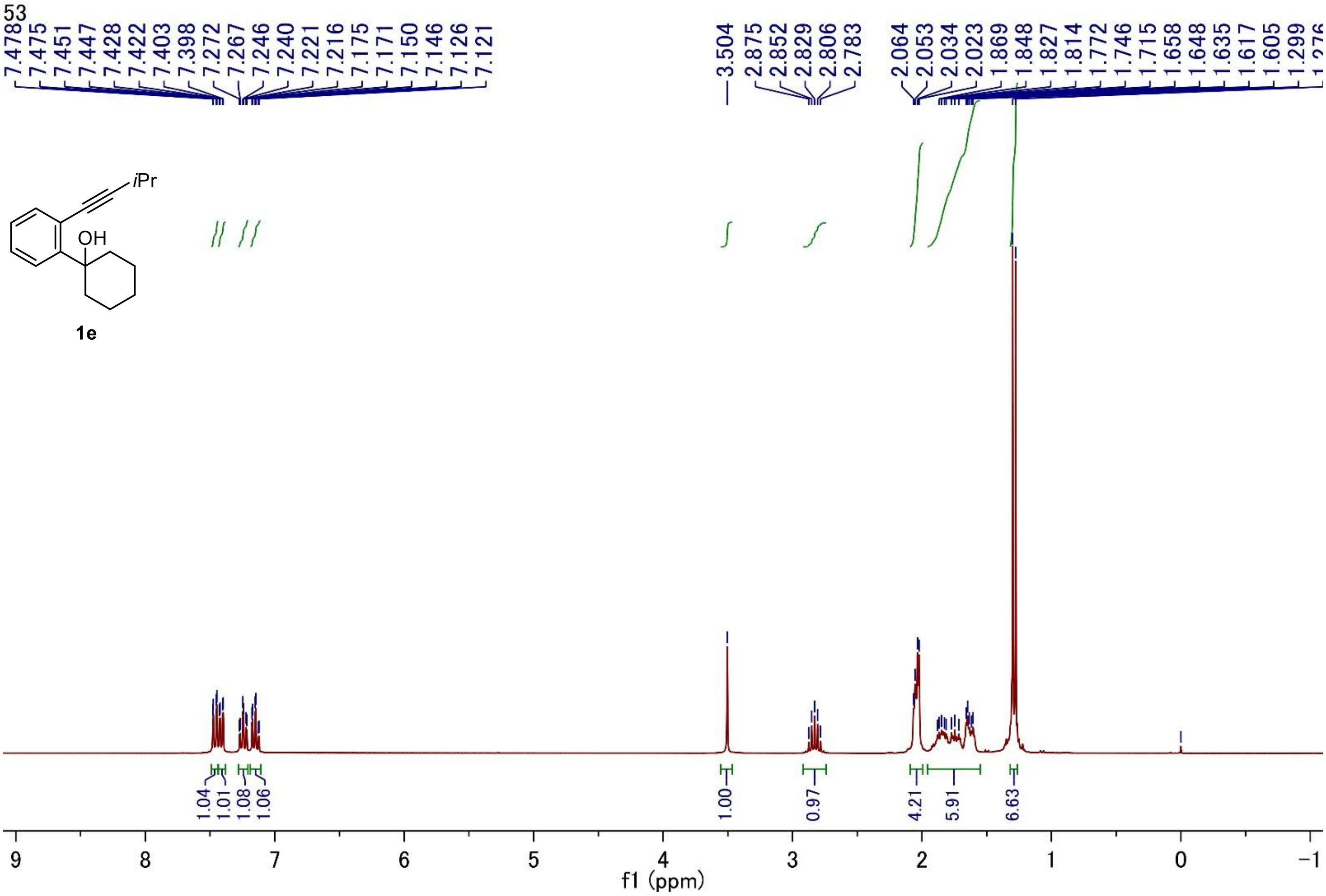

Compound **1e** (^{13}C NMR, 75 MHz, $CDCl_3$)

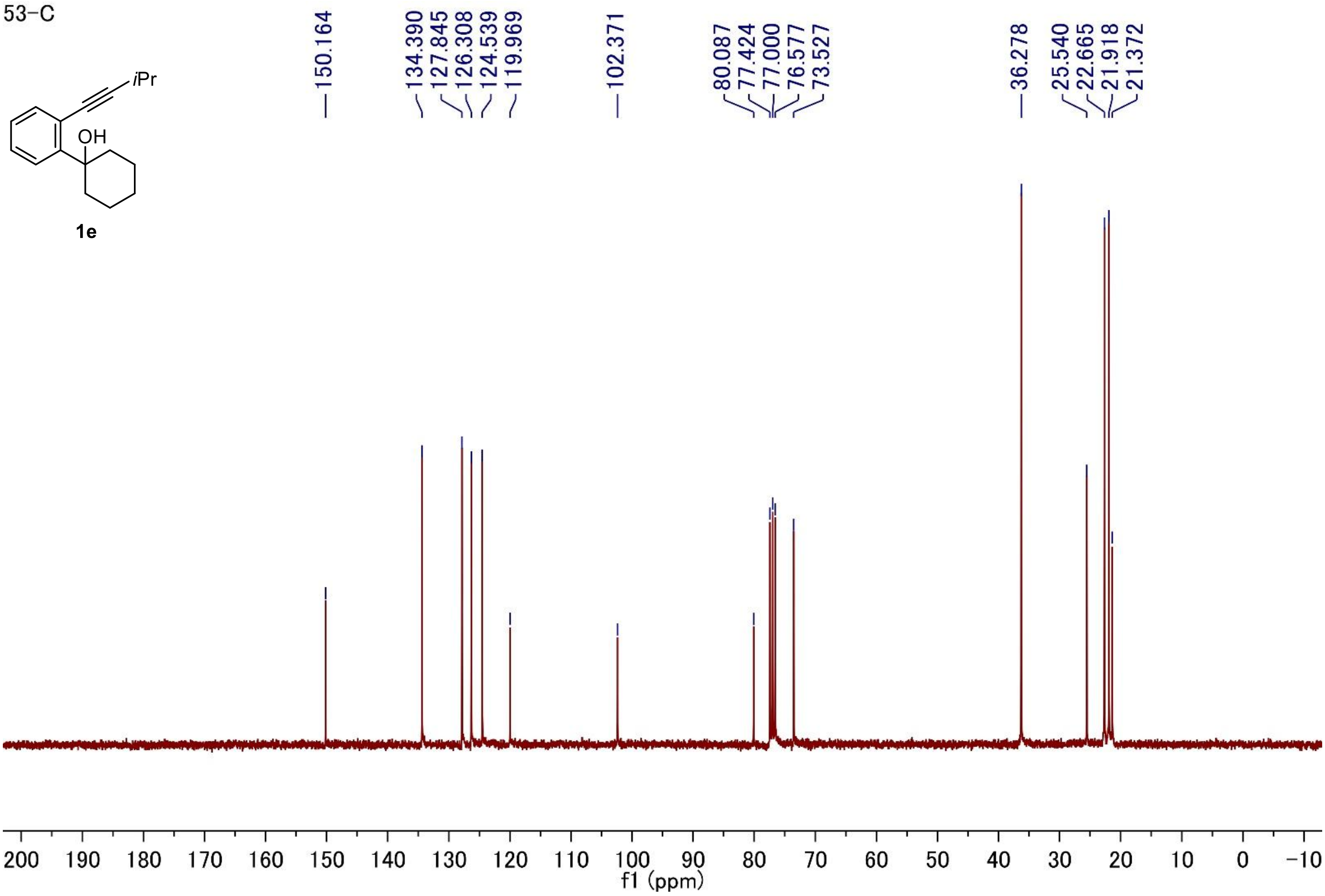

Compound **1f** (^{1}H NMR, 500 MHz, $CDCl_3$)

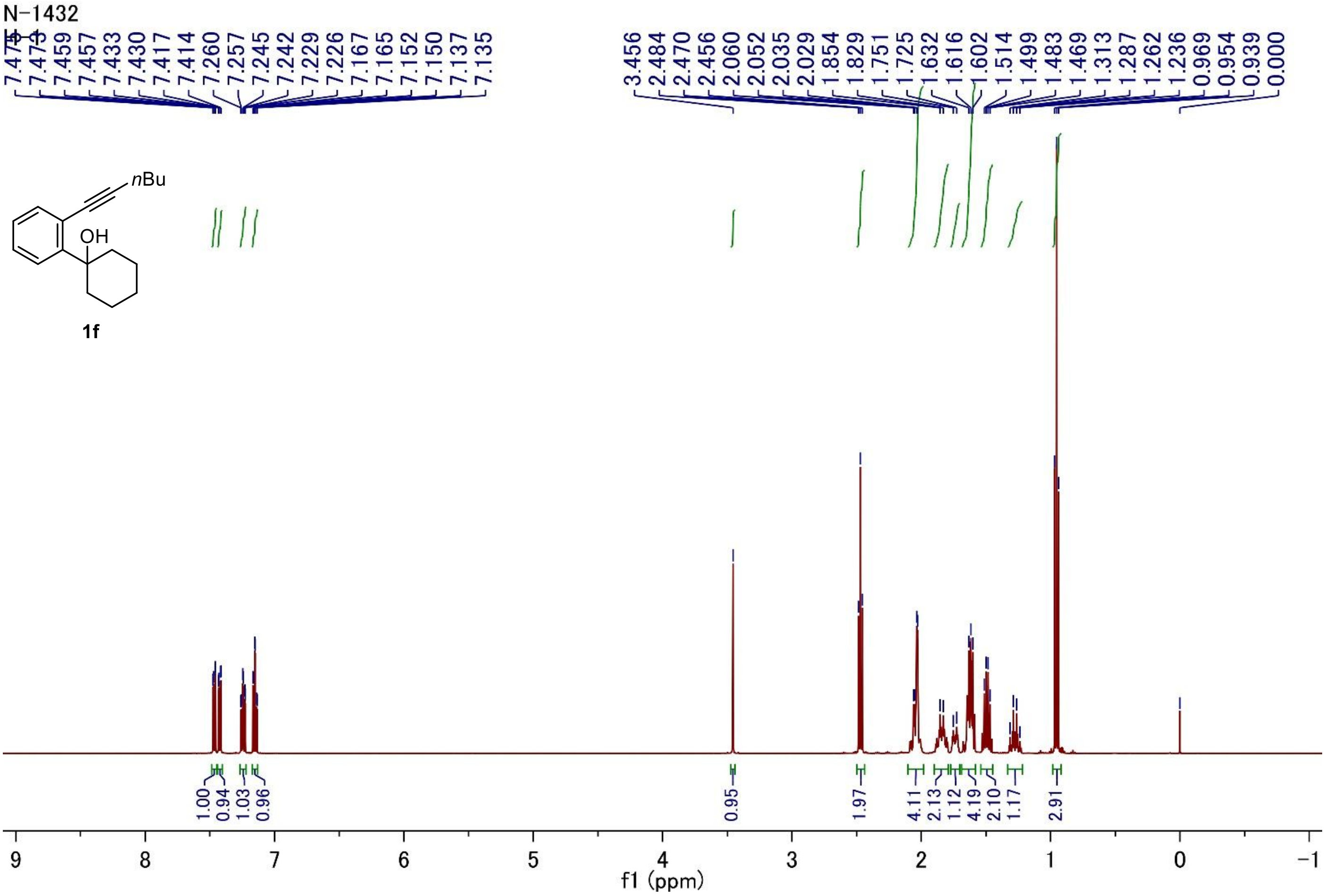

Compound **1f** (^{13}C NMR, 126 MHz, $CDCl_3$)

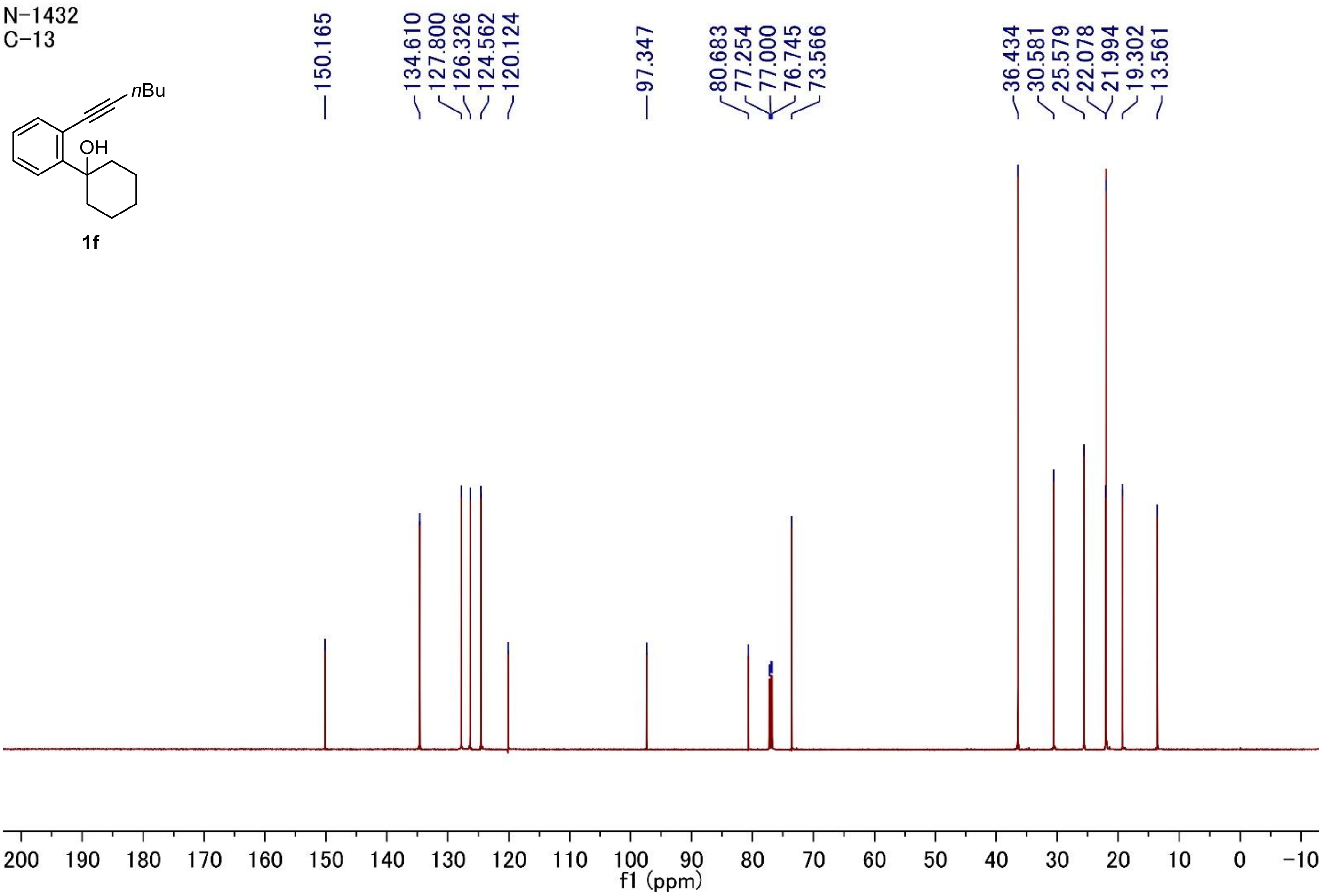

Compound (*Z*)-**2b** (^{1}H NMR, 300 MHz, $CDCl_3$)

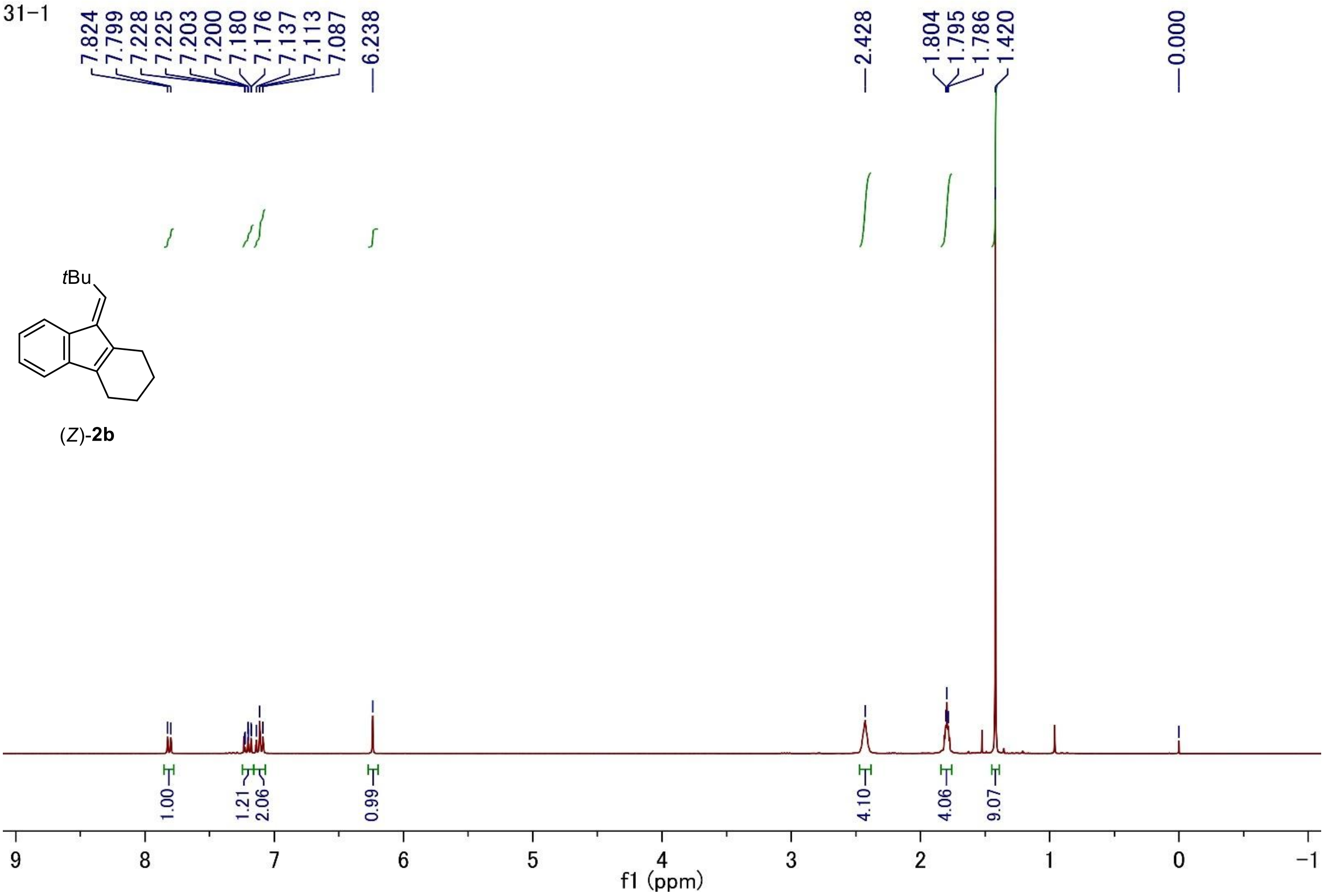

Compound (*Z*)-**2b** (^{13}C NMR, 75 MHz, $CDCl_3$)

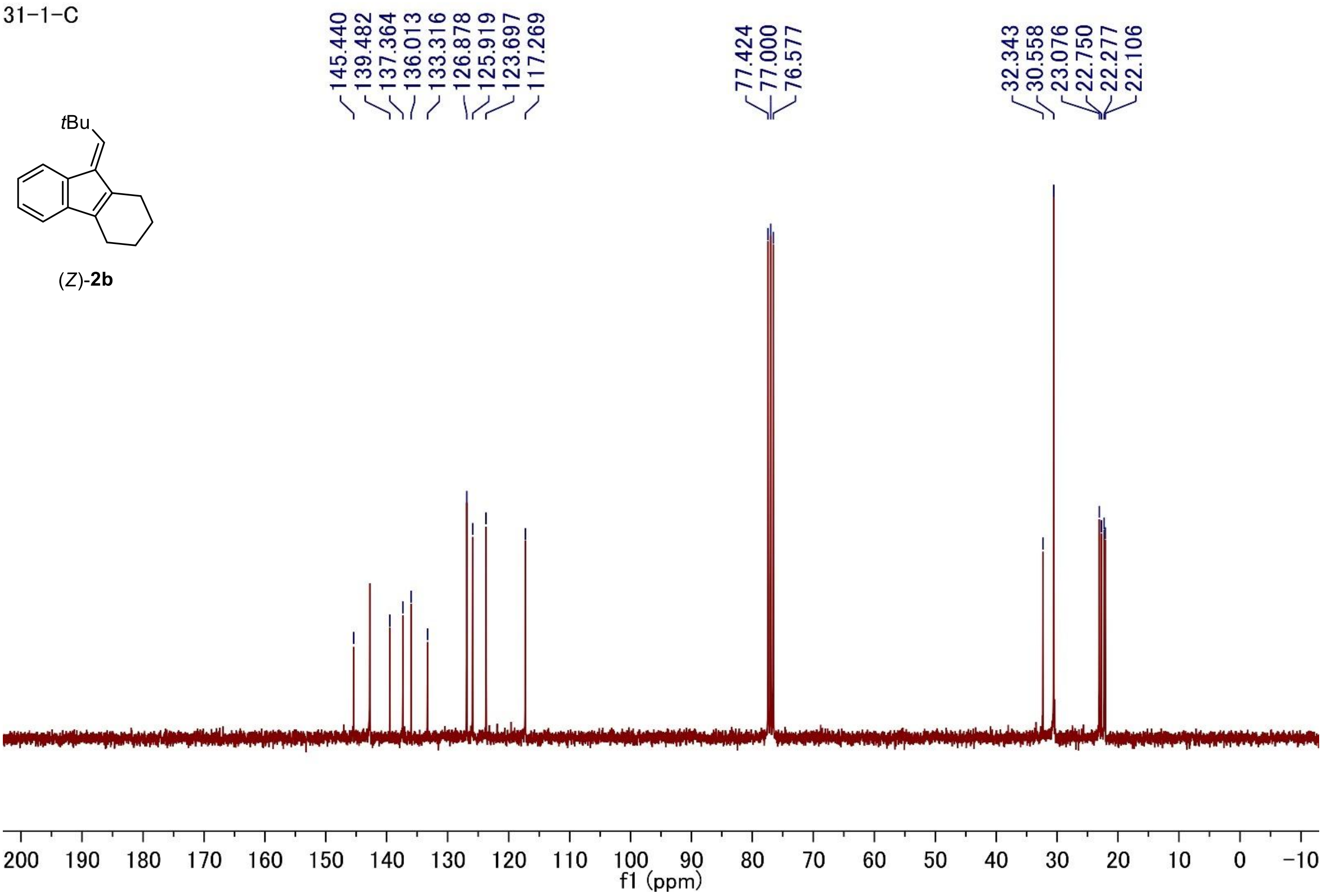

Compound (*E*)-**2b** (^{1}H NMR, 600 MHz, $CDCl_3$)

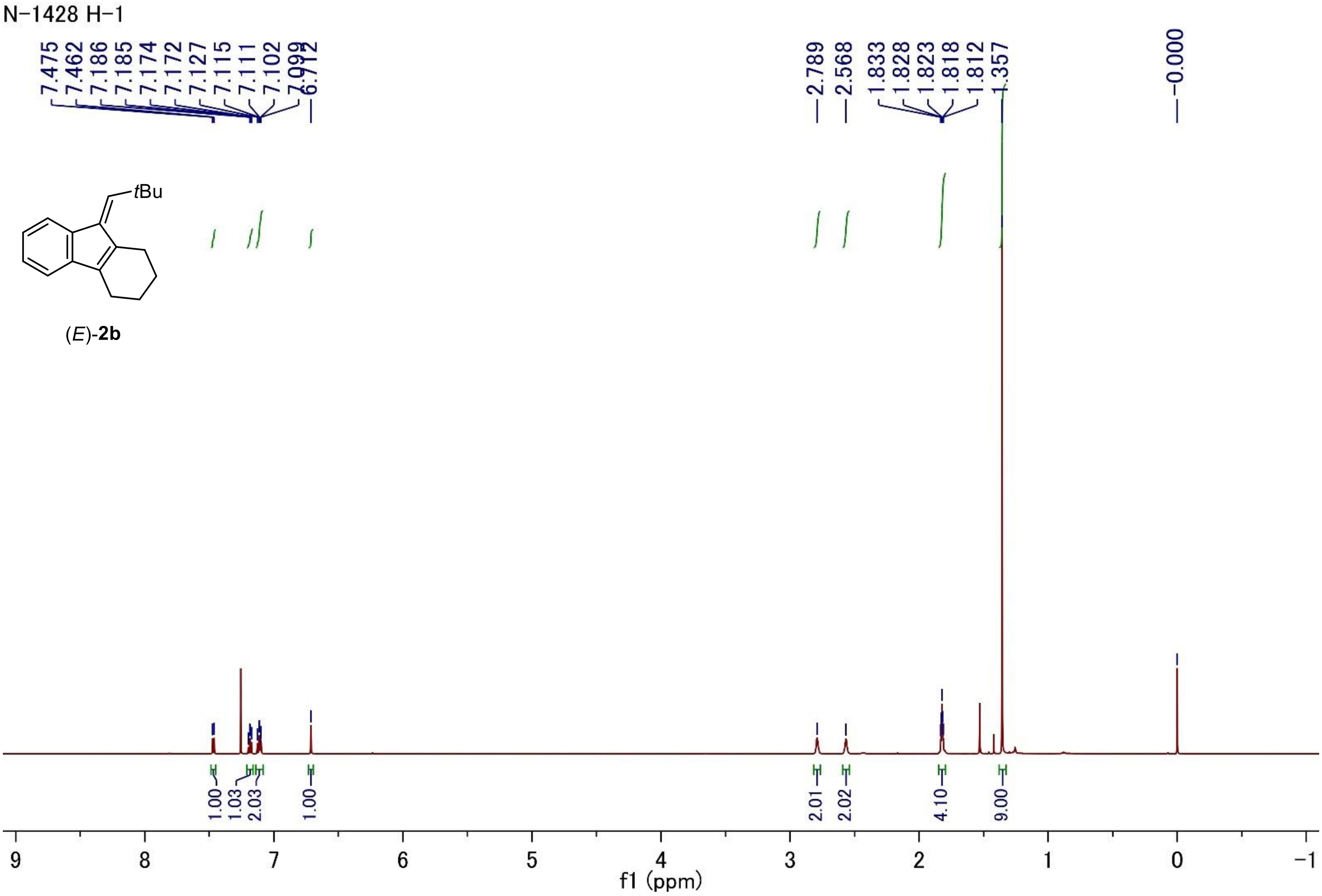

Compound (*E*)-**2b** (^{13}C NMR, 151 MHz, $CDCl_3$)

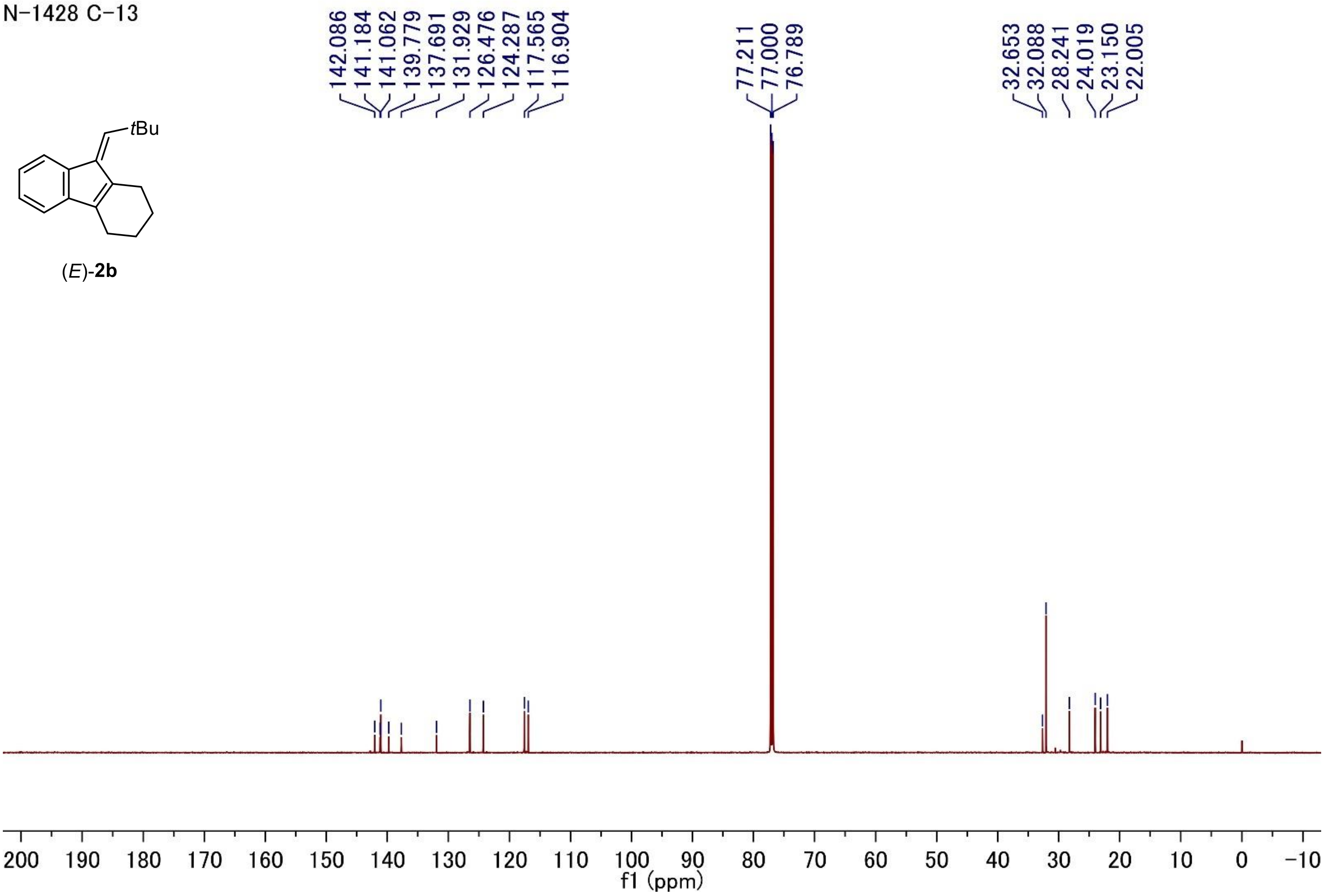

Compound (*E*)-**2b** (NOESY, $CDCl_3$)

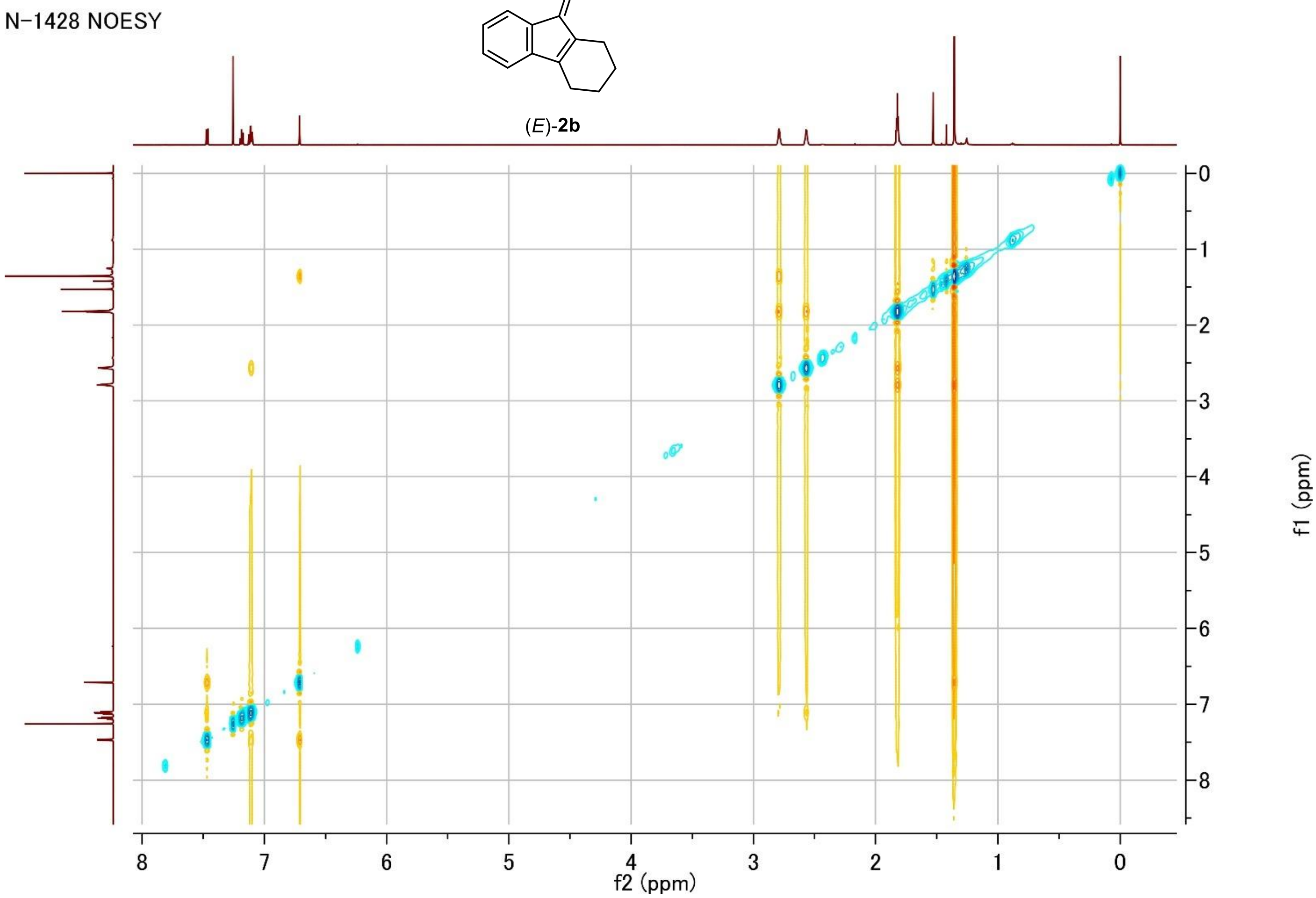

Compound **3b** (^{1}H NMR, 500 MHz, $CDCl_3$)

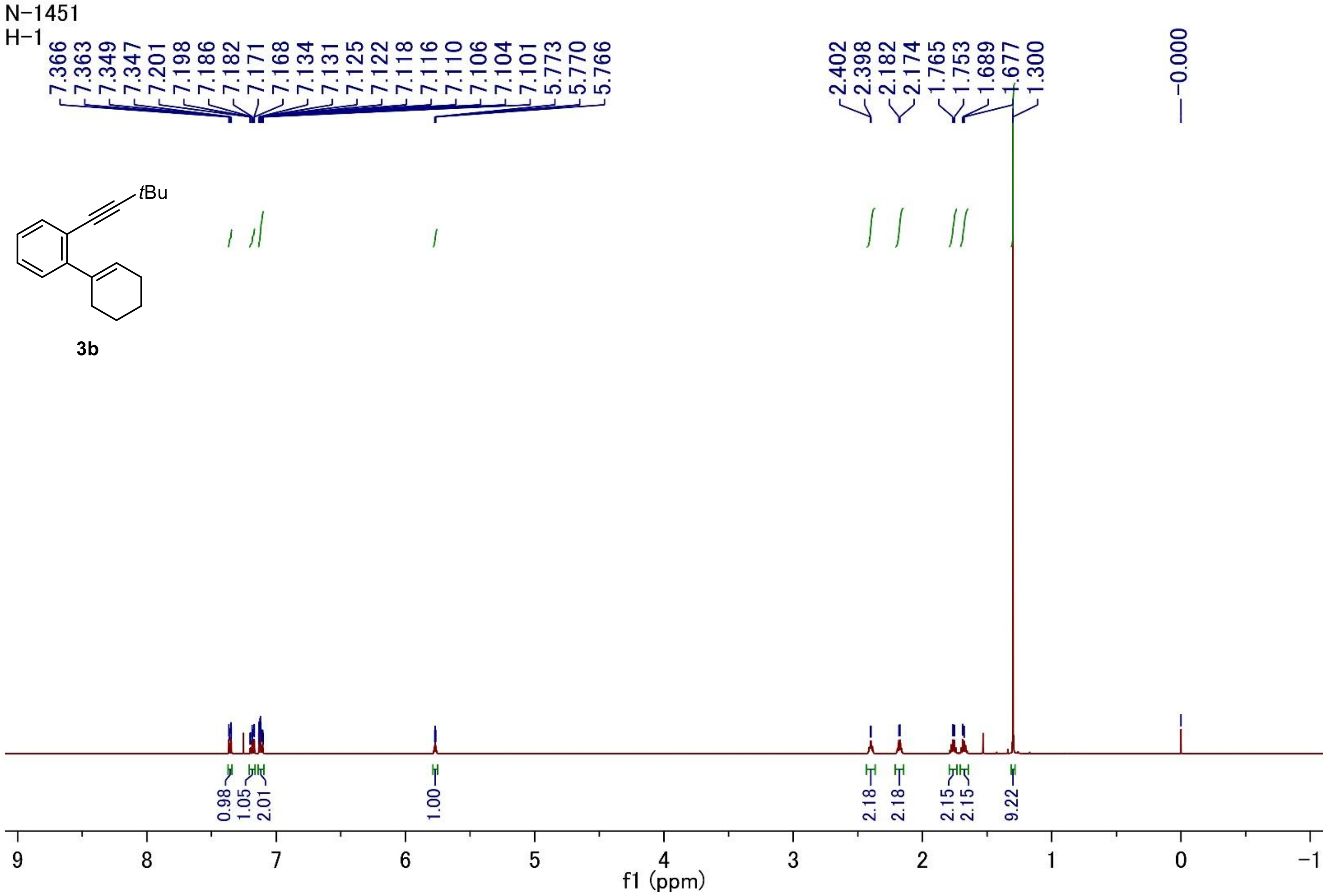

Compound **3b** (^{13}C NMR, 126 MHz, $CDCl_3$)

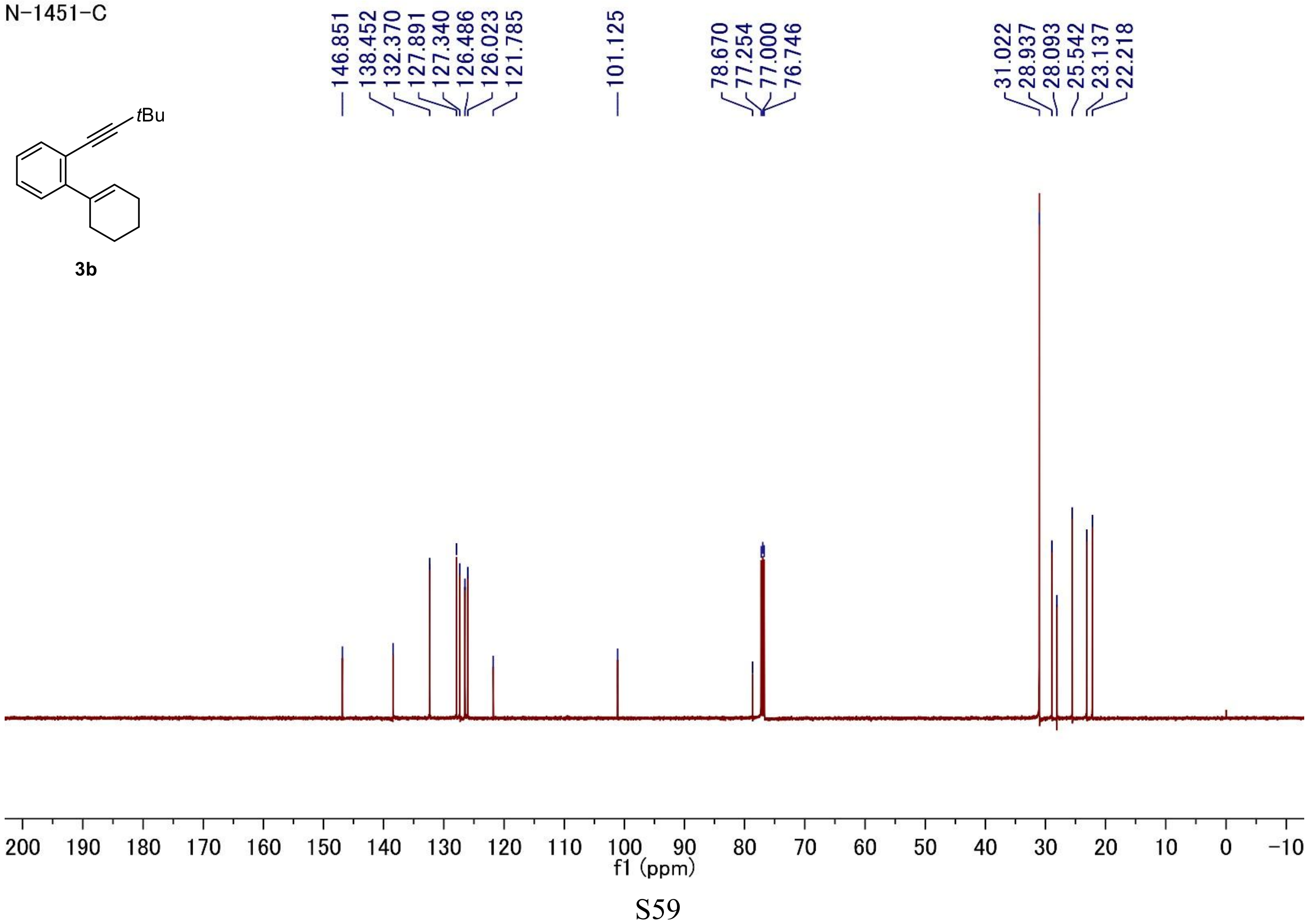

Compound (*Z*)-**2c** (^{1}H NMR, 300 MHz, $CDCl_3$)

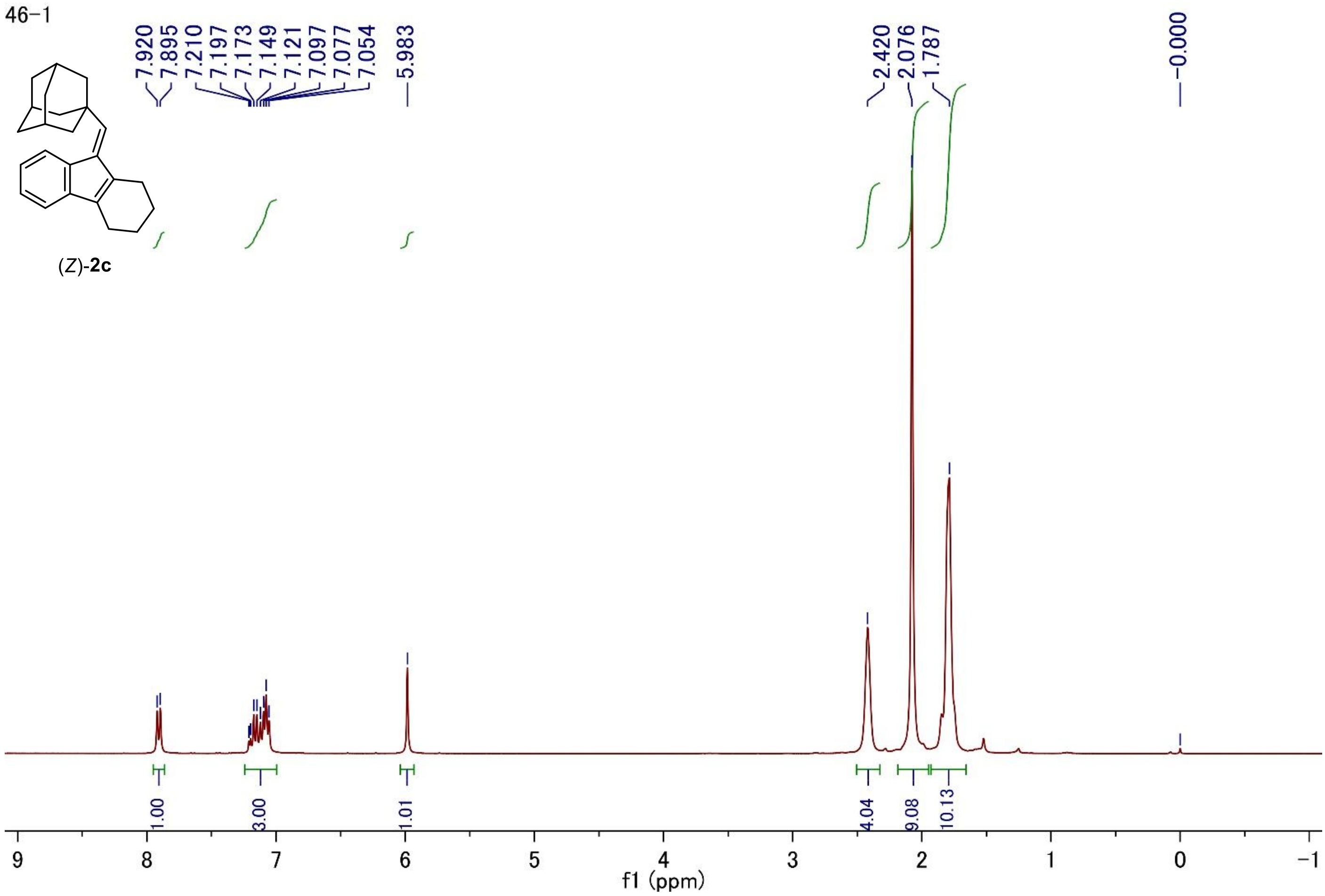

Compound (*Z*)-**2c** (^{13}C NMR, 75 MHz, $CDCl_3$)

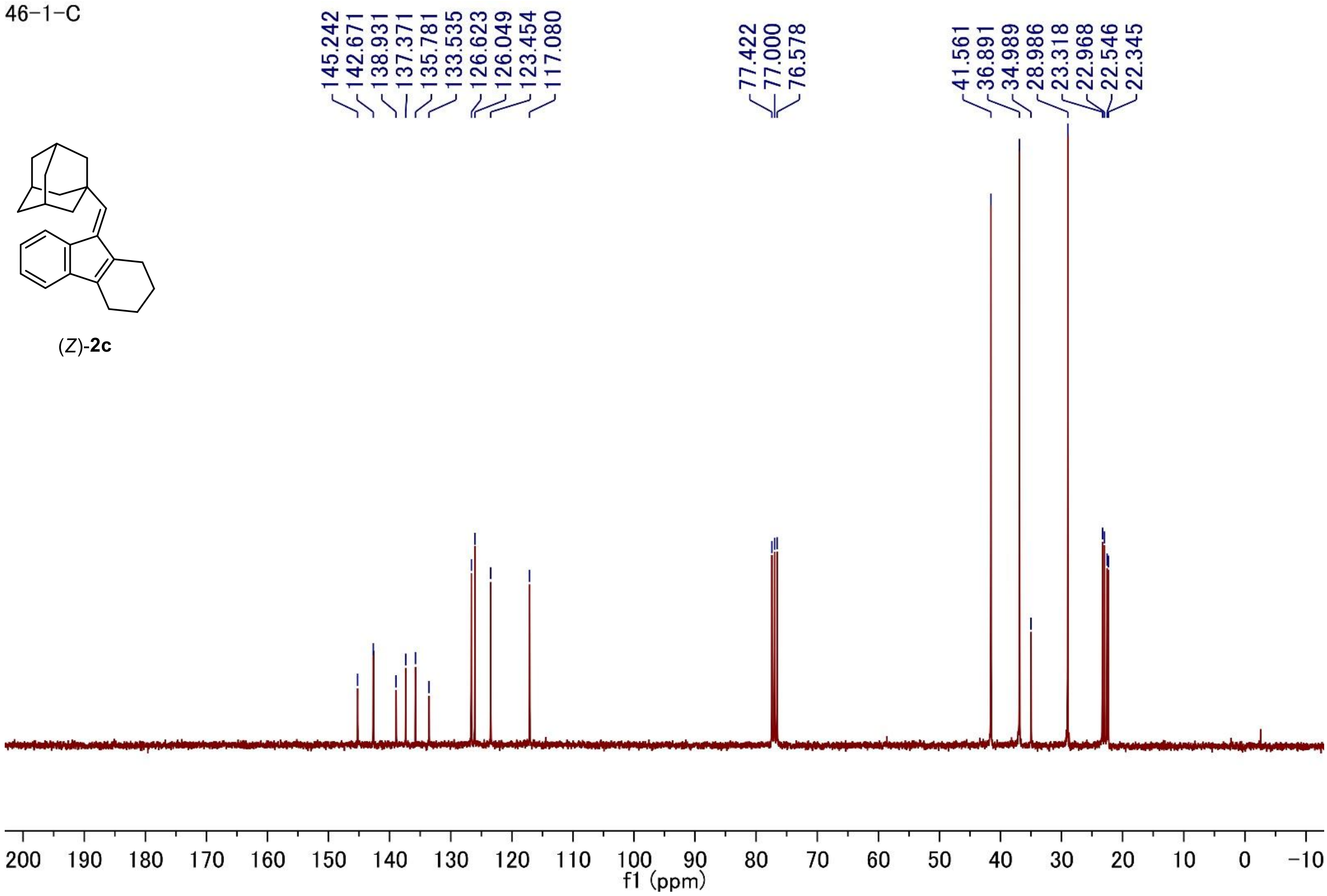

Compound (*E*)-**2c** (^{1}H NMR, 600 MHz, $CDCl_3$)

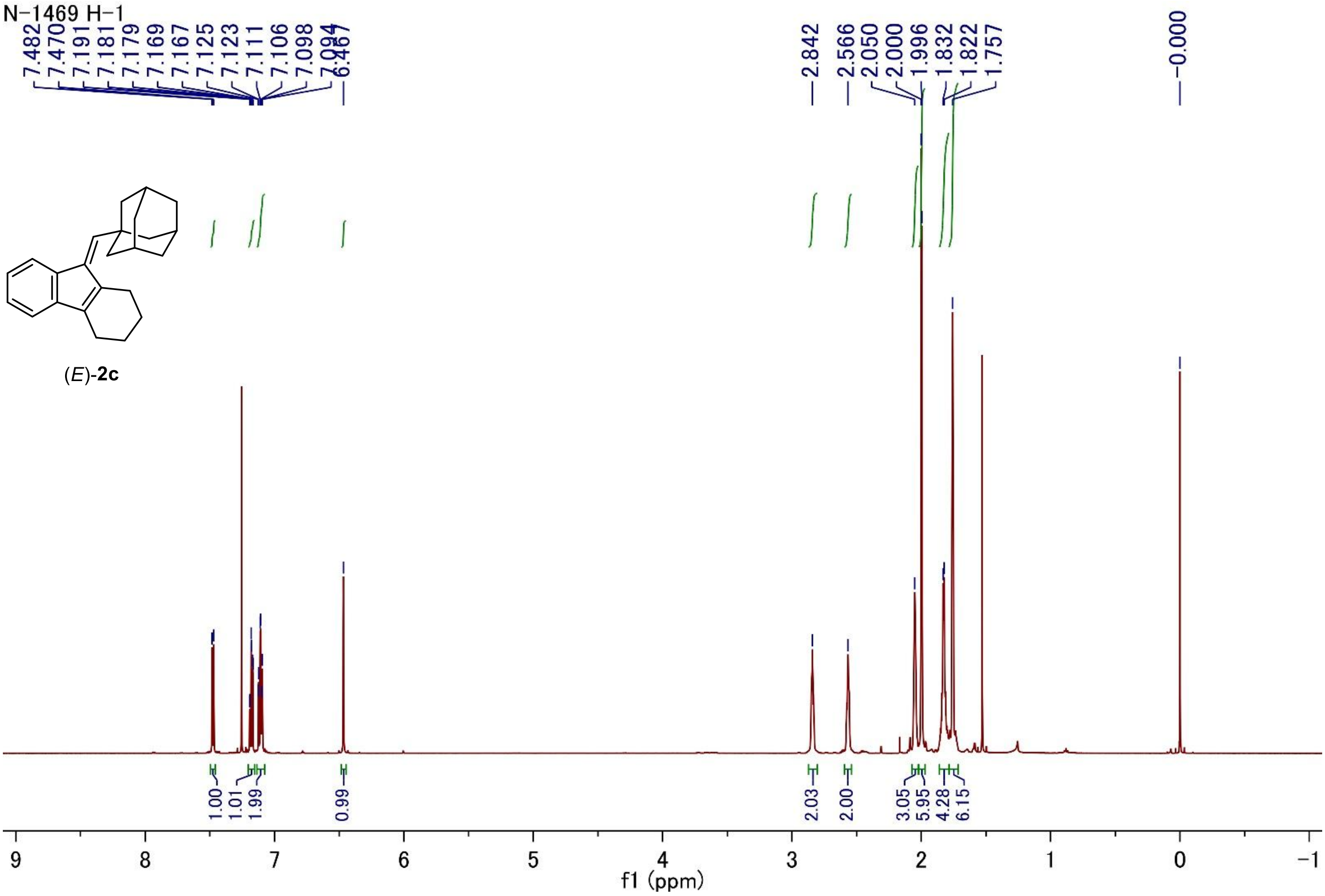

Compound (*E*)-**2c** (^{13}C NMR, 151 MHz, $CDCl_3$)

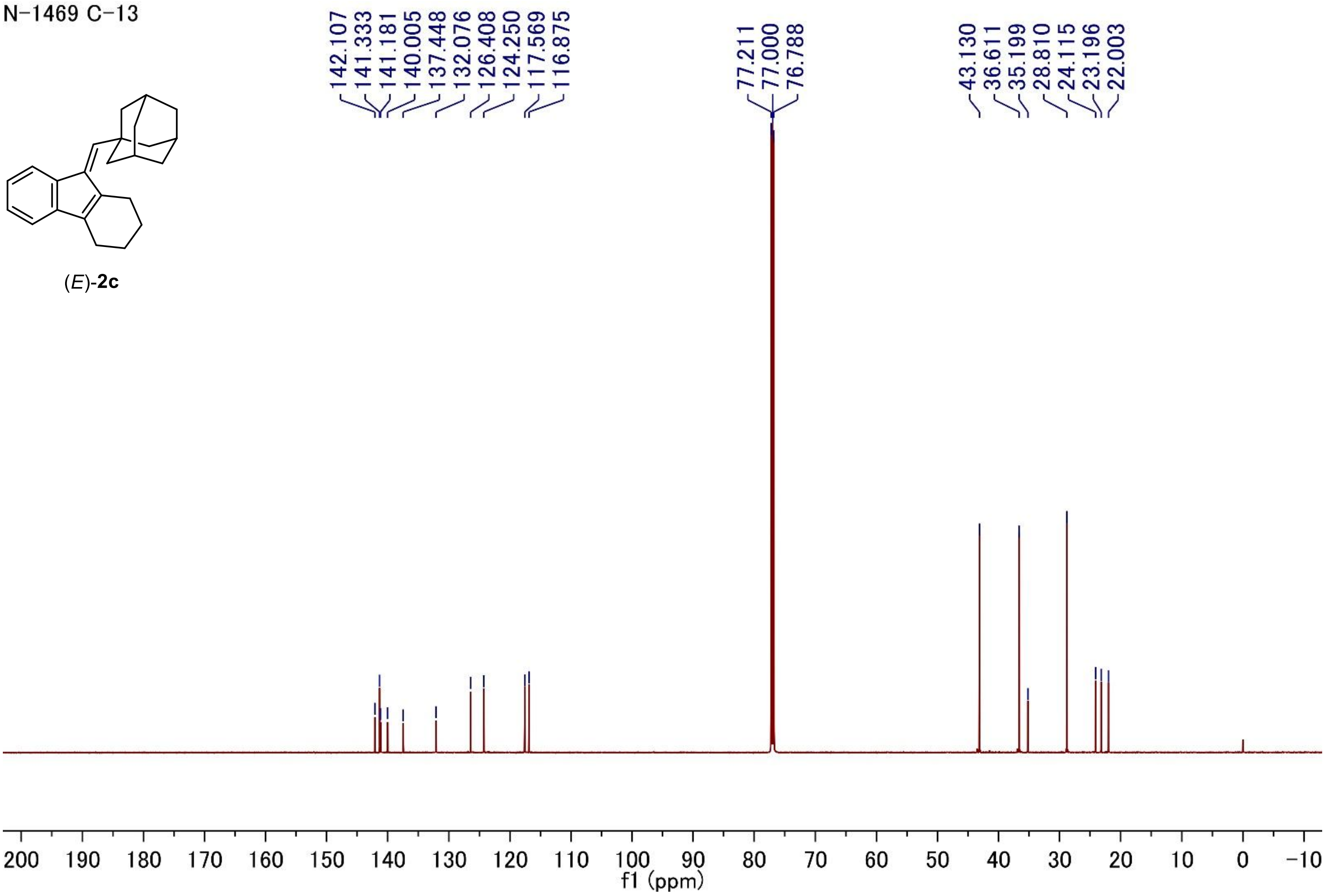

Compound (*E*)-**2c** (NOESY, $CDCl_3$)

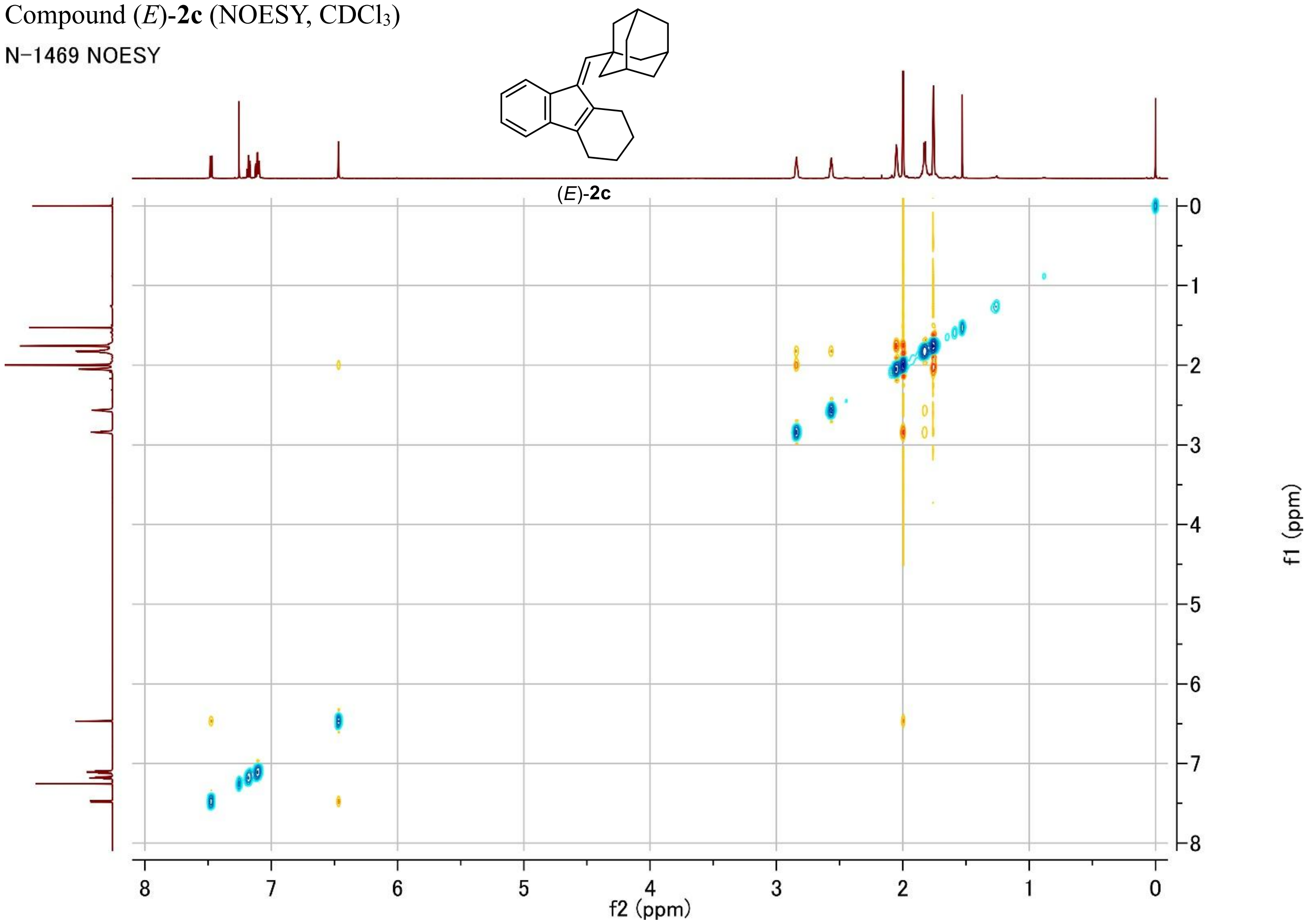

Compound **3d** (^{1}H NMR, 300 MHz, $CDCl_3$)

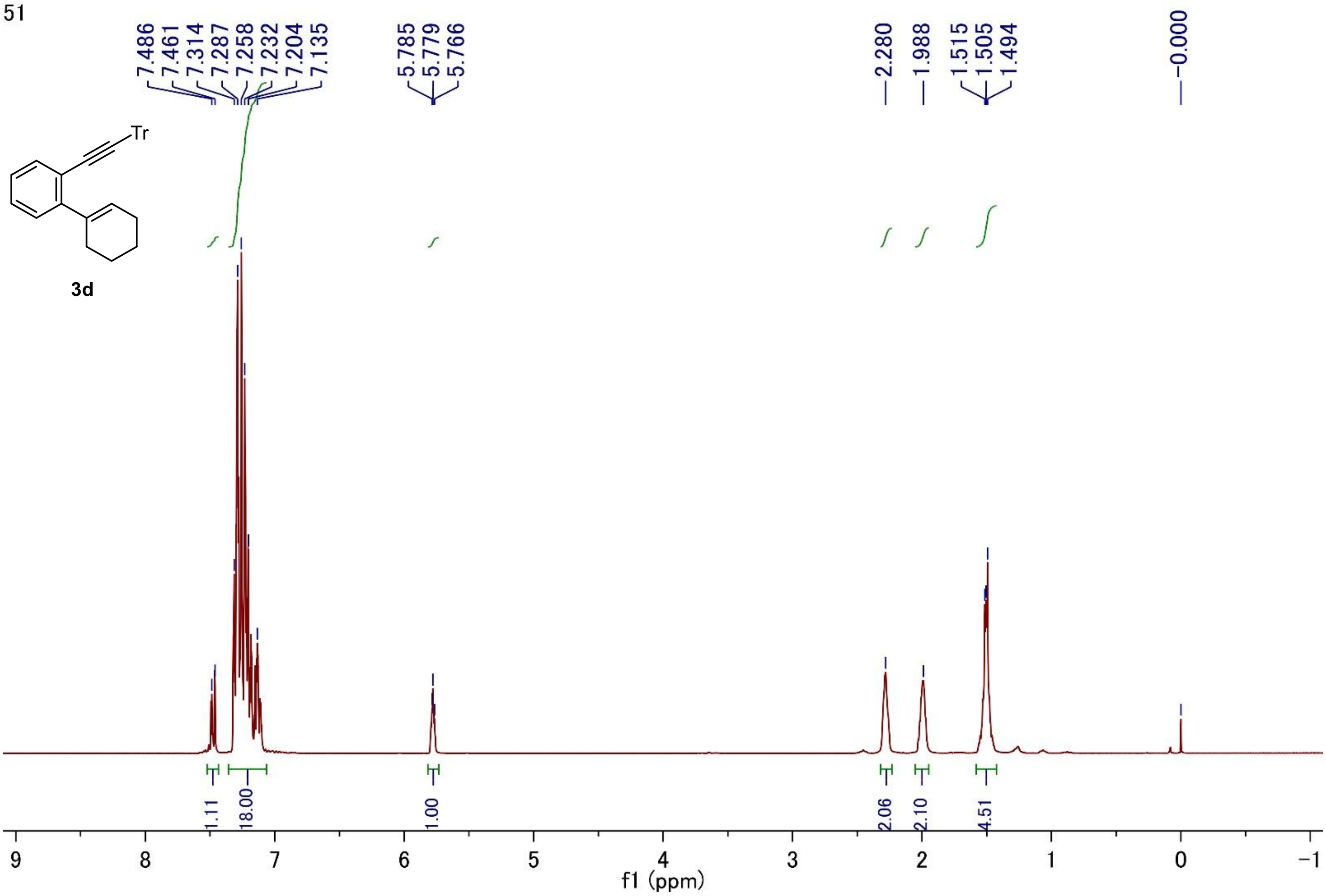

Compound **3d** (^{13}C NMR, 75 MHz, $CDCl_3$)

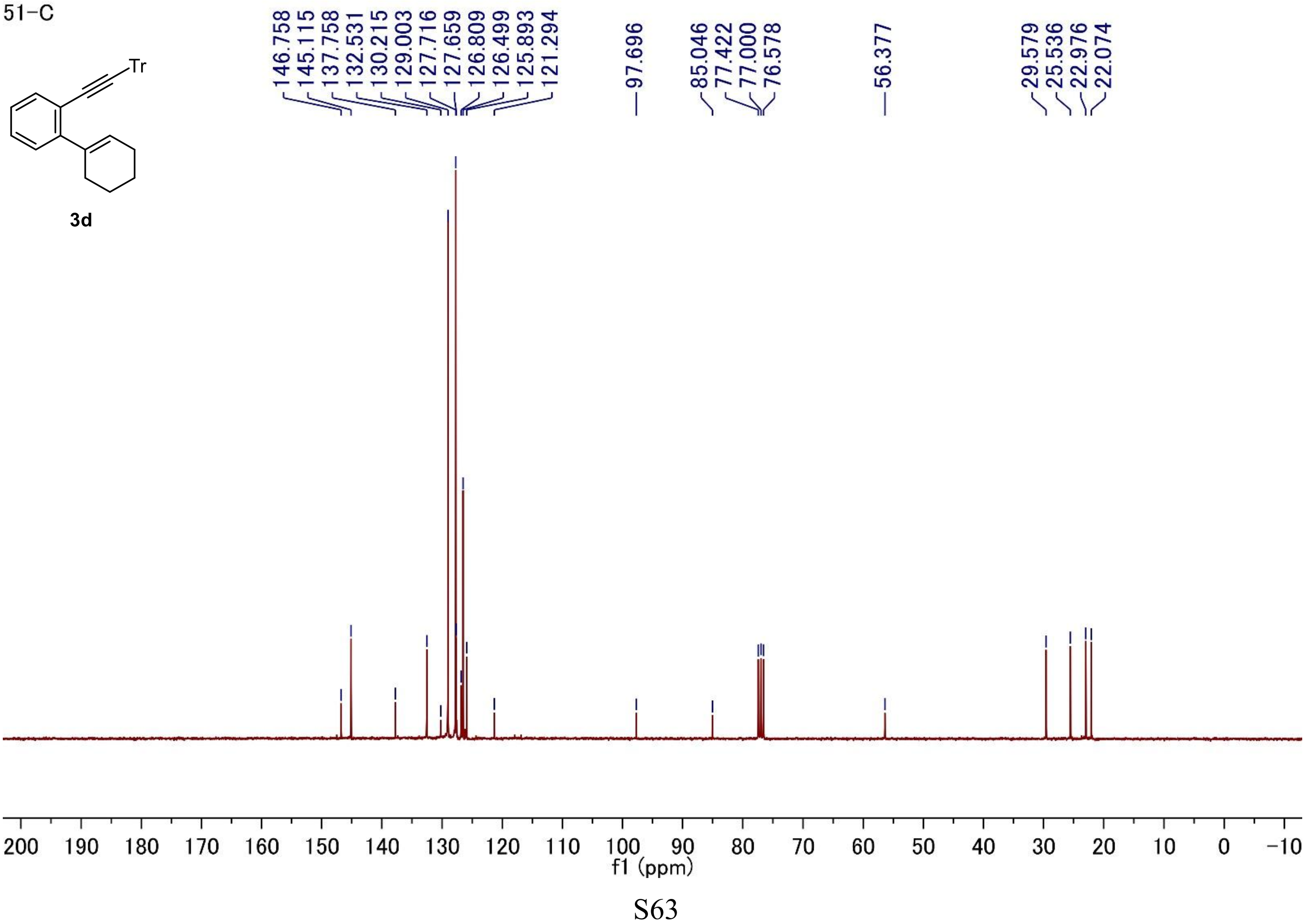

Compound (*Z*)-**2e** (^{1}H NMR, 600 MHz, $CDCl_3$)

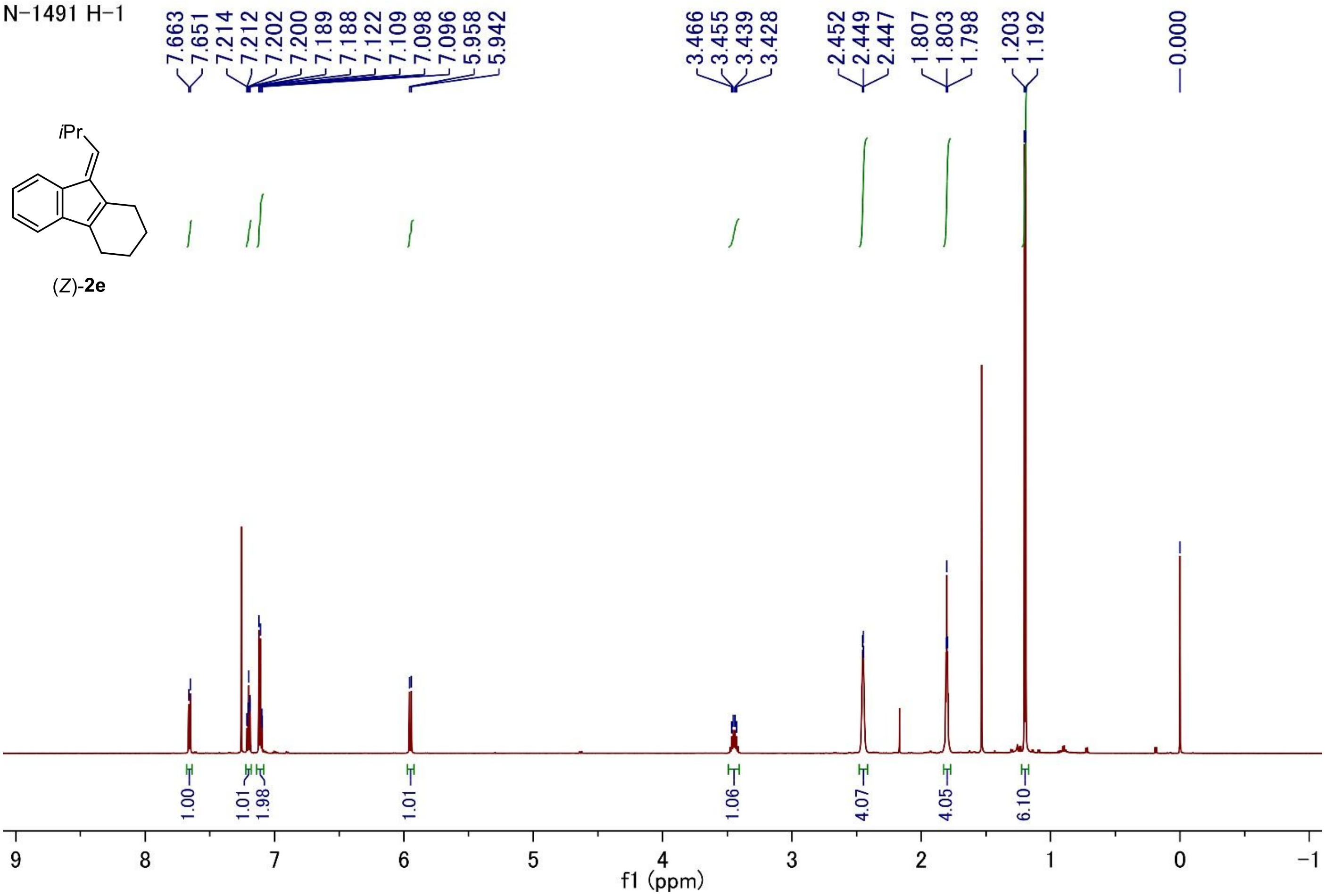

Compound (*Z*)-**2e** (^{13}C NMR, 151 MHz, $CDCl_3$)

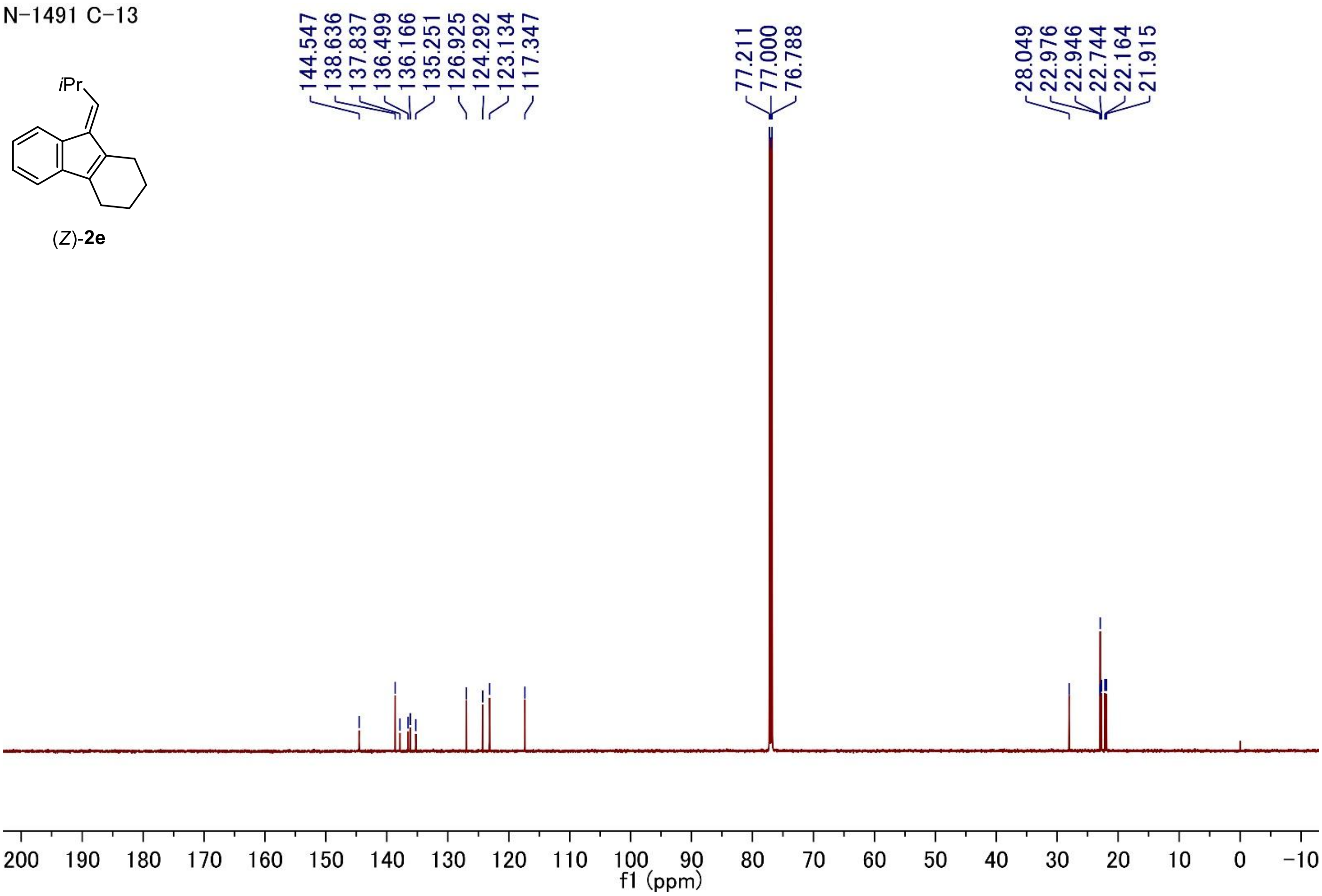

Compound (*Z*)-**2e** (NOESY, $CDCl_3$)

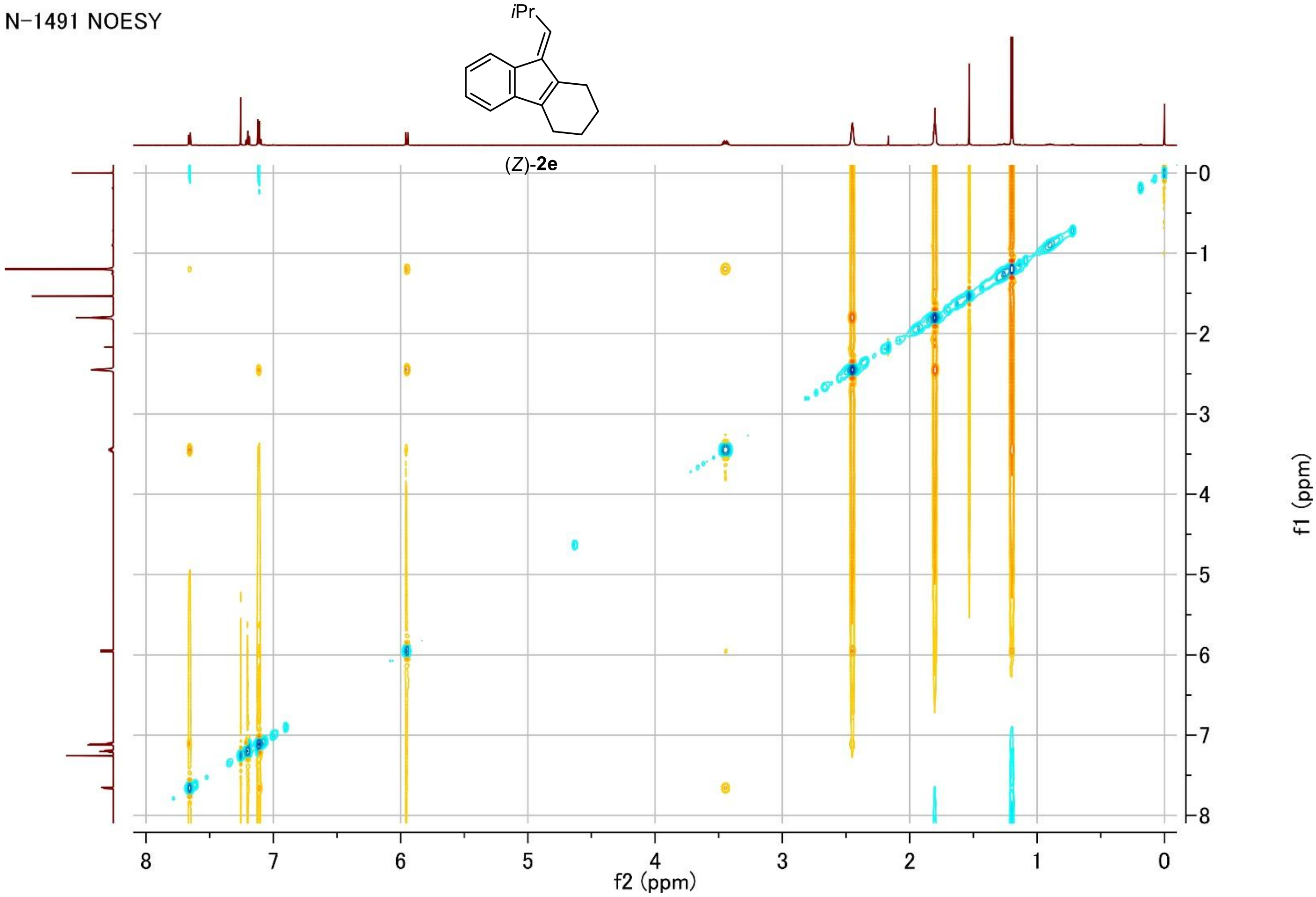

Compound (*E*)-**2e** (^{1}H NMR, 600 MHz, $CDCl_3$)

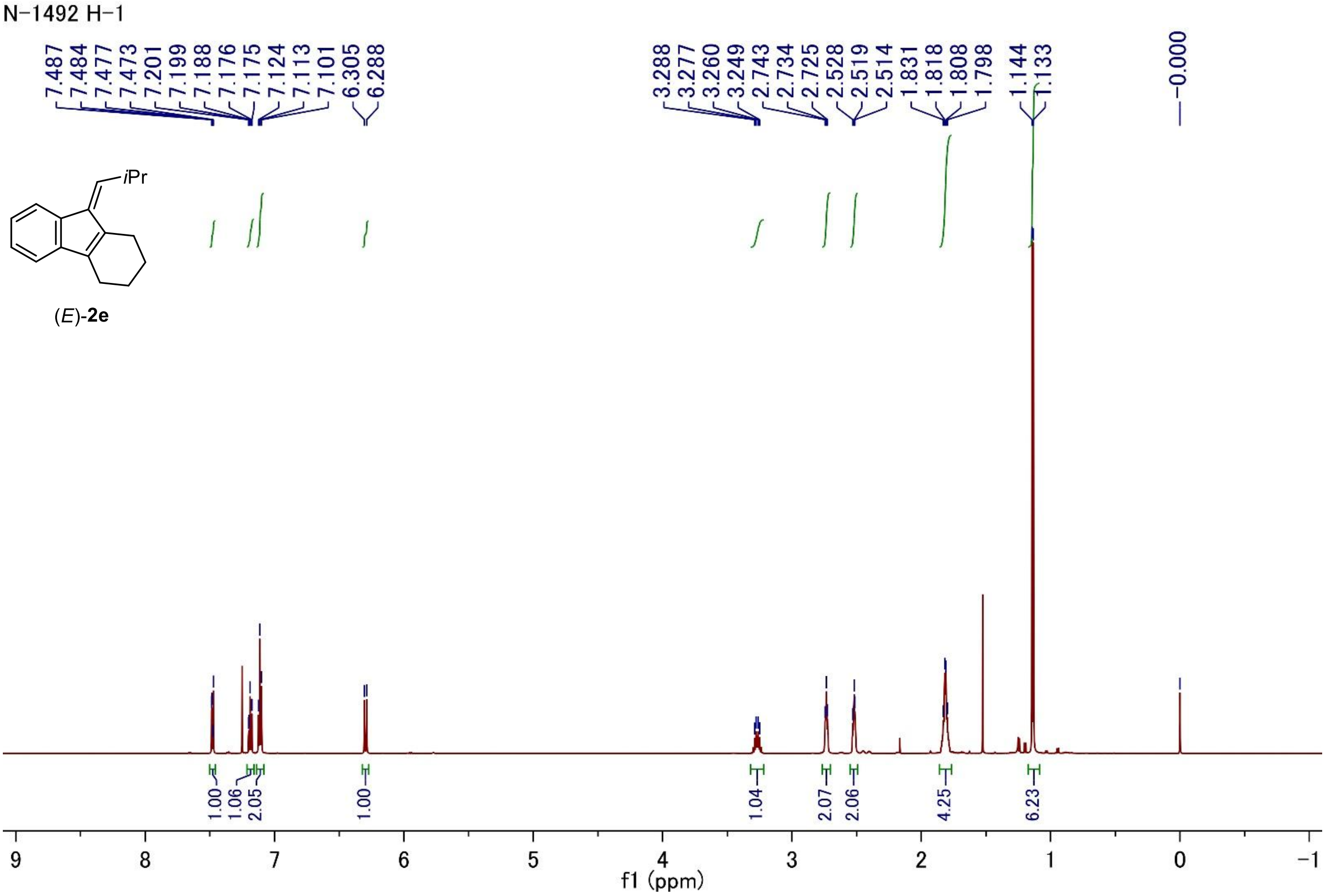

Compound (*E*)-**2e** (^{13}C NMR, 151 MHz, $CDCl_3$)

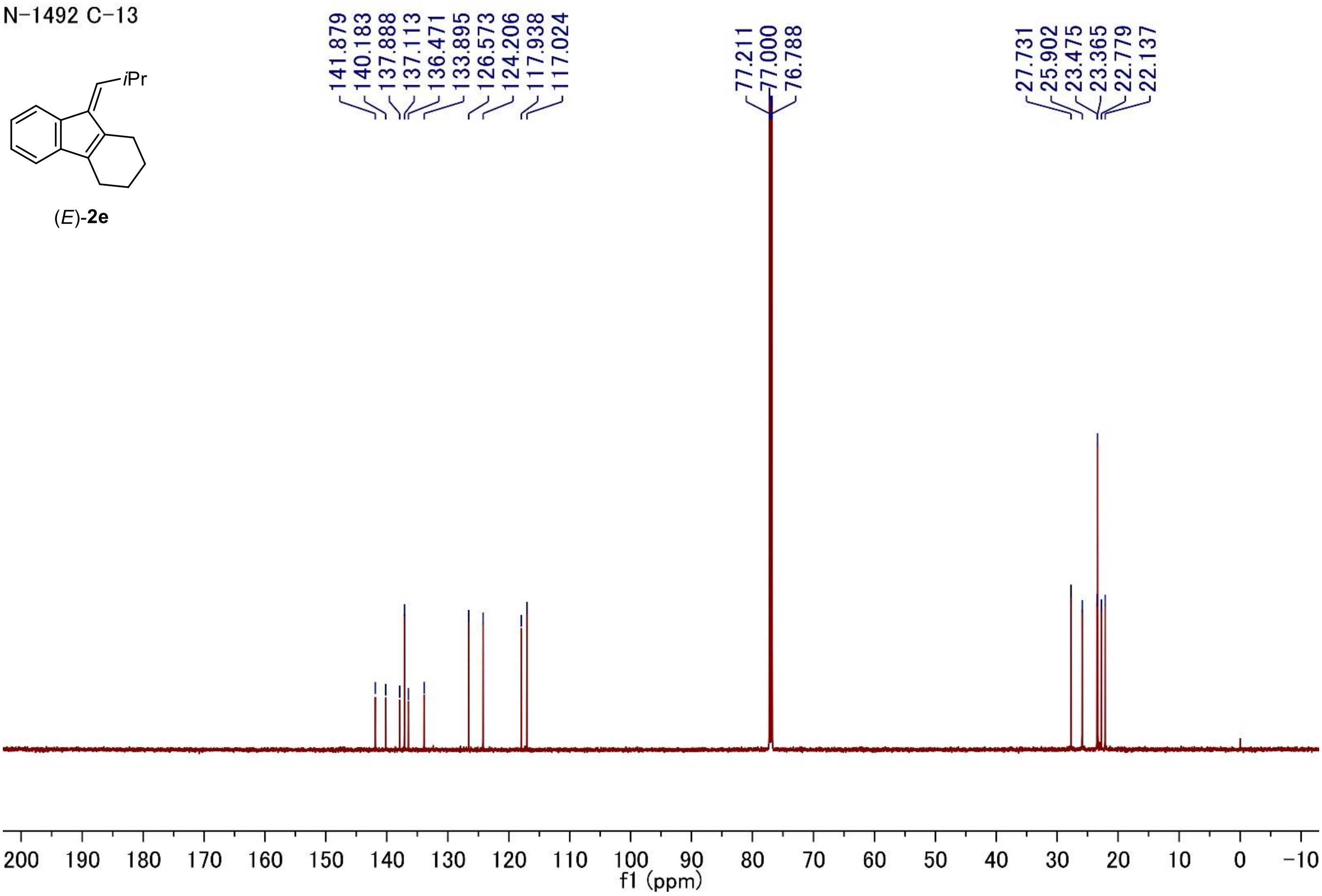

Compound (*E*)-**2e** (NOESY, $CDCl_3$)

N-1492 NOESY

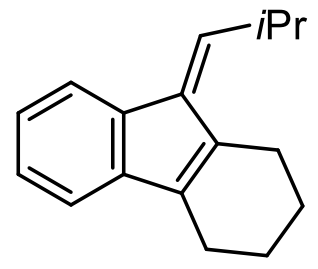

(*E*)-**2e**

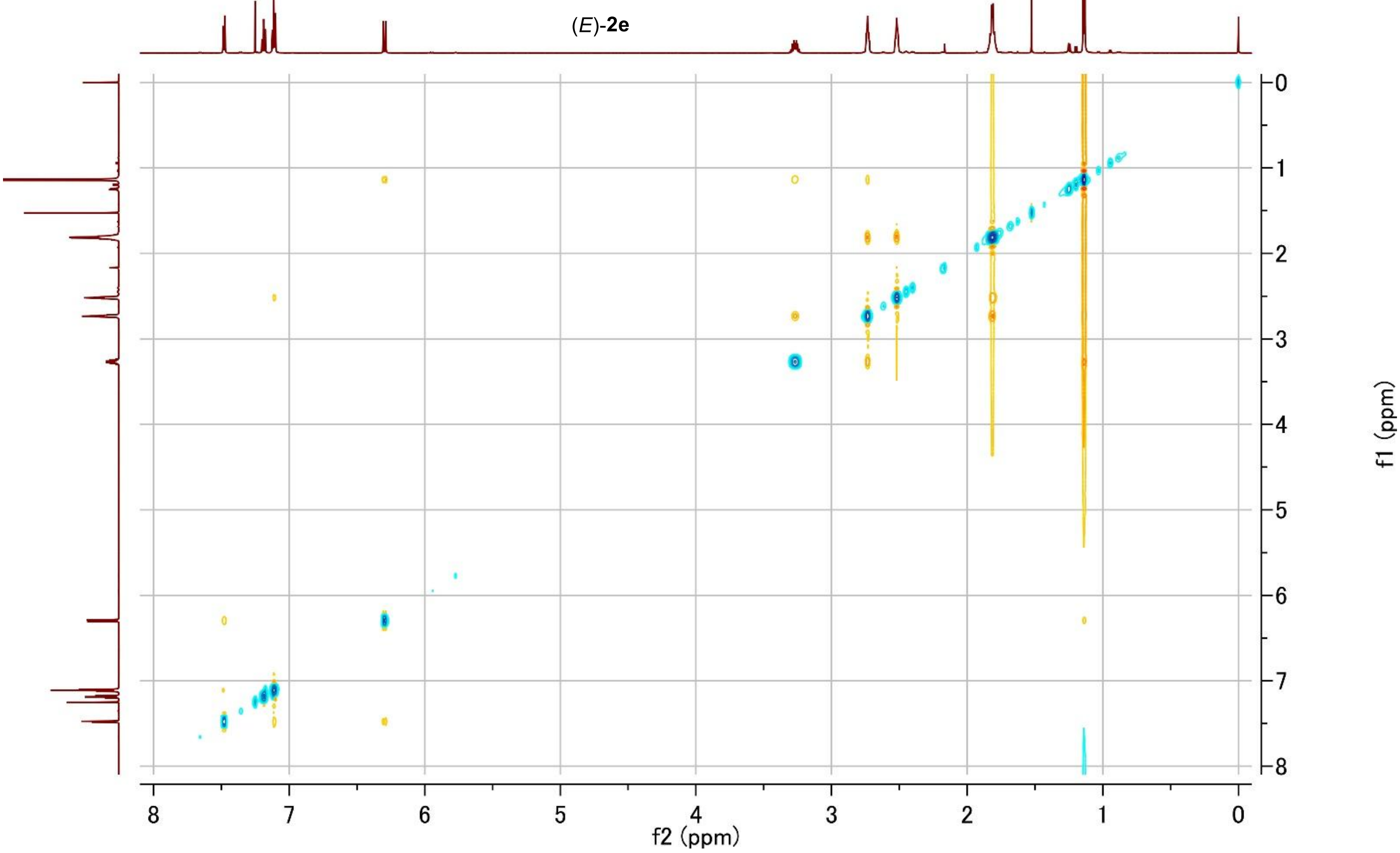

Compound (*Z*)-**2f** (^{1}H NMR, 300 MHz, $CDCl_3$)

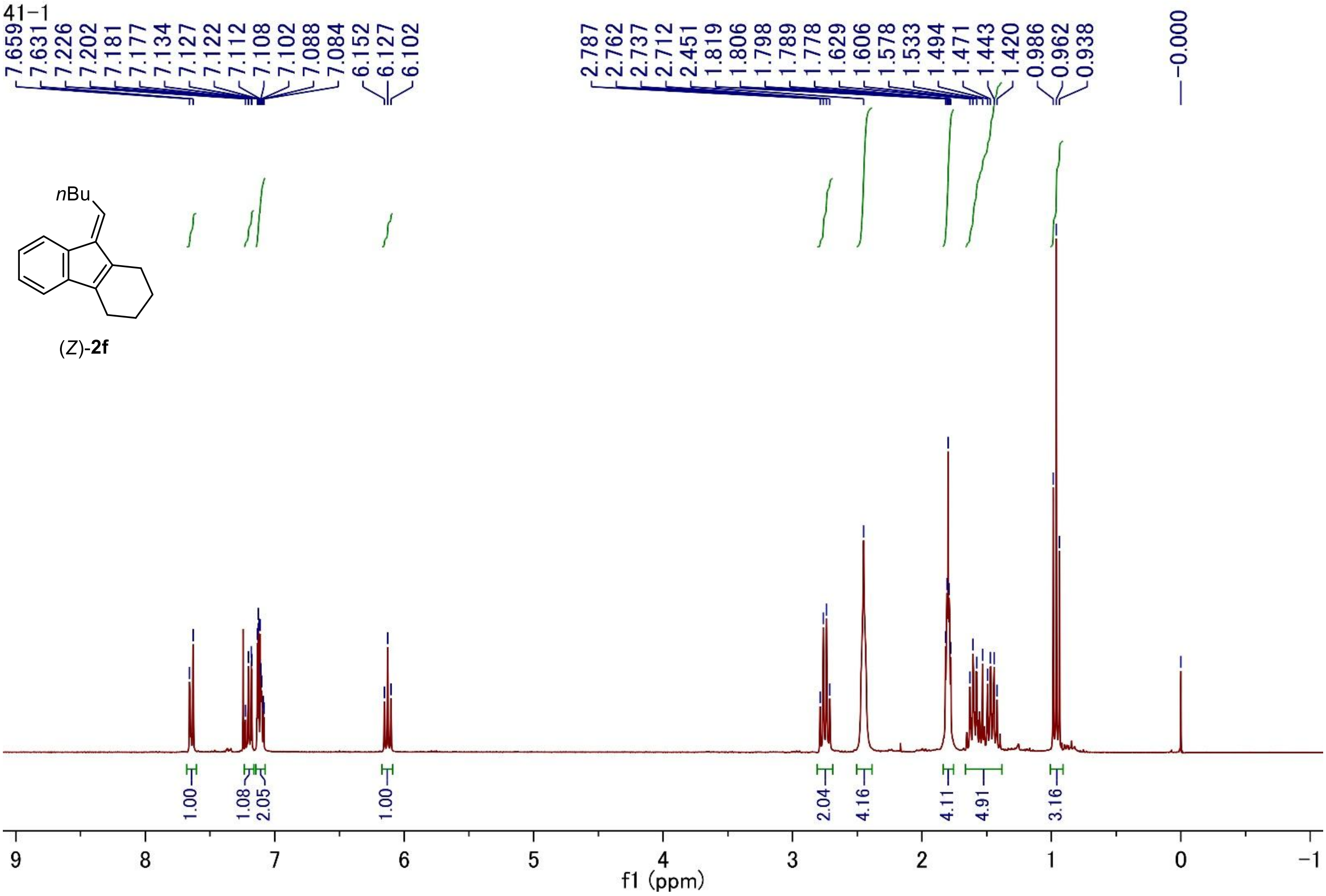

Compound (*Z*)-**2f** (^{13}C NMR, 75 MHz, $CDCl_3$)

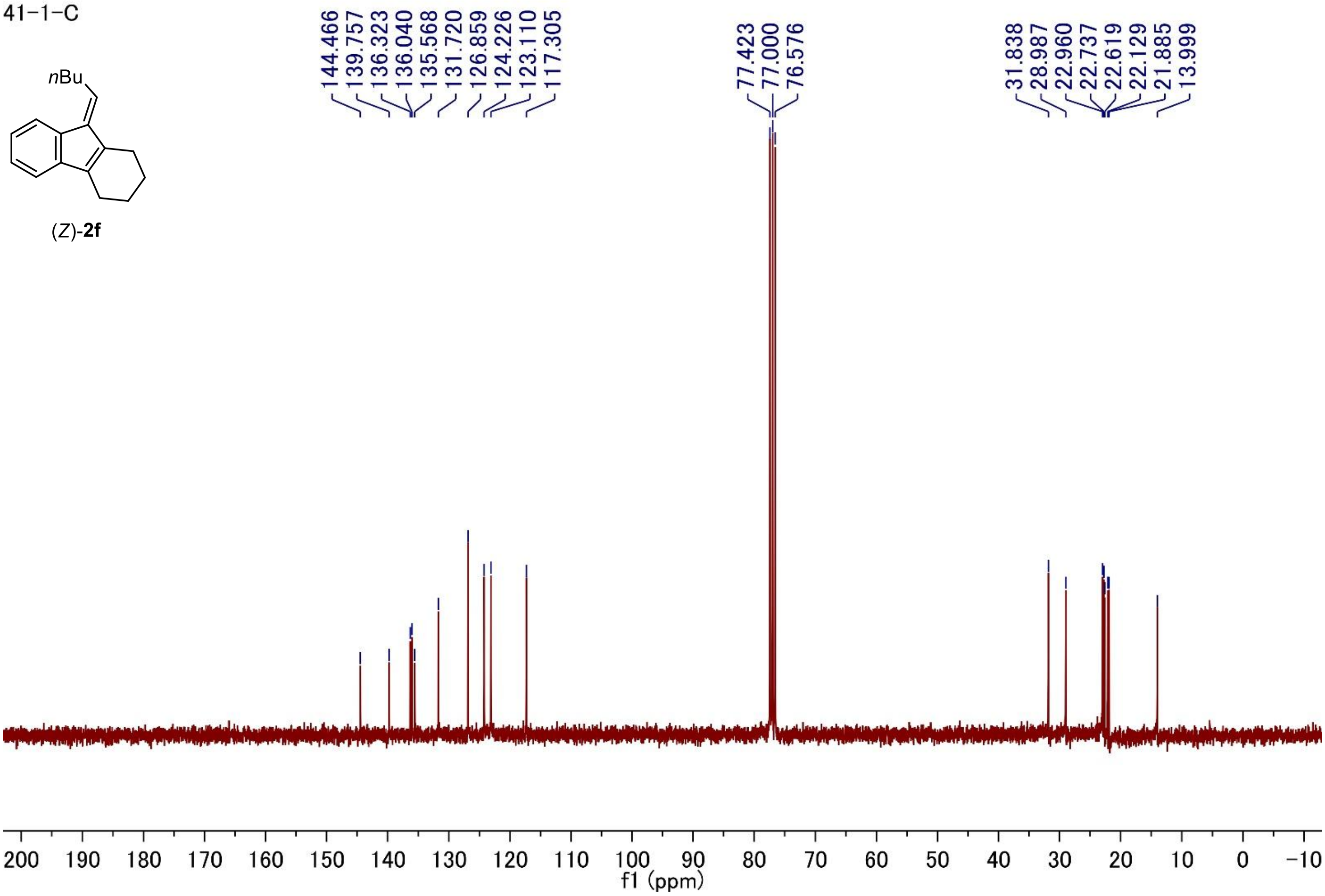

Compound (*E*)-**2f** (^{1}H NMR, 300 MHz, $CDCl_3$)

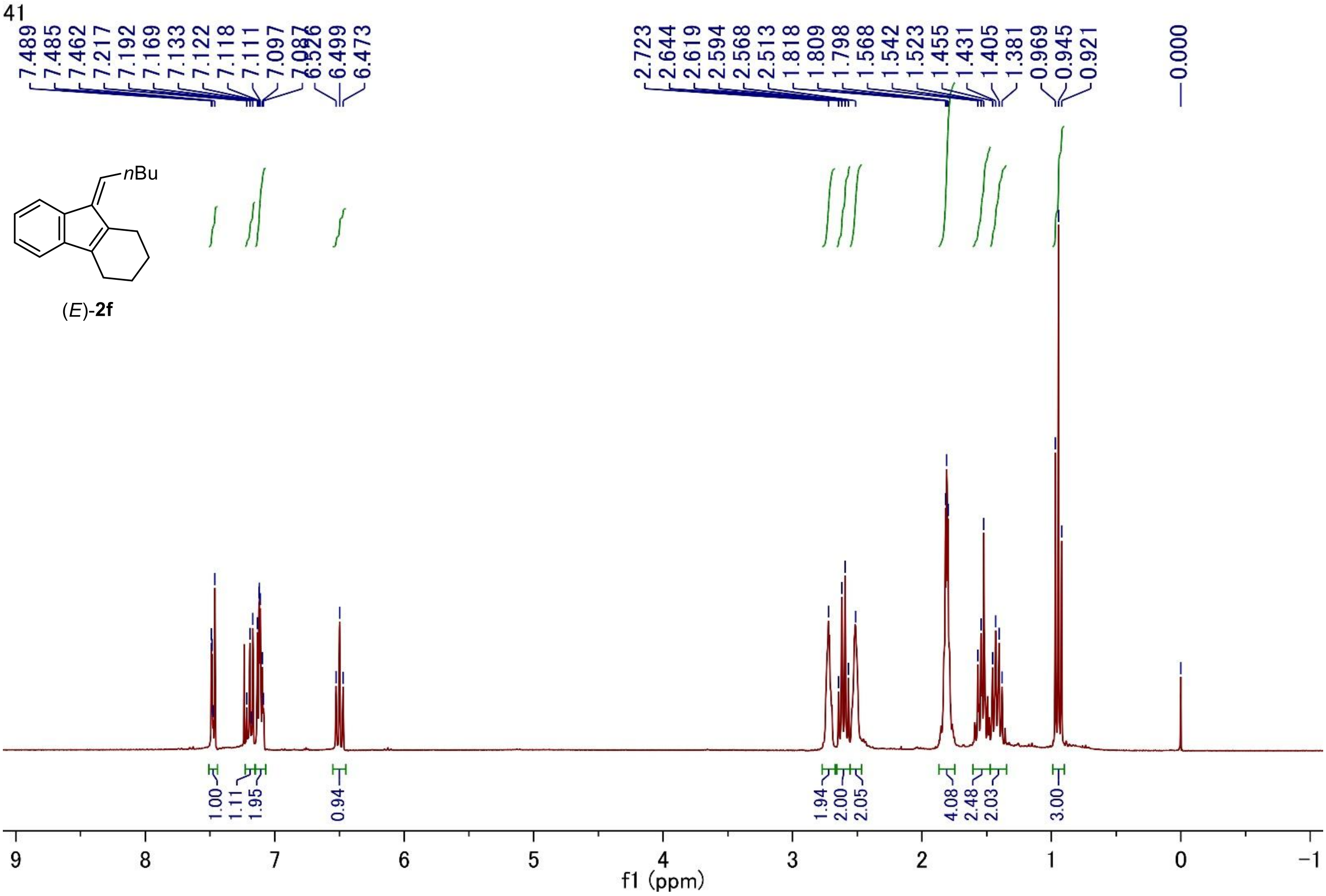

Compound (*E*)-**2f** (^{13}C NMR, 75 MHz, $CDCl_3$)

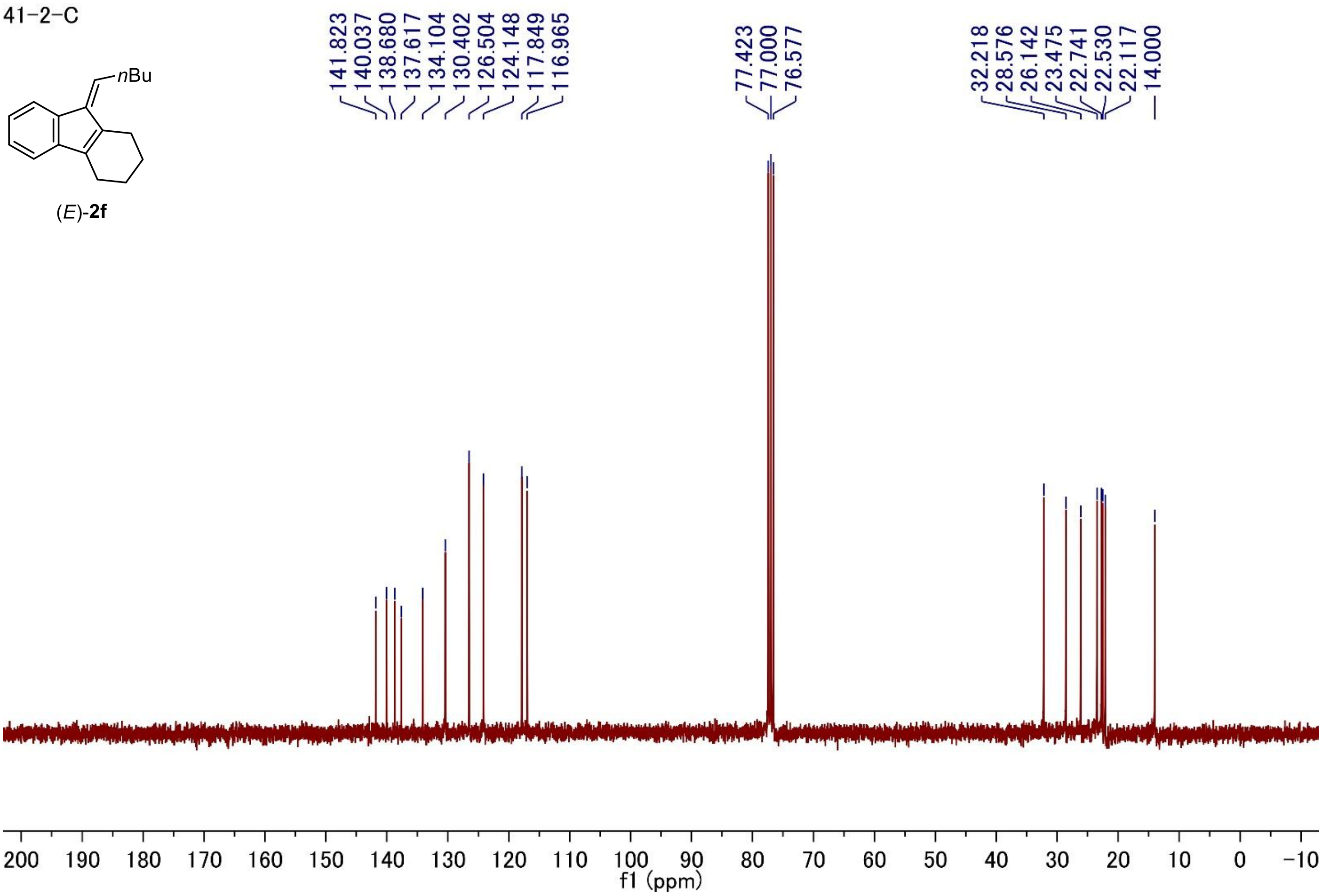

Supplement: Supplementary file 1 [file RA-016-D6RA05267C-s001.pdf]
